# Hydrogen-Bonding Activation of Gold(I) Chloride Complexes: Enantioselective Synthesis of 3(2*H*)-Furanones by a Cycloisomerization-Addition Cascade

**DOI:** 10.1021/acs.orglett.4c02091

**Published:** 2024-07-11

**Authors:** Pilar Elías-Rodríguez, Manuel Benítez, Javier Iglesias-Sigüenza, Elena Díez, Rosario Fernández, José M. Lassaletta, David Monge

**Affiliations:** † Facultad de Química, Departamento de Química Orgánica, Universidad de Sevilla and Centro de Innovación en Química Avanzada (ORFEO−CINQA), C/Prof. García González, 1, 41012 Sevilla, Spain; § Instituto de Investigaciones Químicas (CSIC-US) and Centro de Innovación en Química Avanzada (ORFEO−CINQA), Avda. Américo Vespucio, 49, 41092 Sevilla, Spain

## Abstract

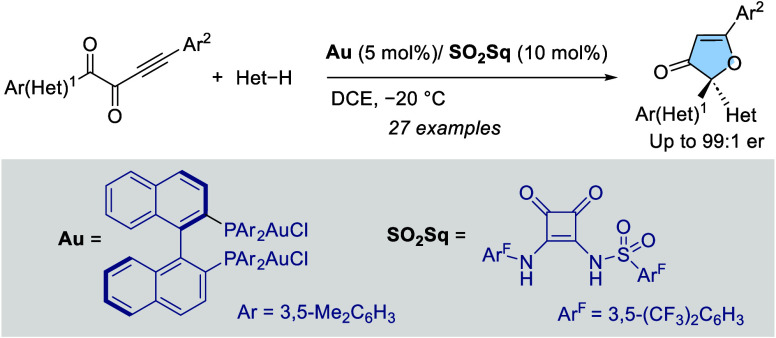

Enantioselective synthesis of 3(2*H*)-furanones has been achieved using the intermolecular H-bonding activation of gold(I) chloride complexes. A DM-BINAP [(*R*)-(+)-2,2′-Bis[di(3,5-xylyl)phoshino]-1,1′-binaphthyl] digold(I) dichloride complex in combination with a sulfonyl squaramide (SO_2_Sq) has been identified as the optimal catalytic system. The process involves a 5-*endo*-*dig* oxa-cyclization followed by stereocontrolled addition of indoles. Interestingly, the soft L*Au–Cl activation by H-bonding allowed the recovery of both L*Au–Cl and the activator after chromatographic purification.

Gold catalysis has become a powerful tool for the synthesis of heterocycles and complex organic molecules.^1^ However, enantioselective transformations have been proven to be not trivial due to inherent characteristics of gold complexes. Nevertheless, the smart design of ligands and implementation of ion-pairing strategies like asymmetric counterion-directed catalysis (ACDC) has allowed one to successfully develop highly enantioselective gold-catalyzed reactions.^2^ Although gold(I) chloride complexes [LAuCl] are the most readily available, stable, and cost-effective precatalysts, the inert character of the Au–Cl bond requires the use of activation strategies (Scheme 1). Typically, activation has been exerted by chloride abstraction employing a silver salt with a weakly coordinating anion (AgX), generating the active catalyst [LAu^+^]X^–^, along with silver chloride. However, silver salts^3^ or the counteranions introduced in the halide scavenging operation^4^ are frequently noninnocent in catalysis. To solve these limitations, several silver-free methodologies have been developed,^5^ such as the use of alkali metal borates (NaBAr^F^_4_), among other metal salts as chloride scavengers,^6^ or protic solvents such as hexafluoroisopropanol (HFIP).^7^ Other appealing strategies include halogen-,^8^ hydrogen-,^9^ and carbon-bonding activations.^10^ In contrast to metal-activated reactions, these dynamic gold strategies, where the cationic active gold species are in equilibrium with the inactive gold chloride complexes [LAuCl], provide soft activations with potential recyclability of the gold precatalyst.

**Scheme 1:**
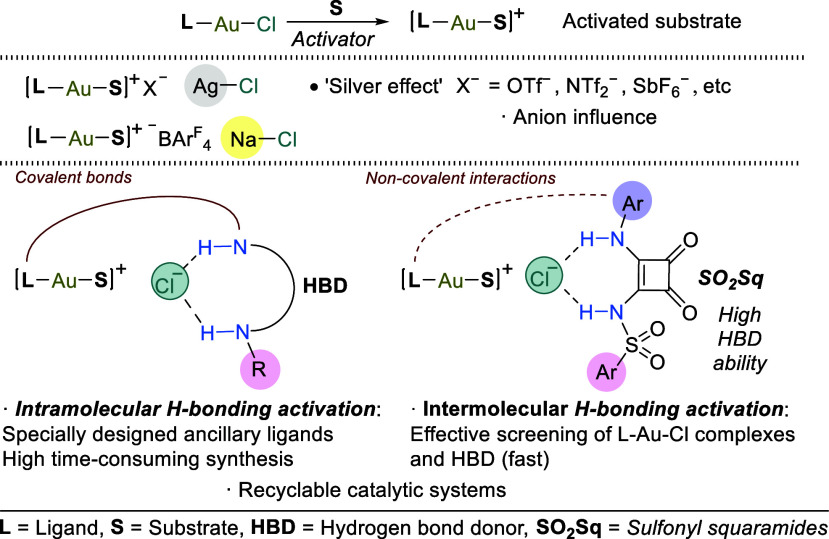
Activations of [LAuCl] via Silver Salts, Borate Salts, and H-Bond Donors in Intra- and Intermolecular Fashion

Regarding H-bonding activation, great efforts have been devoted to the multistep-synthesis of specifically designed ancillary phosphine or NHC ligands bearing mono- and bidentate H-bond donors (HBD), which enabled self-activation of gold(I) chloride complexes (intramolecular H-bonding activation). In the search for gold(I)-catalyzed enantioselective transformations,^2^ some related bifunctional catalysts have been successfully implemented. Marinetti, Guinchard, and co-workers developed a catalyst featuring a phosphine Au(I) chloride complex covalently bound to a chiral phosphoric acid (tethered ACDC), enabling different asymmetric transformations with or without Ag(I) additives.^11^ Alternatively, Echavarren, Franchino, and co-workers combined urea or squaramide/phosphine Au(I) chloride complexes with a BINOL-derived Ag(I) phosphoramidate (H-bonded ACDC) for diverse asymmetric cyclizations.^12^ However, to avoid possible silver effects, further approaches for silver-free activations are still necessary for some enantioselective transformations. We have recently reported that highly acidic sulfonyl squaramides (SO_2_Sq) allowed H-bonding activations in intermolecular fashion, enabling several gold-catalyzed heterocyclizations.^9c^ In this Communication, we present the first implementation of this strategy in enantioselective gold(I) catalysis. The following advantages might be envisioned: (i) Fast and versatile optimization by multiscreening of complexes containing commercially available chiral ligands in combination with a library of HBD activators, avoiding multistep synthesis required for accessing bifunctional tethered complexes and (ii) recyclability of precatalysts and activators.

The Au(I)-catalyzed cycloisomerization-addition cascade reaction between indole-tethered ynediones **1** and indoles **2** was chosen as a platform to evaluate the usefulness of our approach. In this challenging transformation, only developed in the racemic version,^13^ it is expected that intermolecular H-bonding activation of a chiral gold chloride complex [L*Au–Cl] facilitates a 5-*endo*-*dig*-oxa-cyclization of **1**, forming a stabilized carbocationic gold intermediate which might undergo 1,4-addition of indoles **2**, enantiocontrolled by a chiral phosphine (L*), generating biologically relevant 3(2*H*)-furanones^14^**3** containing quaternary stereocenters in enantioenriched form (Scheme 2).

**Scheme 2:**
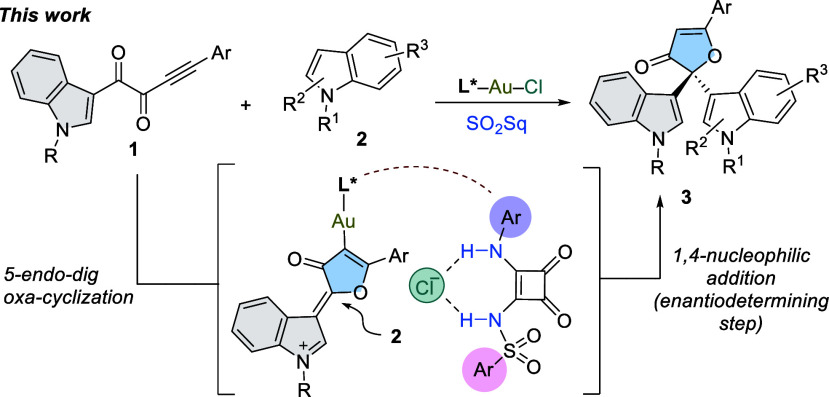
Enantioselective Synthesis of 3(2*H*)-Furanones Using [L*-Au–Cl]/SO_2_Sq as a Catalytic System

Preliminary experiments were conducted for the model cycloisomerization-addition cascade between aromatic ynedione **1A** and indole (**2a**) (Table 1). Employing 5 mol % of BINAP-derived gold(I) complex **Au1**, performed in situ without any activator, product **3Aa** was formed in 90% yield after 16 h in 1,2-dichloroethane at room temperature, albeit as a racemic form (entry 1). However, erratic results were collected by using different batches of Me_2_SAuCl or slightly different reaction times for the preparation of **Au1**.

**Table 1:**
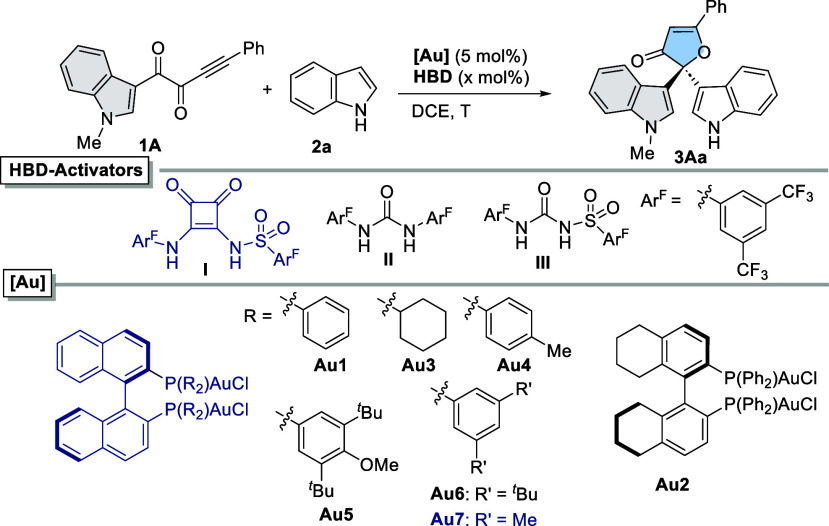
Optimization of the Model Reaction between **1A** and **2a**^a^

Entry	[Au]	Activator	*T* [°C]	Yield [%]^b^	er^c^
1	**Au1**^d^		rt	90	rac.
2^e^	Me_2_SAuCl		rt	>95	rac.
3	**Au1**		rt	15	rac.
4	**Au1**		–20	<5	nd
5	**Au1**	AgSbF_6_^f^	–20	>95	rac.
6		AgSbF_6_^f^	–20	>95	rac.
7	**Au1**	**I**^f^	–20	72	65:35
8	**Au1**	**I**	–20	>95	70:30
9	**Au1**	**II**	–20	44	65:35
10	**Au1**	**III**	–20	73	70:30
11	**Au2**	**I**	–20	83	67:33
12	**Au3**	**I**	–20	16	65:35
13	**Au4**	**I**	–20	>95	72:28
14	**Au5**	**I**	–20	30	62:38
15	**Au6**	**I**	–20	13	63:37
16	**Au7**	**I**	–20	>95	86:14
17	**Au7**	NaBAr^F^_4_	–20	>95	81:19

a Reactions were performed employing **1A** (0.2 mmol), **2a** (0.2 mmol), [Au] (5 mol %), and activator (10 mol %) for 16 h. Au complexes purified by flash chromatography.

b Determined by ^1^H NMR using 1,3,5-trimethoxybenzene as internal standard. An error of ≈5% is assumed.

c Determined by HPLC analysis.

d **Au1** complex performed in situ.

e Reaction time: 6 h.

f 5 mol %.

A control experiment employing 5 mol % of Me_2_SAuCl afforded **3Aa** in quantitative yield after 6 h (entry 2). Purification of **Au1** by flash chromatography prior to use led to reduced catalytic activity (15% yield after 16 h, entry 3), suggesting that irreproducibility issues might be due to catalysis by variable trace amounts of Me_2_SAuCl in preliminary experiments. Cooling to −20 °C practically inhibited the reaction (<5% of **3Aa**, entry 4). Therefore, this temperature was set to analyze the activation of gold(I) complexes by chloride abstraction. The use of AgSbF_6_ as a chloride scavenger provided the desired activation, albeit without enantioinduction (entry 5). Indeed, in a control experiment without **Au1**, the same result was obtained (entry 6), revealing a silver-catalyzed nonselective pathway. Next, HBDs were tested as activators. To our delight, **Au1** in combination with an equimolecular amount of sulfonyl squaramide (SO_2_Sq) **I** (5 mol %) afforded **3Aa** in 72% yield and 65:35 er (entry 7). This result could be further improved up to quantitative yield and 70:30 er by using 10 mol % of **I** (entry 8).^15^ Urea **II** and sulfonyl urea **III** were less efficient activators (entries 9 and 10), showing that the higher acidity of **I** is key for an efficient Au–Cl labilization by H-bonding.^9c^ Next, different chiral gold complexes **Au1**–**Au18** containing commercially available phosphorus-based ligands featuring central-, planar-, and axial chirality were evaluated.^16^ From this screening, digold(I) dichloride complexes from BINAP-type ligands emerged as the most promising precatalysts. Notably, the use of biphenyl instead of binaphthyl scaffolds led to lower enantioselectivities. As a representative example, partially hydrogenated H8-BINAP-derived complex **Au2** afforded **3Aa** in 83% yield with 67:33 er (entry 11). Then, we studied the influence of the phosphine groups (**Au3**–**Au7**). Remarkably, **Au3**, bearing a bulkier and better σ-donor PCy_2_ group, afforded a lower yield (16%) and enantioselectivity (65:35 er) than those of **Au1** (entry 12). Complex **Au4**, containing bis(*p*-tolyl)phosphine groups (Tol-BINAP), provided **3Aa** in quantitative yield and a slightly better 72:28 enantiomeric ratio (entry 13). Conversely, bulkier complexes **Au5** and **Au6**, bearing bulkier aryl groups (4-methoxy-3,5-di-*tert*-butylphenyl and 3,5-di-*tert*-butylphenyl, respectively), exhibited lower catalytic activities and enantioselectivities (entries 14 and 15). Finally, the use of **Au7**, based on DM-BINAP [(*R*)-(+)-2,2′-Bis[di(3,5-xylyl)phoshino]-1,1′-binaphthyl], led to the best enantioselectivity, furnishing **3Aa** in quantitative yield with 86:14 er (entry 16). Additionally, complex **Au7** was subjected to activation by NaBAr^F^_4_, achieving the same level of chemical efficiency, albeit in a lower enantioselectivity (81:19 er, entry 17).^17^ This result highlights the suitability of the disclosed activation by SO_2_Sq **I** for the development of this enantioselective transformation. To further explore the catalytic performance of the optimized system, several possibilities were taken into account for the design of an additional set of sulfonyl squaramides/ureas: (i) Introduction of naphthyl and biaryl motives as in **IV**, **V**, and **VI**, which might project π systems to more distant areas; (ii) incorporation of additional acidic H-bond donors, such as phenolic OH (**VIII**) or sulfonamide NH groups (**VII**, **IX**, and **X**), to assist in the gold(I)–Cl labilization process. Thus, SO_2_Sqs **IV**–**X** were synthesized^16^ and evaluated under optimized conditions (Table 2). The substitution of the 3,3′-bis-trifluoromethylphenyl group attached to NH in **I** by other aryl groups had a negative impact on the catalytic activity and also on the enantioselectivity. The results allowed us to establish a direct correlation between the hydrogen-bond-donating capability of the catalysts (**I** > **IV** > **V** > **VI**; as inferred from the chemical shifts of the Ar–N**H** protons in ^1^H NMR spectra)^16^ and the obtained chemical yields, which progressively dropped to 28% and 18% for less acidic SO_2_Sq **V** and **VI**, respectively. SO_2_Sq **VII**, which incorporates an additional sulfonamide NH at the ortho position of the lower ring of the biphenyl moiety, enabled activation, affording **3Aa** in 70% yield and 85:15 er. Other triple H-bond donors were evaluated. Phenolic-type **VIII** was less active than sulfonamide-type **IX**, albeit the enantioselectivity remained lower than that obtained with SO_2_Sq **I**. Finally, sulfonylurea **X** provided a poor yield of 14%, also highlighting the importance of squaramide geometry to exert this activation. With the best catalytic system **Au7**/SO_2_Sq (**I**), further optimization of the solvent and temperature was carried out. CH_2_Cl_2_ and toluene were compatible solvents, although slightly lower enantioselectivities were obtained (entries 2 and 3). HFIP^18^ promoted the present transformation without any other activator, but no enantioselectivity was observed (entry 4). In the search for a more enantioselective process, lower temperatures were evaluated in different solvents. However, solubility issues hampered further optimization. As an example, the reaction in DCE at −30 °C afforded **3Aa** in 33% yield and 85:15 er (entry 5). We next explored the scope of the reaction (Scheme 3). Regarding the ynedione, *N*-methylpyrrol-tethered substrate **1B** was tolerated, affording (*S*)-**3Ba** in 82% yield and 86:14 er. On the contrary, the presence of a furan ring had a detrimental effect on the enantioselectivity, yielding (*S*)-**3Ca** in 62:38 er. It is worth mentioning that phenyl-bearing ynedione (Ar^1^ = Ph) remained unreactive, while the electron-rich *p*-methoxy-phenyl ring (**1D**, Ar^1^ = 4-MeO-C_6_H_4_) afforded (*R*)-**3Da** in 99% yield, albeit with modest enantioselectivity (64:36 er). *N*-Benzyl- and *N*-methyl-indoles were tolerated, ruling out the necessity of an NH group in the nucleophile to assist the gold(I) chloride activation.^19^ Thus, (*R*)-**3Ab** and (*S*)-**3Ec** were synthesized in excellent yields (>97%) and enantiomeric ratios of 87:13 and 85:15, respectively. Noteworthy, these two enantiomers were accessible simply by exchanging the benzyl and the methyl chains between the substrate and the nucleophile, consistent also with a uniform stereochemical reaction pathway. Next, a set of unprotected and *N*-protected indoles was made to react with various indole- and pyrrole-tethered ynediones **1A-I**. Substitution at C2 (R^2^) by methyl and phenyl groups allowed the proper reaction, which is slower for R^2^ = Ph (36–40 h), providing the desired products (*R*)-**3Ad**-**g** in good-to-excellent yields (89–95%) and good enantioselectivities (83:17–89:11 er). 3-Methylindole (**2h**) also underwent a smooth transformation, affording (*R*)-**3Ah** in moderate yield (55%) but higher enantioselectivity (91:9 er). The effect of indole substitution at 4-, 5-, and 7-positions (R^3^) was also investigated. Electron-donating (R^3^ = OMe at C4) and electron-withdrawing formyl (R^3^ = CHO at C5) substituents provided (*S*)-**3Ai** and (*S*)-**3Ak** in excellent yields (91–92%), without alterations of stereochemical outcomes (86:14–88:12 er).

**Scheme 3:**
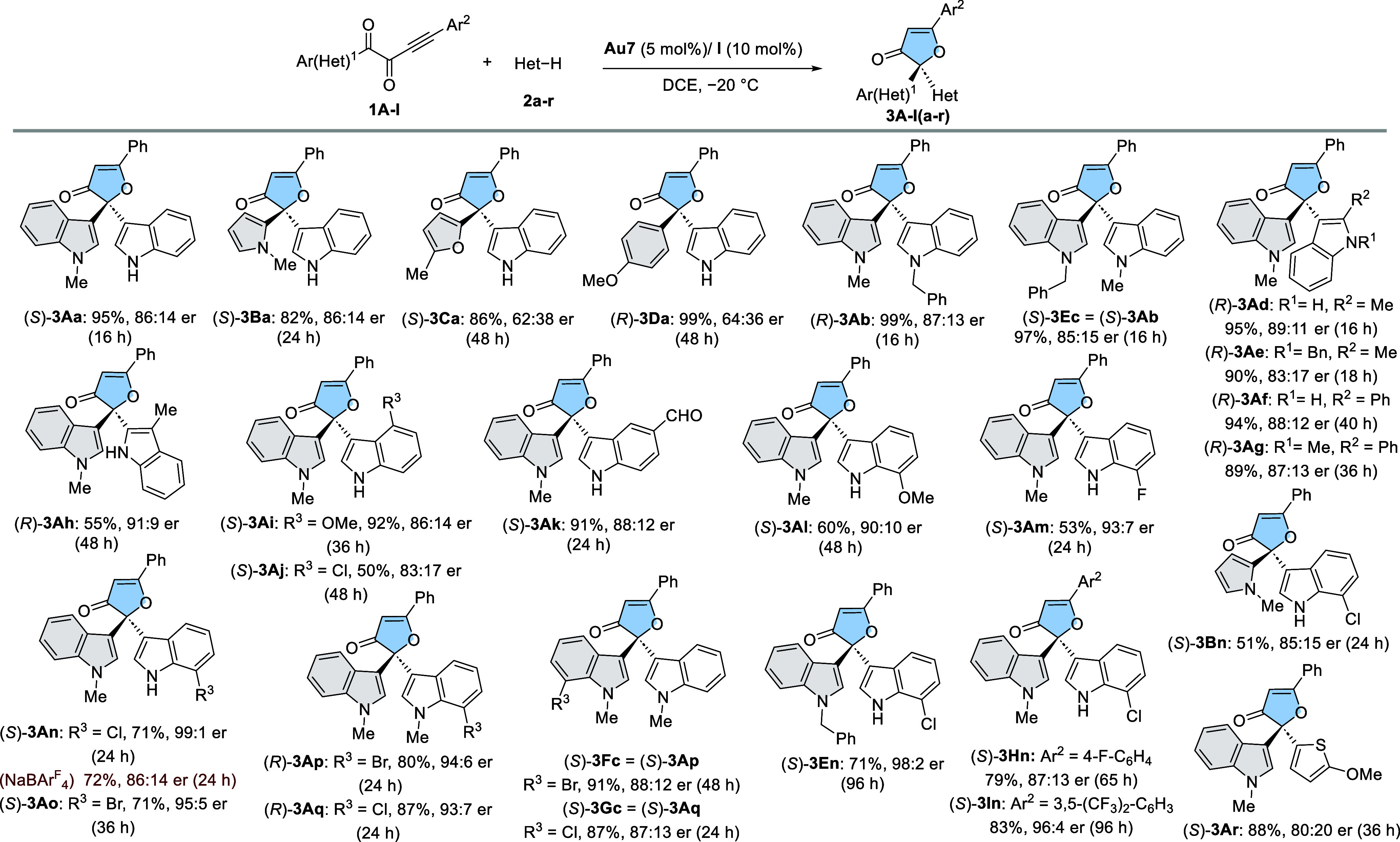
Scope of the Reaction Reactions performed at 0.2 mmol scale. Highlighted in gray aryl (Ar^1^)/heteroaryl scaffolds from ynediones **1**. Yields of isolated products after column chromatography. Enantiomeric excesses were determined by HPLC on chiral stationary phases.

**Table 2:**
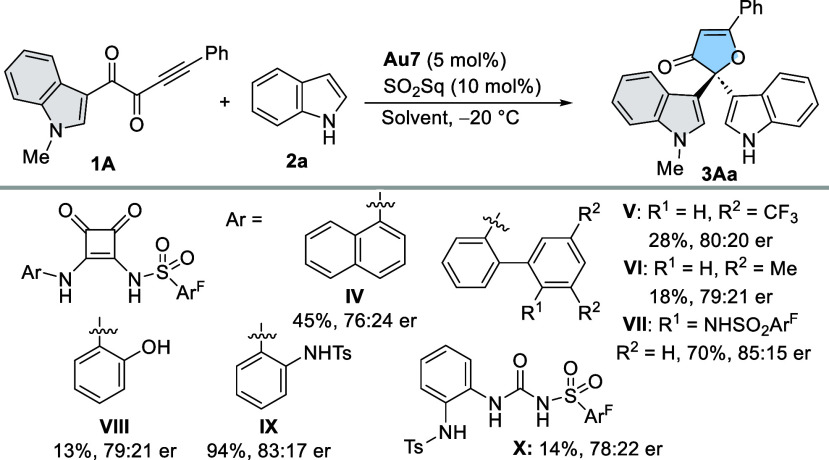
Evaluation of Additional Sulfonyl Squaramides (SO_2_Sq) and Optimization of Other Reaction Parameters^a^

Entry	HBD	Solvent	Yield [%]^b^	er^c^
1	**I**	DCE	>95	86:14
2	**I**	CH_2_Cl_2_	>95	82:18
3	**I**	Toluene	>95	80:20
4^d^		HFIP	80	rac.
5^e^	**I**	DCE	33	85:15

a Reactions were performed at 0.2 mmol scale employing **Au7** (5 mol %) and activator (10 mol %) in DCE for 16 h.

b Determined by ^1^H NMR using 1,3,5-trimethoxybenzene as internal standard. An error of ≈5% is assumed.

c Determined by HPLC analysis.

d Reaction temperature = 0 °C.

e –30 °C.

4-Chloro substituted indole reacted more slowly (48 h), providing (*S*)-**3Aj** in 50% yield and 83:17 er. Substitution at C7 turned out to be optimal in terms of enantioinduction, affording (*S*)-**3Al** (R^3^ = OMe), (*S*)-**3Am** (R^3^ = F), (*S*)-**3An/**(*R*)-**3Aq**/(*S*)-**3En** (R^3^ = Cl), and (*S*)-**3Ao**/(*R*)-**3Ap** (R^3^ = Br), with variable yields (53–87%) and enantioselectivities from good to excellent (90:10–99:1 er), suggesting the key participation of noncovalent interactions involving halogens, most likely of halogen-π type. For comparative purposes, the synthesis of (*S*)-**3An** was also carried out employing NaBAr^F^_4_ as activator, reaching essentially the same yield, although in lower enantioselectivity (86:14 er), confirming that the better performance of SO_2_Sq **I** as activator of this transformation was not an isolated case for the model reaction. 7-Bromo/chloro-substituted indole-tethered ynediones **1F** and **1G** were subjected to reaction with *N*-methylindole (**2c**) to afford the opposite enantiomers (*S*)-**3Fc** and (*S*)-**3Gc** in excellent yields (87–91%), albeit with slightly lower enantioselectivities (88:12 and 87:13 er, respectively). Introduction of fluorinated aryl rings at the ynedione (Ar^2^ = 4-F-C_6_H_4_ and 3,5-(CF_3_)_2_-C_6_H_3_) while maintaining 7-chloro indole as a nucleophile was also investigated, yielding (*S*)-**3Hn** and (*S*)-**3In** in good to excellent enantioselectivities (87:13–96:4 er). Reaction of pyrrole derived substrate **1B** with chlorinated indole **2n** also afforded product (*S*)-**3Bn** in 85:15 er. Other π-excedent heterocycles such as benzofuran and benzothiophene were unreactive, while 2-methoxythiophene (**2r**) reacted smoothly to afford (*S*)-**3Ar** in 88% yield with 80:20 er. In a couple of representative examples, the reaction was scaled up to 1 mmol, affording (*S*)-**3An** and (*S*)-**3Ao** in good yields while maintaining the enantioselectivity. It is noteworthy that racemic mixtures of those products were fairly crystalline and precipitated under reaction media. This circumstance was exploited in the large-scale reaction to obtain essentially pure enantiomers of (*S*)-**3An** and (*S*)-**3Ao** after filtration of the reaction mixtures and subsequent chromatographic purification (Scheme 4). In this last operation, both gold-chloride catalyst **Au7** and sulfonyl squaramide **I** could be recovered (76–78% and 87–90%, respectively) and reused without any loss of efficiency.^16^ This recyclability is highly significant, as the gold chloride precatalyst is not recoverable from reactions using metal salts for activation. Product (*S*)-**3An** was crystallized and its structure elucidated by SC-XRD analysis (Scheme 4), unequivocally confirming the absolute *S* configuration of the newly created stereogenic center. Assuming a uniform reaction pathway, the absolute configuration of all of the other 3(2*H*)-furanones was assigned by analogy.

**Scheme 4:**
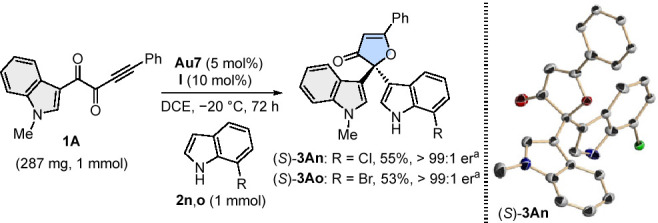
Scale-up and X-ray Structure of (*S*)-**3An** Yields and er of isolated products after filtration and column chromatography.

In summary, a catalytic system formed by the combination of DM-BINAP digold(I) dichloride complex **Au7** and sulfonyl squaramide **I** has allowed the development of asymmetric cycloisomerization/addition cascade reactions, yielding 3(2*H*)-furanones in moderate-to-excellent enantioselectivities. Interestingly, both the Au(I) complex and SO_2_Sq were recycled and reused in subsequent reactions.

## Data Availability

The data underlying this study are available in the published article and its Supporting Information.

## Supporting Information Available

Experimental details, optimizations, spectroscopic and analytical data for new compounds, and HPLC traces (PDF)
FAIR data, including the primary NMR FID files, for compounds **Au3**, **Au5**, **Au6**, **Au8**, **III**, **V**–**X**, **1E**–**G**, **1I**, **3Aa**–**3Ar** (ZIP)
