# Supplementary material for: Hydrogen-Bonding Activation of Gold(I) Chloride Complexes: Enantioselective Synthesis of 3(2H)-Furanones by a Cycloisomerization-Addition Cascade
Source: Org Lett. 2024 Jul 11;26(28):5995–6000. doi: 10.1021/acs.orglett.4c02091 (PMC11267603; doi:10.1021/acs.orglett.4c02091)
Supplement: Supplementary file 1 — ol4c02091_si_001.pdf [file ol4c02091_si_001.pdf]

Supporting information

Hydrogen-Bonding Activation of Gold(I) Chloride Complexes: Enantioselective Synthesis of 3(2*H*)-Furanones by a Cycloisomerization-Addition Cascade

Pilar Elías-Rodríguez,[†] Manuel Benítez,[†] Javier Iglesias-Sigüenza,[†] Elena Díez,[†,*] Rosario Fernández,[†,*] José M. Lassaletta[‡,*] and David Monge[†,*]

† Facultad de Química. Departamento de Química Orgánica. Universidad de Sevilla and Centro de Innovación en Química Avanzada (ORFEO-CINQA), C/Prof. García González, 1, 41012 Sevilla, Spain. E-mail: ediez@us.es, ffernan@us.es, dmonge@us.es

‡ Instituto de Investigaciones Químicas (CSIC-US) and Centro de Innovación en Química Avanzada (ORFEO-CINQA), Avda. Américo Vespucio, 49, 41092 Sevilla, Spain. E-mail: jmlassa@iiq.csic.es

Table of Contents

1. General information……………………………………………………………………………2

2. General procedure for the synthesis of ynediones substrates……………………………………..3

3. General procedure for the synthesis of chiral gold(I) chloride complexes………………………5

4. Synthesis of H-Bond Donor activators (III, V-X)…………………………………………………..8

5. Optimization of the catalytic system for cycloisomerization-addition cascade…………13

6. General procedure for the enantioselective cycloisomerization−indole addition cascade to aromatic ynediones………………………………………………………………………………..16

7. Large-scale reactions………………………………………………………………………....40

8. Catalyst recycling…………………………………………………………………………………42

9. Crystal Data for (*S*)**-3An**………………………………………………………………………43

10. References……………………………………………………………………………………45

11. NMR spectra of new compounds…………………………………………………………..…..47

1. General information

Unless otherwise stated, all reactions were performed in dry glassware under nitrogen or argon atmosphere using standard Schlenk techniques. THF and toluene were distilled over Na/benzophenone, CH_2Cl_2 was distilled from $CaCl_2$, and 1,2-DCE was taken from commercial bottle (Across; 99.5% extra dry) equipped with septa and molecular sieves. Other analytical grade solvents were used without further purification. All chemicals were purchased from Sigma-Aldrich, Across Organics, TCI, Fluorochem and BLD and used as received from commercial sources. Purifications by column chromatography were performed on silica gel (Merck Kieselgel 60). Analytical TLC was performed on aluminium backed plates (1.5 × 5 cm) pre-coated (0.25 mm) with silica gel (Merck, Silica Gel 60 F254). Semipreparative TLC was performed on glass backed plates (5 x 10 cm) pre-coated (0.25 mm) with silica gel (Merck, Silica Gel 60 F254). Compounds were visualized by exposure to UV light at 254 nm and/or by dipping the plates in solutions of $KMnO_4$, vanillin, *p*-anisaldehyde or phosphomolybdic acid stains followed by heating. ^{1}H, ^{13}C, ^{31}P and ^{19}F NMR spectra were collected on a Bruker Avance NEO 300 MHz and Bruker Avance NEO 500 MHz spectrometers at room temperature. ^{1}H NMR spectra were recorded at 300 MHz or 500 MHz (internal reference; $CDCl_3$ = 7.26 ppm; DMSO-d_6 = 2.50 ppm; Acetone-d_6 = 2.05 ppm; CD_2Cl_2 = 5.32 ppm). $^{13}C\{^{1}H\}$ NMR spectra were recorded at 75.5 or 126 MHz (internal reference; $CDCl_3$ = 77.0 ppm; DMSO-d_6 = 39.5 ppm; Acetone-d_6 = 29.84 ppm, CD_2Cl_2 = 53.84 ppm); $^{19}F\{^{1}H\}$ NMR spectra were recorded at 282 MHz or 471 MHz; $^{31}P\{^{1}H\}$ NMR spectra were recorded at 121.5 MHz or 202.5 MHz. Chemical shifts are expressed in parts per million (ppm, δ scale) and coupling constants are in hertz (Hz). Multiplicities were given as: s (singlet), br s (broad singlet), d (doublet), dd (double doublet), t (triplet), q (quartet), and m (multiplet). All the assignments were confirmed by COSY, HSQC and HMBC experiments. The melting point of crystalline solid (*S*)-**3An** was recorded in a metal block and is uncorrected. Optical rotations were measured on a JASCO *P-2000* polarimeter. High resolution mass spectra were recorded on a Q-Exactive spectrometer by a positive electrospray ionization (ESI) method using a Quadrupole-Orbitrap hybrid analyzer. The enantiomeric ratios of the products were determined by chiral stationary phase HPLC (TermoFisher Ultimate 3000, Daicel Chiralpak IA, IB and IC columns, Diode Array Detector DAD-3000(RS), column temperature 30 ºC, back pressure 500-650 psi) or SFC (ACQUITY UPC^2, Trefoil CEL1, CEL2 and AMY1 columns, UPC^2 Photo Diode Array Detector, column temperature 35 ºC, back pressure 4000-4800 psi). Not commercially available substrates **1A-D** and **1H**,[1] precursor **P1**,[2] activators **I**,[2] **II**,[3] **IV**,[2] indoles **2b**, **2e**, **2p** and **2q**,[4] amines **R1-R4**[5] and gold(I) chloride complexes **Au1**,[6] **Au2**,[7] **Au4**,[6] **Au7**,[8] **Au9**,[9] **Au10**,[9] **Au11**,[10] **Au12**,[11] **Au13**,[7] **Au14**,[12] **Au15**,[13] **Au16**,[8] **Au17**,[14] **Au18**[15] were synthetized according to literature procedures.

2. General procedure for the synthesis of novel ynedione substrates[1]

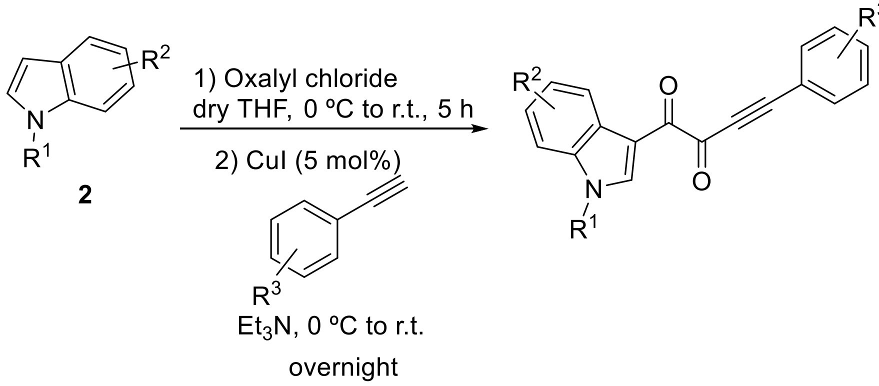

In a flame-dried Schlenk flask, the corresponding *N*-alkyl indole (3.0-6.0 mmol, 1.0 equiv) was dissolved in dry THF (15-30 mL, 0.2 M) and cooled down to 0 ºC. Then, oxalyl chloride (3.0-6.0 mmol, 1.0 equiv) was added dropwise at 0 ºC. The mixture was allowed to warm to room temperature and the reaction was stirred for 5 h. After this time, the reaction was cooled down again to 0 ºC and CuI (0.15-0.30 mmol, 5 mol%), the corresponding ethynylbenzene (3.0-6.0 mmol, 1.0 equiv) and Et_3N (9.0-18.0 mmol, 3.0 equiv) were successively added. The reaction was warmed to room temperature and stirred overnight. After consumption of starting material, water (30 mL) was added to the reaction mixture, the layers were separated, and the aqueous layer was extracted with CH_2Cl_2 (3 x 50 mL). The combined organic layers were dried over $MgSO_4$ and concentrated under reduced pressure. The resulting residue was purified by column chromatography on silica gel to afford the desired ynedione substrates.

Ynedione substrate 1E

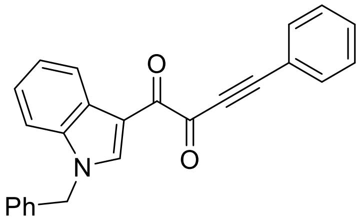

Reaction of 1-benzyl-1*H*-indole **2b** (710 mg, 3.4 mmol, 1.0 equiv) and oxalyl chloride (290 µL, 3.4 mmol, 1.0 equiv) in dry THF (17 mL, 0.2 M), followed by addition of CuI (33.0 mg, 0.17 mmol, 5 mol%), ethynylbenzene (381 µL, 3.4 mmol, 1.0 equiv) and Et_3N (1.5 mL, 10.2 mmol, 3.0 equiv) afforded **ynedione 1E** as a brown solid (363 mg, 0.99 mmol, 29%) after purification by column chromatography (EtOAc-hexane 8:1). **^{1}H NMR** (500 MHz, $CDCl_3$): δ 8.51 (d, *J* = 7.9 Hz, 1H), 8.34 (s, 1H), 7.71-7.70 (m, 2H), 7.51-7.47 (m, 1H), 7.42-7.39 (m, 2H), 7.38-7.29 (m 6H), 7.20-7.18 (m, 2H), 5.39 (s, 2H). **^{13}C NMR** (126 MHz, $CDCl_3$): δ 180.5, 178.7, 139.8, 137.0, 135.3, 133.8, 131.4, 129.3, 128.8, 128.5, 127.6, 127.2, 124.5, 123.8, 123.0, 119.9, 111.5, 110.8, 97.8, 87.9, 51.4. **HRMS** (ESI) *m/z* calcd. for $C_{25}H_{17}O_2NNa$ [M^++Na] 386.1152, found 386.1144.

Ynedione substrate 1F

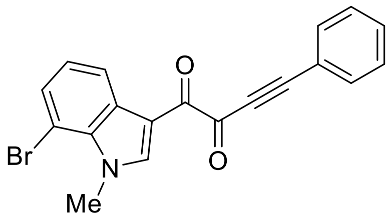

Reaction of 7-bromo-1-methyl-1*H*-indole **2p** (630 mg, 3.0 mmol, 1.0 equiv) and oxalyl chloride (263 µL, 3.0 mmol, 1.0 equiv) in dry THF (15 mL, 0.2 M), followed by addition of CuI (28.6 mg, 0.15 mmol, 5 mol%), ethynylbenzene (336 µL, 3.0 mmol, 1.0 equiv) and Et_3N (1.3 mL, 9.0 mmol, 3.0 equiv) afforded **ynedione 1F** as a yellow solid (357 mg, 0.98 mmol, 33%) after purification by column chromatography (EtOAc-hexane 10:1). **^{1}H NMR** (300 MHz, $CDCl_3$): δ 8.46 (d, *J* = 7.9 Hz, 1H), 8.30 (s, 1H), 7.70 (d, *J* = 7.2 Hz, 2H), 7.52-7.38 (m, 4H), 7.16 (t, *J* = 7.8 Hz, 1H), 4.22 (s, 3H). **^{13}C NMR** (75.5 MHz, $CDCl_3$): δ 180.0, 178.3, 142.8, 134.0, 133.8, 131.5, 130.5, 129.4, 128.8, 124.8, 122.2, 119.8, 110.4, 104.6, 98.1, 87.7, 38.5. **HRMS** (ESI) *m/z* calcd. for $C_{19}H_{12}O_2N^{79}BrNa$ [M^++Na] 387.9944, found 387.9938. *m/z* calcd. for $C_{19}H_{12}O_2N^{81}BrNa$ [M^++Na] 389.9923, found 389.9917.

Ynedione substrate 1G

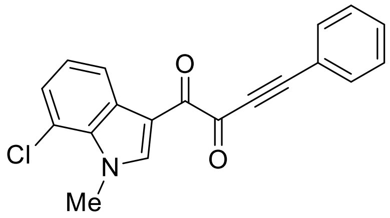

Reaction of 7-chloro-1-methyl-1*H*-indole **2q** (1.0 g, 6.0 mmol, 1.0 equiv) and oxalyl chloride (525 µL, 6.0 mmol, 1.0 equiv) in dry THF (30 mL, 0.2 M), followed by addition of CuI (57.1 mg, 0.3 mmol, 5 mol%), ethynylbenzene (672 µL, 6.0 mmol, 1.0 equiv) and Et_3N (2.5 mL, 18.0 mmol, 3.0 equiv) afforded **ynedione 1G** as a yellow solid (534 mg, 1.66 mmol, 28%) after purification by column chromatography (EtOAc-hexane 10:1). **^{1}H NMR** (300 MHz, $CDCl_3$): δ 8.37 (dd, *J* = 7.4, 1.6 Hz, 1H), 8.25 (s, 1H), 7.69-7.66 (m, 2H), 7.49-7.43 (m, 1H), 7.40-7.35 (m, 2H), 7.25-7.17 (m, 2H), 4.17 (s, 2H). **^{13}C NMR** (75.5 MHz, $CDCl_3$): δ 180.0, 178.3, 142.6, 133.8, 132.8, 131.5, 130.4, 128.8, 125.9, 124.5, 121.6, 119.8, 117.8, 110.6, 98.1, 87.7, 38.3. **HRMS** (ESI) *m/z* calcd. for $C_{19}H_{12}O_2NClNa$ [M^++Na] 344.0449, found 344.0446.

Ynedione substrate 1I

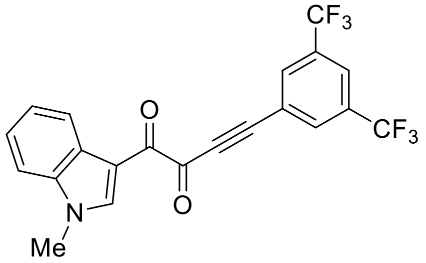

Reaction of *N*-methyl indole **2c** (390 µL, 3.0 mmol, 1.0 equiv) and oxalyl chloride (263 µL, 3.0 mmol, 1.0 equiv) in dry THF (15 mL, 0.2 M), followed by addition of CuI (28.6 mg, 0.15 mmol, 5 mol%), 1-ethynyl-3,5-bis(trifluoromethyl)benzene (540 µL, 3.0 mmol, 1.0 equiv) and Et_3N (1.3 mL, 9.0 mmol, 3.0 equiv) afforded **ynedione 1I** as an orange-to-brown solid (420 mg, 0.99 mmol, 33%) after purification by column chromatography (EtOAc-hexane 8:1). **^{1}H NMR** (300 MHz, $CDCl_3$): δ 8.48-8.43 (m, 1H), 8.39 (s, 1H), 8.14 (s, 2H), 7.97 (s, 1H), 7.41-7.37 (m, 3H), 3.91 (s, 3H). **^{13}C NMR** (75.5 MHz, $CDCl_3$): δ 179.1, 177.9, 140.5, 137.4, 133.3 (q, $J_{C,F}$ = 3.0 Hz), 132.6 (q, $J_{C,F}$ = 34.2 Hz), 127.3, 124.6, 124.5-124.4 (m), 124.0, 122.9, 122.7 (q, $J_{C,F}$ = 273.1 Hz), 122.5, 110.8, 110.2, 92.0, 89.2, 34.0. **^{19}F NMR** (282 MHz, $CDCl_3$): δ −63.2 (s, 6F). **HRMS** (ESI) *m/z* calcd. for $C_{21}H_{11}O_2NF_6Na$ [M^++Na] 446.0586, found 446.0579.

3. General procedure for the synthesis of chiral gold(I) chloride complexes

To a stirred solution of AuCl·SMe_2 (0.055-0.186 mmol, 2.0 equiv) in dry CH_2Cl_2 (0.5-1.2 mL) cooled at 0 ºC was added dropwise a solution of the corresponding chiral bisphosphine ligand (0.025-0.093 mmol, 1.0 equiv) in dry CH_2Cl_2 (0.5-1.2 mL). The cooling bath was removed, and the reaction mixture was stirred at room temperature for the specified time. Then, the solvent was concentrated, and the resulting residue was purified by flash chromatography to afford the desired bisphosphine gold(I) chloride complex.

(*R*)-Cy-BINAP·$(AuCl)_2$ (Au3)

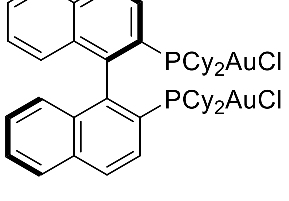

Reaction of Me_2SAuCl (57 mg, 0.186 mmol, 2.0 equiv) in dry CH_2Cl_2 (1.2 mL) and (*R*)-Cy-BINAP ligand (60 mg, 0.093 mmol, 1.0 equiv) in dry CH_2Cl_2 (1.2 mL) for 18 h and flash chromatography (EtOAc-Hex- CH_2Cl_2 4:4:1), afforded **(*R*)-Cy-BINAP·$(AuCl)_2$ (Au3)** as a white solid (99.3 mg, 0.089 mmol, 96%). **$[\alpha]_D^{25}$**= – 5.1 (*c* 1, $CHCl_3$). **^{1}H NMR** (300 MHz, $CDCl_3$): δ 8.28 (d, *J* = 9.0 Hz, 2H), 8.01 (d, *J* = 6.0 Hz, 2H), 7.76 (dd, *J* = 9.0, 7.0 Hz, 2H), 7.57-7.52 (m, 2H), 7.25-7.20 (m, 2H), 6.84 (d, *J* = 9.0 Hz, 2H), 2.74-2.64 (m, 2H), 2.37-2.33 (m, 2H), 2.20-2.14

(m, 4H), 1.95-1.84 (m, 4H), 1.77-1.52 (m, 16H), 1.47-1.31 (m, 6H), 1.23-1.10 (m, 10H). **^{13}C NMR** (75.5 MHz, $CDCl_3$): δ 144.5-144.3 (m), 134.7-134.6 (m), 130.0 (d, $J_{C,P}$ = 7.8 Hz), 129.0, 128.4, 127.8, 127.5 (d, $J_{C,P}$ = 3.4 Hz), 127.4, 126.9, 126.8, 37.4 (d, $J_{C,P}$ = 11.7 Hz), 36.9 (d, $J_{C,P}$ = 11.0 Hz), 33.7 (d, $J_{C,P}$ = 4.6 Hz), 31.5, 30.7, 29.5, 27.1 (d, $J_{C,P}$ = 8.5 Hz), 26.9 (d, $J_{C,P}$ = 7.2 Hz), 26.6, 26.5, 25.9, 25.6. **^{31}P NMR** (121.5 MHz, $CDCl_3$): δ 38.9 (s). **HRMS** (ESI) *m/z* calcd. for $C_{44}H_{56}Au_2Cl_2P_2Na$ [M^++Na] 1133.2458, found 1133.2450.

(*R*)-DTBM-BINAP·$(AuCl)_2$ (Au5)

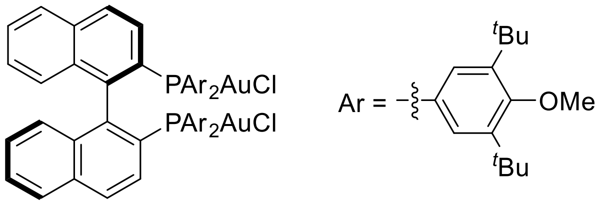

Reaction of Me_2SAuCl (25.5 mg, 0.084 mmol, 2.0 equiv) in dry CH_2Cl_2 (1 mL) and (*R*)-DTBM-BINAP ligand (50 mg, 0.042 mmol, 1.0 equiv) in dry CH_2Cl_2 (1 mL) for 20 h and flash chromatography (EtOAc-Hex 1:15), afforded **(*R*)-DTBM-BINAP·$(AuCl)_2$ (Au5)** as a white solid (69.5 mg, 0.042 mmol, quant.). **$[\alpha]_D^{25}$**= + 6.0 (*c* 1, $CHCl_3$). **^{1}H NMR** (500 MHz, $CDCl_3$): δ 8.24 (d, *J* = 9.1 Hz, 2H), 7.85 (d, *J* = 8.4 Hz, 2H), 7.64-7.62 (m, 2H), 7.45 (s, 2H), 7.42 (s, 2H), 7.26-7.23 (m, 2H), 7.12 (s, 2H), 7.09 (s, 2H), 6.43-6.40 (m, 2H), 6.33 (d, *J* = 8.6 Hz, 2H), 3.77 (s, 6H), 3.66 (s, 6H), 1.38 (s, 36H), 1.20 (s, 36H). **^{13}C NMR** (126 MHz, $CDCl_3$): δ 163.0, 162.3, 144.5 (d, $J_{C,P}$ = 11.7 Hz), 144.5 (d, $J_{C,P}$ = 12.4 Hz), 142.4-142.2 (m), 134.1-133.5 (m), 130.4 (d, $J_{C,P}$ = 5.3 Hz), 129.3 (d, $J_{C,P}$ = 8.8 Hz), 128.2, 127.7, 127.6, 127.4, 127.1, 125.1, 123.9, 123.3, 122.4, 121.8, 64.7, 64.6, 36.2, 36.0, 32.0, 31.9. **^{31}P NMR** (202.5 MHz, $CDCl_3$): δ 24.9 (s). **HRMS** (ESI) *m/z* calcd. for $C_{80}H_{104}O_4Au_2Cl_2P_2Na$ [M^++Na] 1677.6010, found 1677.5987.

(*R*)-DTB-BINAP·$(AuCl)_2$ (Au6)

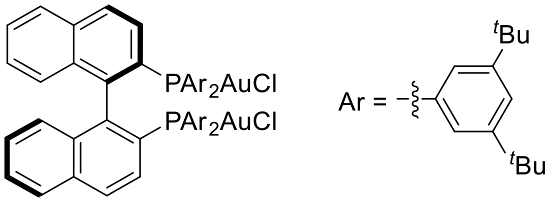

Reaction of Me_2SAuCl (18 mg, 0.055 mmol, 2.0 equiv) in dry CH_2Cl_2 (0.5 mL) and (*R*)-DTB-BINAP ligand[16] (29.3 mg, 0.025 mmol, 1.0 equiv) in dry CH_2Cl_2 (1.0 mL) for 24 h and flash chromatography (EtOAc-Hex 1:15), afforded **(*R*)-DTB-BINAP·$(AuCl)_2$ (Au6)** as a white solid (39.1 mg, 0.025 mmol, quant.). **$[\alpha]_D^{25}$**= + 18.7 (*c* 1, $CHCl_3$). **^{1}H NMR** (500 MHz, $CDCl_3$): δ 8.22 (d, *J* = 8.2 Hz, 2H), 7.87 (d, *J* = 8.2 Hz, 2H), 7.65-7.61 (m, 2H), 7.53-7.52 (m, 2H), 7.43-7.42 (m, 2H), 7.39 (d, *J* =1.6 Hz, 2H), 7.36 (d, *J* =1.6 Hz, 2H), 7.26-7.22 (m, 2H), 7.10 (d, *J* = 1.8 Hz, 2H), 7.07 (d, *J* = 1.8 Hz, 2H), 6.38-6.33 (m, 4H), 1.27 (s, 36H), 1.14 (s, 36H). **^{13}C NMR** (126 MHz,

$CDCl_3$): δ 151.5 (d, $J_{C,P}$ = 11.6 Hz), 151.4 (d, $J_{C,P}$ = 11.6 Hz), 134.2, 133.8, 133.7, 130.7-130.6 (m), 129.5-129.3 (m), 129.2, 129.0, 128.8, 128.3, 127.8, 127.4, 127.3, 126.8, 126.3, 125.3, 35.2, 35.0, 31.5, 31.4. **^{31}P NMR** (202.5 MHz, $CDCl_3$): δ 25.9 (s). **HRMS** (ESI) *m/z* calcd. for $C_{76}H_{96}Au_2Cl_2P_2Na$ [M^++Na] 1557.5588, found 1557.5574.

(*R*)-Xyl-Garphos·(AuCl)$_2$ (Au8)

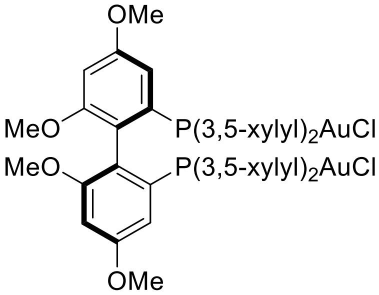

Reaction of Me_2SAuCl (33 mg, 0.106 mmol, 2.0 equiv) in dry CH_2Cl_2 (0.75 mL) and (*R*)-Xyl-Garphos ligand (40 mg, 0.053 mmol, 1.0 equiv) in dry CH_2Cl_2 (0.75 mL) for 24 h and flash chromatography (EtOAc-Hex 1:2), afforded **(*R*)-Xyl-Garphos·(AuCl)$_2$ (Au8)** as a white solid (54.7 mg, 0.045 mmol, 85%). **$[\alpha]_D^{25}$**= + 5.9 (*c* 1, CH_2Cl_2). **^{1}H NMR** (500 MHz, CD_2Cl_2): δ 7.13-7.10 (m, 6H), 7.05-7.01 (m, 6H), 6.54 (d, *J* = 2.2 Hz, 2H), 6.43 (dd, *J* = 11.9, 2.2 Hz, 2H), 3.73 (s, 6H), 3.16 (s, 6H), 2.30 (s, 12H), 2.27 (s, 12H). **^{13}C NMR** (126 MHz, CD_2Cl_2): δ 161.4 (d, $J_{C,P}$ = 13.8 Hz), 159.9 (d, $J_{C,P}$ = 15.8 Hz), 138.9-138.8 (m), 133.4 (d, $J_{C,P}$ = 1.8 Hz), 133.2 (d, $J_{C,P}$ = 2.3 Hz), 132.3, 132.2, 132.1, 130.8, 130.5, 130.3, 130.0, 129.4, 129.0, 123.1-122.9 (m), 113.7 (d, $J_{C,P}$ = 6.7 Hz), 101.9, 56.1, 55.1, 21.6, 21.5. **^{31}P NMR** (202.5 MHz, CD_2Cl_2): δ 23.8 (s). **HRMS** (ESI) *m/z* calcd. for $C_{48}H_{52}O_4Au_2Cl_2P_2Na$ [M^++Na] 1241.1941, found 1241.1914.

4. Synthesis of Hydrogen-Bond Donor activators (III, V-X)

Synthesis of activator III

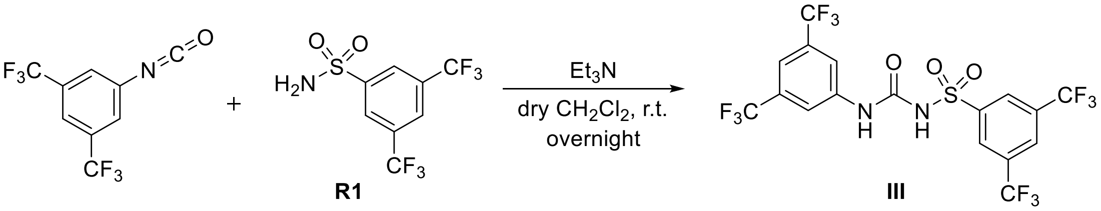

In a flame-dried Schlenk flask, **R1** (146 mg, 0.5 mmol, 1.0 equiv) was suspended in dry CH_2Cl_2 (2 mL, 0.25 M) and dry Et_3N (139 µL, 1.0 mmol, 2.0 equiv) was added at room temperature. After stirring for 5 min, 3,5-bis(trifluoromethyl)phenyl isocyanate (88 µL, 0.5 mmol, 1.0 equiv) was added dropwise. The resulting solution was stirred overnight at room temperature. Then, the mixture was diluted with CH_2Cl_2 (15 mL) and washed with H_2O (3 x 20 mL), dried over $MgSO_4$, filtered, and concentrated under reduced pressure. The resulting residue was purified by column chromatography on silica gel (Hex-EtOAc 1:1 → 1:4) to afford organocatalyst **III** as an off-white solid (172 mg, 0.31 mmol, 63%). **^{1}H NMR** (300 MHz, DMSO-d_6): *NH-SO$_2$Ar^F signal is not observed* δ 9.37 (s, 1H), 8.33 (s, 2H), 8.22 (s, 1H), 8.08 (s, 2H), 7.38 (s, 1H). **^{13}C NMR** (126 MHz, DMSO-d_6): δ 158.3, 149.3, 144.0, 130.3 (q, $J_{C,F}$ = 32.2 Hz), 130.0 (q, $J_{C,F}$ = 33.3 Hz), 127.3 (q, $J_{C,F}$ = 3.4 Hz), 123.7-123.6 (m), 123.5 (q, $J_{C,F}$ = 272.3 Hz), 123.1 (q, $J_{C,F}$ = 273.1 Hz), 116.6 (q, $J_{C,F}$ = 2.6 Hz), 112.1-112.0 (m). **^{19}F NMR** (282 MHz, DMSO-d_6): δ −61.4 (s, 6F), −61.7 (s, 6F). **HRMS** (ESI) *m/z* calcd. for $C_{17}H_7O_3N_2F_{12}S$ [M^+−H] 546.9991, found 546.9971

Synthesis of activator V

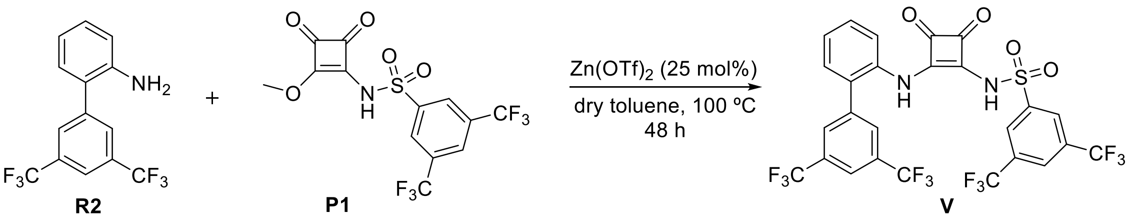

In a flame-dried Schlenk flask, **R2** (275 mg, 0.9 mmol, 1.8 equiv) was added to a stirred solution of **P1** (202 mg, 0.5 mol, 1.0 equiv) and zinc(II) trifluoromethanesulfonate (46.4 mg, 0.125 mmol, 25 mol%) in dry toluene (2 mL, 0.25 M) at room temperature. The resulting mixture was stirred at 100 ºC in an oil bath for 48 h. Then, the solvent was evaporated under reduced pressure and the resulting residue was purified by column chromatography on silica gel (Hex-EtOAc 1:4 → 1:8) to afford organocatalyst **V** as a white solid (163 mg, 0.24 mmol, 48%). **^{1}H NMR** (300 MHz, DMSO-d_6): *NH-SO$_2$Ar^F signal is not observed* δ 9.32 (s, 1H), 8.29 (s, 2H), 8.25 (s, 1H), 8.00 (s, 1H), 7.95 (s, 2H), 7.54 (d, *J* = 7.8 Hz, 1H), 7.44-7.38 (m, 2H), 7.24 (td, *J* = 7.5, 1.0 Hz, 1H). **^{13}C**

NMR (126 MHz, DMSO-d_6): δ 189.1, 185.7, 175.3, 171.4, 147.9, 141.0, 135.8, 130.6 (q, $J_{C,F}$ = 33.5 Hz), 130.6, 130.4, 130.3 (q, $J_{C,F}$ = 33.0 Hz), 130.0 (br s), 129.3, 126.9 (q, $J_{C,F}$ = 2.1 Hz), 124.6, 124.4-124.3 (m), 123.7, 123.2 (q, $J_{C,F}$ = 272.9 Hz), 122.8 (q, $J_{C,F}$ = 273.2 Hz), 120.8-120.7 (m). **^{19}F NMR** (471 MHz, DMSO-d_6): δ −61.6 (s, 6F), −61.7 (s, 6F). **HRMS** (ESI) *m/z* calcd. for $C_{26}H_{12}O_4N_2F_{12}NaS$ [M$^+$+Na] 699.0218, found 699.0215.

Synthesis of activator VI

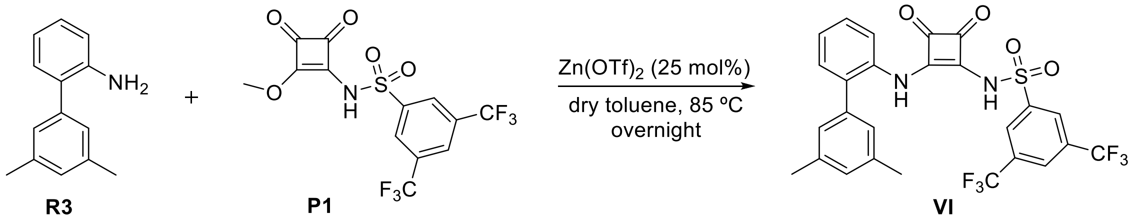

In a flame-dried Schlenk flask, **R3** (178 mg, 0.9 mmol, 1.8 equiv) was added to a stirred solution of **P1** (202 mg, 0.5 mol, 1.0 equiv) and zinc(II) trifluoromethanesulfonate (46.4 mg, 0.125 mmol, 25 mol%) in dry toluene (2 mL, 0.25 M) at room temperature. The resulting mixture was stirred at 85 ºC in an oil bath overnight. Then, the solvent was evaporated under reduced pressure and the resulting residue was purified by column chromatography on silica gel (Hex-EtOAc 1:10) to afford organocatalyst **VI** as a pale-yellow solid (175 mg, 0.31 mmol, 62%). **^{1}H NMR** (500 MHz, DMSO-d_6): *NH-SO$_2$Ar^F signal is not observed* δ 8.84 (s, 1H), 8.28 (s, 1H), 8.22 (s, 2H), 7.61 (dd, *J* = 8.1, 0.7 Hz, 1H), 7.31 (td, *J* = 7.8, 1.7 Hz, 1H), 7.20 (dd, *J* = 7.6, 1.6 Hz, 1H), 7.11 (td, *J* = 7.5, 1.1 Hz, 1H), 6.97 (s, 3H), 2.24 (s, 6H). **^{13}C NMR** (126 MHz, DMSO-d_6): δ 189.7, 185.6, 174.8, 170.5, 147.7, 137.7, 132.1, 130.8 (q, $J_{C,F}$ = 33.6 Hz), 130.0, 128.9, 126.8, 126.6-126.5 (m), 126.1 (q, $J_{C,F}$ = 273.4 Hz), 124.7-124.5 (m), 123.4, 121.2, 20.8. **^{19}F NMR** (282 MHz, DMSO-d_6): δ −61.5 (s, 6F). **HRMS** (ESI) *m/z* calcd. for $C_{26}H_{18}O_4N_2F_6NaS$ [M$^+$+Na] 591.0784, found 591.0782.

Synthesis of I-1 and activator VII

·Synthesis of I-1

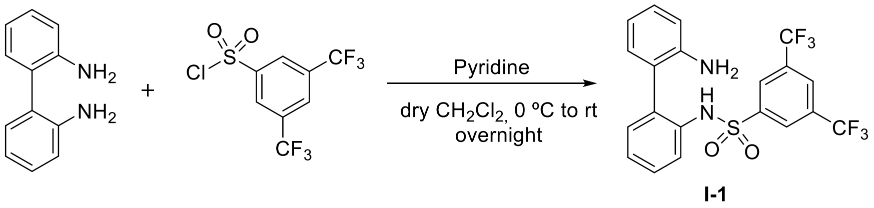

In a round-bottom flask, [1,1'-biphenyl]-2,2'-diamine (368.5 mg, 2.0 mmol, 1.0 equiv) and dry pyridine (1.9 mL, 24.0 mmol, 12.0 equiv) were dissolved in dry CH_2Cl_2 (20 mL, 0.1 M) and cooled to 0 ºC. Then, a solution of 3,5-bis(trifluoromethyl)benzenesulfonyl chloride (638 mg, 2.0

mmol, 1.0 equiv) in dry CH_2Cl_2 (10 mL, 0.2 M) was added over 4 h while the reaction was maintained at 0 ºC. After addition, the dark-red solution was allowed to warm to room temperature and stirred overnight. The resulting mixture was washed sequentially with 5% aqueous HCl (2 x 30 mL), H_2O (50 mL) and brine (50 mL), dried over $MgSO_4$, filtered, and concentrated under reduced pressure. The residue was passed through a pad of silica gel eluting with CH_2Cl_2 and concentrated again. The obtained solid was dissolved in a mixture of Hexane-Et_2O 1:1, heated until most of the solvent was evaporated and then stored at –20 ºC for 24 h. After this time, the yellow precipitate was filtered off and washed with cold Hexane (10 mL) to afford the pure amine **I-1** (408 mg, 0.9 mmol, 44%). **^{1}H NMR** (300 MHz, DMSO-d_6): δ 8.55 (s, 1H), 7.84 (s, 1H), 7.75 (dd, *J* = 8.0, 1.1 Hz, 1H), 7.58 (s, 2H), 7.50 (td, *J* = 7.7, 1.6 Hz, 1H), 7.38 (td, *J* = 7.5, 1.3 Hz, 1H), 7.19 (dd, *J* = 7.6, 1.5 Hz, 1H), 7.07 (td, *J* = 7.9, 1.4 Hz, 1H), 6.74 (dd, *J* = 8.0, 0.8 Hz, 1H), 6.49 (td, *J* = 7.5, 0.9 Hz, 1H), 6.20 (dd, *J* = 7.6, 1.4 Hz, 1H), 3.60 (s, 2H). **^{13}C NMR** (75.5 MHz, DMSO-d_6): δ 141.5, 140.9, 132.8, 132.5, (q, $J_{C,F}$ = 34.2 Hz), 130.6, 130.0 (q, $J_{C,F}$ = 268.2 Hz), 129.5, 129.4, 128.5, 126.7 (q, $J_{C,F}$ = 3.2 Hz), 126.0-125.8 (m), 125.6, 120.8, 120.6, 117.1. **^{19}F NMR** (282 MHz, DMSO-d_6): δ −62.9 (s, 6F). **HRMS** (ESI) *m/z* calcd. for $C_{20}H_{15}O_2N_2F_6S$ [M^++H] 461.0753, found 461.0750.

·Synthesis of activator VII

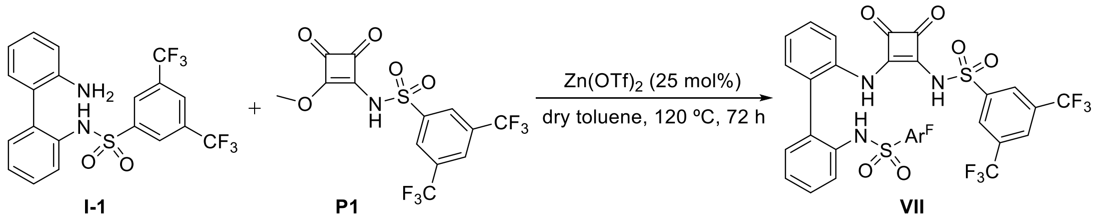

In a flame-dried Schlenk flask, **I-1** (345 mg, 0.7 mmol, 1.5 equiv) was added to a stirred solution of **P1** (202 mg, 0.5 mol, 1.0 equiv) and zinc(II) trifluoromethanesulfonate (46.4 mg, 0.125 mmol, 25 mol%) in dry toluene (2 mL, 0.25 M) at room temperature. The resulting mixture was stirred at 120 ºC in an oil bath for 72 h. After this time, the solvent was evaporated under reduced pressure and the resulting residue was purified by column chromatography on silica gel (EtOAc-Acetone 15:1) to afford organocatalyst **VII** as a pale beige solid (125.4 mg, 0.15 mmol, 30%). **^{1}H NMR** (300 MHz, DMSO-d_6): *one of NH-SO_2Ar^F signal is not observed* δ 9.87 (s, 1H), 8.75 (s, 1H), 8.37 (s, 1H), 8.22 (s, 1H), 8.15 (s, 2H), 8.07 (s, 2H), 7.60 (d, *J* = 8.1 Hz, 1H), 7.33-7.18 (m, 4H), 6.98-6.89 (m, 2H), 6.82 (d, *J* = 7.4 Hz, 1H). **^{13}C NMR** (126 MHz, DMSO-d_6): δ 190.1, 185.4, 173.7, 169.9, 147.5, 142.7, 136.5, 136.4, 133.7, 131.9, 131.1 (q, $J_{C,F}$ = 33.7 Hz), 130.7 (q, $J_{C,F}$ = 33.4 Hz), 130.4, 129.2, 128.6, 127.9, 127.8, 127.7 (br s), 127.1 (br s), 126.4 (br s), 124.5-124.4 (m), 122.7 (q, $J_{C,F}$ = 273.2 Hz), 122.4, 122.4 (q, $J_{C,F}$ = 273.3 Hz), 121.1. **^{19}F NMR** (471 MHz, DMSO-d_6): δ −61.5 (s, 6F), −61.6 (s, 6F). **HRMS** (ESI) *m/z* calcd. for $C_{32}H_{17}O_6N_3F_{12}NaS_2$ [M^++Na] 854.0259, found 854.0250.

Synthesis of activator VIII

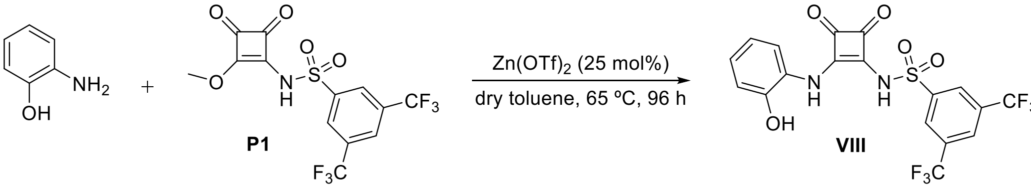

In a flame-dried Schlenk flask, 2-aminophenol (99.2 mg, 0.9 mmol, 1.8 equiv) was added to a stirred solution of **P1** (202 mg, 0.5 mol, 1.0 equiv) and zinc(II) trifluoromethanesulfonate (46.4 mg, 0.125 mmol, 25 mol%) in dry toluene (4 mL, 0.125 M) at room temperature. The resulting mixture was stirred at 65 ºC in an oil bath for 48 h. Then, an additional amount of 2-aminophenol (55 mg, 0.5 mmol, 1.0 equiv) was added and the reaction was allowed to stir for other 48 h at 65 ºC. After this time, the solvent was evaporated under reduced pressure and the resulting residue was purified by column chromatography on silica gel (EtOAc → EtOAc-MeOH 100:1) to afford organocatalyst **VIII** as a dark-orange solid (103.2 mg, 0.21 mmol, 43%). **^{1}H NMR** (500 MHz, DMSO-d_6): *NH-SO$_2$Ar^F signal is not observed* δ 10.06 (s, 1H), 9.00 (s, 1H), 8.40 (s, 2H), 8.29 (s, 1H), 7.84 (dd, *J* = 7.9, 0.9 Hz, 1H), 6.89-6.81 (m, 2H), 6.77-6.73 (m, 1H). **^{13}C NMR** (126 MHz, DMSO-d_6): δ 189.3, 185.7, 174.4, 170.0, 147.8, 146.3, 130.9 (q, $J_{C,F}$ = 33.7 Hz), 127.2, 126.9-126.8 (m), 124.8-124.6 (m), 123.0, 122.8 (q, $J_{C,F}$ = 273.4 Hz), 119.3, 119.1, 114.8. **^{19}F NMR** (471 MHz, DMSO-d_6): δ −61.5 (s, 6F). **HRMS** (ESI) *m/z* calcd. for $C_{18}H_{10}O_5N_2F_6NaS$ [M$^+$+Na] 503.0107, found 503.0105.

Synthesis of activator IX

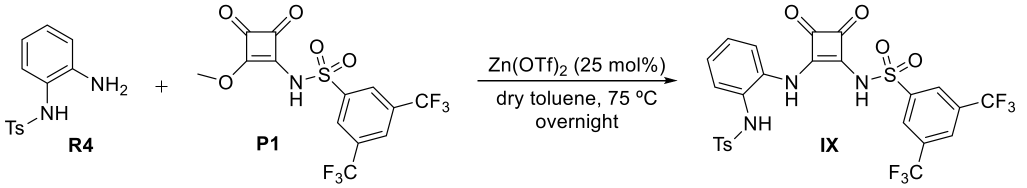

In a flame-dried Schlenk flask, **R4** (262 mg, 1.0 mmol, 2.0 equiv) was added to a stirred solution of **P1** (202 mg, 0.5 mol, 1.0 equiv) and zinc(II) trifluoromethanesulfonate (46.4 mg, 0.125 mmol, 25 mol%) in dry toluene (2 mL, 0.25 M) at room temperature. The resulting mixture was stirred at 75 ºC in an oil bath overnight. Then, the solvent was evaporated under reduced pressure and the resulting residue was purified by column chromatography on silica gel (Hex-EtOAc 1:4 → 1:8). The obtained solid was washed with cold Et_2O (20 mL) to afford organocatalyst **IX** as an off-white solid (253.7 mg, 0.40 mmol, 80%). **^{1}H NMR** (500 MHz, DMSO-d_6): *NH-SO$_2$Ar^F signal is not observed* δ 9.67 (s, 1H), 8.84 (s, 1H), 8.60 (s, 2H), 8.32 (s, 1H), 7.32-7.29 (m, 3H), 7.16 (t, *J* = 7.3 Hz, 1H), 7.04-7.02 (m, 1H), 6.99.-6.96 (m, 1H), 6.83 (d, *J* = 8.1 Hz, 2H), 2.00 (s, 3H). **^{13}C NMR** (126 MHz, DMSO-d_6): δ 188.6, 184.4, 175.7, 170.5, 148.4, 143.0, 136.3, 134.5, 130.7 (q, $J_{C,F}$ = 33.5 Hz), 128.9, 128.5, 127.8 (q, $J_{C,F}$ = 3.1 Hz), 127.6, 126.2, 125.3, 124.5-124.4 (m), 123.4,

122.9 (q, $J_{C,F}$ = 273.4 Hz), 122.0 (br s), 20.6. **^{19}F NMR** (282 MHz, DMSO-d_6): δ −61.4 (s, 6F). **HRMS** (ESI) *m/z* calcd. for $C_{25}H_{17}O_6N_3F_6NaS_2$ [M^++Na] 656.0355, found 656.0350.

Synthesis of activator X

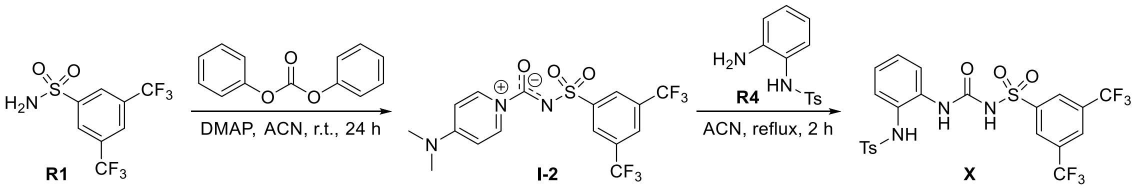

Activator **X** was synthesized following a reported procedure.[17] In a round-bottom flask, **R1** (586 mg, 2.0 mmol, 1.0 equiv) and DMAP (488 mg, 4.0 mmol, 2.0 equiv) were dissolved in ACN (4 mL, 0.5 M) at room temperature and stirred for few minutes. Then, diphenyl carbonate (476 mg, 2.2 mmol, 1.1 equiv) was added in one portion. The resulting mixture was stirred at room temperature for 24 h. Then, the formed precipitate was filtered off under vacuum and washed with water and cold MeOH thoroughly to obtain carbamoylide **I-2** as a white solid (425 mg) which was used in the next step without further purification.

Carbamoylide **I-2** (221 mg, 0.5 mmol, 1.0 equiv) and **R4** (144 mg, 0.55 mmol, 1.1 equiv) were suspended in ACN (5 mL, 0.1 M) and the resulting mixture was stirred at reflux (in an oil bath) for 2 h. Then, the solvent was evaporated under reduced pressure and the obtained white solid was suspended in H_2O and treated with 1% aqueous HCl until pH~2. The aqueous phase was removed by filtration, and the solid compound was purified by column chromatography on silica gel (Hex-EtOAc 1:1 → 1:4) to afford the pure organocatalyst **X** as a white solid (108 mg, 0.19 mmol, 37%). **^{1}H NMR** (500 MHz, DMSO-d_6): *NH-SO$_2$Ar^F signal is not observed* δ 10.09 (s, 1H), 8.39 (s, 2H), 8.24 (s, 1H), 8.06 (s, 1H), 7.40-7.38 (m, 3H), 7.20 (d, J = 8.1 Hz, 2H), 7.01-6.97 (m, 1H), 6.89-6.78 (m, 2H), 2.31 (s, 3H). **^{13}C NMR** (126 MHz, DMSO-d_6): δ 142.8, 137.1, 135.5, 129.9 (q, $J_{C,F}$ = 33.3 Hz), 129.3, 127.5-127.4 (m), 126.8, 126.3, 126.4, 125.8, 123.7-123.5 (m), 123.0 (q, $J_{C,F}$ = 273.1 Hz), 122.0, 121.6, 20.9. **^{19}F NMR** (471 MHz, DMSO-d_6): δ −61.3 (s, 6F). **HRMS** (ESI) *m/z* calcd. for $C_{22}H_{17}O_5N_3F_6NaS_2$ [M^++Na] 604.0406, found 604.0406.

5. Optimization of the catalytic system for cycloisomerization-addition cascade

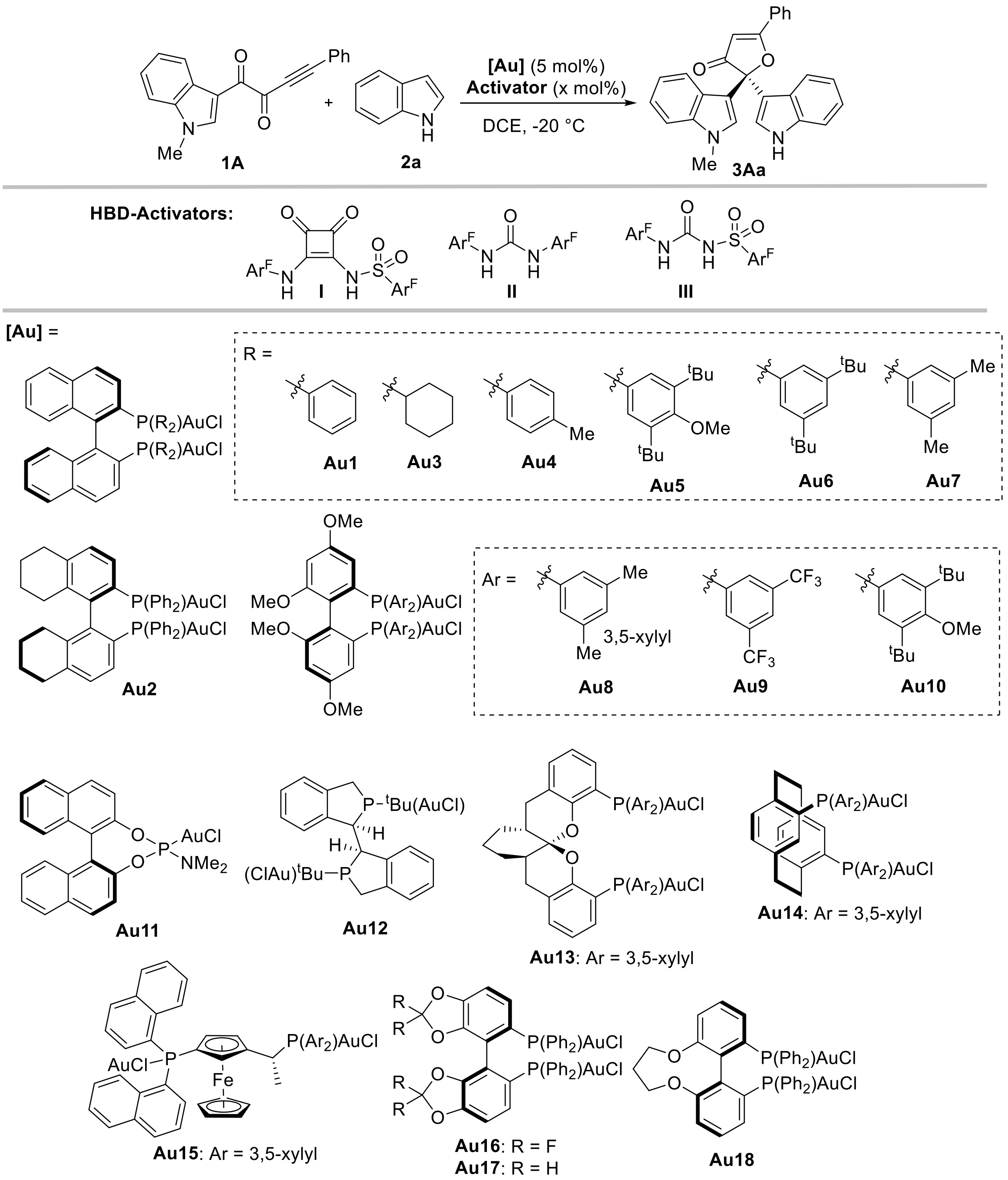

In a flame-dried Schlenk, a solution of aromatic ynedione **1A** (57.5 mg, 0.2 mmol, 1.0 equiv) and the corresponding indole **2a** (23.7 mg, 0.2 mmol, 1.0 equiv) in anhydrous 1,2-DCE (0.6 mL, 0.33 M) was cooled at −20 ºC for 10 mins. Then, the **[Au]** complex (0.01 mmol, 5 mol%) and the corresponding activator (0.01-0.02 mmol, 5-10 mol%) were added. The reaction mixture was stirred at −20 ºC for 16 h. Then, the reaction was quenched with Et_3N (1.4 µL, 0.01 mmol) and the solvent was eliminated under reduced pressure. NMR yields were determined by ^1H NMR (Acetone-d_6) using 1,3,5- trimethoxybenzene as internal standard (see **Table 1**). The product **3Aa** was isolated by semipreparative TLC (EtOAc-hexane 1:2).

Spectroscopic data of product **3Aa** are in accordance with those reported in literature.[18] The following diagnostic signals were integrated to quantify the amount of starting material and products: for substrate **1A**, peak at 3.85 ppm (s, 3H); for product **3Aa**, peaks at 10.11 ppm (s, 1H) and 6.45 ppm (s, 1H).

Enantiomeric ratios were determined by chiral HPLC analysis using Chiralpak-IA column (hexane/propan-2-ol 80:20, flow rate 1 mL/min).

Table 1 Optimization of the catalytic system for model cycloisomerization-addition cascade between **1A** and **2a**.[a]

Entry	[Au]	Activator (x mol%)	Yield [%][b]	*er*[c]
1	**Au1**	**I** (5)	72	65:35
2	**Au1**	**I** (10)	>95	70:30
3	**Au1**	**II** (10)	44	65:35
4	**Au1**	**III** (10)	73	70:30
5	**Au2**	**I** (10)	83	67:33
6	**Au3**	**I** (10)	16	65:35
7	**Au4**	**I** (10)	>95	72:28
8	**Au4**	$NaBAr^F_4$ (5)	>95	71:29
9	**Au5**	**I** (10)	30	62:38
10	**Au6**	**I** (10)	13	63:37
11	**Au7**	**I** (10)	>95	86:14
12	**Au7**	$NaBAr^F_4$ (10)	>95	81:19
13	**Au7**	$NaBAr^F_4$ (5)	>95	84:16
14	**Au8**	**I** (10)	49	85:15
15	**Au8**	$NaBAr^F_4$ (5)	94	82:18
16	**Au9**	**I** (10)	<5	nd
17	**Au9**	$NaBAr^F_4$ (5)	>95	80:20
18	**Au10**	**I** (10)	26	58:42
19	**Au10**	$NaBAr^F_4$ (5)	>95	61:39
20	**Au11**	**I** (10)	49	*rac.*
21	**Au11**	$NaBAr^F_4$ (5)	>95	*rac.*
22	**Au12**	**I** (10)	>95	84:16
23	**Au12**	$NaBAr^F_4$ (5)	>95	76:24
24	**Au13**	**I** (10)	>95	70:30
25	**Au13**	$NaBAr^F_4$ (5)	>95	*rac.*
26	**Au14**	**I** (10)	72	70:30
27	**Au15**	**I** (10)	12	69:31
28	**Au16**	**I** (10)	26	62:38
29	**Au16**	$NaBAr^F_4$ (5)	>95	66:34
30	**Au17**	**I** (10)	22	57:43
31	**Au17**	$NaBAr^F_4$ (5)	94	63:37
32	**Au18**	**I** (10)	75	71:29

[a] Reactions were performed at 0.2 mmol scale employing [Au] (5 mol%) and activator (10 mol%) for 16 h at -20 °C. **Au** complexes were purified by flash chromatography prior to use. [b] Determined by ^{1}H-NMR using 1,3,5-trimethoxybenzene as internal standard. An error margin of ≈5% is assumed. [c] Determined by chiral HPLC analysis.

From the initial screening of different hydrogen bond donors (HBD) as activators, sulfonyl squaramide (SO_2Sq) **I** was identified as the most promising activator, affording the desired product **3Aa** in quantitative yield and a promising 70:30 er, using 10 mol% of **I** (entries 1-4). To further improve the enantioselectivity, different gold complexes **Au1-Au18** were evaluated. First, we studied the influence of diphosphine groups (PR_2) in BINAP based-complexes **Au3-Au7**. A marked increase of enantioinduction was observed for derivate **Au7**, furnishing **3Aa** in quantitative yield and 86:14 er (entries 6-11). The use of partially hydrogenated BINAP-derived complex **Au2** was unsuccessfully tested, leading to a lower enantioselectivity of 67:33 er (entry 5). Other axially chiral complexes **Au8-Au10**, bearing biphenyl scaffolds, were checked. Complex **Au8**, containing bis-(*m*-xylyl)phosphine groups, afforded **3Aa** in 49% yield and the higher enantioselectivity of this series (85:15 er, entry 14). Other structural modifications of complex **Au8** were unsuccessfully tested. Complexes **Au9** and **Au10**, bearing 3,3'-bis-trifluoromethylphenyl group and 4-methoxy-3,5-di-tert-butylphenyl group, respectively, exhibited an inhibition or lower catalytic activities in this order (entries 16 and 18). Phosphoramidite-based complex **Au11** was less active, affording **3Aa** in 49% yield as racemic (entry 20). The catalytic performance of dinuclear complexes bearing central chirality, such as **Au12** (entry 22) and **Au13** (entry 24), was also competitive, the former yielding **3Aa** in 84:16 er. Complex **Au14** bearing a cyclophane-type ligand was competent (entry 26), while ferrocene-based complex **Au15** afforded a low yield of 12% (entry 27), probably due to the steric hindrance exerted by naphthyl rings in one of the phosphines, thereby preventing the activation. **Au16** and **Au17** also showed low catalytic activity (entries 28 and 30), questioning whether the presence of accessible oxygens (H-bond acceptors) in the ligand backbone might be involved in H-bonding with SO_2Sq **I**, thus hampering the activation process, although this trend is not so pronounced for other oxygen-containing complexes such as **Au8**, **Au13** or **Au18**. In fact, **Au18** afforded **3Aa** in 75% yield and 71:29 er (entry 32). Overall, the results summarized in Table 1 indicate that using **Au7**, based on DM-BINAP, led to the best reactivity/enantioselectivity.

To confirm that the low yields collected in some specific cases are mainly due to an inefficient activation of gold chloride complexes by SO_2Sq **I**, the use of **Au7** and others in combination with $NaBAr^F_4$ allowed to obtain quantitative yields in most of the cases, albeit without significant improvements of the enantioselectivities (see the comparative study in Table 1). This result highlights the suitability of the activation of gold chloride complexes by SO_2Sq **I** for developing of enantioselective transformations.

6. General procedure for the enantioselective cycloisomerization−indole addition cascade to aromatic ynediones

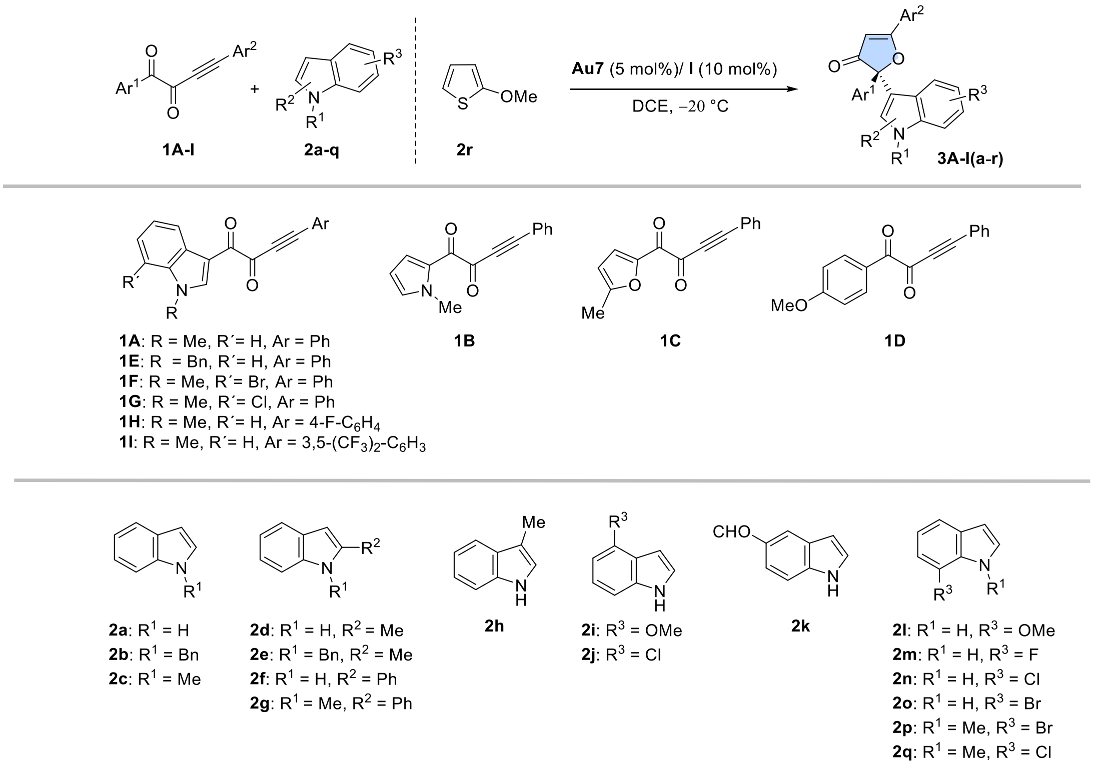

General procedure: In a flame-dried Schlenk flask, a solution of aromatic ynedione **1** (0.2 mmol, 1.0 equiv) and the corresponding indole **2** (or other hetero/carbocycles) (0.2 mmol, 1.0 equiv) in anhydrous 1,2-DCE (0.6 mL, 0.33 M) was cooled at –20 ºC for 10 mins. Then, **Au7** (12 mg, 0.01 mmol, 5 mol%) and **I** (12 mg, 0.02 mmol, 10 mol%) were added. The reaction mixture was stirred at –20 ºC for the specified time. Then, the reaction was purified directly by column chromatography on silica gel to afford pure products **3**. Enantiomeric ratios were determined by HPLC or SFC analysis using chiral columns.

Racemic products were synthesized in an analogous procedure using PPh_3AuCl (5.2 mg, 0.01 mmol, 5 mol%) and **I** (6.0 mg, 0.01 mmol, 5 mol%) as the catalytic system.

(*S*)-2-(1*H*-indol-3-yl)-2-(1-methyl-1*H*-indol-3-yl)-5-phenylfuran-3(2*H*)-one [(*S*)-3Aa]

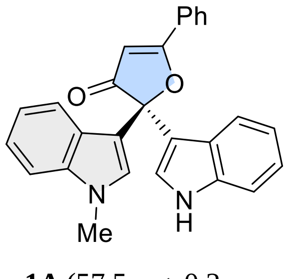

Following the general procedure from **1A** (57.5 mg, 0.2 mmol) and indole **2a** (23.7 mg, 0.2 mmol) for 16 h, compound (*S*)-**3Aa** was obtained as a pale brown solid after purification by column chromatography on silica gel (Hexane → EtOAc-hexane 1:3 → 1:1) (77.7 mg, 0.19 mmol, 95%,

86:14 er). **[α]$_D$^{23}**= + 19.1 (*c* 1, DMSO). Spectroscopic and physical data were consistent with literature values.[18] **^{1}H NMR** (500 MHz, DMSO-*d*$_6$): δ 11.22 (s, 1H), 8.06-8.04 (m, 2H), 7.65-7.62 (m, 1H), 7.59-7.56 (m, 2H), 7.48 (d, *J* = 8.0 Hz, 2H), 7.44 (t, *J* = 8.3 Hz, 2H), 7.23 (s, 1H), 7.20 (d, *J* = 2.4 Hz, 1H), 7.15 (t, *J* = 7.6 Hz, 1H), 7.08 (t, *J* = 7.5 Hz, 1H), 6.97-6.91 (m, 2H), 6.62 (s, 1H), 3.75 (s, 3H). **^{13}C NMR** (126 MHz, DMSO-*d*$_6$): δ 202.0, 182.6, 137.2, 136.7, 133.0, 129.2, 128.9, 128.7, 127.1, 125.7, 125.4, 124.9, 121.5, 121.4, 120.2, 119.9, 119.2, 119.1, 111.9, 111.8, 111.1, 110.1, 99.0, 89.5, 32.4.

Enantioselectivity was determined by chiral HPLC analysis using Chiralpak-IA column (hexane/propan-2-ol 80:20, flow rate 1 mL/min): τ_{major} = 19.1 min, τ_{minor} = 21.7 min.

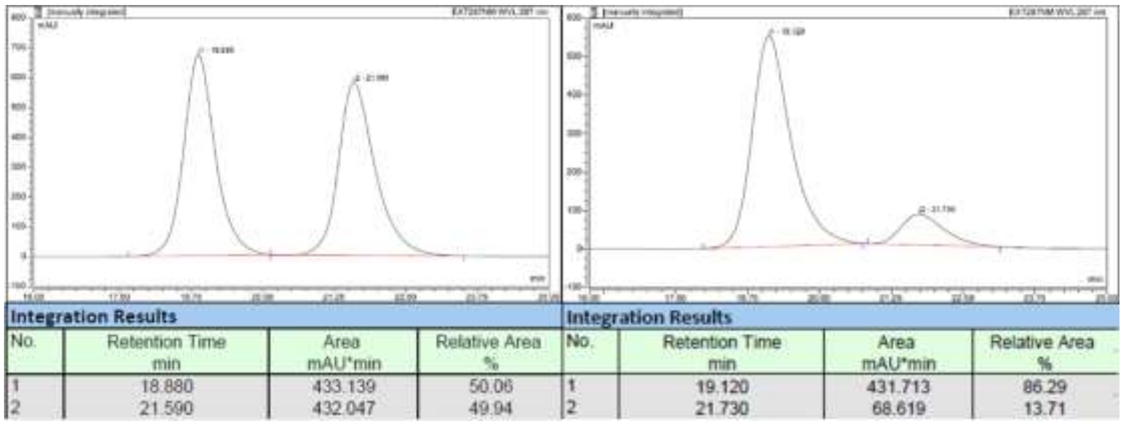

Integration Results

No.	Retention Time min	Area mAU*min	Relative Area %
1	18.880	433.139	50.06
2	21.590	432.047	49.94

Integration Results

No.	Retention Time min	Area mAU*min	Relative Area %
1	19.120	431.713	86.29
2	21.730	68.619	13.71

(*S*)-2-(1*H*-indol-3-yl)-2-(1-methyl-1*H*-pyrrol-2-yl)-5-phenylfuran-3(2*H*)-one [(*S*)-3Ba]

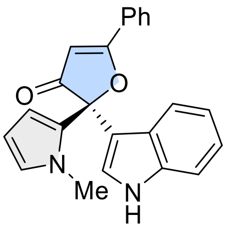

Following the general procedure from **1B** (47.5 mg, 0.2 mmol) and indole **2a** (23.7 mg, 0.2 mmol) for 24 h, compound (*S*)-**3Ba** was obtained as a pale-yellow solid after purification by column chromatography on silica gel (Hexane → EtOAc-hexane 1:2) (58.2 mg, 0.16 mmol, 82%, 86:14 er). **[α]$_D$^{23}**= − 190.5 (*c* 1, DMSO). **^{1}H NMR** (500 MHz, DMSO-*d*$_6$): δ 11.28 (d, *J* = 1.6 Hz, 1H), 8.07-8.05 (m, 2H), 7.68-7.65 (m, 1H), 7.61-7.58 (m, 2H), 7.40 (d, *J* = 8.2 Hz, 1H), 7.16 (d, *J* = 8.1 Hz, 1H), 7.09-7.06 (m, 2H), 6.91-6.87 (m, 1H), 6.84-6.83 (m, 1H), 6.58 (s, 1H), 6.10 (dd, *J* = 3.7, 1.9 Hz, 1H), 5.99 (dd, *J* = 3.7, 2.7 Hz, 1H), 3.49 (s, 3H). **^{13}C NMR** (126 MHz, DMSO-*d*$_6$): δ 200.5, 182.1, 136.7, 133.1, 129.2, 128.4, 127.2, 127.1, 125.8, 124.9, 124.2, 121.5, 119.4, 19.3, 111.9, 111.1, 110.0, 106.0, 99.2, 88.0, 35.0. **HRMS** (ESI) *m/z* calcd. for $C_{23}H_{18}O_2N_2Na$ [M^++Na] 377.1260, found 377.1254.

Enantioselectivity was determined by chiral SFC using Trefoil CEL1 column (20% MeOH, flow rate 2 mL/min): τ_{major} = 1.0 min, τ_{minor} = 1.4 min.

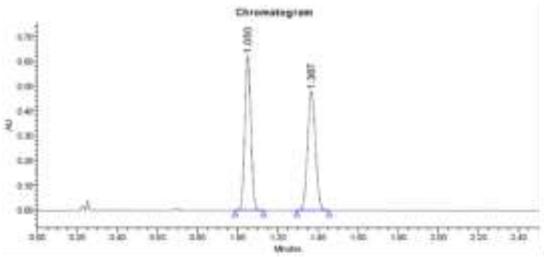

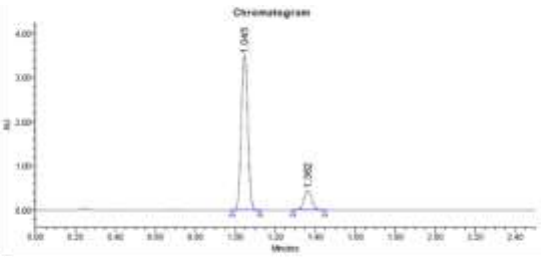

Peak Results

	Retention Time (min)	Relative Area (%)	Area	Height (µV)
1	1.050	49.98	1321300	626215
2	1.367	50.02	1322196	481072

Peak Results

	Retention Time (min)	Relative Area (%)	Area	Height (µV)
1	1.045	86.45	7929423	3499277
2	1.362	13.55	1242480	444367

(*S*)-2-(1*H*-indol-3-yl)-5'-methyl-5-phenyl-[2,2'-bifuran]-3(2*H*)-one [(*S*)-3Ca]

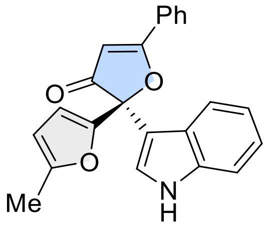

Following the general procedure from **1C** (47.6 mg, 0.2 mmol) and indole **2a** (23.7 mg, 0.2 mmol) for 48 h, compound (*S*)-**3Ca** was obtained as a pale-yellow solid after purification by column chromatography on silica gel (Hexane → EtOAc-hexane 1:2) (60.8 mg, 0.17 mmol, 86%, 62:38 er). **$[\alpha]_D^{23}$**= − 16.2 (*c* 1, DMSO). **^{1}H NMR** (500 MHz, DMSO-d_6): δ 11.33 (d, *J* = 1.5 Hz, 1H), 8.03-8.01 (m, 2H), 7.65 (t, *J* = 7.4 Hz, 1H), 7.59-7.55 (m, 3H), 7.43 (d, *J* = 8.2 Hz, 1H), 7.35 (d, *J* = 2.7 Hz, 1H), 7.13-7.10 (m, 1H), 7.01-6.98 (m, 1H), 6.59 (s, 1H), 6.16 (d, *J* = 3.2 Hz, 1H), 6.07 (dd, *J* = 3.2, 1.0 Hz, 1H), 2.23 (s, 3H). **^{13}C NMR** (126.6 MHz, DMSO-d_6): δ 199.5, 183.2, 153.0, 148.3, 136.6, 133.2, 129.2, 128.3, 127.2, 125.3, 124.5, 121.6, 120.1, 119.3, 112.0, 111.9, 109.1, 106.6, 99.2, 86.8, 13.4. **HRMS** (ESI) *m/z* calcd. for $C_{23}H_{17}O_3NNa$ [M^++Na] 378.1101, found 378.1097.

Enantioselectivity was determined by chiral SFC using Trefoil CEL1 column (20% MeOH, flow rate 2 mL/min): τ_{major} = 1.0 min, τ_{minor} = 1.3 min.

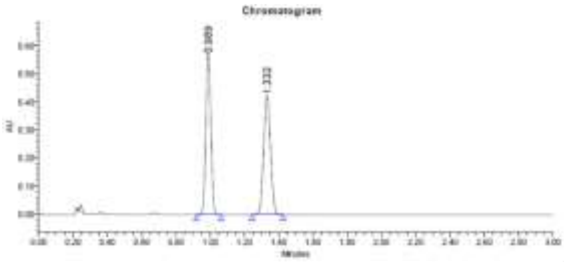

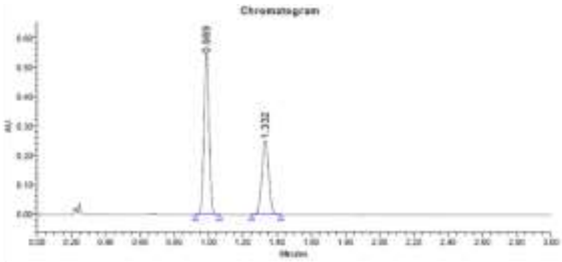

Peak Results

	Retention Time (min)	Relative Area (%)	Area	Height (µV)
1	0.989	50.07	1177658	567555
2	1.332	49.93	1174532	422343

Peak Results

	Retention Time (min)	Relative Area (%)	Area	Height (µV)
1	0.989	61.62	1127008	542876
2	1.332	38.38	702050	251666

(*R*)-2-(1*H*-indol-3-yl)-2-(4-methoxyphenyl)-5-phenylfuran-3(2*H*)-one [(*R*)-3Da]

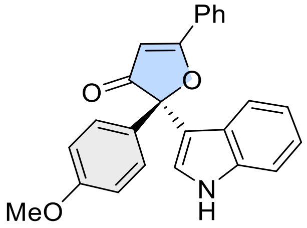

Following the general procedure from **1D** (52.9 mg, 0.2 mmol) and indole **2a** (23.7 mg, 0.2 mmol) for 48 h, the compound (*R*)-**3Da** was obtained as a pale-yellow solid after purification by column chromatography on silica gel (Hexane → EtOAc-hexane 1:2) (76.1, 0.20 mmol, 99%, 64:36 er). $[\alpha]_D^{23}$= + 125.7 (*c* 1, DMSO). **^{1}H NMR** (500 MHz, DMSO-d_6): δ 11.28 (d, *J* = 2.0 Hz 1H), 8.07-8.05 (m, 2H), 7.68-7.64 (m, 1H), 7.59 (t, *J* = 7.5 Hz, 2H), 7.41 (d, *J* = 8.2 Hz, 1H), 7.38-7.35 (m, 2H), 7.30 (d, *J* = 8.0 Hz, 1H), 7.22 (d, *J* = 2.7 Hz, 1H), 7.10-7.07 (m, 1H), 6.95-6.91 (m, 3H), 6.54 (s, 1H), 3.73 (s, 3H). **^{13}C NMR** (126 MHz, DMSO-d_6): δ 201.8, 183.1, 159.1, 136.7, 133.1, 129.9, 129.2, 128.4, 127.2, 126.9, 125.5, 125.3, 121.6, 119.7, 119.3, 113.7, 112.1, 111.9, 99.0, 90.7, 55.1. **HRMS** (ESI) *m/z* calcd. for $C_{25}H_{19}O_3NNa$ [M$^+$+Na] 404.1257, found 404.1250. Enantioselectivity was determined by chiral SFC using Trefoil CEL1 column (20% MeOH, flow rate 2 mL/min): τ_{minor} = 1.8 min, τ_{major} = 2.2 min.

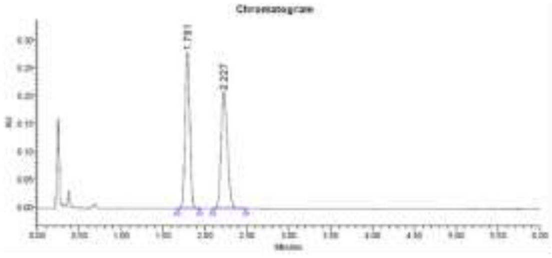

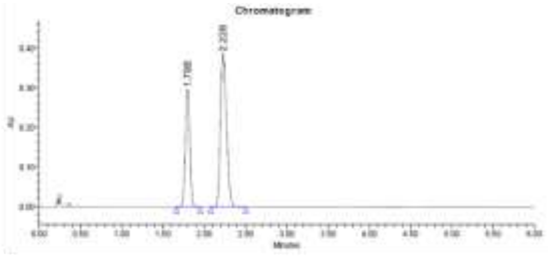

Peak Results

	Retention Time (min)	Relative Area (%)	Area	Height (µV)
1	1.791	50.03	1086739	279441
2	2.227	49.97	1085403	208684

Peak Results

	Retention Time (min)	Relative Area (%)	Area	Height (µV)
1	1.798	36.17	1133330	298119
2	2.228	63.83	2000176	389623

(*R*)-2-(1-benzyl-1*H*-indol-3-yl)-2-(1-methyl-1*H*-indol-3-yl)-5-phenylfuran-3(2*H*)-one [(*R*)-3Ab]

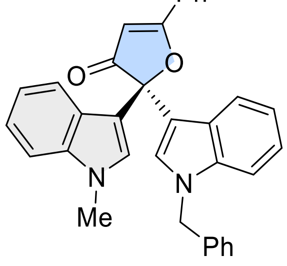

Following the general procedure from **1A** (57.5 mg, 0.2 mmol) and indole **2b** (41.5 mg, 0.2 mmol) for 16 h, compound (*R*)-**3Ab** was obtained as a yellow solid after purification by column

chromatography on silica gel (Hexane → EtOAc-hexane 1:2) (99.6 mg, 0.2 mmol, 99%, 87:13 er). **[α]$_D^{25}$**= – 2.6 (*c* 1, DMSO). **^{1}H NMR** (500 MHz, DMSO-*d$_6$*): δ 8.06-8.04 (m, 2H), 7.66-7.63 (m, 1H), 7.59-7.56 (t, *J* = 7.5 Hz, 2H), 7.50-7.45 (m, 2H), 7.44-7.42 (m, 3H), 7.31-7.28 (m, 2H), 7.26 (s, 1H), 7.25-7.22 (m, 1H), 7.18-7.13 (m, 3H), 7.10-7.07 (m, 1H), 6.98-6.93 (m, 2H), 6.62 (s, 1H), 5.42 (s, 2H), 3.76 (s, 3H). **^{13}C NMR** (126 MHz, DMSO-*d$_6$*): δ 201.8, 182.7, 138.0, 137.2, 136.6, 133.0, 129.2, 128.7, 128.6, 128.5, 127.3, 127.2, 127.0, 126.9, 126.0, 125.7, 121.6, 121.5, 120.3, 119.5, 119.2, 111.5, 111.0, 110.6, 110.1, 99.0, 89.2, 49.0, 32.5. **HRMS** (ESI) *m/z* calcd. for $C_{34}H_{26}O_2N_2Na$ [M$^+$+Na] 517.1886, found 517.1875.

Enantioselectivity was determined by chiral HPLC analysis using Chiralpak-IA column (hexane/propan-2-ol 80:20, flow rate 1 mL/min): τ_{major} = 14.6 min, τ_{minor} = 18.3 min.

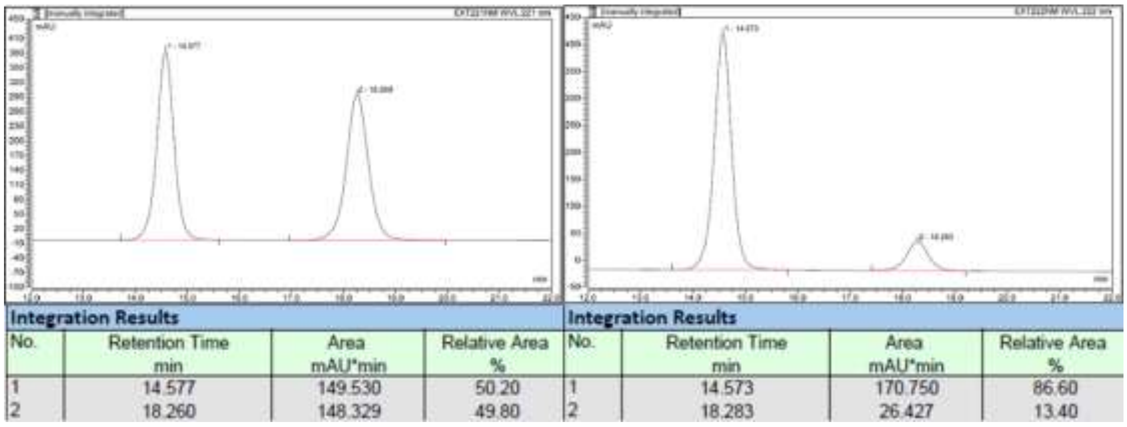

Integration Results

No.	Retention Time min	Area mAU*min	Relative Area %
1	14.577	149.530	50.20
2	18.260	148.329	49.80

Integration Results

No.	Retention Time min	Area mAU*min	Relative Area %
1	14.573	170.750	86.60
2	18.283	26.427	13.40

(*S*)-2-(1-benzyl-1*H*-indol-3-yl)-2-(1-methyl-1*H*-indol-3-yl)-5-phenylfuran-3(2*H*)-one [(*S*)-3Ec]

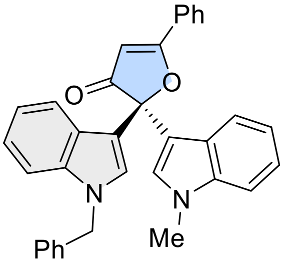

Following the general procedure from **1E** (72.7 mg, 0.2 mmol) and indole **2c** (27.3 mg, 0.2 mmol) for 16 h, compound (*S*)-**3Ec** was obtained as a yellow solid after purification by column chromatography on silica gel (Hexane → EtOAc-hexane 1:2) (95.5 mg, 0.19 mmol, 97%, 85:15 er). **[α]$_D^{25}$**= + 4.4 (*c* 1, DMSO). Spectral data are in accordance with enantiomer (*R*)-**3Ab**.

Enantioselectivity was determined by chiral HPLC analysis using Chiralpak-IA column (hexane/propan-2-ol 80:20, flow rate 1 mL/min): τ_{minor} = 14.6 min, τ_{major} = 18.2 min.

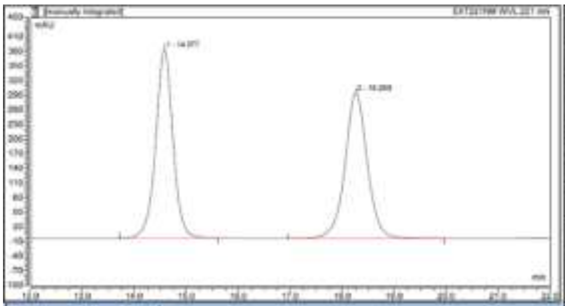

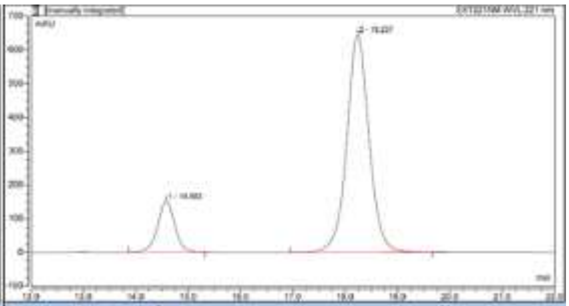

Integration Results

No.	Retention Time min	Area mAU*min	Relative Area %
1	14.577	149.530	50.20
2	18.260	148.329	49.80

Integration Results

No.	Retention Time min	Area mAU*min	Relative Area %
1	14.583	57.895	15.39
2	18.237	318.348	84.61

(*R*)-2-(1-methyl-1*H*-indol-3-yl)-2-(2-methyl-1*H*-indol-3-yl)-5-phenylfuran-3(2*H*)-one [(*R*)-3Ad]

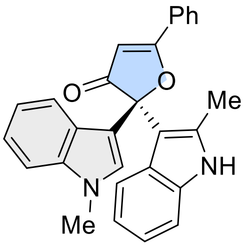

Following the general procedure from **1A** (57.5 mg, 0.2 mmol) and indole **2d** (27.0 mg, 0.2 mmol) for 16 h, compound (*R*)-**3Ad** was obtained as a brown solid after purification by column chromatography on silica gel (Hexane → EtOAc-hexane 1:6 → 1:1) (78.5 mg, 0.19 mmol, 95%, 89:11 er). $[\alpha]_D^{24}$= − 227.9 (*c* 1, DMSO). Spectroscopic data were consistent with literature values.[18] **^{1}H NMR** (500 MHz, DMSO-d_6): δ 11.08 (s, 1H), 8.04-8.02 (m, 2H), 7.66-7.63 (m, 1H), 7.59-7.56 (m, 2H), 7.45-7.39 (m, 2H), 7.29 (s, 1H), 7.25 (d, *J* = 8.0 Hz, 1H), 7.20 (d, *J* = 8.1 Hz, 1H), 7.14 (t, *J* = 7.4 Hz, 1H), 6.96-6.91 (m, 2H), 6.75 (t, *J* = 7.5 Hz, 1H), 6.61 (s, 1H), 3.79 (s, 3H), 2.06 (s, 3H). **^{13}C NMR** (126 MHz, DMSO-d_6): δ 202.2, 182.1, 137.2, 134.8, 134.3, 132.9, 129.2, 128.7, 128.4, 127.1, 126.8, 126.4, 121.5, 120.2, 120.1, 119.5, 119.3, 118.7, 111.7, 110.5, 110.0, 106.4, 99.4, 90.2, 32.5, 13.1.

Enantioselectivity was determined by chiral HPLC analysis using Chiralpak-IA column (hexane/propan-2-ol 80:20, flow rate 1 mL/min): τ_{minor} = 12.8 min, τ_{major} = 20.4 min.

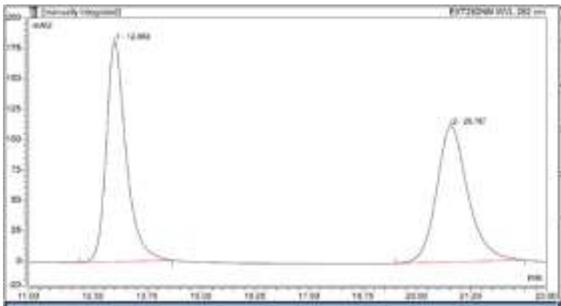

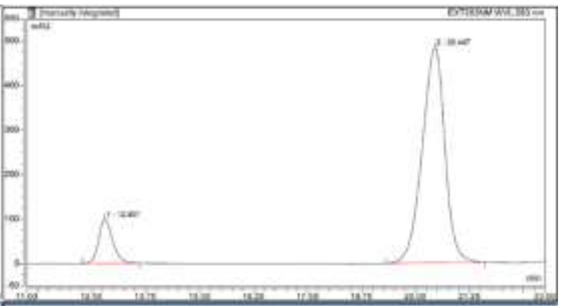

Integration Results

No.	Retention Time min	Area mAU*min	Relative Area %
1	12.993	96.842	50.73
2	20.787	94.074	49.27

Integration Results

No.	Retention Time min	Area mAU*min	Relative Area %
1	12.807	37.328	11.28
2	20.447	293.530	88.72

(*R*)-2-(1-benzyl-2-methyl-1*H*-indol-3-yl)-2-(1-methyl-1*H*-indol-3-yl)-5-phenylfuran-3(2*H*)-one [(*R*)-3Ae]

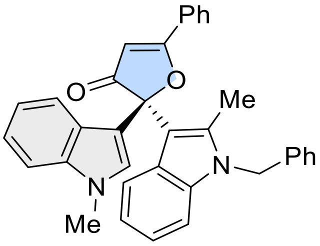

Following the general procedure from **1A** (57.5 mg, 0.2 mmol) and indole **2e** (44.3 mg, 0.2 mmol) for 18 h, compound (*R*)-**3Ae** was obtained as a brown solid after purification by column chromatography on silica gel (Hexane → EtOAc-hexane 1:5 → 1:2) (91 mg, 0.18 mmol, 90%, 83:17 er). $[\alpha]_D^{25}$= – 188.3 (*c* 1, DMSO). **^{1}H NMR** (500 MHz, DMSO-d_6): δ 8.05-8.03 (m, 2H), 7.66-7.63 (m, 1H), 7.60-7.57 (m, 2H), 7.44-7.33 (m, 5H), 7.28-7.12 (m, 4H), 7.01-6.98 (m, 1H), 6.94-6.91 (m, 3H), 6.84-6.81 (m, 1H), 6.65 (s, 1H), 5.41 (s, 2H), 3.79 (s, 3H), 2.03 (s, 3H). **^{13}C NMR** (126 MHz, DMSO-d_6): δ 202.1, 182.1, 138.0, 137.2, 136.0, 135.8, 133.0, 129.3, 128.6, 128.6, 128.3, 127.2, 127.0, 126.3, 125.8, 121.6, 120.7, 120.0, 119.8, 119.4, 119.2, 112.0, 110.1, 109.7, 107.5, 99.6, 90.1, 45.4, 32.5, 11.2. **HRMS** (ESI) *m/z* calcd. for $C_{35}H_{28}O_2N_2Na$ $[M^+ +Na]$ 531.2043, found 531.2042.

Enantioselectivity was determined by chiral HPLC analysis using Chiralpak-IA column (hexane/propan-2-ol 80:20, flow rate 1 mL/min): τ_{minor} = 14.0 min, τ_{major} = 17.8 min.

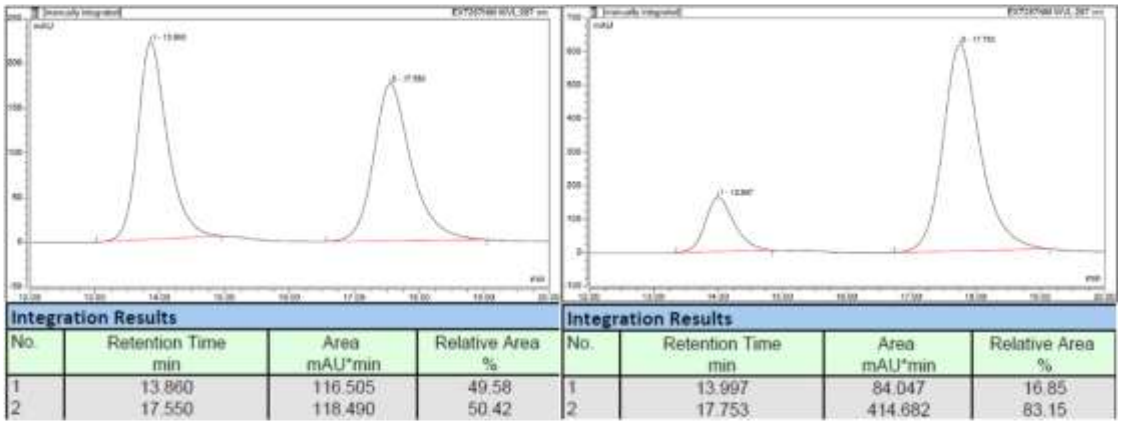

Integration Results

No.	Retention Time min	Area mAU*min	Relative Area %
1	13.860	116.505	49.58
2	17.550	118.490	50.42

Integration Results

No.	Retention Time min	Area mAU*min	Relative Area %
1	13.997	84.047	16.85
2	17.753	414.682	83.15

(*R*)-2-(1-methyl-1*H*-indol-3-yl)-2-(2-phenyl-1*H*-indol-3-yl)-5-phenylfuran-3(2*H*)-one [(*R*)-3Af]

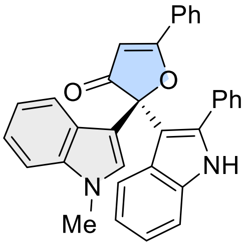

Following the general procedure from **1A** (57.5 mg, 0.2 mmol) and indole **2f** (39.4 mg, 0.2 mmol) for 40 h, compound (*R*)-**3Af** was obtained as a brown solid after purification by column chromatography on silica gel (Hexane → EtOAc-hexane 1:4 → 1:2) (90.0 mg, 0.19 mmol, 94%, 88:12 er). $[\alpha]_D^{25}$= – 279.4 (*c* 1, DMSO). **^{1}H NMR** (500 MHz, DMSO-d_6): δ 11.37 (s, 1H), 7.67 (dd, *J* = 8.2, 1.0 Hz, 2H), 7.56 (t, *J* = 7.4 Hz, 1H), 7.44 (t, *J* = 7.8 Hz, 2H), 7.32 (dd, *J* = 8.2, 3.8 Hz, 2H), 7.29 (d, *J* = 8.0 Hz, 1H), 7.24-7.21 (m, 3H), 7.13 (s, 1H), 7.11-7.01 (m, 5H), 6.87-6.82 (m, 2H), 6.53 (s, 1H), 3.62 (s, 3H). **^{13}C NMR** (126 MHz, DMSO-d_6): δ 202.1, 182.3, 137.9, 136.9, 135.6, 133.3, 132.7, 129.4, 129.2, 128.8, 128.5, 127.1, 127.0, 126.6, 126.2, 121.2, 120.7, 120.0, 119.0, 118.9, 111.4, 111.1, 109.6, 107.6, 99.0, 89.8, 32.2. **HRMS** (ESI) *m/z* calcd. for $C_{33}H_{24}O_2N_2Na$ [M$^+$+Na] 503.1730, found 503.1728.

Enantioselectivity was determined by chiral SFC using Trefoil AMY1 column (20% MeOH, flow rate 2 mL/min): τ_{major} = 4.9 min, τ_{minor} = 6.2 min.

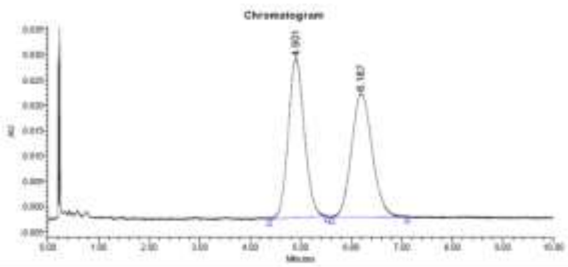

Peak Results

	Retention Time (min)	Relative Area (%)	Area	Height (µV)
1	4.901	50.07	695579	31406
2	6.187	49.93	693671	24394

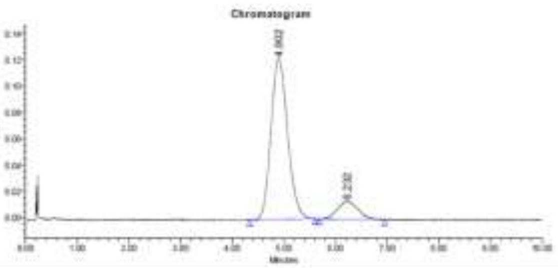

Peak Results

	Retention Time (min)	Relative Area (%)	Area	Height (µV)
1	4.902	88.03	2744035	122256
2	6.232	11.97	372959	13404

(*R*)-2-(1-methyl-1*H*-indol-3-yl)-2-(1-methyl-2-phenyl-1*H*-indol-3-yl)-5-phenylfuran-3(2*H*)-one [(*R*)-3Ag]

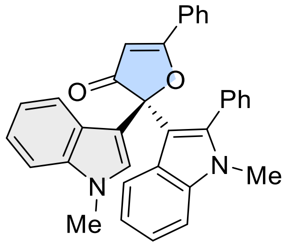

Following the general procedure from **1A** (57.5 mg, 0.2 mmol) and indole **2g** (41.5 mg, 0.2 mmol) for 36 h, compound (*R*)-**3Ag** was obtained as a yellowish solid after purification by column chromatography on silica gel (Hexane → EtOAc-hexane 1:2 → 1:1) (88.3 mg, 0.18 mmol, 89%, 87:13 er). $[\alpha]_D^{25}$= – 438.8 (*c* 1, DMSO). **^{1}H NMR** (500 MHz, DMSO-d_6): δ 7.61 (dd, *J* = 8.3, 1.1 Hz, 2H), 7.55 (t, *J* = 7.4 Hz, 1H), 7.44-7.41 (m, 4H), 7.37 (d, *J* = 7.5 Hz, 1H), 7.27-7.21 (m, 3H), 7.14-7.05 (m, 4H), 6.92-6.80 (m, 4H), 6.45 (s, 1H), 3.62 (s, 3H), 3.33 (s, 3H). **^{13}C NMR** (126 MHz, DMSO-d_6): δ 201.9, 182.2, 139.0, 137.0, 136.3, 132.6, 131.7, 130.5, 129.6, 129.0, 128.7, 128.4, 127.4, 127.1, 126.9, 126.7, 126.4, 125.4, 121.4, 121.3, 120.7, 120.1, 119.3, 119.1, 111.1, 109.8, 109.7, 108.3, 98.8, 89.4, 32.2, 30.2. **HRMS** (ESI) *m/z* calcd. for $C_{34}H_{26}O_2N_2Na$ [M^++Na] 517.1886, found 517.1883.

Enantioselectivity was determined by chiral SFC using Trefoil CEL1 column (20% MeOH, flow rate 2 mL/min): τ_{major} = 1.7 min, τ_{minor} = 2.0 min.

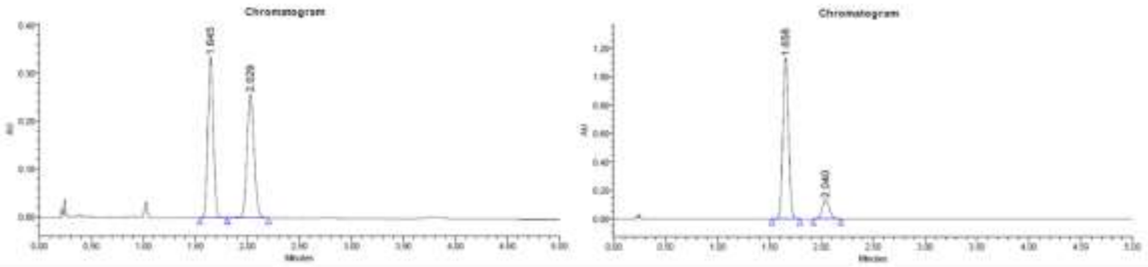

Peak Results

	Retention Time (min)	Relative Area (%)	Area	Height (µV)
1	1.645	49.99	1244499	337001
2	2.029	50.01	1245025	258232

Peak Results

	Retention Time (min)	Relative Area (%)	Area	Height (µV)
1	1.656	86.74	4304707	1134935
2	2.040	13.26	657962	135360

(*R*)-2-(1-methyl-1*H*-indol-3-yl)-2-(3-methyl-1*H*-indol-2-yl)-5-phenylfuran-3(2*H*)-one [(*R*)-3Ah]

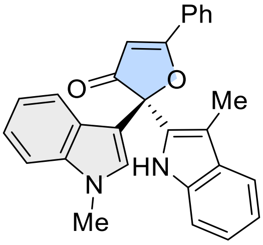

Following the general procedure from **1A** (57.5 mg, 0.2 mmol) and indole **2h** (27.0 mg, 0.2 mmol) for 48 h, compound (*R*)-**3Ah** was obtained as a brown solid after purification by column chromatography on silica gel (Toluene → EtOAc-toluene 1:10 → 1:5) (46.2 mg, 0.11 mmol, 55%, 91:9 er). $[\alpha]_D^{24}$= – 397.5 (*c* 1, DMSO). **^{1}H NMR** (300 MHz, DMSO-d_6): δ 10.53 (s, 1H), 8.09-8.06 (m, 2H), 7.69-7.57 (m, 3H), 7.48-7.44 (m, 2H), 7.36-7.32 (m, 3H), 7.19-7.14 (m, 1H), 7.09-7.03 (m, 1H), 7.00-6.94 (m, 2H), 6.68 (s, 1H), 3.80 (s, 3H), 2.13 (s, 3H). **^{13}C NMR** (75.5 MHz, DMSO-d_6): δ 200.5, 183.2, 137.2, 135.2, 133.2, 129.6, 129.1, 129.0, 128.8, 128.4, 127.4, 126.1, 121.7, 121.4, 119.6, 119.5, 118.4, 118.1, 111.6, 110.2, 109.8, 107.9, 99.3, 88.3, 32.5, 9.0. **HRMS** (ESI) *m/z* calcd. for $C_{28}H_{23}O_2N_2$ [M^++H] 419.1748, found 419.1754.

Enantioselectivity was determined by chiral HPLC analysis using Chiralpak-IB column (hexane/propan-2-ol 90:10, flow rate 1 mL/min): τ_{minor} = 15.3 min, τ_{major} = 42.6 min.

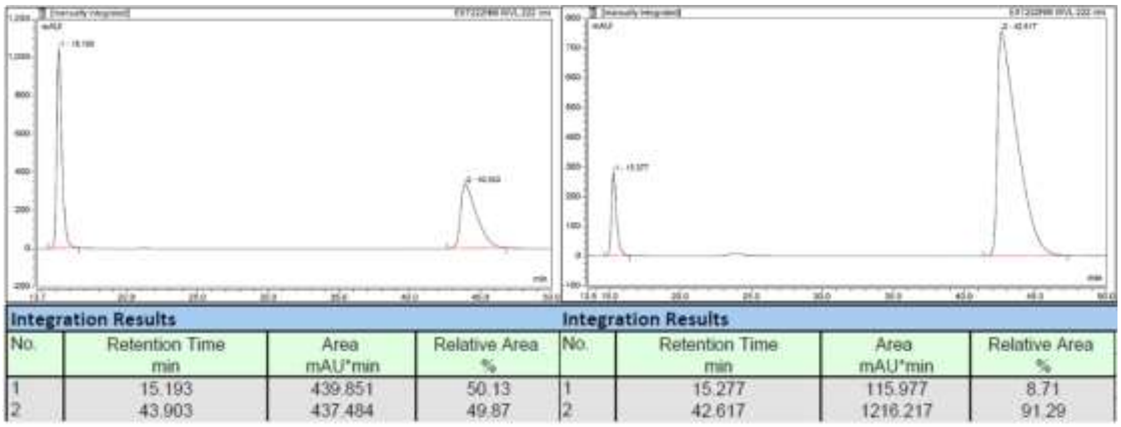

Integration Results

No.	Retention Time min	Area mAU*min	Relative Area %
1	15.193	439.851	50.13
2	43.903	437.484	49.87

Integration Results

No.	Retention Time min	Area mAU*min	Relative Area %
1	15.277	115.977	8.71
2	42.617	1216.217	91.29

(*S*)-2-(4-methoxy-1*H*-indol-3-yl)-2-(1-methyl-1*H*-indol-3-yl)-5-phenylfuran-3(2*H*)-one [(*S*)-3Ai]

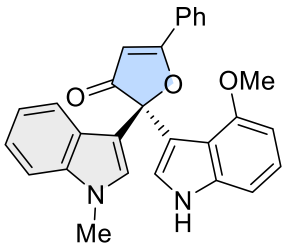

Following the general procedure from **1A** (57.5 mg, 0.2 mmol) and indole **2i** (29.4 mg, 0.2 mmol) for 36 h, compound (*S*)-**3Ai** was obtained as a green solid after purification by column chromatography on silica gel (Hexane → EtOAc-hexane 1:3 → 1:1) (79.6 mg, 0.18 mmol, 92%, 86:14 er). $[\alpha]_D^{25}$= + 13.7 (*c* 1, DMSO). Spectroscopic data were consistent with literature values.[18] **^{1}H NMR** (500 MHz, DMSO-d_6): δ 11.08 (d, *J* = 2.3 Hz, 1H), 7.99 (dd, *J* = 8.2, 1.3 Hz, 2H), 7.70 (d, *J* = 8.0 Hz, 1H), 7.60-7.52 (m, 3H), 7.44 (d, *J* = 8.3 Hz, 1H), 7.28 (s, 1H), 7.17-7.14 (m, 1H), 7.03-6.98 (m, 3H), 6.65 (d, *J* = 2.7 Hz, 1H), 6.48 (s, 1H), 6.42 (dd, *J* = 7.4, 1.0 Hz, 1H), 3.78 (s, 3H), 3.49 (s, 3H). **^{13}C NMR** (126 MHz, DMSO-d_6): δ 202.4, 181.8, 153.2, 138.7, 137.1, 132.2, 129.6, 129.0, 127.7, 127.5, 126.7, 126.4, 122.5, 121.4, 121.3, 119.0, 115.7, 111.8, 111.4, 109.9, 104.9, 99.9, 99.3, 88.6, 54.3, 32.4.

Enantioselectivity was determined by chiral SFC using Trefoil CEL1 column (20% MeOH, flow rate 2 mL/min): τ_{minor} = 2.8 min, τ_{major} = 3.3 min.

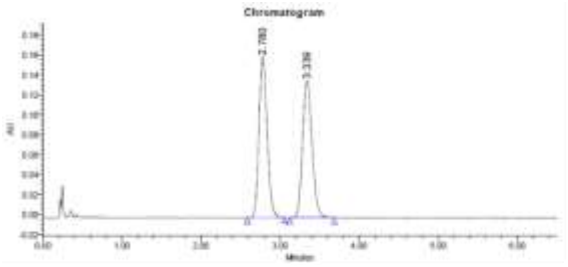

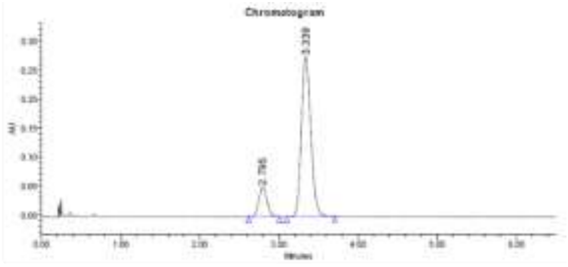

Peak Results

	Retention Time (min)	Relative Area (%)	Area	Height (µV)
1	2.780	49.98	1148305	161299
2	3.339	50.02	1149089	137040

Peak Results

	Retention Time (min)	Relative Area (%)	Area	Height (µV)
1	2.795	13.80	367830	51506
2	3.339	86.20	2297595	275570

(*S*)-2-(4-chloro-1*H*-indol-3-yl)-2-(1-methyl-1*H*-indol-3-yl)-5-phenylfuran-3(2*H*)-one [(*S*)-3Aj]

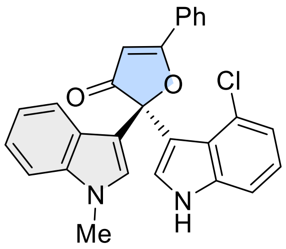

Following the general procedure from **1A** (57.5 mg, 0.2 mmol) and indole **2j** (30.9 mg, 0.2 mmol) for 48 h, compound (*S*)-**3Aj** was obtained as a yellow solid after purification by column chromatography on silica gel (Hexane → EtOAc-hexane 1:2) (44.3 mg, 0.10 mmol, 50%, 83:17 er). $[\alpha]_D^{25}$= + 341.0 (*c* 1, DMSO). Spectroscopic data were consistent with literature values.[18] **^{1}H NMR** (500 MHz, DMSO-d_6): δ 11.65 (d, *J* = 2.6 Hz, 1H), 8.08-8.05 (m, 2H), 7.65-7.62 (m, 1H), 7.59-7.56 (m, 2H), 7.44 (dd, *J* = 8.1, 1.0 Hz, 1H), 7.40 (d, *J* = 8.3 Hz, 1H), 7.36 (d, *J* = 2.8 Hz, 1H), 7.28 (d, *J* = 8.0 Hz, 1H), 7.13-7.09 (m, 2H), 7.05 (dd, *J* = 7.6, 0.9 Hz, 1H), 6.92-6.89 (m, 2H), 6.63 (s, 1H), 3.70 (s, 3H). **^{13}C NMR** (126 MHz, DMSO-d_6): δ 202.2, 182.4, 139.0, 137.1, 132.9, 129.2, 128.8, 127.7, 127.1, 125.1, 124.6, 123.4, 122.5, 121.2, 121.0, 119.6, 119.1, 113.2, 111.1, 110.3, 110.0, 99.5, 88.8, 32.4.

Enantioselectivity was determined by chiral HPLC analysis using Chiralpak-IA column (hexane/propan-2-ol 80:20, flow rate 1 mL/min): τ_{major} = 24.5 min, τ_{minor} = 29.3 min.

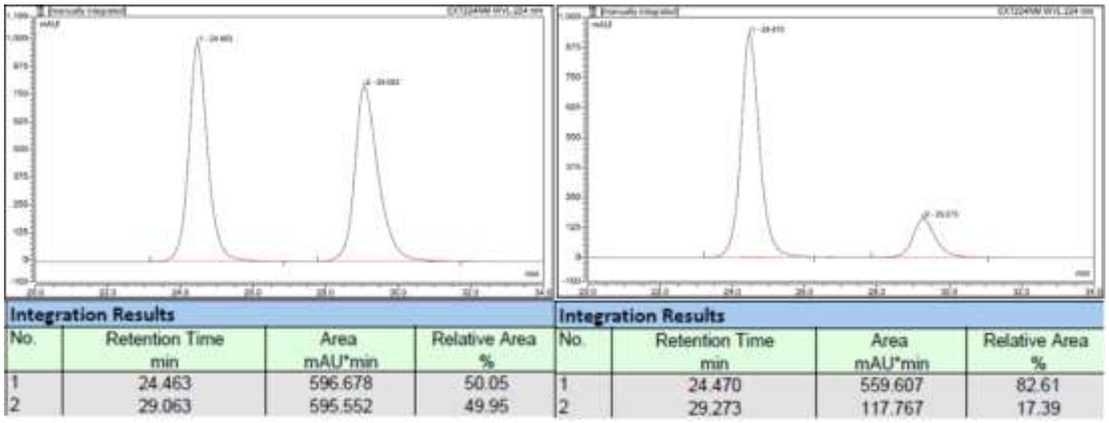

Integration Results

No.	Retention Time min	Area mAU*min	Relative Area %
1	24.463	596.678	50.05
2	29.063	595.552	49.95

Integration Results

No.	Retention Time min	Area mAU*min	Relative Area %
1	24.470	559.607	82.61
2	29.273	117.767	17.39

(*S*)-3-(2-(1-methyl-1*H*-indol-3-yl)-3-oxo-5-phenyl-2,3-dihydrofuran-2-yl)-1*H*-indole-5-carbaldehyde [(*S*)-3Ak]

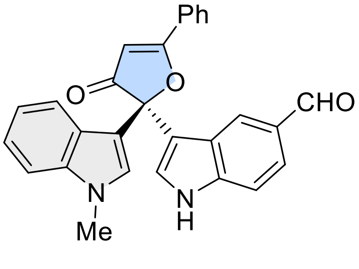

Following the general procedure from **1A** (57.5 mg, 0.2 mmol) and indole **2k** (29.0 mg, 0.2 mmol) for 24 h, compound (*S*)-**3Ak** was obtained as a yellow solid after purification by column chromatography on silica gel (Hexane → EtOAc-hexane 1:2 → 1:1) (78.1 mg, 0.18 mmol, 91%, 88:12 er). $[\alpha]_D^{25}$= − 25.3 (*c* 1, DMSO). Spectroscopic data were consistent with literature values.[18] **^{1}H NMR** (500 MHz, DMSO-d_6): δ 11.77 (d, *J* = 2.1 Hz, 1H), 9.87 (s, 1H), 8.14 (s, 1H), 8.09-8.07 (m, 2H), 7.67-7.64 (m, 2H), 7.60-7.57 (m, 3H), 7.48 (d, *J* = 2.6 Hz, 1H), 7.43 (dd, *J* = 11.5, 8.1 Hz, 2H), 7.22 (s, 1H), 7.15 (ddd, *J* = 8.2, 7.1, 1.1 Hz, 1H), 6.96 (ddd, *J* = 8.0, 7.1, 0.9 Hz, 1H), 6.65 (s, 1H), 3.75 (s, 3H). **^{13}C NMR** (126 MHz, DMSO-d_6): δ 201.8, 192.5, 182.9, 140.3,

137.2, 133.1, 129.4, 129.1, 128.9, 128.6, 127.2, 126.6, 125.6, 125.2, 125.1, 121.7, 121.6, 119.8, 119.4, 113.8, 112.7, 110.9, 110.2, 99.0, 89.0, 32.5.

Enantioselectivity was determined by chiral HPLC analysis using Chiralpak-IC column (hexane/propan-2-ol 80:20, flow rate 1 mL/min): τ_{minor} = 30.6 min, τ_{major} = 35.2 min.

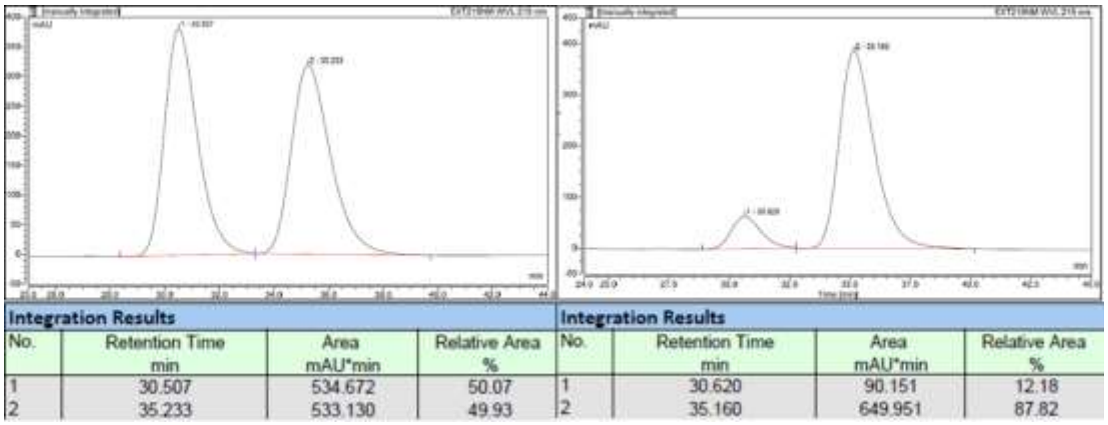

Integration Results

No.	Retention Time min	Area mAU*min	Relative Area %
1	30.507	534.672	50.07
2	35.233	533.130	49.93

Integration Results

No.	Retention Time min	Area mAU*min	Relative Area %
1	30.620	90.151	12.18
2	35.160	649.951	87.82

(*S*)-2-(7-methoxy-1*H*-indol-3-yl)-2-(1-methyl-1*H*-indol-3-yl)-5-phenylfuran-3(2*H*)-one [(*S*)-3Al]

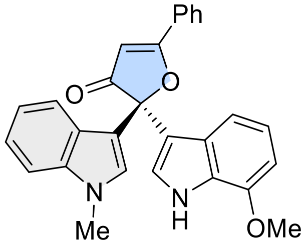

Following the general procedure from **1A** (57.5 mg, 0.2 mmol) and indole **2l** (30.9 mg, 0.2 mmol) for 48 h, compound (*S*)-**3Al** was obtained as an off-white solid after purification by column chromatography on silica gel (EtOAc-hexane 1:2) (51.9 mg, 0.12 mmol, 60%, 90:10 er). $[\alpha]_D^{25}$= + 3.3 (*c* 1, DMSO). **^{1}H NMR** (500 MHz, DMSO-d_6): δ 11.32 (s, 1H), 8.04-8.03 (m, 2H), 7.66-7.63 (m, 1H), 7.59-7.56 (m, 2H), 7.44-7.42 (m, 2H), 7.20 (s, 1H), 7.34 (t, *J* = 7.3 Hz, 1H), 7.05 (d, *J* = 8.1 Hz, 1H), 7.03 (d, *J* = 2.7 Hz, 1H), 6.95 (t, *J* = 7.5 Hz, 1H), 6.85 (t, *J* = 7.8 Hz, 1H), 6.65 (d, *J* = 7.8 Hz, 1H), 6.60 (s, 1H), 3.90 (s, 3H), 3.75 (s, 3H). **^{13}C NMR** (126 MHz, DMSO-d_6): δ 202.0, 182.6, 146.3, 137.2, 133.0, 129.2, 128.9, 128.7, 127.1, 126.9, 126.8, 125.7, 124.4, 121.5, 120.2, 119.8, 119.3, 112.7, 112.4, 111.1, 110.1, 101.9, 99.0, 89.4, 55.2, 32.5. **HRMS** (ESI) *m/z* calcd. for $C_{28}H_{22}O_3N_2Na$ [M^++Na] 457.1523, found 457.1519.

Enantioselectivity was determined by chiral HPLC analysis using Chiralpak-IA column (hexane/propan-2-ol 80:20, flow rate 1 mL/min): τ_{minor} = 30.6 min, τ_{major} = 39.2 min.

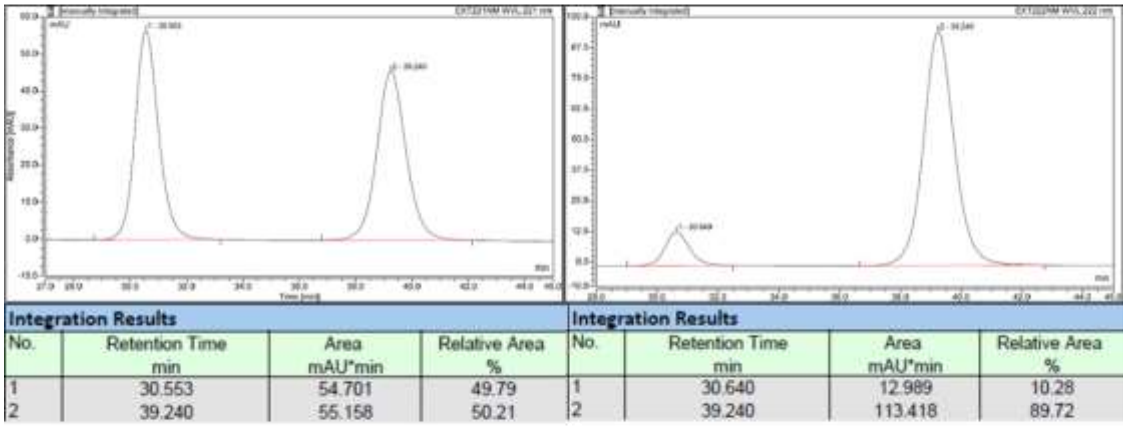

Integration Results			
No.	Retention Time min	Area mAU*min	Relative Area %
1	30.553	54.701	49.79
2	39.240	55.158	50.21

Integration Results			
No.	Retention Time min	Area mAU*min	Relative Area %
1	30.640	12.989	10.28
2	39.240	113.418	89.72

(*S*)-2-(7-fluoro-1*H*-indol-3-yl)-2-(1-methyl-1*H*-indol-3-yl)-5-phenylfuran-3(2*H*)-one [(*S*)-3Am]

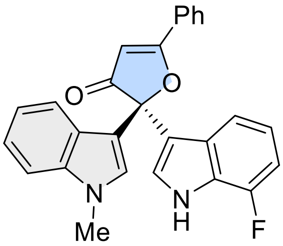

Following the general procedure from **1A** (57.5 mg, 0.2 mmol) and indole **2m** (27.0 mg, 0.2 mmol) for 24 h, compound (*S*)-**3Am** was obtained as an off-white solid after purification by column chromatography on silica gel (EtOAc-hexane 1:2 → 1:1) (44.7 mg, 0.11 mmol, 53%, 93:7 er). $[\alpha]_D^{25}$= – 8.1 (*c* 1, DMSO). Spectroscopic data were consistent with literature values.[18] **^{1}H NMR** (500 MHz, DMSO-d_6): δ 11.78 (d, *J* = 2.1 Hz, 1H), 8.06-8.04 (m, 2H), 7.66-7.63 (m, 1H), 7.59-7.56 (m, 2H), 7.46-7.42 (m, 2H), 7.30 (dd, *J* = 7.2, 1.1 Hz, 1H), 7.25-7.24 (m, 2H), 7.16-7.13 (m, 1H), 6.98-6.88 (m, 3H), 6.63 (s, 1H), 3.75 (s, 3H). **^{13}C NMR** (126 MHz, DMSO-d_6): δ 201.8, 182.8, 149.3 (d, $J_{C,F}$ = 243.2 Hz), 137.2, 133.0, 129.2, 129.0, 128.6, 127.2, 125.8, 125.6, 124.7 (d, $J_{C,F}$ = 13.6 Hz), 121.5, 120.1, 119.6 (d, $J_{C,F}$ = 5.9 Hz), 119.3, 116.2 (d, $J_{C,F}$ = 2.6 Hz), 113.1 (d, $J_{C,F}$ = 1.7 Hz), 110.8, 110.1, 106.3 (d, $J_{C,F}$ = 15.6 Hz), 99.0, 89.0, 32.4. **^{19}F NMR** (471 MHz, DMSO-d_6): δ –133.3.

Enantioselectivity was determined by chiral SFC using Trefoil CEL2 column (20% MeOH, flow rate 2 mL/min): τ_{major} = 3.2 min, τ_{minor} = 5.3 min.

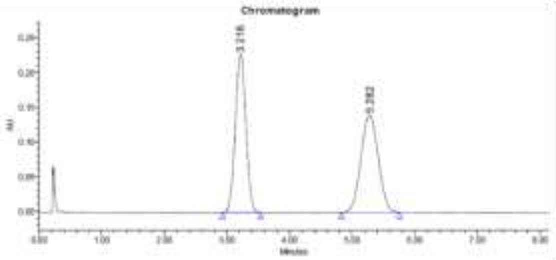

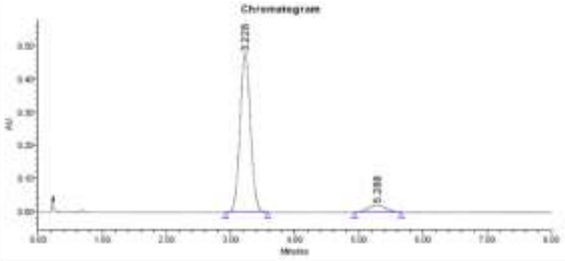

Peak Results

	Retention Time (min)	Relative Area (%)	Area	Height (μV)
1	3.216	50.19	2697817	228014
2	5.282	49.81	2677085	140206

Peak Results

	Retention Time (min)	Relative Area (%)	Area	Height (μV)
1	3.228	93.13	5491297	480402
2	5.286	6.87	405176	22112

(*S*)-2-(7-chloro-1*H*-indol-3-yl)-2-(1-methyl-1*H*-indol-3-yl)-5-phenylfuran-3(2*H*)-one [(*S*)-3An]

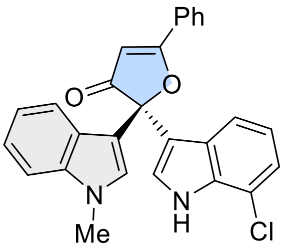

Following the general procedure from **1A** (57.5 mg, 0.2 mmol) and indole **2n** (30.3 mg, 0.2 mmol) for 24 h, compound (*S*)-**3An** was obtained as a yellow solid after purification by column chromatography on silica gel (EtOAc-hexane 1:2 → 1:1) (62.4 mg, 0.14 mmol, 71%, 99:1 er). **$[\alpha]_D^{25}$**= + 5.2 (*c* 1, DMSO). **^{1}H NMR** (500 MHz, DMSO-d_6): δ 11.63 (s, 1H), 8.05 (d, *J* = 7.4 Hz, 2H), 7.65 (t, *J* = 7.3 Hz, 1H), 7.58 (t, *J* = 7.5 Hz, 2H), 7.46-7.43 (m, 3H), 7.24-7.23 (m, 2H), 7.19-7.13 (m, 2H), 6.98-6.94 (m, 2H), 6.63 (s, 1H), 3.75 (s, 3H). **^{13}C NMR** (126 MHz, DMSO-d_6): δ 201.8, 182.8, 137.2, 133.5, 133.1, 129.2, 129.1, 128.6, 127.3, 127.2, 125.8, 125.6, 121.6, 121.1, 120.3, 120.0, 119.4, 119.1, 116.2, 113.3, 110.7, 110.2, 99.0, 89.0, 32.5. **HRMS** (ESI) *m/z* calcd. for $C_{27}H_{19}O_2N_2ClNa$ [M^++Na] 461.1027, found 461.1023.

Enantioselectivity was determined by chiral SFC using Trefoil CEL2 column (20% MeOH, flow rate 2 mL/min): τ_{major} = 4.4 min, τ_{minor} = 7.3 min.

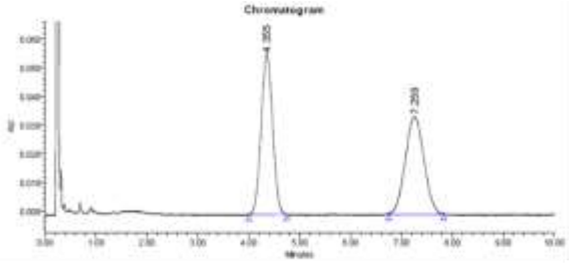

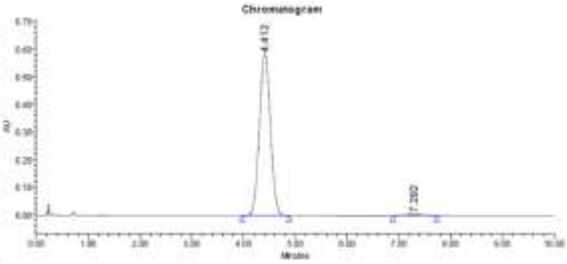

Peak Results

	Retention Time (min)	Relative Area (%)	Area	Height (μV)
1	4.355	50.15	873438	55950
2	7.259	49.85	868054	34166

Peak Results

	Retention Time (min)	Relative Area (%)	Area	Height (μV)
1	4.412	98.76	8733377	585934
2	7.292	1.24	109597	4901

(*S*)-2-(7-bromo-1*H*-indol-3-yl)-2-(1-methyl-1*H*-indol-3-yl)-5-phenylfuran-3(2*H*)-one [(*S*)-3Ao]

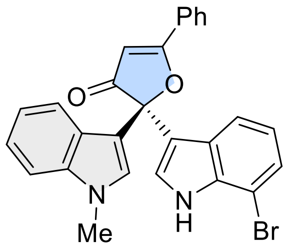

Following the general procedure from **1A** (57.5 mg, 0.2 mmol) and indole **2o** (40.0 mg, 0.2 mmol) for 36 h, compound (*S*)-**3Ao** was obtained as a yellowish solid after purification by column chromatography on silica gel (EtOAc-hexane 1:2 → 1:1) (67.4 mg, 0.14 mmol, 71%, 95:5 er). **$[\alpha]_D^{25}$**= − 11.9 (*c* 1, DMSO). **^{1}H NMR** (500 MHz, DMSO-*d_6*): δ 11.49 (d, *J* = 2.3 Hz, 1H), 8.05-8.04 (m, 2H), 7.66-7.62 (m, 1H), 7.59-7.56 (m, 2H), 7.49 (d, *J* = 8.0 Hz, 1H), 7.44-7.42 (m, 2H), 7.33 (dd, *J* = 7.6, 0.5 Hz, 1H), 7.23 (s, 1H), 7.22 (s, 1H), 7.16-7.13 (m, 1H), 6.98-6.95 (m, 1H), 6.90 (t, *J* = 7.8 Hz, 1H), 6.63 (s, 1H), 3.75 (s, 3H). **^{13}C NMR** (126 MHz, DMSO-*d_6*): δ 201.8, 182.9, 137.2, 135.0, 133.1, 129.3, 129.1, 128.6, 127.2, 127.1, 125.8, 125.6, 124.2, 121.6, 120.8, 120.0, 119.6, 119.4, 113.3, 110.7, 110.2, 104.6, 99.1, 89.1, 32.5. **HRMS** (ESI) *m/z* calcd. for $C_{27}H_{19}O_2N_2{}^{79}BrNa$ [M^++Na] 505.0522, found 505.0519. *m/z* calcd. for $C_{27}H_{19}O_2N_2{}^{81}BrNa$ [M^++Na] 507.0502, found 507.0497.

Enantioselectivity was determined by chiral SFC using Trefoil CEL2 column (20% MeOH, flow rate 2 mL/min): τ_{major} = 5.3 min, τ_{minor} = 8.8 min.

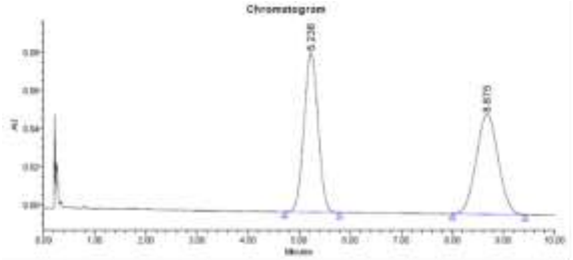

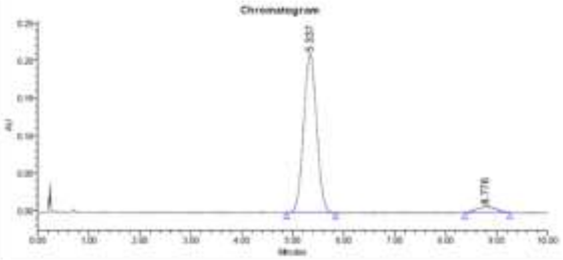

Peak Results

	Retention Time (min)	Relative Area (%)	Area	Height (µV)
1	5.238	50.26	1584925	83416
2	8.675	49.74	1568257	52221

Peak Results

	Retention Time (min)	Relative Area (%)	Area	Height (µV)
1	5.337	94.76	3750381	212088
2	8.776	5.24	207487	7916

(*R*)-2-(7-bromo-1*H*-indol-3-yl)-2-(1-methyl-1*H*-indol-3-yl)-5-phenylfuran-3(2*H*)-one [(*R*)-3Ap]

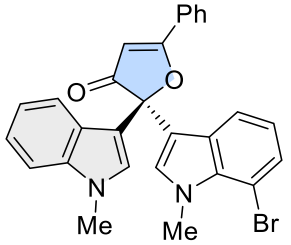

Following the general procedure from **1A** (57.5 mg, 0.2 mmol) and indole **2p** (58.0 mg, 0.2 mmol) for 24 h, compound (*R*)-**3Ap** was obtained as a pale-green solid after purification by column chromatography on silica gel (EtOAc-hexane 1:2) (80.0 mg, 0.16 mmol, 80%, 94:6 er). $[\alpha]_D^{25}$= − 20.1 (*c* 1, DMSO). **^{1}H NMR** (500 MHz, DMSO-d_6): δ 8.04 (d, *J* = 7.5 Hz, 2H), 7.66-7.63 (m, 1H), 7.59-7.56 (m, 2H), 7.51 (d, *J* = 8.0 Hz, 1H), 7.44-7.41 (m, 2H), 7.33-7.31 (m, 2H), 7.22 (s, 1H), 7.14 (t, *J* = 7.6 Hz, 1H), 6.96 (t, *J* = 7.6 Hz, 1H), 6.85 (t, *J* = 7.8 Hz, 1H), 6.62 (s, 1H), 4.09 (s, 3H), 3.74 (s, 3H). **^{13}C NMR** (126 MHz, DMSO-d_6): δ 201.5, 182.7, 137.2, 133.2, 133.1, 131.9, 129.4, 129.2, 129.1, 128.6, 127.2, 126.5, 125.5, 121.5, 120.8, 120.2, 119.9, 119.4, 111.1, 110.6, 110.1, 103.4, 99.0, 88.7, 36.5, 32.4. **HRMS** (ESI) *m/z* calcd. for $C_{28}H_{21}O_2N_2{}^{79}BrNa$ [M$^+$+Na] 519.0679, found 519.0667. *m/z* calcd. for $C_{28}H_{21}O_2N_2{}^{81}BrNa$ [M$^+$+Na] 521.0658, found 521.0646. Enantioselectivity was determined by chiral SFC using Trefoil CEL1 column (20% MeOH, flow rate 2 mL/min): τ_{minor} = 2.5 min, τ_{major} = 3.6 min.

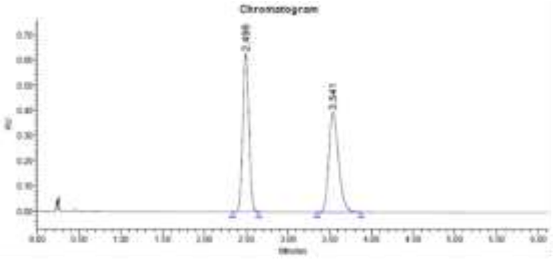

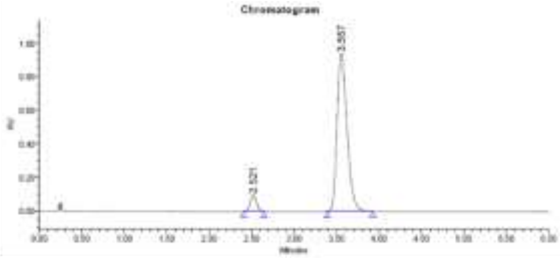

Peak Results

	Retention Time (min)	Relative Area (%)	Area	Height (μV)
1	2.496	50.18	3231657	630615
2	3.541	49.82	3208910	398004

Peak Results

	Retention Time (min)	Relative Area (%)	Area	Height (μV)
1	2.521	6.00	490390	95276
2	3.557	94.00	7676704	941137

(*S*)-2-(7-bromo-1*H*-indol-3-yl)-2-(1-methyl-1*H*-indol-3-yl)-5-phenylfuran-3(2*H*)-one [(*S*)-3Fc]

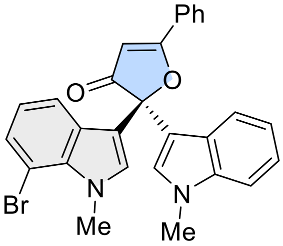

Following the general procedure from **1F** (73.2 mg, 0.2 mmol) and indole **2c** (26 µL, 0.2 mmol) for 48 h, compound (*S*)-**3Fc** was obtained as a yellow solid after purification by column chromatography on silica gel (EtOAc-hexane 1:2) (90.3 mg, 0.18 mmol, 91%, 88:12 er). $[\alpha]_D^{25}$= + 11.2 (*c* 1, DMSO). Spectral data are in accordance with enantiomer (*R*)-**3Ap**.

Enantioselectivity was determined by chiral SFC using Trefoil CEL1 column (20% MeOH, flow rate 2 mL/min): τ_{major} = 2.5 min, τ_{minor} = 3.6 min.

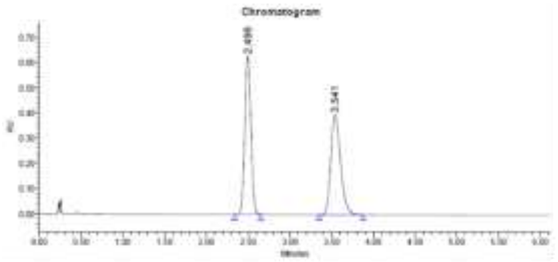

Peak Results

	Retention Time (min)	Relative Area (%)	Area	Height (µV)
1	2.496	50.18	3231657	630615
2	3.541	49.82	3208910	398004

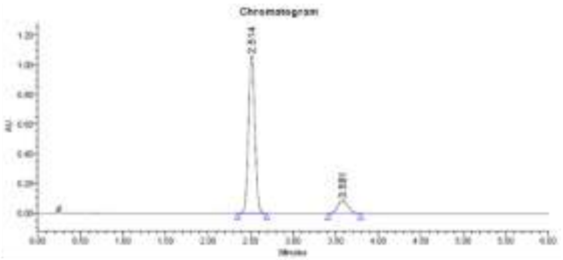

Peak Results

	Retention Time (min)	Relative Area (%)	Area	Height (µV)
1	2.514	88.46	5531884	1064204
2	3.581	11.54	721762	89000

(*R*)-2-(7-chloro-1*H*-indol-3-yl)-2-(1-methyl-1*H*-indol-3-yl)-5-phenylfuran-3(2*H*)-one [(*R*)-3Aq]

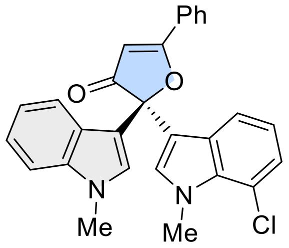

Following the general procedure from **1A** (57.5 mg, 0.2 mmol) and indole **2q** (30.3 mg, 0.2 mmol) for 24 h, compound (*R*)-**3Aq** was obtained as a yellow solid after purification by column chromatography on silica gel (EtOAc-hexane 1:4 → 1:2) (79.2 mg, 0.17 mmol, 87%, 93:7 er). **$[\alpha]_D^{25}$**= − 19.0 (*c* 1, DMSO). **^{1}H NMR** (500 MHz, DMSO-d_6): δ 8.05-8.03 (m, 2H), 7.66-7.63 (m, 1H), 7.58 (t, *J* = 7.5 Hz, 2H), 7.46 (dd, *J* = 8.0, 0.8 Hz, 1H), 7.43 (d, *J* = 8.7 Hz, 2H), 7.33 (s, 1H), 7.22 (s, 1H), 7.16-7.13 (m, 2H), 6.98-6.90 (m, 2H), 6.63 (s, 1H), 4.08 (s, 3H), 3,75 (s, 3H). **^{13}C NMR** (126 MHz, DMSO-d_6): δ 201.6, 182.7, 137.2, 133.1, 132.1, 131.7, 129.2, 129.1, 129.0, 128.6, 127.2, 125.6, 123.0, 121.5, 120.4, 119.9, 119.7, 119.4, 116.2, 111.32, 110.6, 110.1, 99.0, 88.7, 36.4, 32.4. **HRMS** (ESI) *m/z* calcd. for $C_{28}H_{21}O_2N_2ClNa$ [M^++Na] 475.1184, found 475.1178.

Enantioselectivity was determined by chiral SFC using Trefoil CEL1 column (20% MeOH, flow rate 2 mL/min): τ_{minor} = 2.1 min, τ_{major} = 2.6 min.

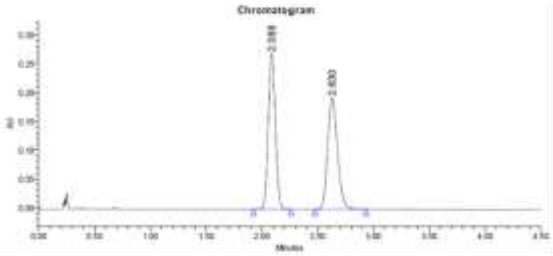

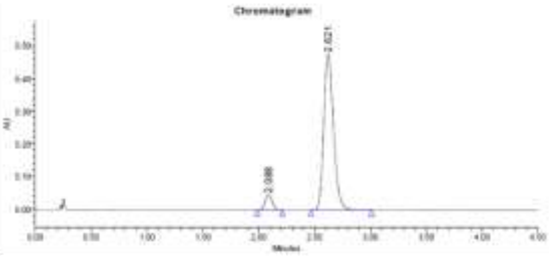

Peak Results

	Retention Time (min)	Relative Area (%)	Area	Height (µV)
1	2.088	50.11	1188995	270772
2	2.630	49.89	1183958	193641

Peak Results

	Retention Time (min)	Relative Area (%)	Area	Height (µV)
1	2.088	6.63	205507	46724
2	2.621	93.37	2894777	478193

(*S*)-2-(7-chloro-1*H*-indol-3-yl)-2-(1-methyl-1*H*-indol-3-yl)-5-phenylfuran-3(2*H*)-one [(*S*)-3Gc]

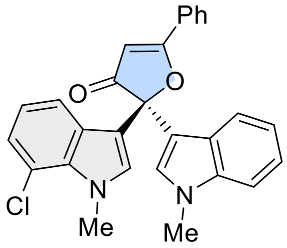

Following the general procedure from **1G** (57.5 mg, 0.2 mmol) and indole **2c** (26 µL, 0.2 mmol) for 24 h, compound (*S*)-**3Gc** was obtained as a yellow solid after purification by column chromatography on silica gel (EtOAc-hexane 1:4 → 1:2) (79.0 mg, 0.17 mmol, 87%, 87:13 er). $[\alpha]_D^{25}$= + 14.1 (*c* 1, DMSO). Spectral data are in accordance with enantiomer (*R*)-**3Aq**.

Enantioselectivity was determined by chiral SFC using Trefoil CEL1 column (20% MeOH, flow rate 2 mL/min): τ_{major} = 2.1 min, τ_{minor} = 2.6 min.

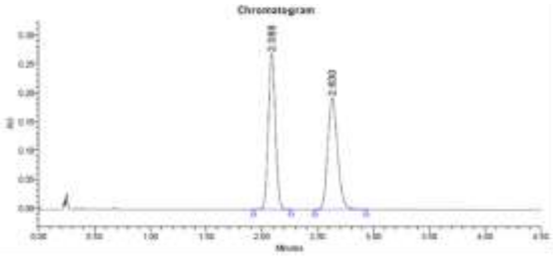

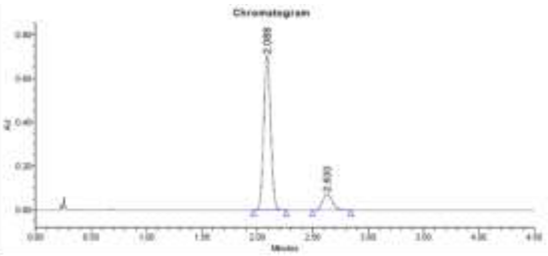

Peak Results

	Retention Time (min)	Relative Area (%)	Area	Height (µV)
1	2.088	50.11	1188995	270772
2	2.630	49.89	1183958	193641

Peak Results

	Retention Time (min)	Relative Area (%)	Area	Height (µV)
1	2.088	87.28	3101601	705593
2	2.630	12.72	452056	73573

(*S*)-2-(1-benzyl-1*H*-indol-3-yl)-2-(7-chloro-1*H*-indol-3-yl)-5-phenylfuran-3(2*H*)-one [(*S*)-3En]

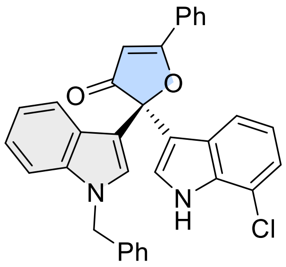

Following the general procedure from **1E** (72.7 mg, 0.2 mmol) and indole **2n** (30.3 mg, 0.2 mmol) for 96 h, compound (*S*)-**3En** was obtained as a pale-yellow solid after purification by column chromatography on silica gel (Hexane → EtOAc-hexane 1:4 → 1:2) (73.5 mg, 0.17 mmol, 71%, 98:2 er). $[\alpha]_D^{25}$= − 13.6 (*c* 1, DMSO). **^{1}H NMR** (500 MHz, DMSO-d_6): δ 11.64 (d, *J* = 2.4 Hz, 1H), 8.08-8.06 (m, 2H), 7.66-7.63 (m, 1H), 7.60-7.56 (m, 2H), 7.48-7.43 (m, 4H), 7.31-7.15 (m, 7H), 7.10-7.07 (m, 1H), 6.97-6.93 (m, 2H), 6.65 (s, 1H), 5.42 (s, 2H). **^{13}C NMR** (126.6 MHz, DMSO-d_6): δ 201.7, 182.8, 138.0, 136.6, 133.6, 133.1, 129.2, 128.7, 128.5, 128.4, 127.3, 127.2, 127.1, 126.9, 125.9, 125.6, 121.7, 121.1, 120.2, 120.1, 119.6, 119.2, 116.2, 113.3, 111.3, 110.7, 99.1, 88.9, 49.0. **HRMS** (ESI) *m/z* calcd. for $C_{33}H_{23}O_2N_2ClNa$ [M^++Na] 537.1340, found 537.1334.

Enantioselectivity was determined by chiral SFC using Trefoil CEL2 column (20% MeOH, flow rate 2 mL/min): τ_{major} = 6.5 min, τ_{minor} = 9.3 min.

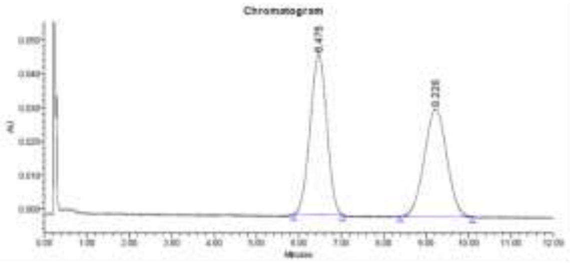

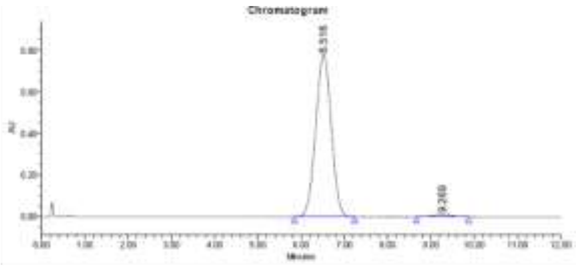

Peak Results

	Retention Time (min)	Relative Area (%)	Area	Height (μV)
1	6.475	52.41	1291368	47392
2	9.226	47.59	1172712	31932

Peak Results

	Retention Time (min)	Relative Area (%)	Area	Height (μV)
1	6.516	98.39	19541631	778373
2	9.269	1.61	319378	9715

(*S*)-2-(7-chloro-1*H*-indol-3-yl)-5-(4-fluorophenyl)-2-(1-methyl-1*H*-indol-3-yl)furan-3(2*H*)-one [(*S*)-3Hn]

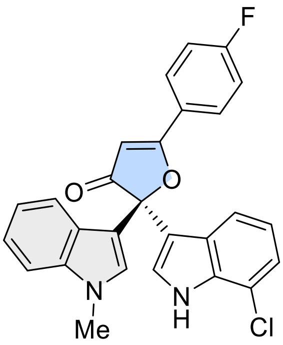

Following the general procedure from **1H** (61.1 mg, 0.2 mmol) and indole **2n** (30.3 mg, 0.2 mmol) for 65 h, compound (*S*)-**3Hn** was obtained as a pale-yellow solid after purification by column chromatography on silica gel (EtOAc-hexane 1:2) (72.3 mg, 0.16 mmol, 79%, 87:13 er). **[α]$_D^{25}$**= − 19.1 (*c* 1, DMSO). **^{1}H NMR** (500 MHz, DMSO-*d$_6$*): δ 11.63 (d, *J* = 2.3 Hz, 1H), 8.15-8.11 (m, 2H), 7.45-7.41 (m, 5H), 7.25-7.23 (m, 2H), 7.19-7.13 (m, 2H), 6.98-6.94 (m, 2H), 6.63 (s, 1H), 3.75 (s, 3H). **^{13}C NMR** (126.6 MHz, DMSO-*d$_6$*): δ 201.6, 181.7, 164.8 (d, $J_{C,F}$ = 252.2 Hz), 137.2, 133.5, 130 (d, $J_{C,F}$ = 9.3 Hz), 129.1, 127.2, 125.8, 125.6, 125.2 (d, $J_{C,F}$ = 2.7 Hz), 121.5, 121.1, 120.3, 120.0, 119.4, 119.1, 116.5 (d, $J_{C,F}$ = 22.1 Hz), 116.2, 113.2, 110.7, 110.2, 99.0, 89.1, 32.5. **^{19}F NMR** (471 MHz, DMSO-*d$_6$*): δ −105.6. **HRMS** (ESI) *m/z* calcd. for $C_{27}H_{18}O_2N_2ClFNa$ [M$^+$+Na] 479.0933, found 479.0928.

Enantioselectivity was determined by chiral SFC using Trefoil CEL1 column (15% MeOH, flow rate 2 mL/min): τ_{minor} = 4.2 min, τ_{major} = 4.7 min.

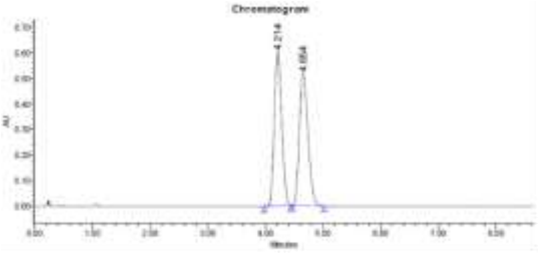

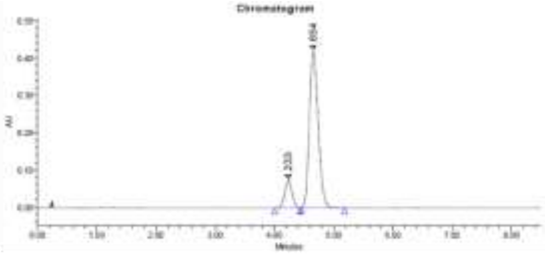

Peak Results

	Retention Time (min)	Relative Area (%)	Area	Height (μV)
1	4.214	50.11	5368392	603139
2	4.654	49.89	5344883	516598

Peak Results

	Retention Time (min)	Relative Area (%)	Area	Height (μV)
1	4.233	12.73	628236	72152
2	4.654	87.27	4307631	418078

(*S*)-5-(3,5-bis(trifluoromethyl)phenyl)-2-(7-chloro-1*H*-indol-3-yl)-2-(1-methyl-1*H*-indol-3-yl) furan-3(2*H*)-one [(*S*)-3In]

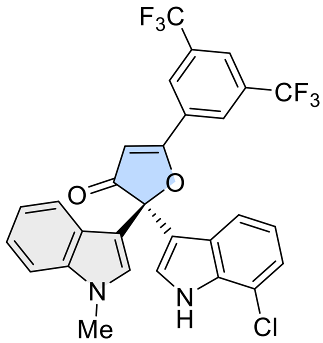

Following the general procedure from **1l** (84.7 mg, 0.2 mmol) and indole **2n** (30.3 mg, 0.2 mmol) for 96 h, compound (*S*)-**3ln** was obtained as a greenish solid after purification by column chromatography on silica gel (Hexane → EtOAc-hexane 1:4 → 1:2) (95.3 mg, 0.17 mmol, 83%, 96:4 er). $[\alpha]_D^{25}$= − 27.9 (*c* 1, DMSO). **^{1}H NMR** (500 MHz, DMSO-d_6): δ 11.68 (s, 1H), 8.64 (s, 2H), 8.40 (s, 1H), 7.45-7.40 (m, 3H), 7.33-7.31 (m, 2H), 7.19-7.13 (m, 2H), 7.08 (s, 1H), 6.98-6.93 (m, 2H), 3.76 (s, 3H). **^{13}C NMR** (126 MHz, DMSO-d_6): δ 201.9, 179.0, 137.2, 133.6, 131.3 (q, $J_{C,F}$ = 33.6 Hz), 131.1, 129.4, 127.6-127.5 (m), 127.2, 126.2 (br s), 125.5, 122.9 (q, $J_{C,F}$ = 273.0 Hz), 121.6, 121.1, 120.4, 119.9, 119.5, 119.0, 116.2, 112.6, 110.2, 110.0, 101.7, 89.7, 32.5. **^{19}F NMR** (471 MHz, DMSO-d_6): δ −61.3 (s, 6F). **HRMS** (ESI) *m/z* calcd. for $C_{29}H_{17}O_2N_2ClF_6Na$ [M^++Na] 597.0775, found 597.0772.

Enantioselectivity was determined by chiral SFC using Trefoil CEL1 column (15% MeOH, flow rate 2 mL/min): τ_{minor} = 1.0 min, τ_{major} = 1.1 min.

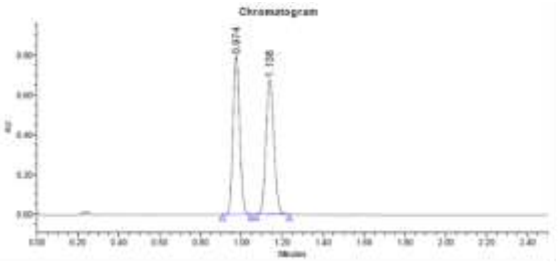

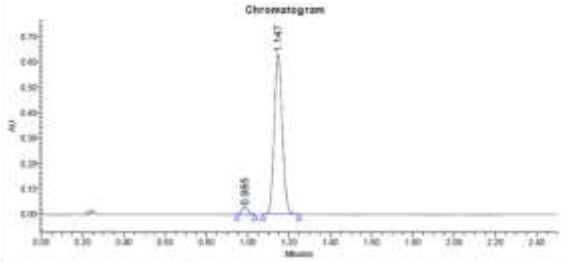

Peak Results

	Retention Time (min)	Relative Area (%)	Area	Height (µV)
1	0.974	49.62	1805117	797088
2	1.138	50.38	1832724	678591

Peak Results

	Retention Time (min)	Relative Area (%)	Area	Height (µV)
1	0.985	3.63	63999	29223
2	1.147	96.37	1700522	630915

(*S*)-2-(7-chloro-1*H*-indol-3-yl)-2-(1-methyl-1*H*-pyrrol-2-yl)-5-phenylfuran-3(2*H*)-one [(*S*)-3Bn]

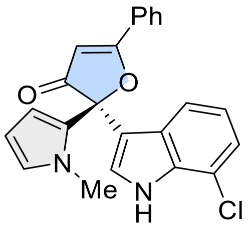

Following the general procedure from **1B** (47.5 mg, 0.2 mmol) and indole **2n** (30.3 mg, 0.2 mmol) for 24 h compound (*S*)-**3Bn** was obtained as a pale-yellow solid after purification by column chromatography on silica gel (Hexane → EtOAc-hexane 1:2) (40.0 mg, 0.10 mmol, 51%, 85:15 er). $[\alpha]_D^{25}$= − 211.8 (*c* 1, DMSO). **^{1}H NMR** (500 MHz, DMSO-d_6): δ 11.69 (d, *J* = 1.7 Hz, 1H), 8.08-8.06 (m, 2H), 7.69-7.66 (m, 1H), 7.60 (t, *J* = 7.6 Hz, 2H), 7.19 (dd, *J* = 7.6, 0.5 Hz, 1H), 7.14-7.12 (m, 2H), 6.93 (t, *J* = 7.9 Hz, 1H), 6.85-6.84 (m, 1H), 6.60 (s, 1H), 6.09 (dd, *J* = 3.7, 1.9 Hz, 1H), 5.99 (dd, *J* = 3.7, 2.7 Hz, 1H), 3.47 (s, 3H). **^{13}C NMR** (126 MHz, DMSO-d_6): δ 200.2, 182.3, 133.5, 133.2, 129.2, 128.3, 127.2, 126.7, 126.6, 126.0, 125.0, 121.1, 120.4, 118.6, 116.2, 112.6, 110.3, 106.1, 99.1, 87.4, 35.0. **HRMS** (ESI) *m/z* calcd. for $C_{23}H_{17}O_2N_2ClNa$ [M^++Na] 411.0871, found 411.0865.

Enantioselectivity was determined by chiral SFC using Trefoil CEL1 column (20% MeOH, flow rate 2 mL/min): τ_{major} = 1.0 min, τ_{minor} = 1.2 min.

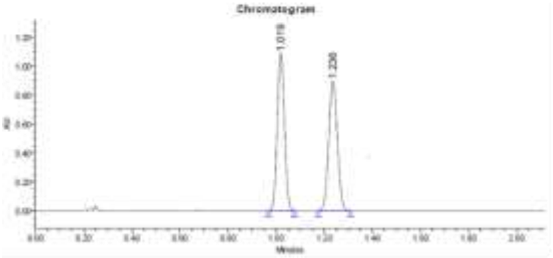

Peak Results

	Retention Time (min)	Relative Area (%)	Area	Height (µV)
1	1.019	50.01	2270801	1092703
2	1.236	49.99	2270006	900236

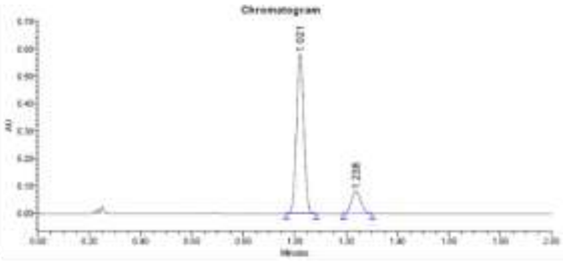

Peak Results

	Retention Time (min)	Relative Area (%)	Area	Height (µV)
1	1.021	85.07	1163520	581811
2	1.238	14.93	204211	81183

(*S*)-2-(2-methoxythiophen-5-yl)-2-(1-methyl-1*H*-indol-3-yl)-5-phenylfuran-3(2*H*)-one [(*S*)-3Ar]

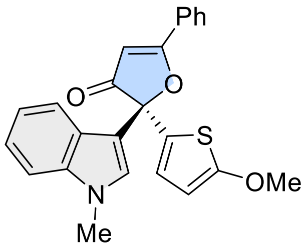

Following the general procedure from **1A** (57.5 mg, 0.2 mmol) and **2r** (22.8 mg, 0.2 mmol) for 36 h, compound (*S*)-**3Ar** was obtained as a yellowish solid after purification by column chromatography on silica gel (Hexane → EtOAc-hexane 1:5 → 1:2) (70.8 mg, 0.18 mmol, 88%, 80:20 er). $[\alpha]_D^{25}$= – 84.0 (*c* 1, DMSO). Spectroscopic data were consistent with literature values.[18] ^{1}**H NMR** (300 MHz, DMSO-d_6): δ 8.04-8.01 (m, 2H), 7.67-7.56 (m, 3H), 7.48-7.43 (m, 2H), 7.36 (s, 1H), 7.20-7.15 (m, 1H), 7.05-6.99 (m, 1H), 6.71 (d, *J* = 4.0 Hz, 1H), 6.58 (s, 1H), 6.20 (d, *J* = 4.0 Hz, 1H), 3.83 (s, 3H), 3.78 (s, 3H). ^{13}**C NMR** (75.5 MHz, DMSO-d_6): δ 200.2, 182.9, 166.1, 137.1, 133.3, 129.2, 128.2, 127.2, 126.0, 125.5, 124.1, 121.7, 120.0, 119.6, 110.3, 110.2, 103.4, 98.6, 88.7, 60.1, 32.5.

Enantioselectivity was determined by chiral HPLC analysis using Chiralpak-IA column (hexane/propan-2-ol 80:20, flow rate 1 mL/min): τ_{minor} = 11.5 min, τ_{major} = 18.9 min.

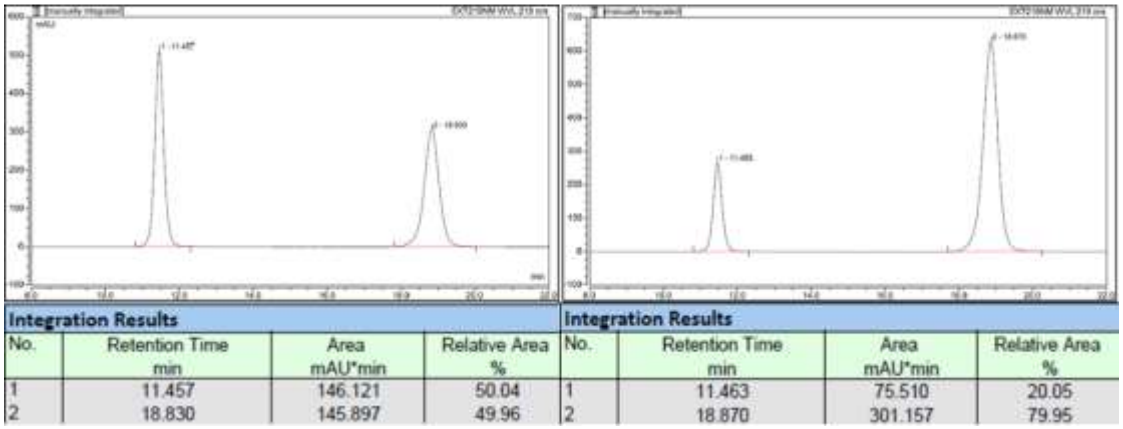

Integration Results

No.	Retention Time min	Area mAU*min	Relative Area %
1	11.457	146.121	50.04
2	18.830	145.897	49.96

Integration Results

No.	Retention Time min	Area mAU*min	Relative Area %
1	11.463	75.510	20.05
2	18.870	301.157	79.95

7. Large-scale reactions

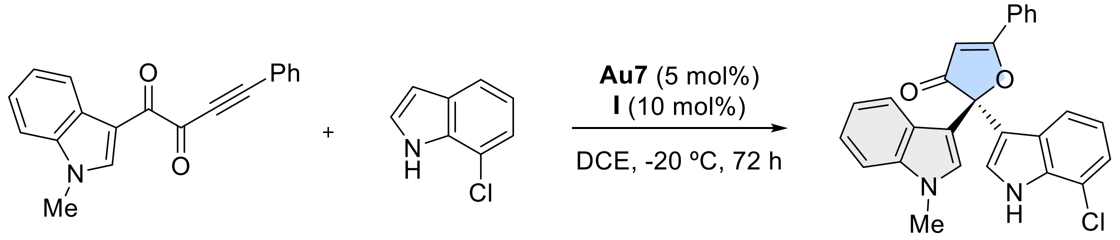

1A **2n** (*S*)-**3An**

Reaction performed at 1.0 mmol scale: In a flame-dried Schlenk flask, a solution of substrate **1A** (287 mg, 1.0 mmol) and the corresponding indole **2n** (152 mg, 1.0 mmol, 1.0 equiv) in anhydrous 1,2-DCE (3.0 mL, 0.33 M) was cooled at –20 ºC for 10 mins. Then, **Au7** (60 mg, 0.05 mmol, 5 mol%) and **I** (60 mg, 0.1 mmol, 10 mol%) were added. The reaction mixture was stirred at –20 ºC for 72 h. Then, the resulting suspension was directly filtered off under vacuum and the vessel was washed with cold Et_2O (20 mL) and cold MeOH (10 mL).* The remaining organic phase was concentrated under vacuum and then purified by column chromatography on silica gel (EtOAc-hexane 1:2 → 1:1) to afford pure product (*S*)-**3An** as a yellow solid (242 mg, 0.55 mmol, 55%, >99:1 er). $[\alpha]_D^{25}$= + 5.8 (*c* 1, DMSO). The corresponding gold complex **Au7** (46.8 mg, 0.04 mmol, 78%) and activator **I** (52.0 mg, 0.09 mmol, 87%) were also recovered during purification. Enantioselectivity was determined by chiral SFC using Trefoil CEL2 column (20% MeOH, flow rate 2 mL/min): τ_{major} = 4.3 min, τ_{minor} = 7.1 min.

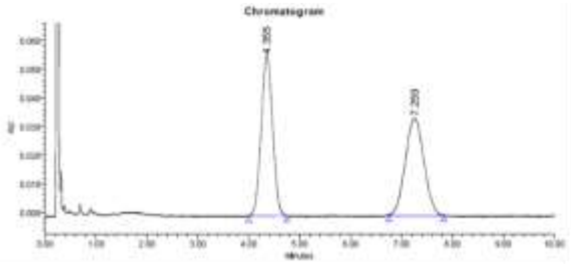

Peak Results

	Retention Time (min)	Relative Area (%)	Area	Height (μV)
1	4.355	50.15	873438	55950
2	7.259	49.85	868054	34166

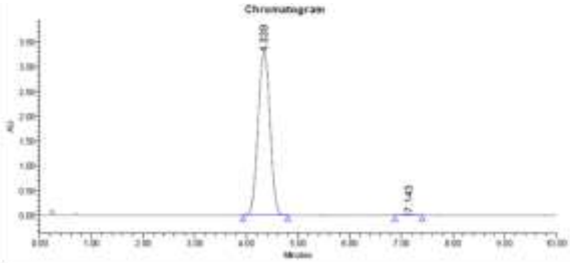

Peak Results

	Retention Time (min)	Relative Area (%)	Area	Height (μV)
1	4.339	99.44	52880801	3252088
2	7.143	0.56	296673	16255

*The white precipitate isolated during filtration was analyzed by chiral SFC using Trefoil CEL2 column (20% MeOH, flow rate 2 mL/min) showing a racemic mixture of compound **3An** (50.0 mg, 0.11 mmol, 11%).

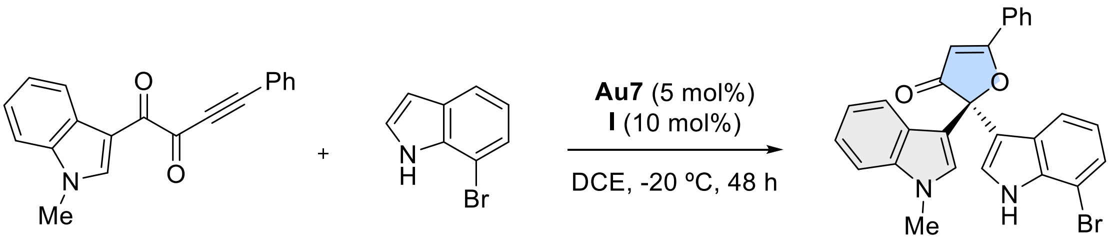

1A **2o** (S)-**3Ao**

Reaction performed at 1.0 mmol scale: In a flame-dried Schlenk flask, a solution of substrate **1A** (287 mg, 1.0 mmol) and the corresponding indole **2o** (200 mg, 1.0 mmol, 1.0 equiv) in anhydrous 1,2-DCE (3.0 mL, 0.33 M) was cooled at –20 ºC for 10 mins. Then, **Au7** (60 mg, 0.05 mmol, 5 mol%) and **I** (60 mg, 0.1 mmol, 10 mol%) were added. The reaction mixture was stirred at –20 ºC for 48 h. Then, the resulting suspension was directly filtered off under vacuum and the vessel was washed with cold Et_2O (20 mL) and cold MeOH (10 mL).* The remaining organic phase was concentrated under vacuum and then purified by column chromatography on silica gel (EtOAc-hexane 1:3 → 1:2) to afford pure product (S)-**3Ao** as a yellowish solid (256 mg, 0.53 mmol, 53%, >99:1 er). $[\alpha]_D^{25}$= – 15.7 (c 1, DMSO). The corresponding gold complex **Au7** (45.6 mg, 0.04 mmol, 76%) and activator **I** (51.4 mg, 0.09 mmol, 86%) were also recovered during purification.

Enantioselectivity was determined by chiral SFC using Trefoil CEL2 column (20% MeOH, flow rate 2 mL/min): τ_{major} = 5.3 min, τ_{minor} = 8.8 min.

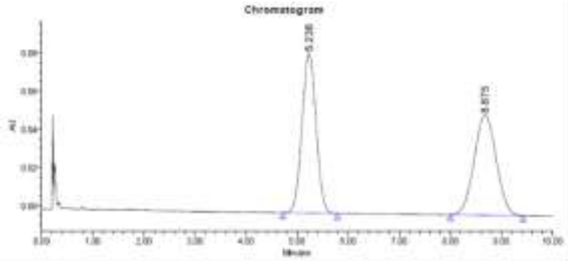

Peak Results

	Retention Time (min)	Relative Area (%)	Area	Height (µV)
1	5.236	50.26	1584925	83416
2	8.675	49.74	1568257	52221

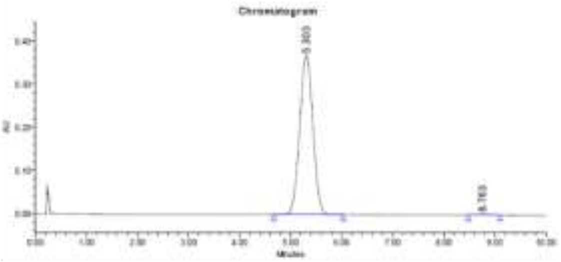

Peak Results

	Retention Time (min)	Relative Area (%)	Area	Height (µV)
1	5.303	99.57	6633996	370031
2	8.763	0.43	28568	1443

*The white precipitate isolated during filtration was analyzed by chiral SFC using Trefoil CEL2 column (20% MeOH, flow rate 2 mL/min) showing a racemic mixture of compound **3Ao** (70.9 mg, 0.15 mmol, 15%).

8. Catalyst recycling

The recovered chiral gold(I) complex **Au7** and the activator **I** were used in successive catalytic cycles without appreciable loss of efficiency.

General procedure for successive catalytic cycles: In a flame-dried Schlenk flask, a solution of substrate **1A** (0.2-1.0 mmol, 1.0 equiv) and the corresponding indole **2n** (0.2-1.0 mmol, 1.0 equiv) in anhydrous 1,2-DCE (0.6-3.0 mL, 0.33 M) was cooled at –20 ºC for 10 mins. Then, complex **Au7** (0.01-0.05 mmol, 5 mol%) and activator **I** (0.02-0.1 mmol, 10 mol%) were added. The reaction mixture was stirred at –20 ºC until consumption of starting material (TLC monitoring). Then, the resulting suspension was directly filtered off under vacuum and the vessel was washed with cold Et_2O (20 mL) and cold MeOH (10 mL). The remaining organic phase was concentrated under vacuum and then purified by column chromatography on silica gel (EtOAc-hexane 1:3 → 1:2) to afford pure product (*S*)-**3An** as a yellowish solid. The corresponding gold complex **Au7** and activator **I** were recovered during successive purifications and reused in the following catalytic cycle.

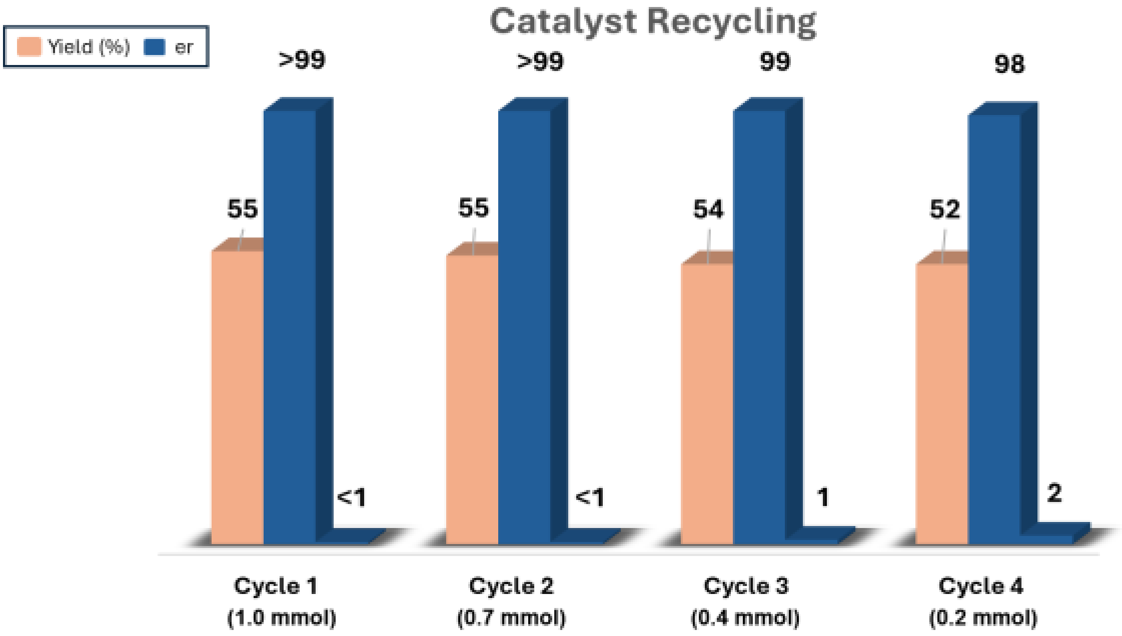

9. Crystal data

Crystallization of (*S*)-**3An** by slow diffusion of pentane into a solution of (*S*)-**3An** in dichloromethane afforded suitable crystals for X-ray analysis. Mp = 156-158 ºC

For crystallography, suitable-sized crystals were coated with FOMBLIN oil and mounted on a glass fiber. Data collection was performed at 100 K using a Bruker SMART APEX II CCD area detector on a D8 goniometer. Mo Kα1 radiation (λ = 0.71073 Å) from a graphite monochromator was utilized. A Bruker Cryo-Flex low-temperature device was used to maintain the temperature. Data collection was processed with APEX-W2D-NT, cell refinement and data reduction with SAINT-Plus1, and absorption correction using the multiscan method applied by SADABS. The crystal structure was solved by direct methods and refined based on F2 (SHELXTL). Non-hydrogen atoms had anisotropic displacement parameters refined, and hydrogen atoms attached to these atoms were placed in geometrically idealized positions and refined using a riding model.

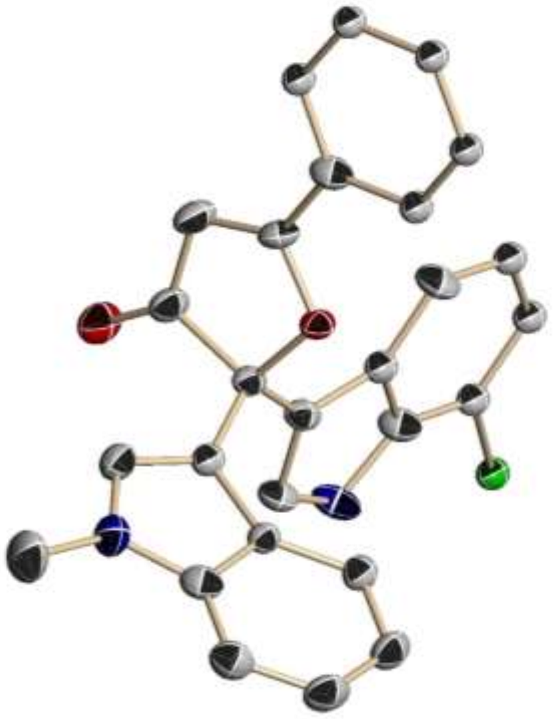

Figure 1. ORTEP plot of (*S*)-**3An** with thermal ellipsoids set at the 50% probability level. Hydrogen atoms are omitted for clarity.

Table 2. Crystal data and structure refinement for (*S*)-**3An**:

Empirical formula	$C_{55}H_{37}Cl_4N_4O_4$
Formula weight	959.741
Temperature/K	193.00
Crystal system	monoclinic
Space group	$P2_1$
a/Å	9.9535(3)
b/Å	23.1178(8)
c/Å	10.9022(4)
α/°	90
β/°	106.984(1)
γ/°	90
Volume/Å^3	2399.22(14)
Z	2
ρ_{calc}g/cm^3	1.329
μ/mm^{-1}	0.298
F(000)	991.7
Crystal size/mm^3	0.2 × 0.12 × 0.08
Radiation	Mo Kα (λ = 0.71073)
2Θ range for data collection/°	3.9 to 56.66
Index ranges	$-13 \leq h \leq 12$, $-30 \leq k \leq 30$, $-14 \leq l \leq 14$
Reflections collected	51681
Independent reflections	11957 [R_{int} = 0.0691, R_{sigma} = 0.0530]
Data/restraints/parameters	11957/64/634
Goodness-of-fit on F^2	1.099
Final R indexes [I>=2σ (I)]	R_1 = 0.0520, wR_2 = 0.1281
Final R indexes [all data]	R_1 = 0.0604, wR_2 = 0.1338
Largest diff. peak/hole / e Å^{-3}	0.44/-0.45
Flack parameter	0.09(3)

10. References

[1] Substrates **1A-C** and **1H**: E. Merkul, J. Dohe, C. Gers, F. Rominger, T. J. J. Müller, *Angew. Chem. Int. Ed.* **2011**, *50*, 2966–2969. Substrate **1D**: X. Kong, G. Zhang, S. Yang, X. Liu, X. Fang, *Adv. Synth. Catal.* **2017**, *359*, 2729–2734.

[2] P. Elías-Rodríguez, E. Matador, M. Benítez, T. Tejero, E. Díez, R. Fernández, P. Merino, D. Monge, J. M. Lassaletta, *J. Org. Chem*. **2023**, *88*, 2487–2492.

[3] F. Pessagno, M. Blair, M. H. Muldoon, P. Manesiotis, *ACS Appl. Polym. Mater.* **2022**, *4*, 7770–7777.

[4] Indole **2b**: J. M. Fraile, K. Le Jeune, J. A. Mayoral, N. Ravasiob, F. Zaccheria, *Org. Biomol. Chem.* **2013**, *11*, 4327–4332. Indole **2e**: K. Nemoto, S. Tanaka, M. Konno, S. Onozawa, M. Chiba, Y. Tanaka, Y. Sasaki, R. Okubo, T. Hattori, *Tetrahedron* **2016**, *72*, 734–745. Indole **2p**: G. L. Tolnai, S. Ganss, J. P. Brand, J. Waser, *Org. Lett.* **2013**, *15*, 112-115. Indole **2q**: W. J.; Burgess, D. Jakas, W.F Huffman, W.H. Miller, K.A. Newlander, M.A. Seefeld, I.N. Uzinskas, *World Intellectual Property Organization* WO 03/088897, **2003**.

[5] Amine **R1**: M. Kinsella, P. G. Duggan, J. Muldoon, K. S. Eccles, S. E. Lawrence, C. M. Lennon, *Eur. J. Org.* **2011**, 1125–1132. Amine **R2**: W. Wang, Y. Guo, K. Sun, S. Wang, S. Zhang, C. Liu, Q.-Y. Chen, *J. Org. Chem.* **2018**, *83*, 14588–14599. Amine **R3**: Z. Zuo, J. Liu, J. Nan, L. Fan, W. Sun, Y. Wang, X. Luan, *Angew. Chem. Int. Ed.* **2015**, *54*, 15385–15389. Amine **R4**: D. Rivillo, H. Gulyás, J. Benet-Buchholz, E. C. Escudero-Adán, Z. Freixa, P. W. N. M. van Leeuwen, *Angew. Chem. Int. Ed.* **2007**, *46*, 7247–7250.

[6] M. P. Muñoz, J. Adrio, J. C. Carretero, A. M. Echavarren, *Organometallics* **2005**, *24*, 1293–1300.

[7] Gold(I) complexes **Au2** and ent-**Au13**: S. Handa, D. J. Lippincott, D. H. Aue, B. H. Lipshutz, *Angew. Chem. Int. Ed.* **2014**, *53*, 10658–10662.

[8] A. D. Melhado, M. Luparia, F. D. Toste, *J. Am. Chem. Soc.* **2007**, *129*, 12638–12639.

[9] J.-K. Yu, C. Czekelius, *Eur. J. Org. Chem.* **2022**, e202200027.

[10] I. Alonso, B. Trillo, F. López, S. Montserrat, G. Ujaque, L. Castedo, A. Lledós, J. L. Mascareñas, *J. Am. Chem. Soc.* **2009**, *131*, 13020–13030.

[11] M.-Z. Wang, C.-Y. Zhou, Z. Guo, E. L.-M. Wong, M.-K. Wong, C.-M. Che, *Chem. Asian J.* **2011**, *6*, 812–824.

[12] R. J. Felix, D. Weber, O. Gutierrez, D. J. Tantillo, M. R. Gagné, *Nat. Chem.* **2012**, *4*, 405–409.

[13] C. García-Morales, B. Ranieri, I. Escofet, L. López-Suarez, C. Obradors, A. I. Konovalov, A. M. Echavarren, *J. Am. Chem. Soc.* **2017**, *139*, 13628–13631.

M.J. Johansson, D.J. Gorin, S.T. Staben, F.D. Toste, *J. Am. Chem. Soc.*, **2005**, *127*, 18002-18003.

[14] M.J. Johansson, D.J. Gorin, S.T. Staben, F.D. Toste, *J. Am. Chem. Soc.,* **2005**, *127*, 18002-18003.

[15] J. Himmelstrup, M. B. Buendia, X. Sun, S. Kramer, *Chem. Commun.* **2019**, *55*, 12988–12991.

[16] M. Goto, M. Yamano (Takeda Chemical Industries), *European Patent Office* WO 2003/048174, **2003**.
[17] A. Kornicka, Z. Brzozowski, F.Sączewski, *Green Chem.* **2006**, *8*, 647-656.
[18] D. Bag, S. D. Sawant, *Org. Lett.* **2022**, 2*4*, 4930–4934.

11. NMR spectra of compounds

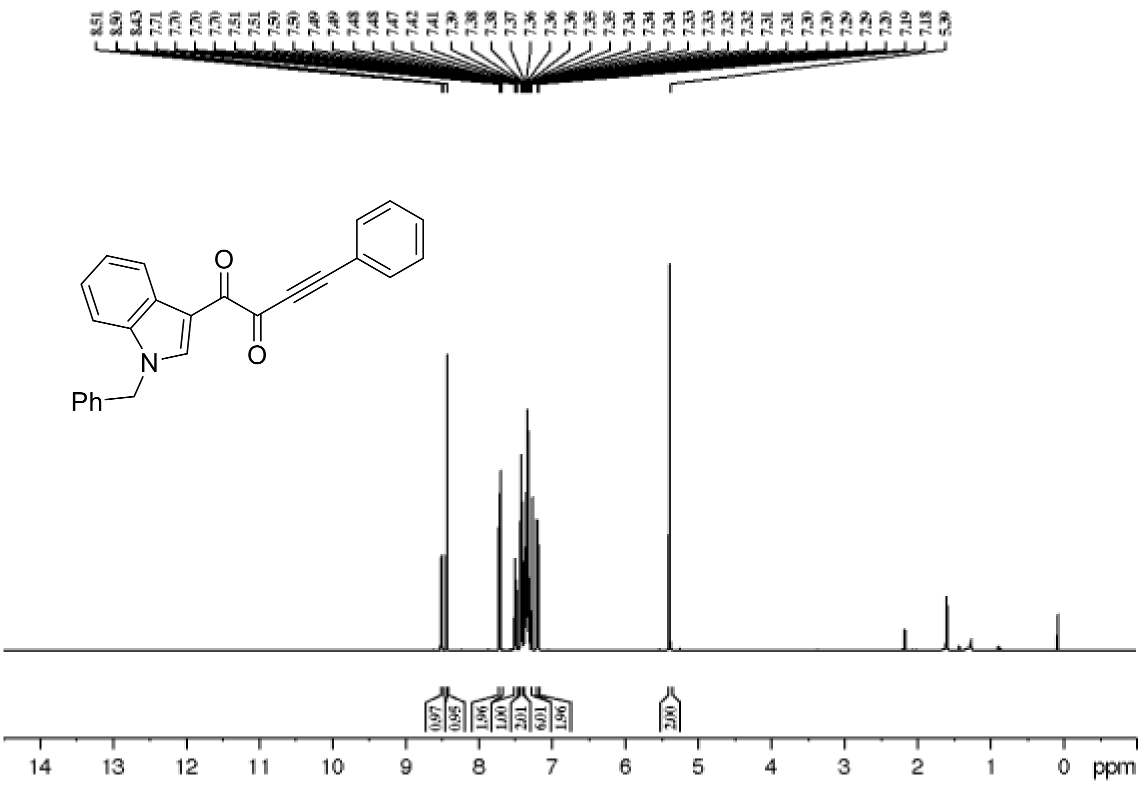

^{1}H NMR (500 MHz, $CDCl_3$) of **1E**

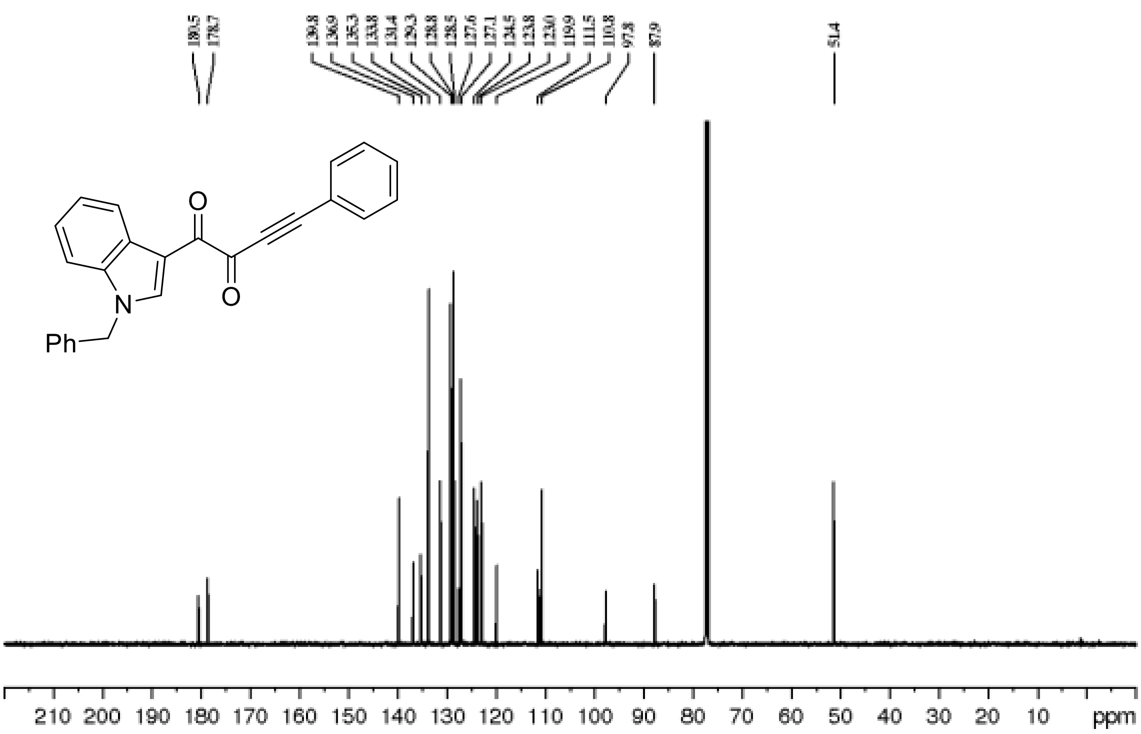

^{13}C NMR (126 MHz, $CDCl_3$) of **1E**

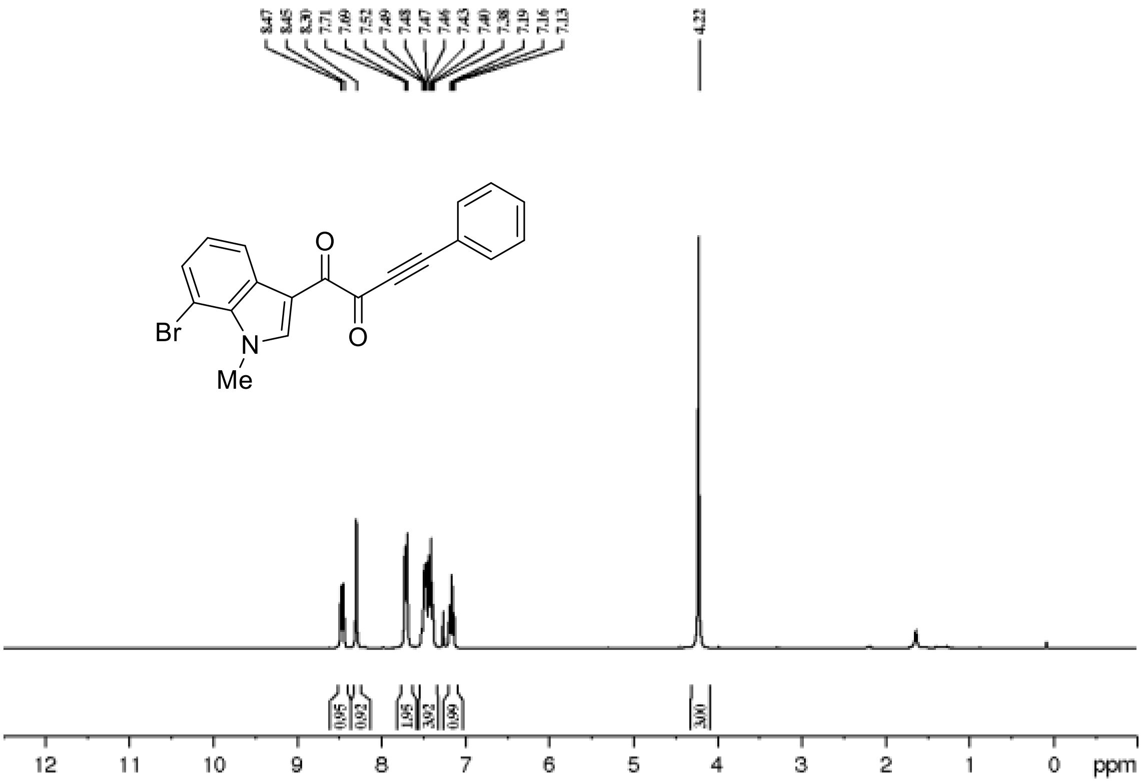

^{1}H NMR (300 MHz, $CDCl_3$) of **1F**

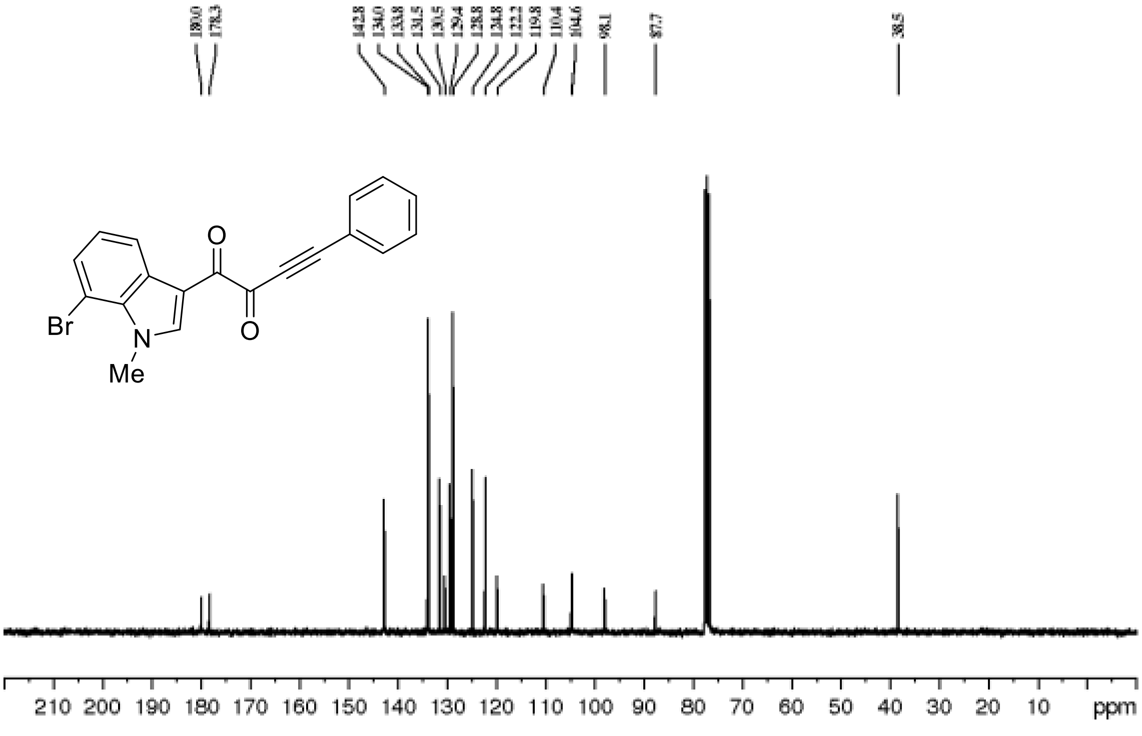

^{13}C NMR (75.5 MHz, $CDCl_3$) of **1F**

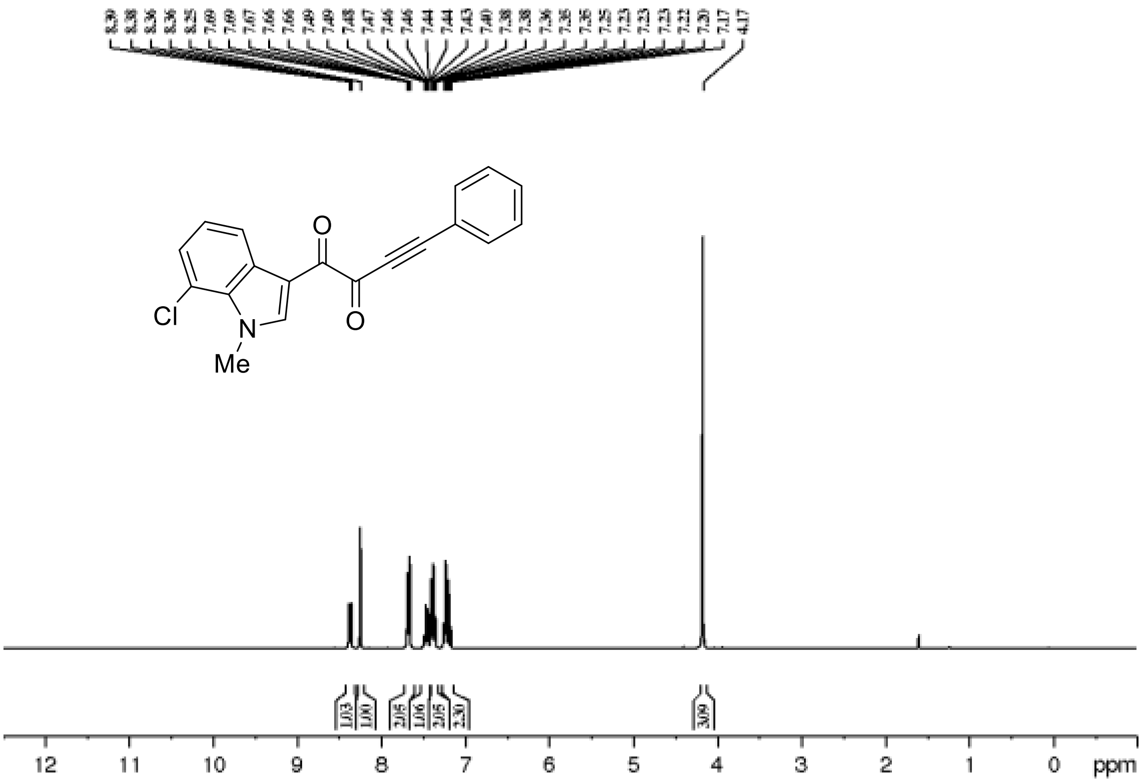

^{1}H NMR (300 MHz, $CDCl_3$) of **1G**

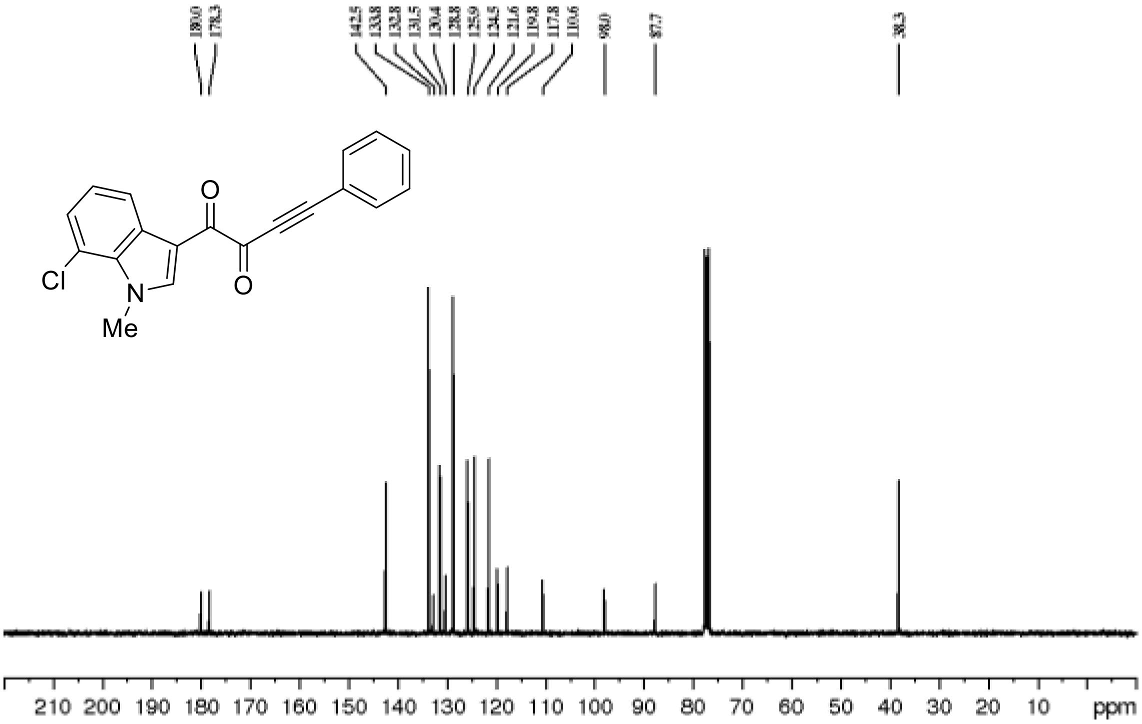

^{13}C NMR (75.5 MHz, $CDCl_3$) of **1G**

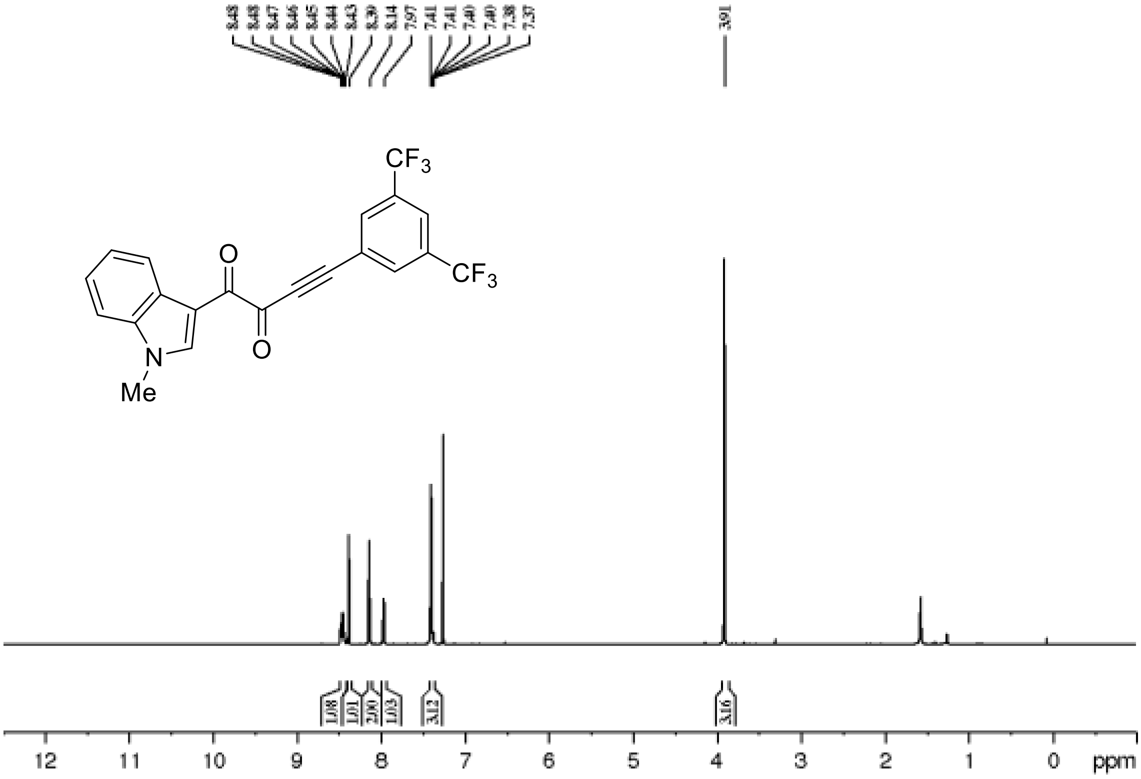

1H NMR (300 MHz, $CDCl_3$) of **1I**

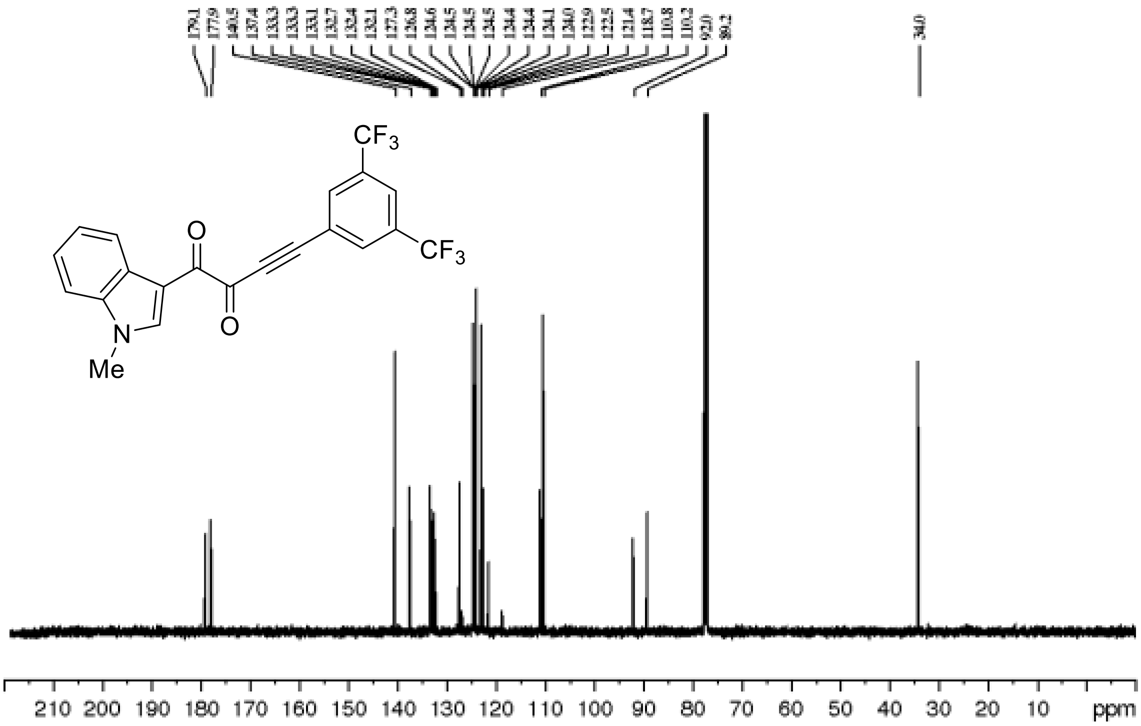

13C NMR (75.5 MHz, $CDCl_3$) of **1I**

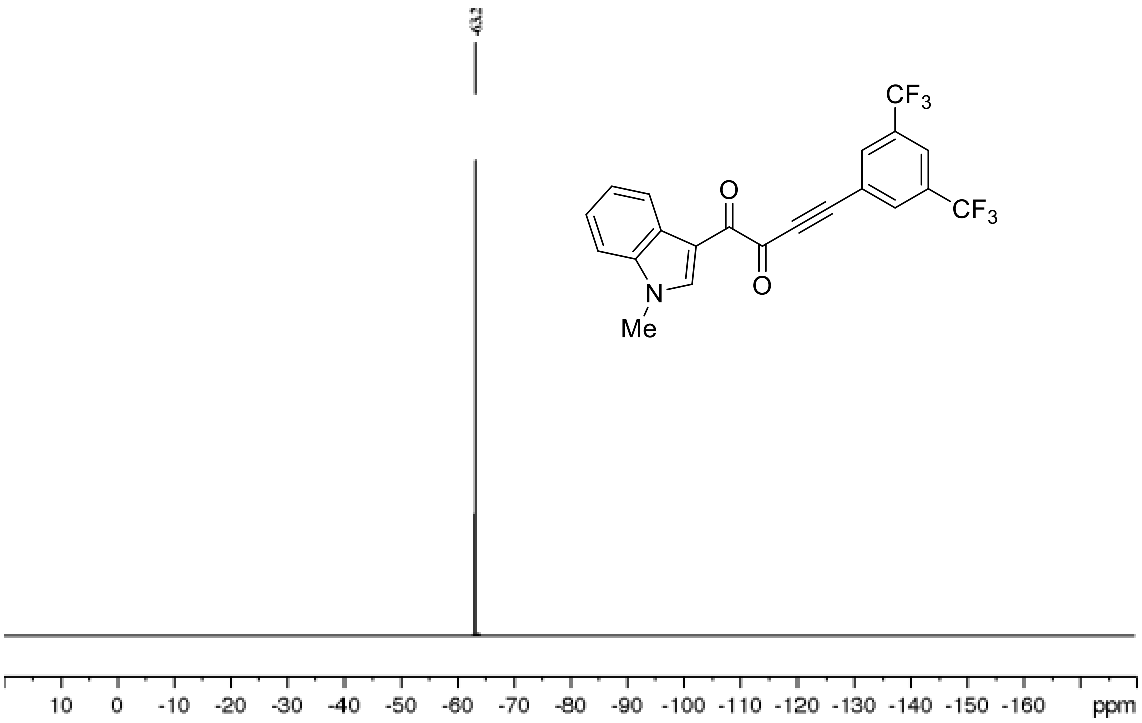

^{19}F NMR (282 MHz, $CDCl_6$) of **1I**

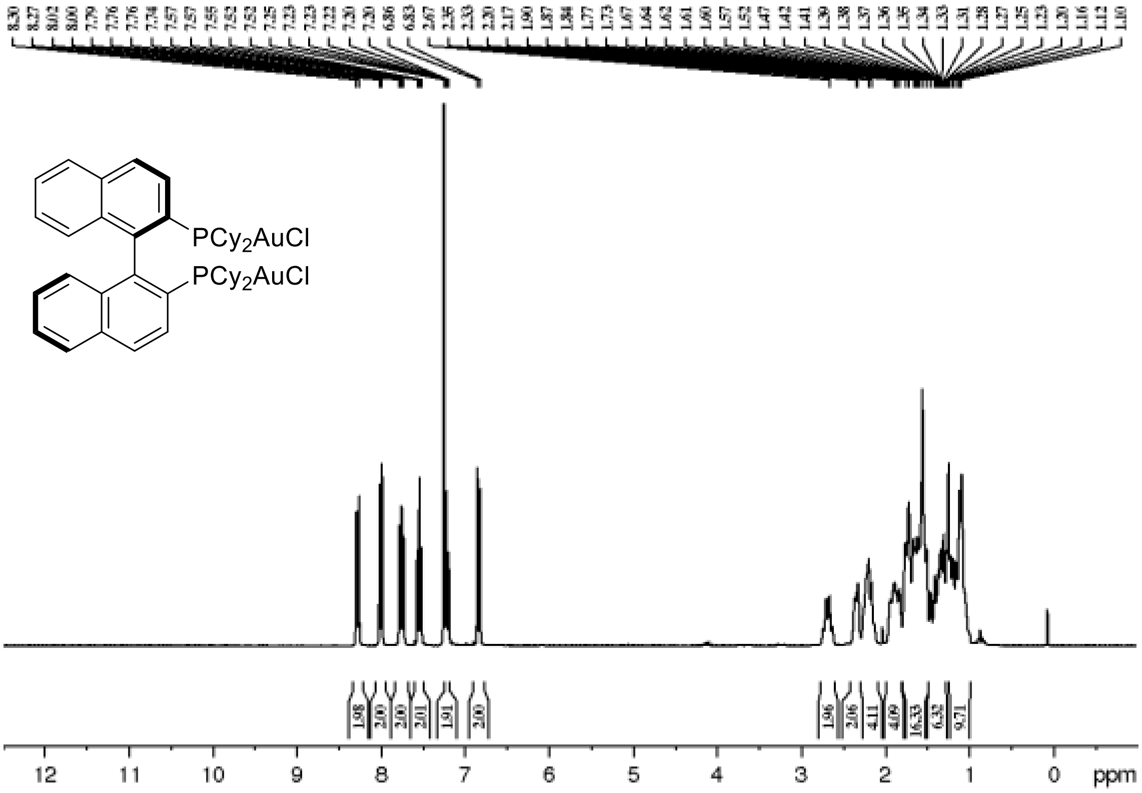

^{1}H NMR (300 MHz, $CDCl_3$) of **Au3**

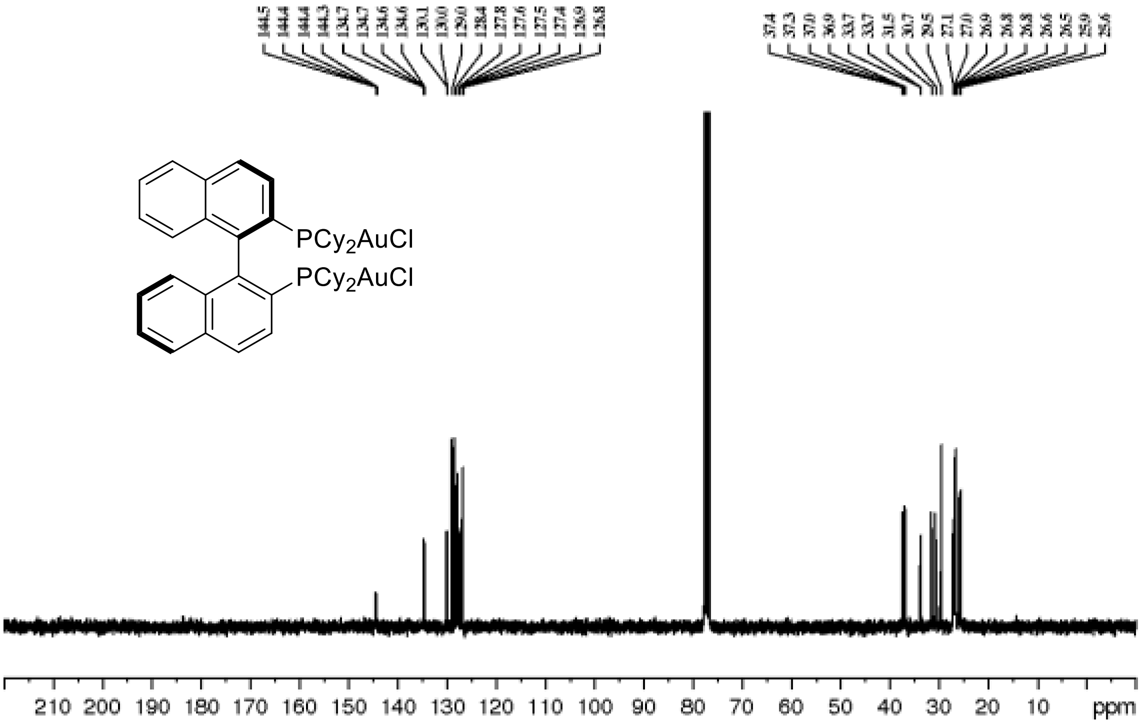

^{13}**C NMR** (75.5 MHz, $CDCl_3$) of **Au3**

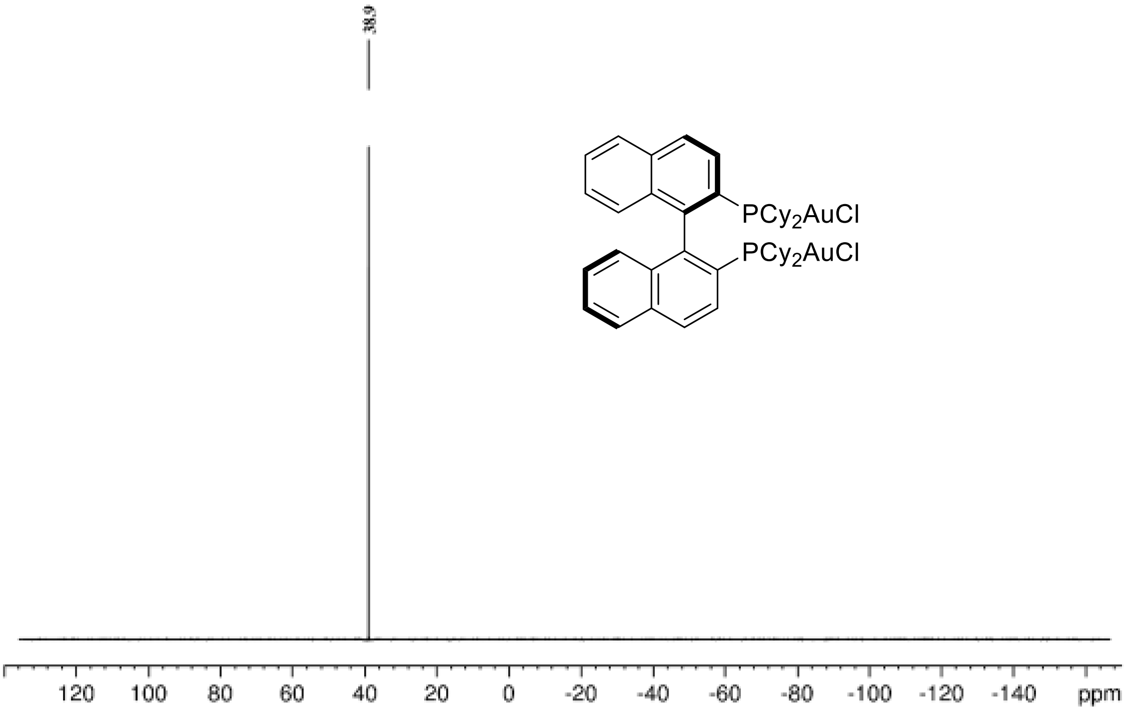

^{31}**P NMR** (121.5 MHz, $CDCl_3$) of **Au3**

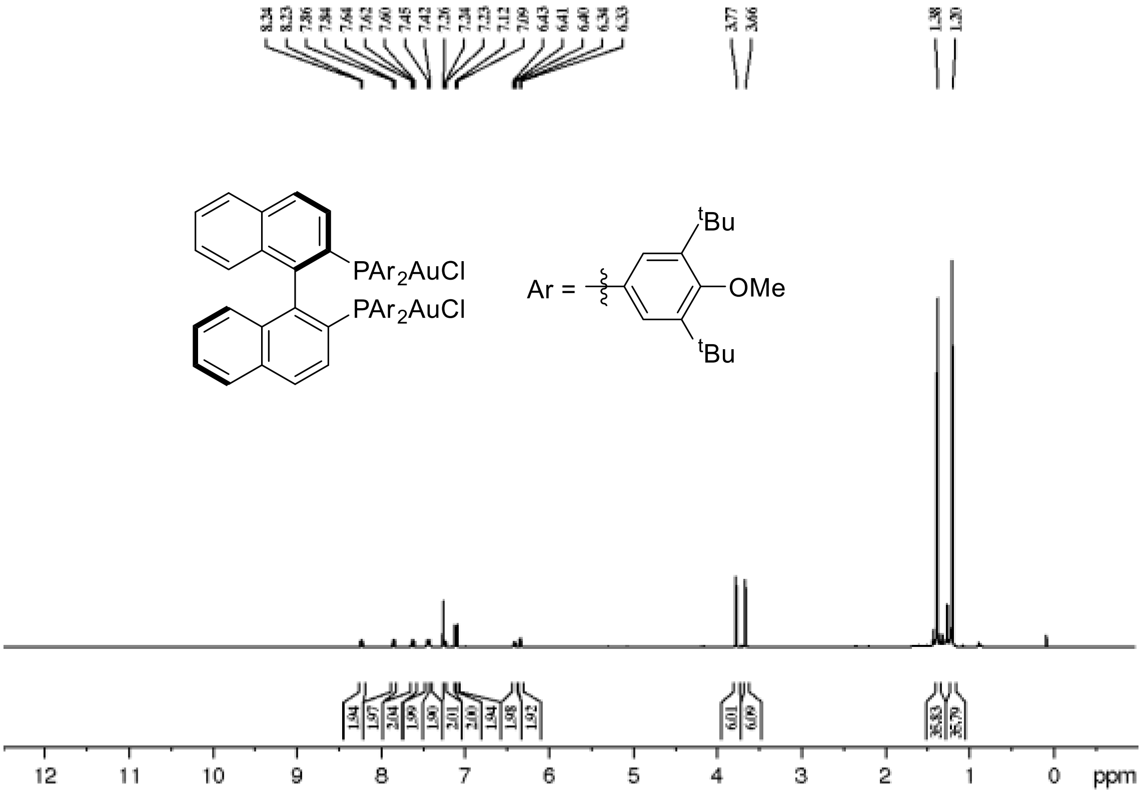

^{1}H NMR (500 MHz, $CDCl_3$) of **Au5**

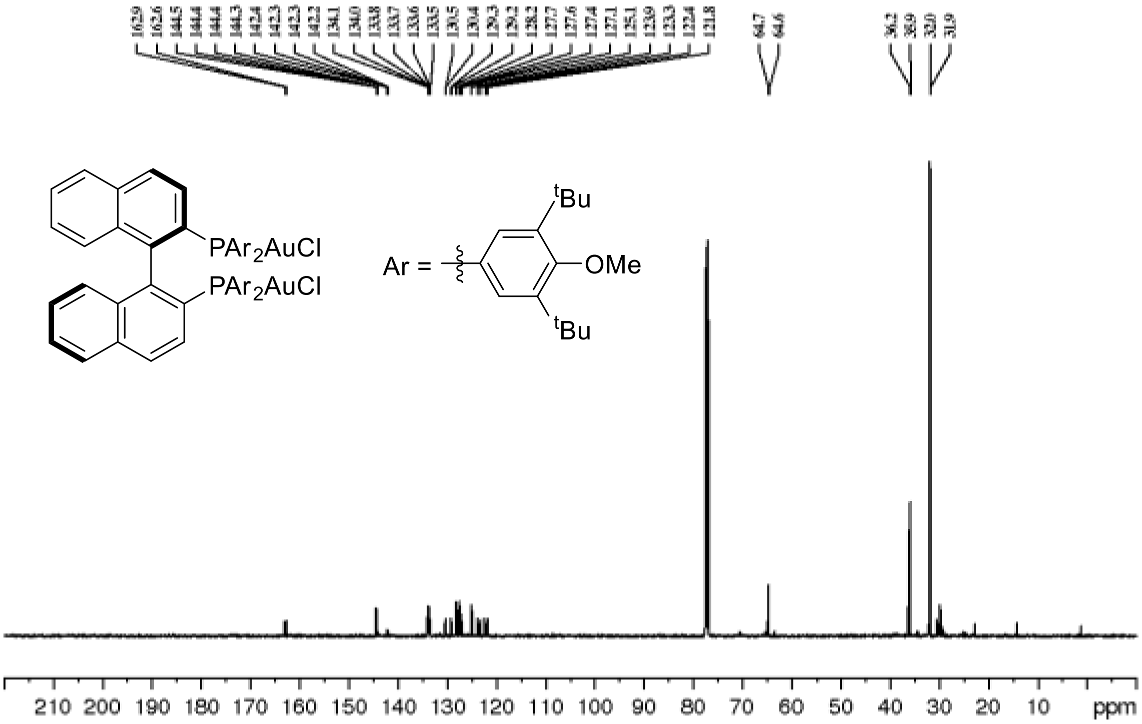

^{13}C NMR (126 MHz, $CDCl_3$) of **Au5**

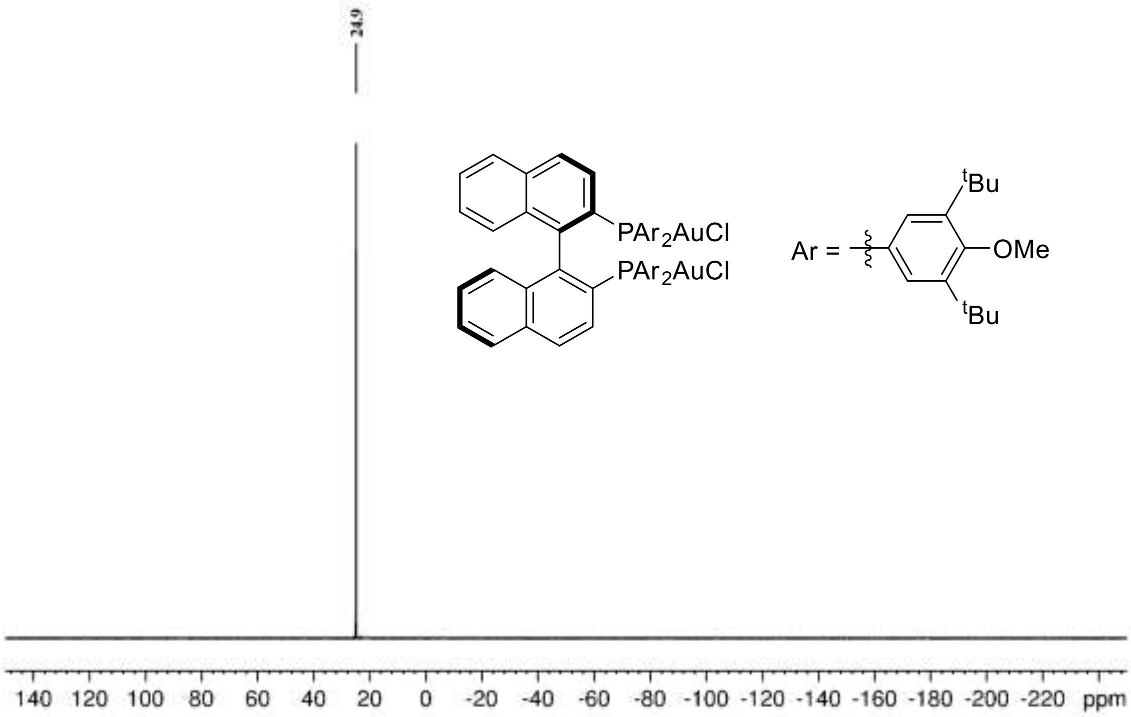

^{31}P NMR (202.5 MHz, $CDCl_3$) of **Au5**

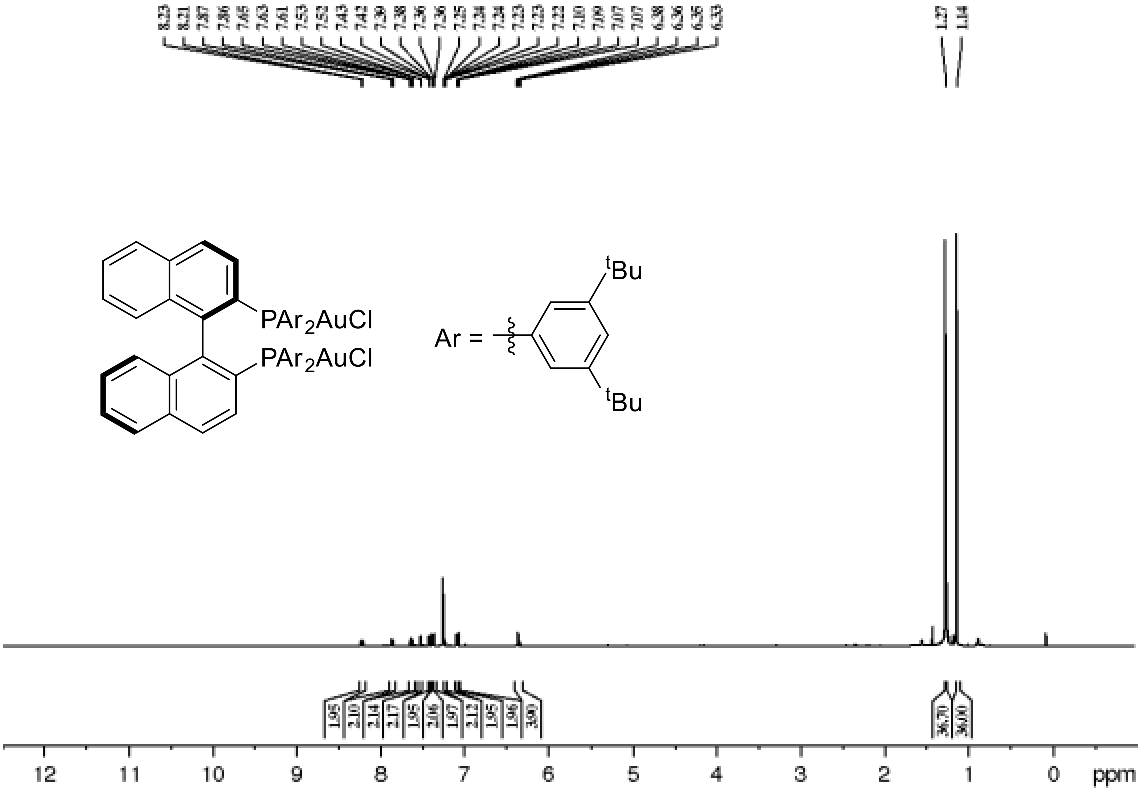

^{1}H NMR (500 MHz, $CDCl_3$) of **Au6**

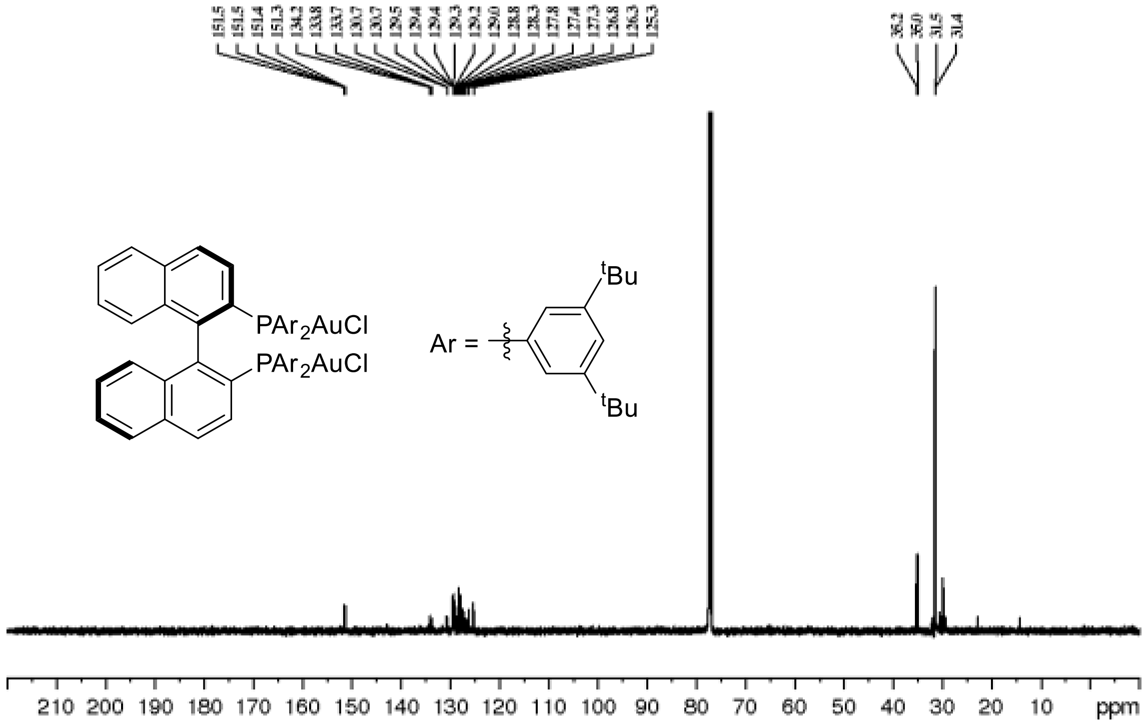

^{13}C NMR (126 MHz, $CDCl_3$) of **Au6**

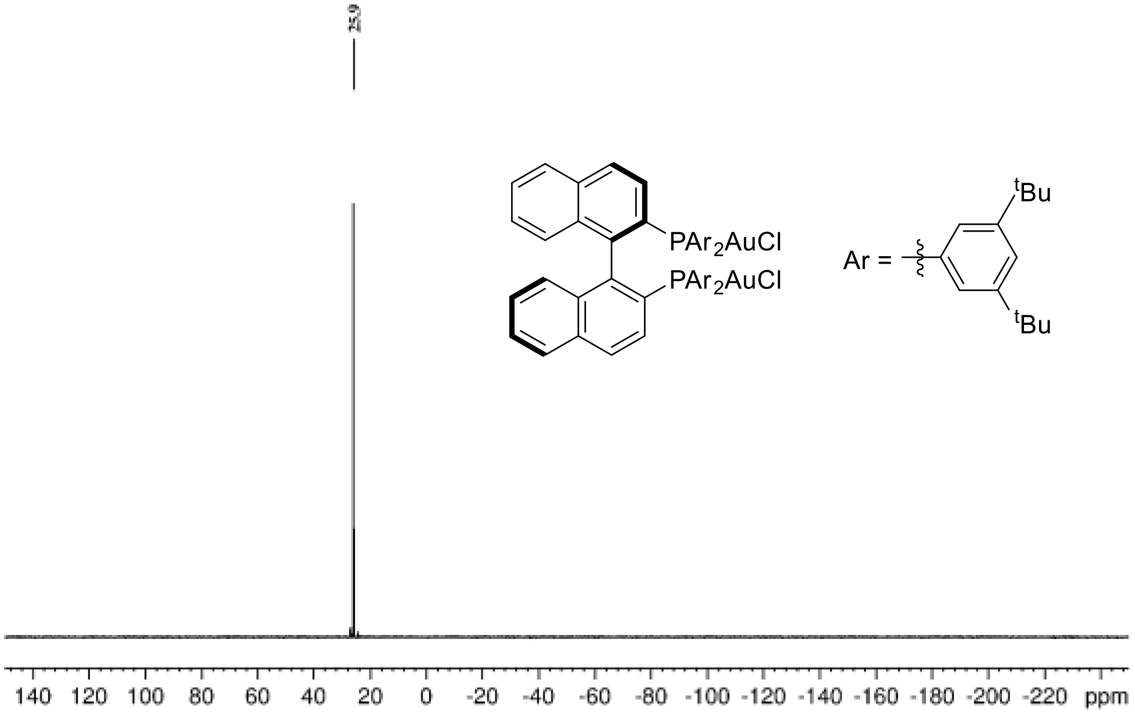

^{31}P NMR (202.5 MHz, $CDCl_3$) of **Au6**

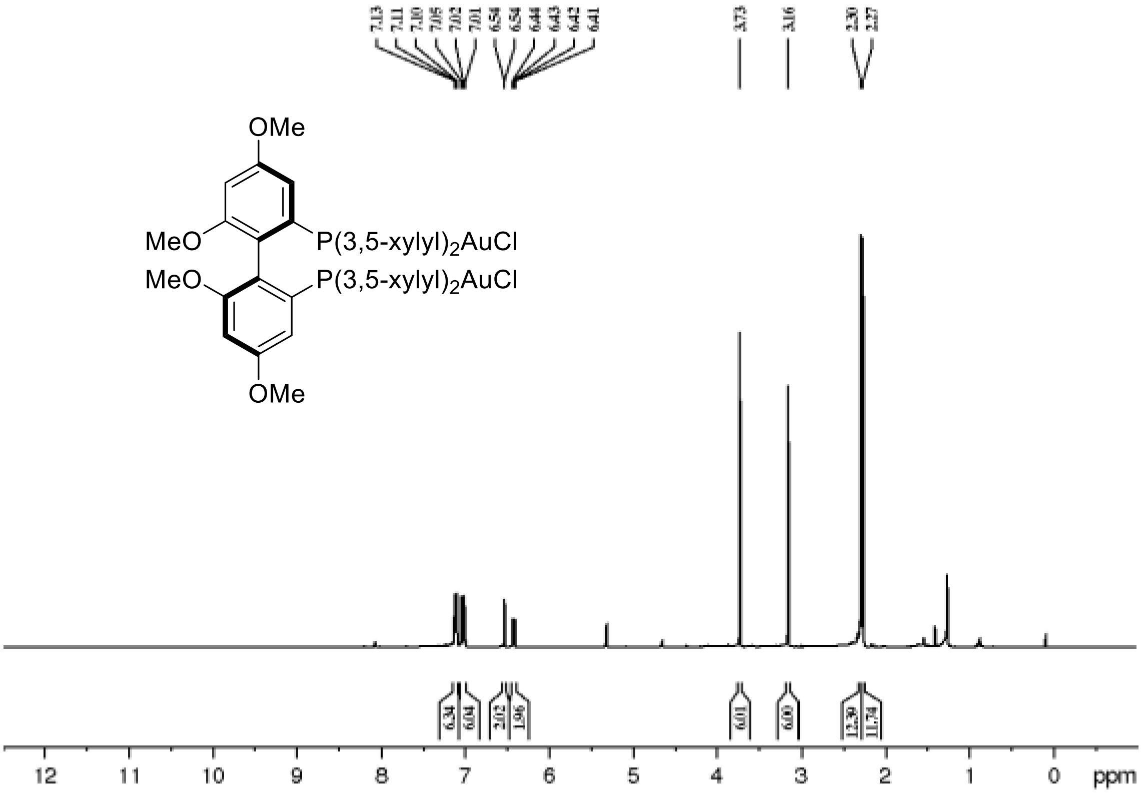

^{1}H NMR (500 MHz, CD_2Cl_2) of **Au8**

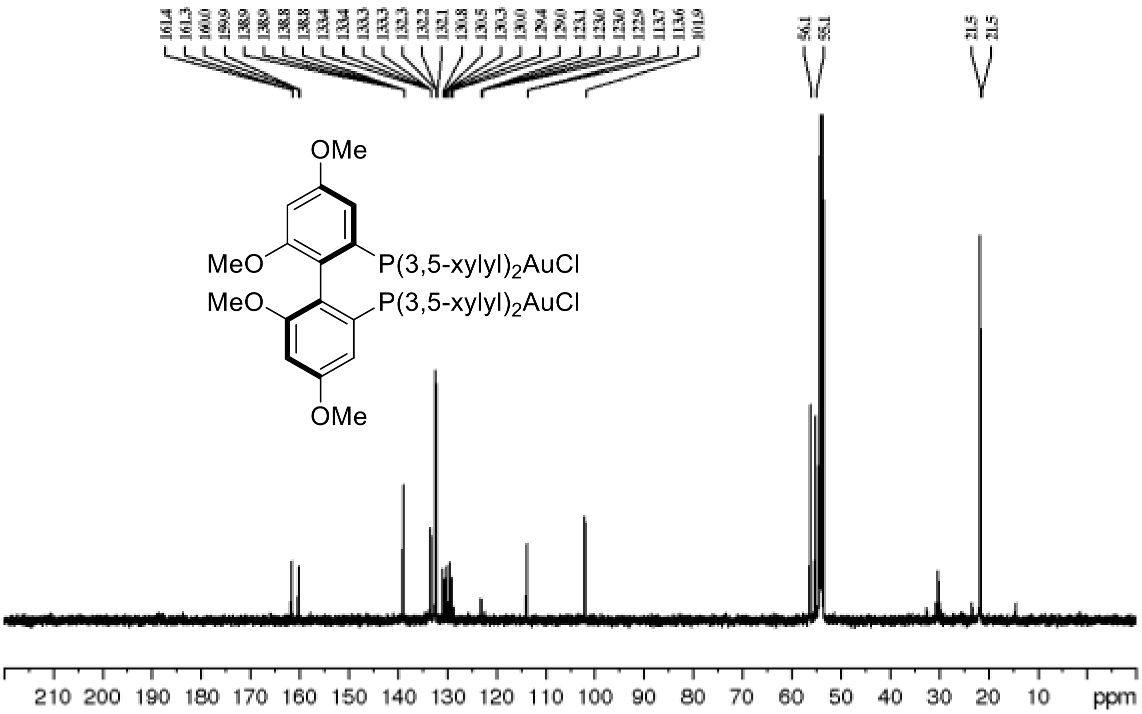

^{13}C NMR (126 MHz, CD_2Cl_2) of **Au8**

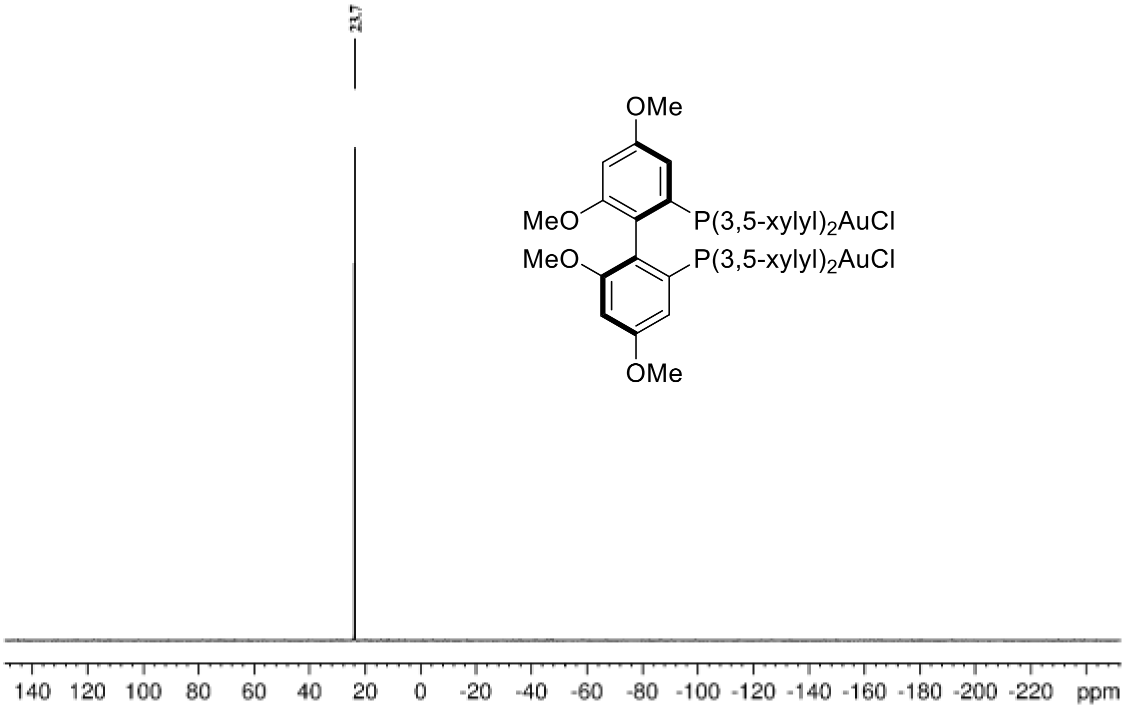

^{31}P NMR (202.5 MHz, CD_2Cl_2) of **Au8**

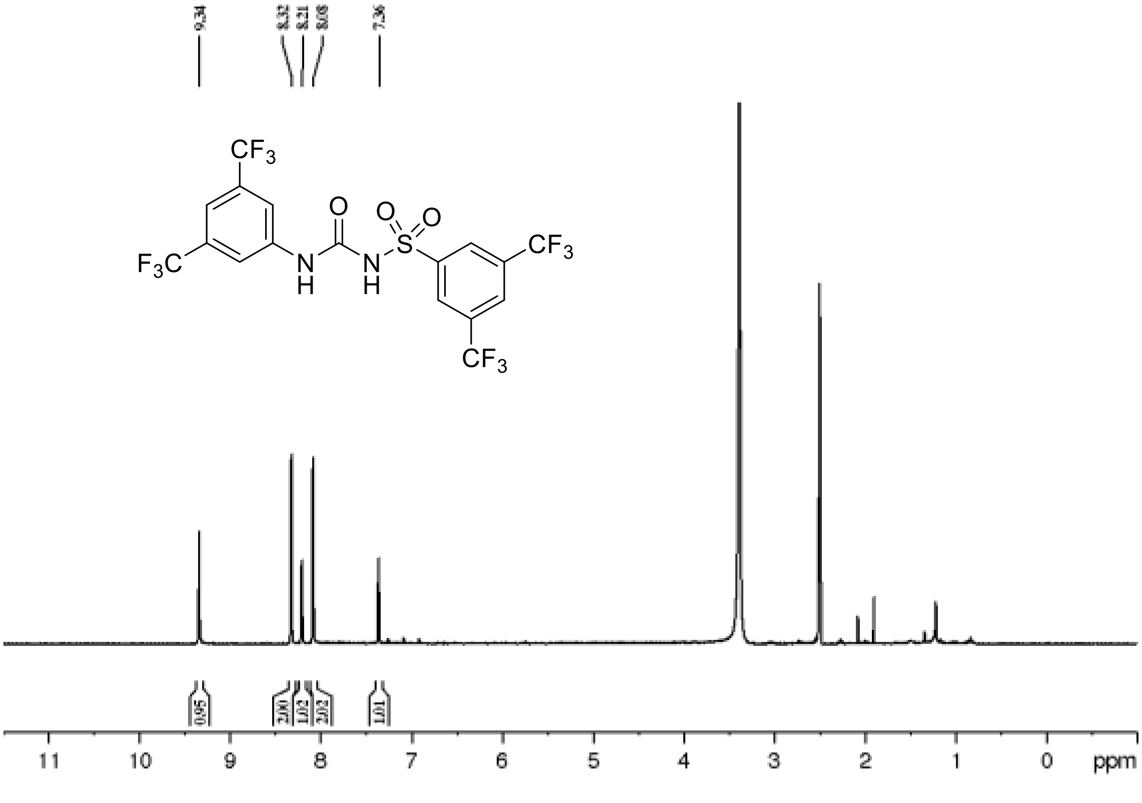

^{1}H NMR (300 MHz, DMSO-d_6) of **III**

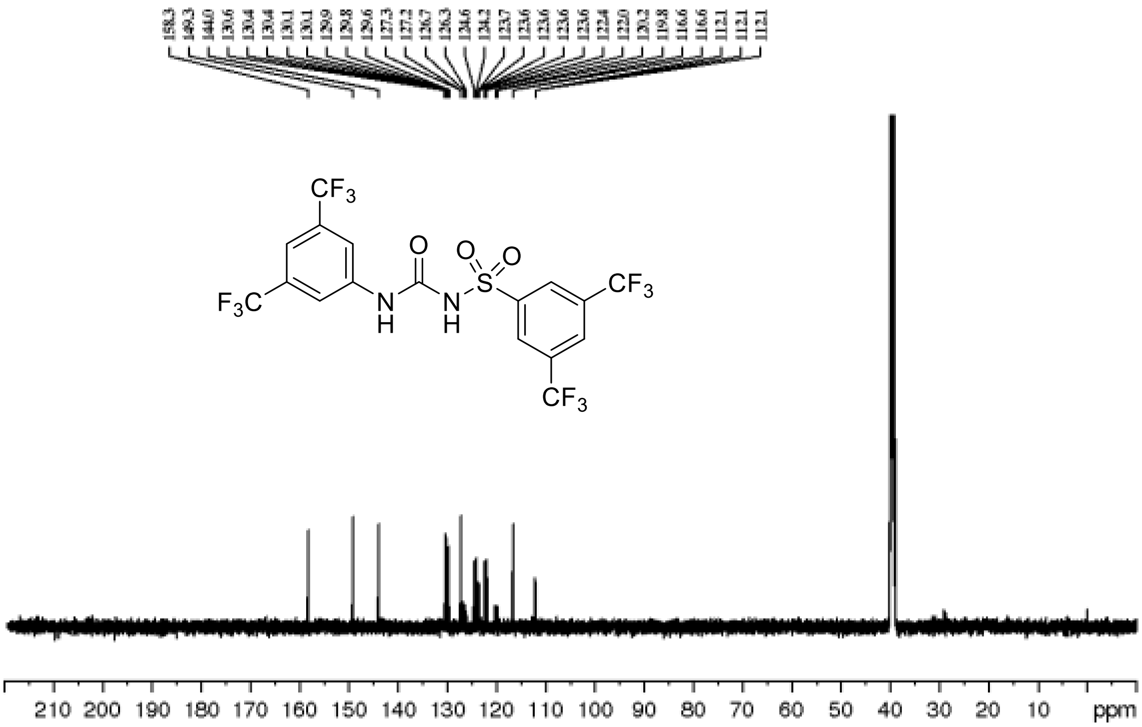

^{13}C NMR (126 MHz, DMSO-d_6) of **III**

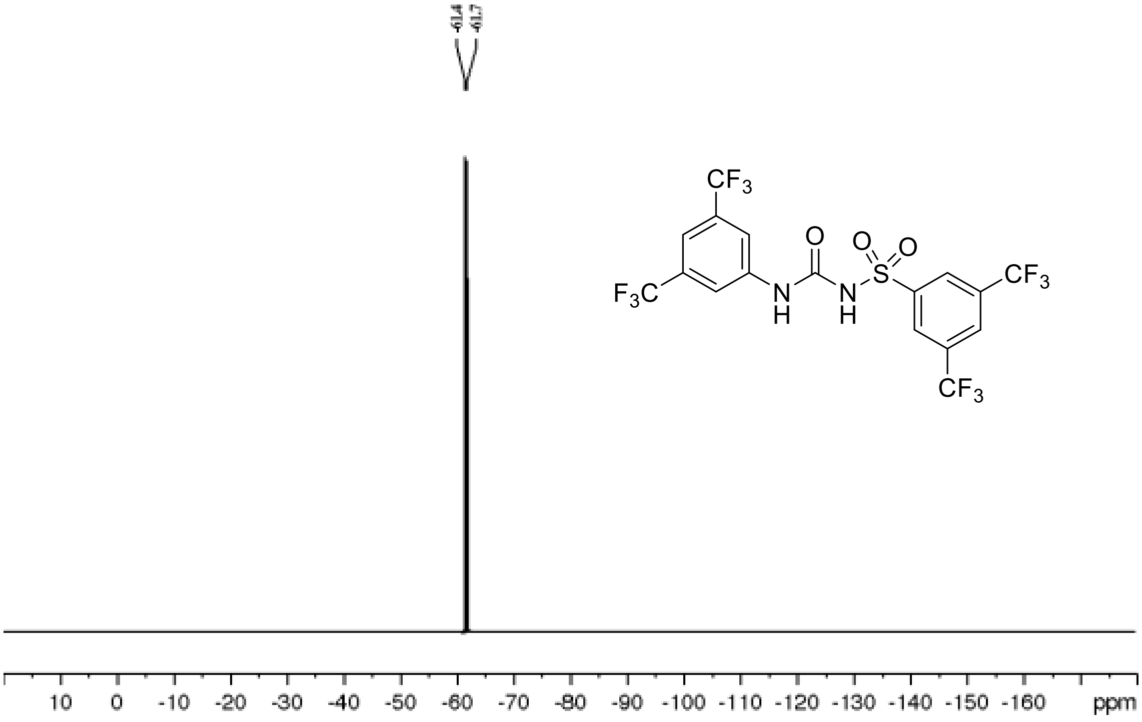

^{19}F NMR (282 MHz, DMSO-d_6) of **III**

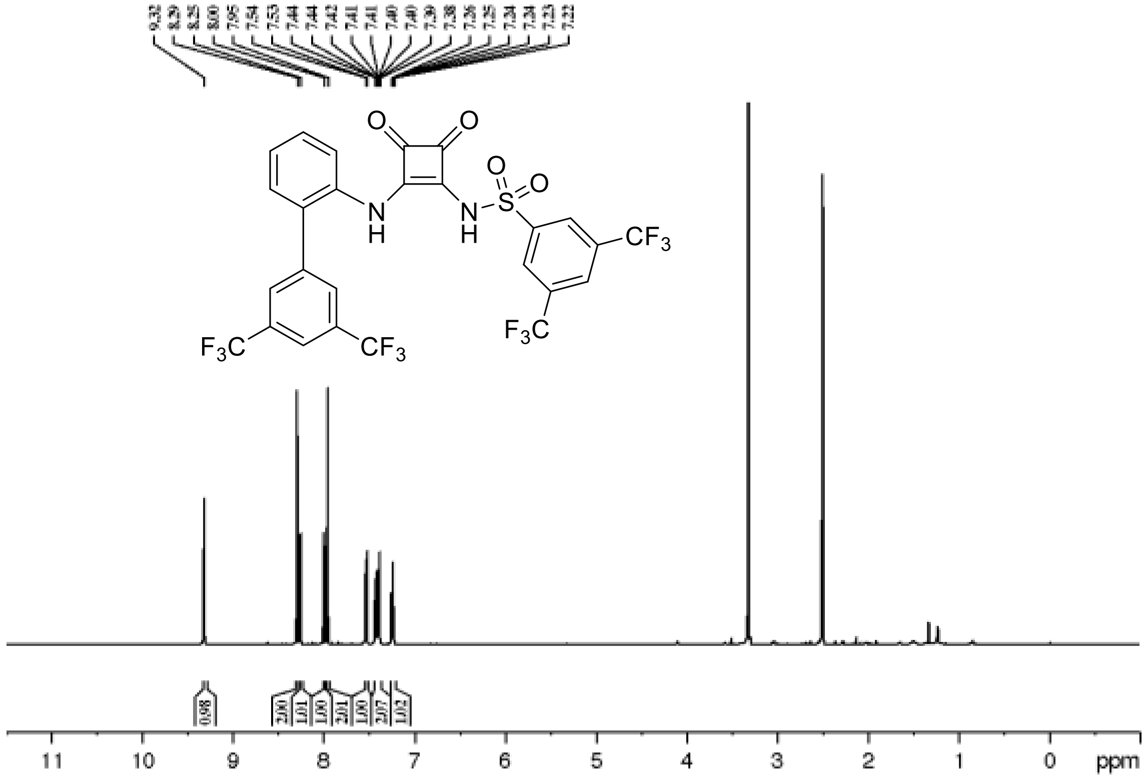

^{1}H NMR (500 MHz, DMSO-d_6) of **V**

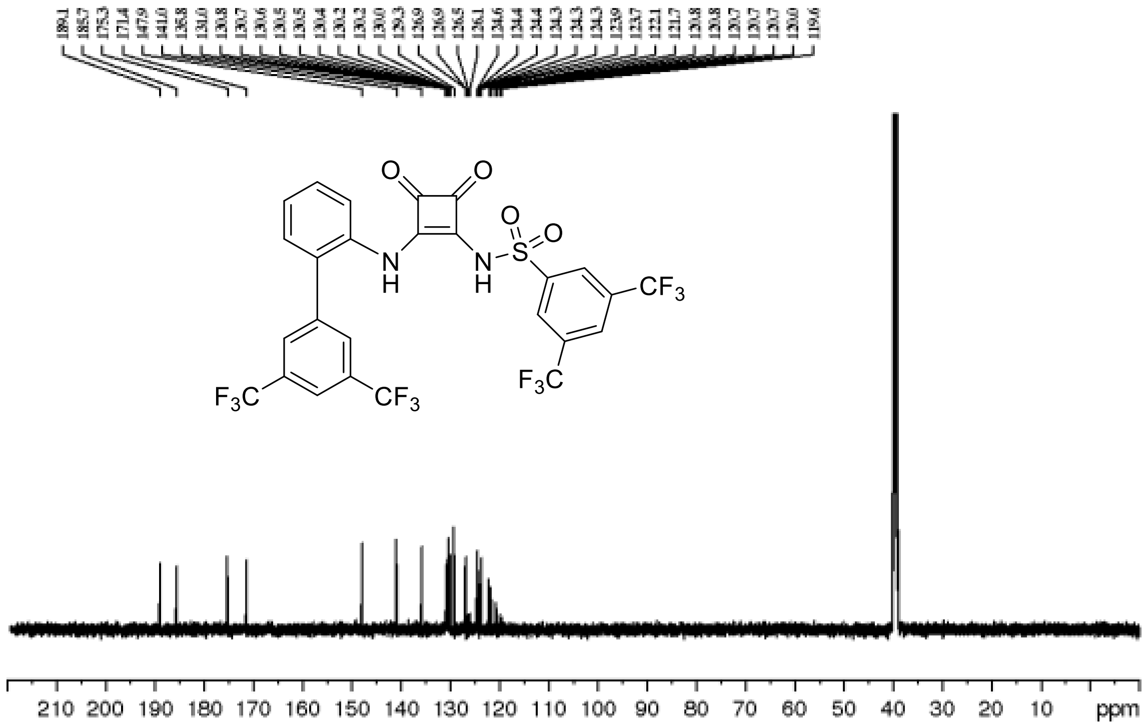

^{13}C NMR (126 MHz, DMSO-d_6) of **V**

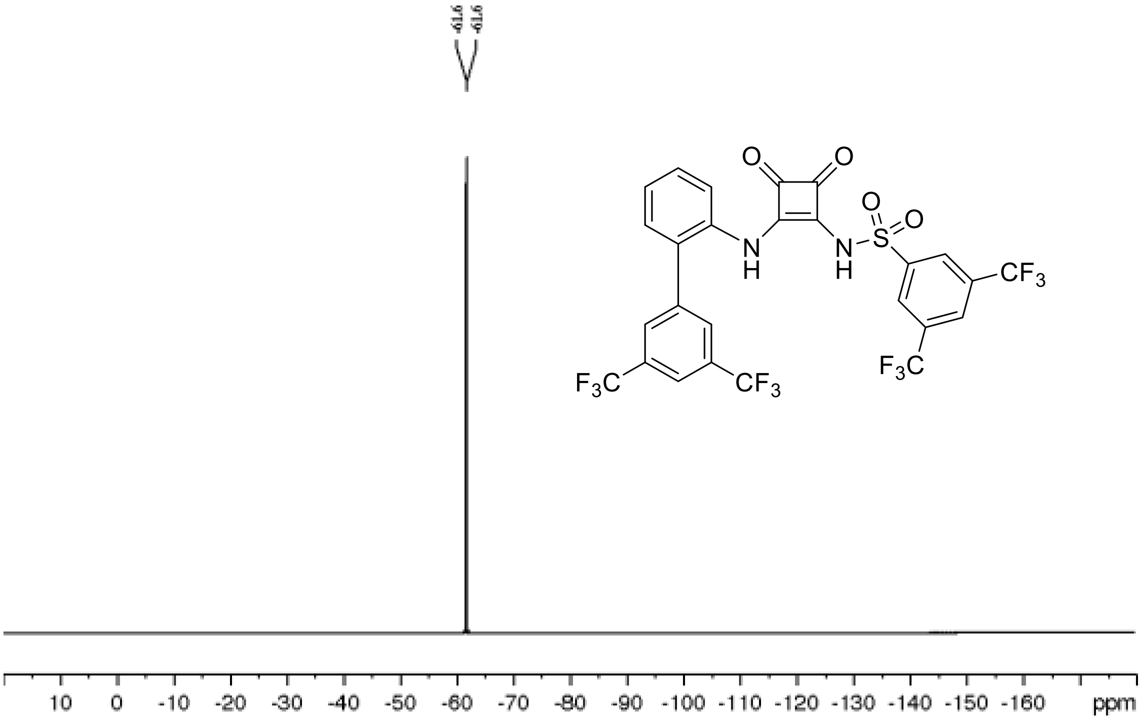

^{19}F NMR (471 MHz, DMSO-d_6) of **V**

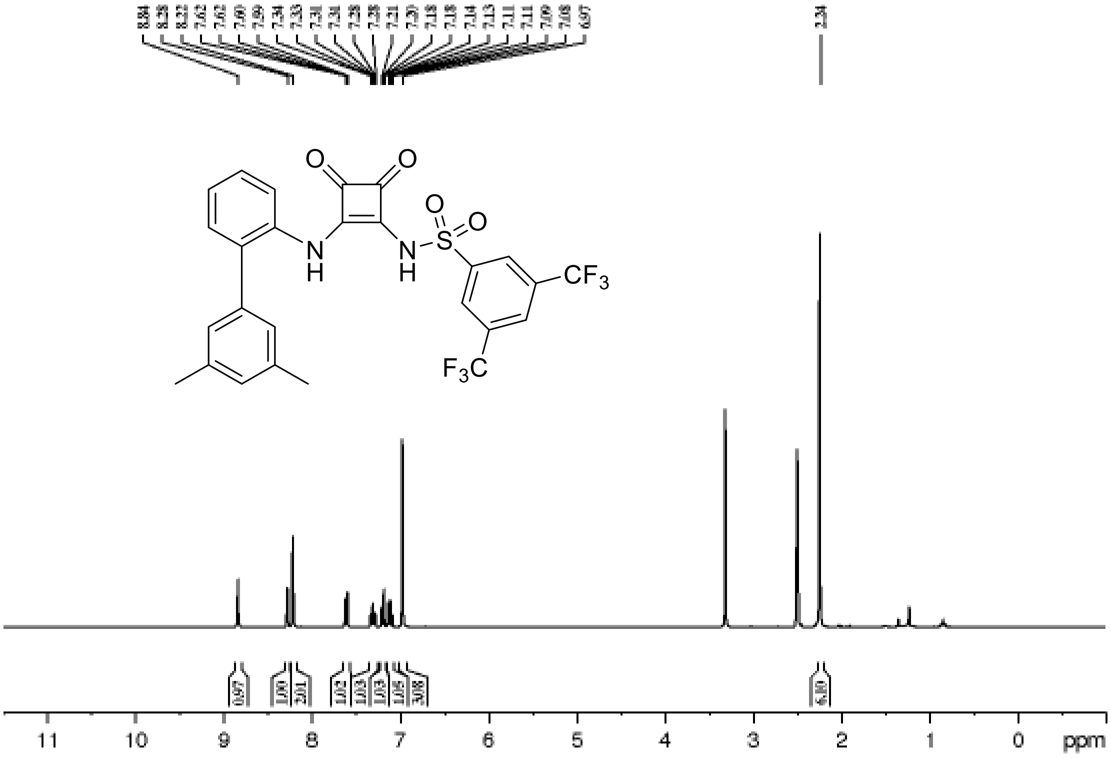

^{1}H NMR (500 MHz, DMSO-d_6) of **VI**

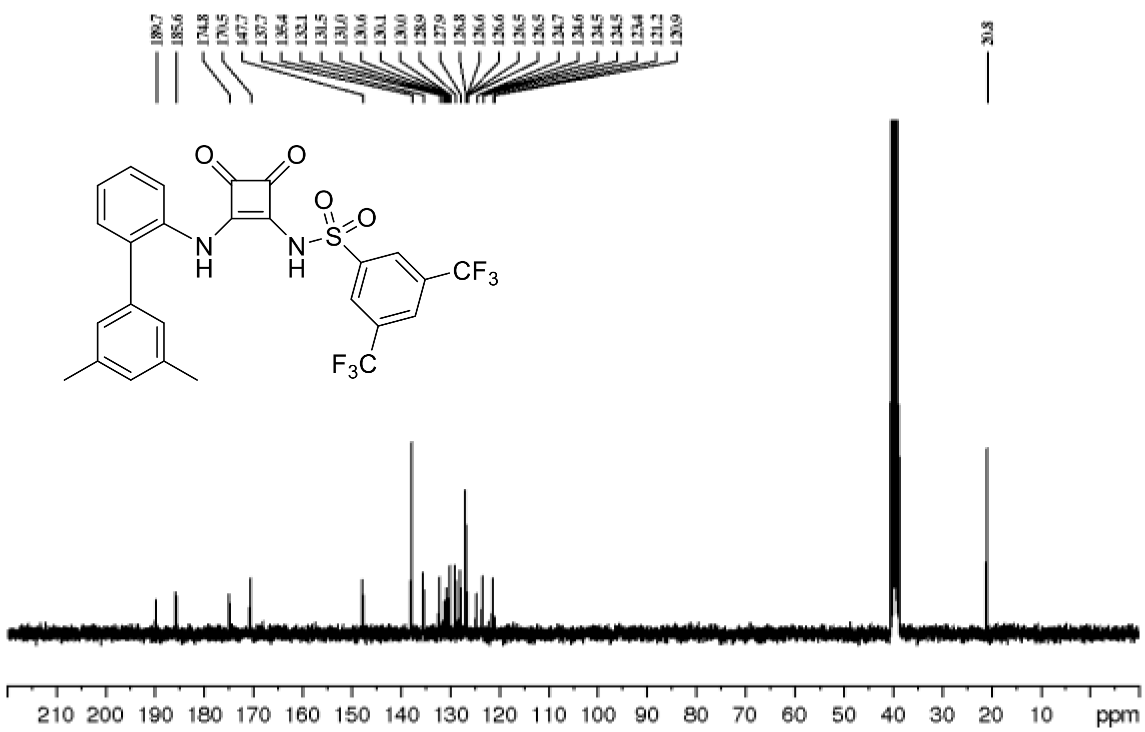

^{13}C NMR (126 MHz, DMSO-d_6) of **VI**

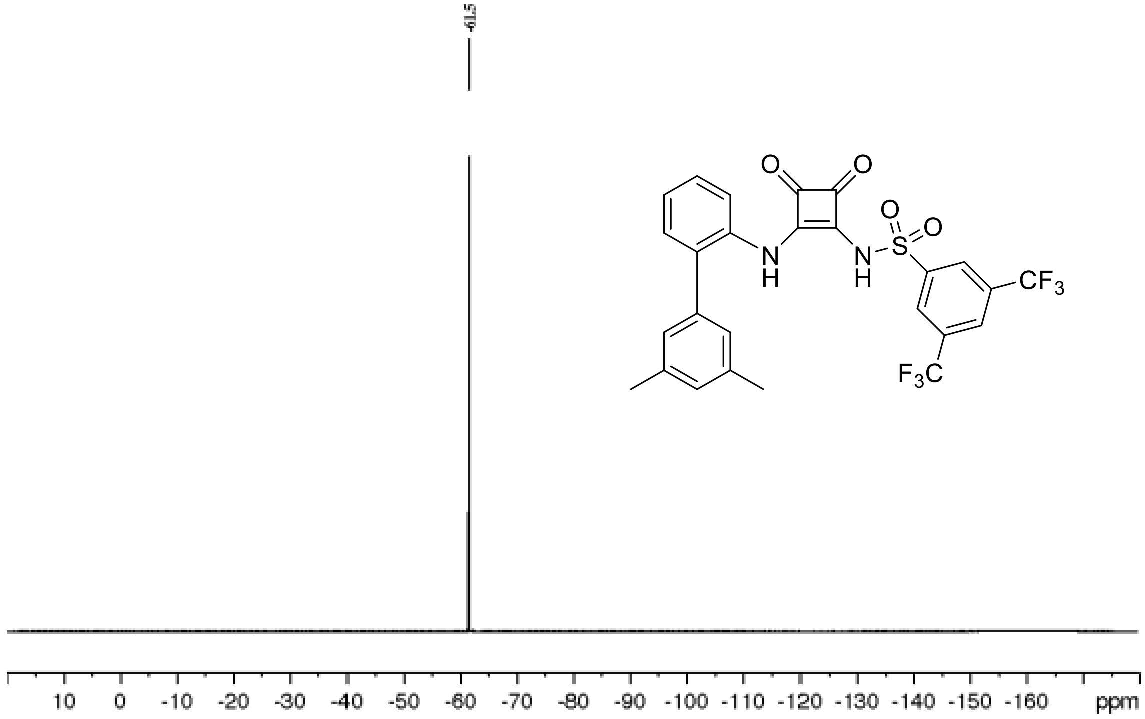

^{19}F NMR (282 MHz, DMSO-d_6) of **VI**

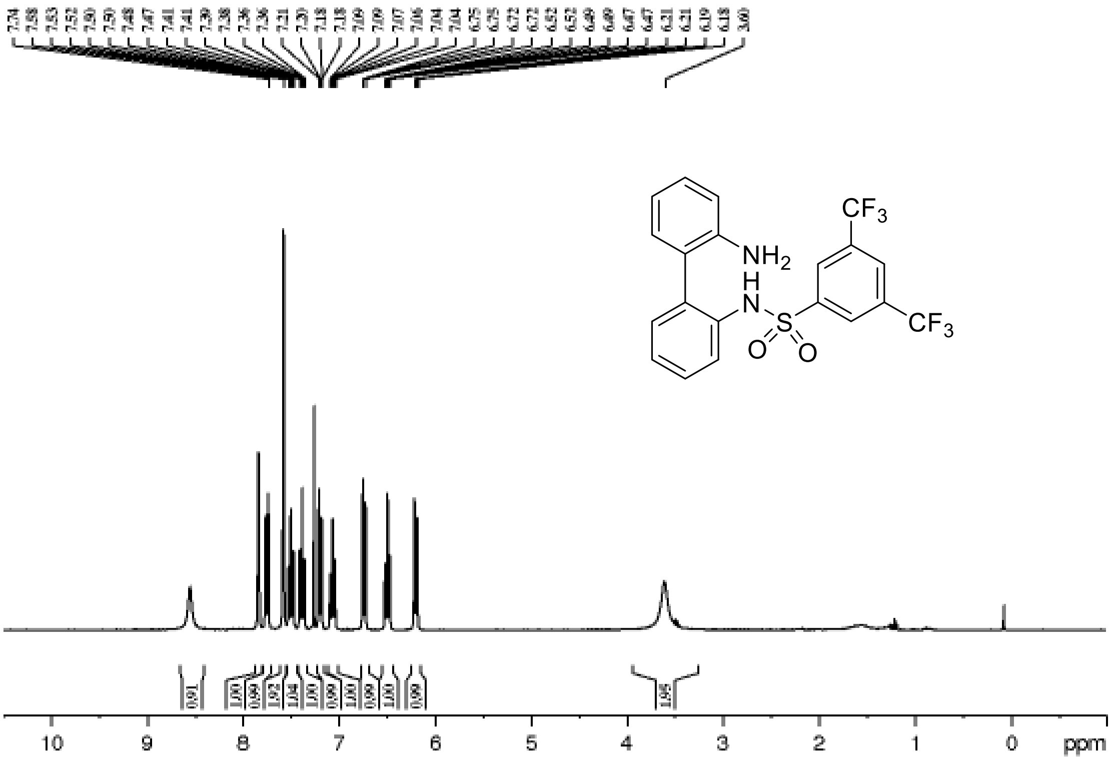

^{1}H NMR (300 MHz, DMSO-*d*$_6$) of **I-1**

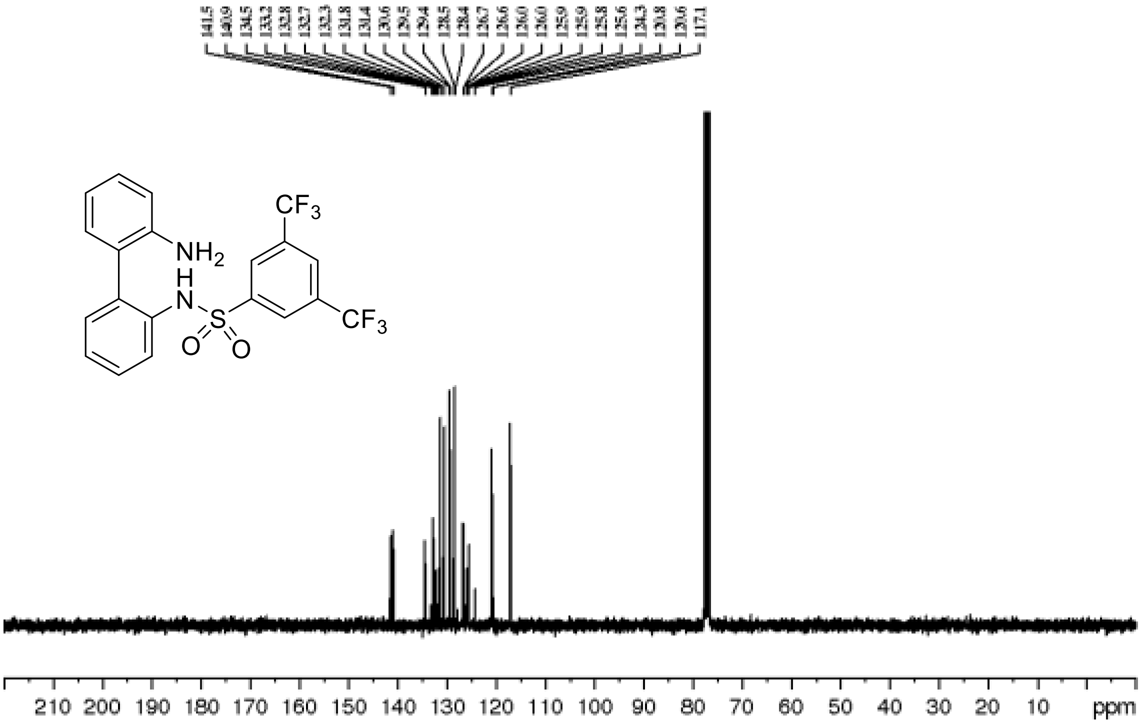

^{13}C NMR (75.5 MHz, DMSO-*d*$_6$) of **I-1**

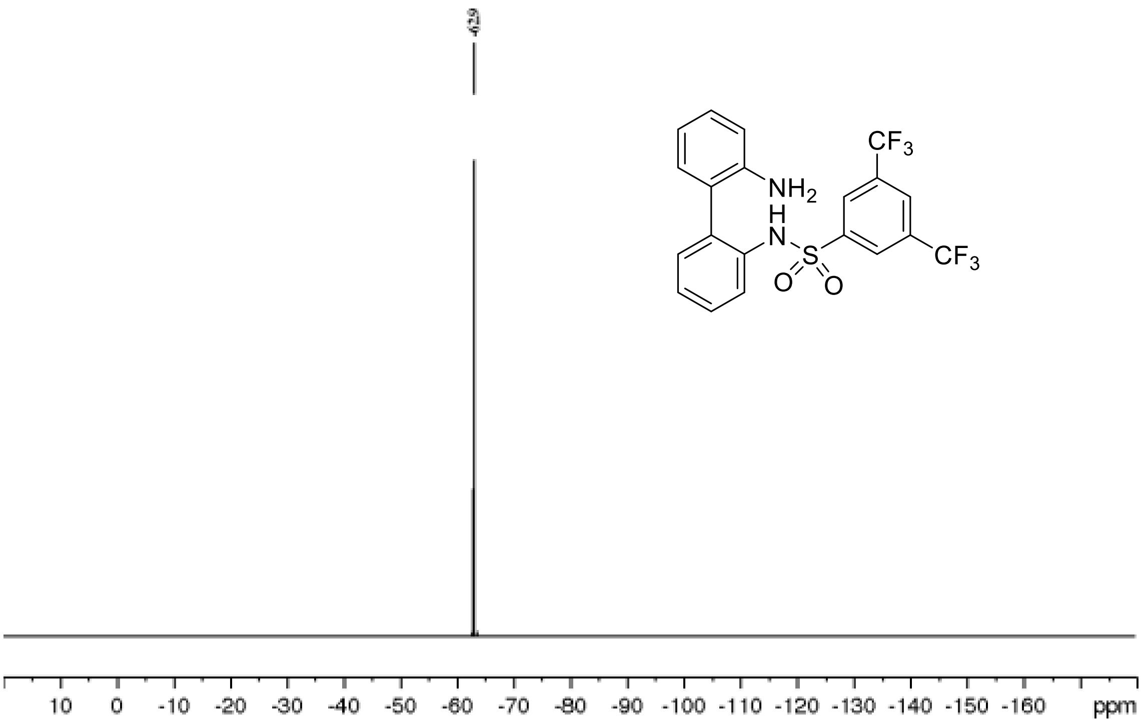

^{19}F NMR (282 MHz, DMSO-d_6) of **I-1**

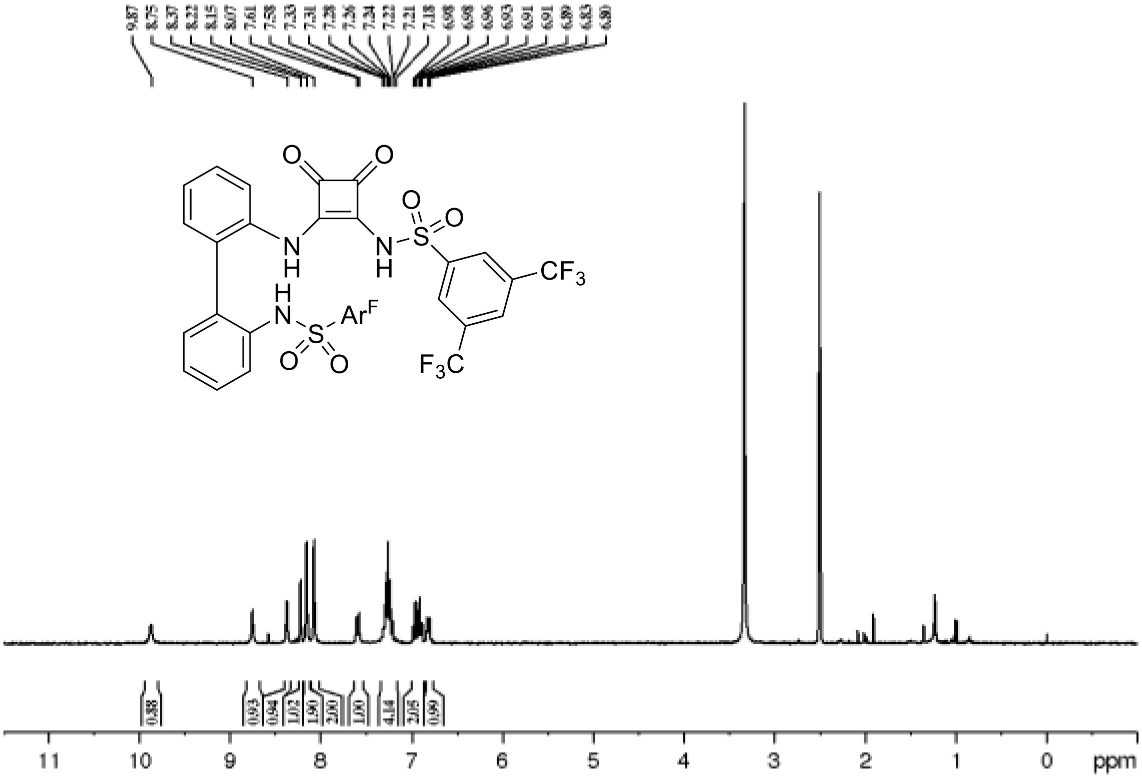

^{1}H NMR (300 MHz, DMSO-d_6) of **VII**

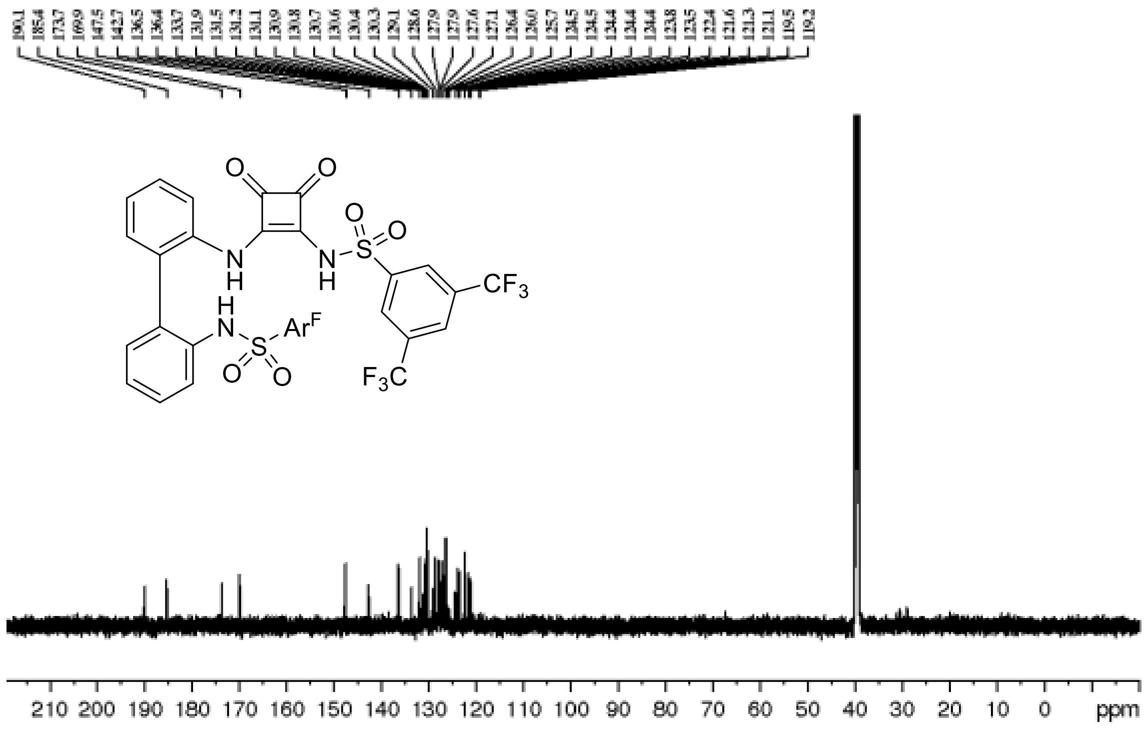

^{13}C NMR (126 MHz, DMSO-d_6) of **VII**

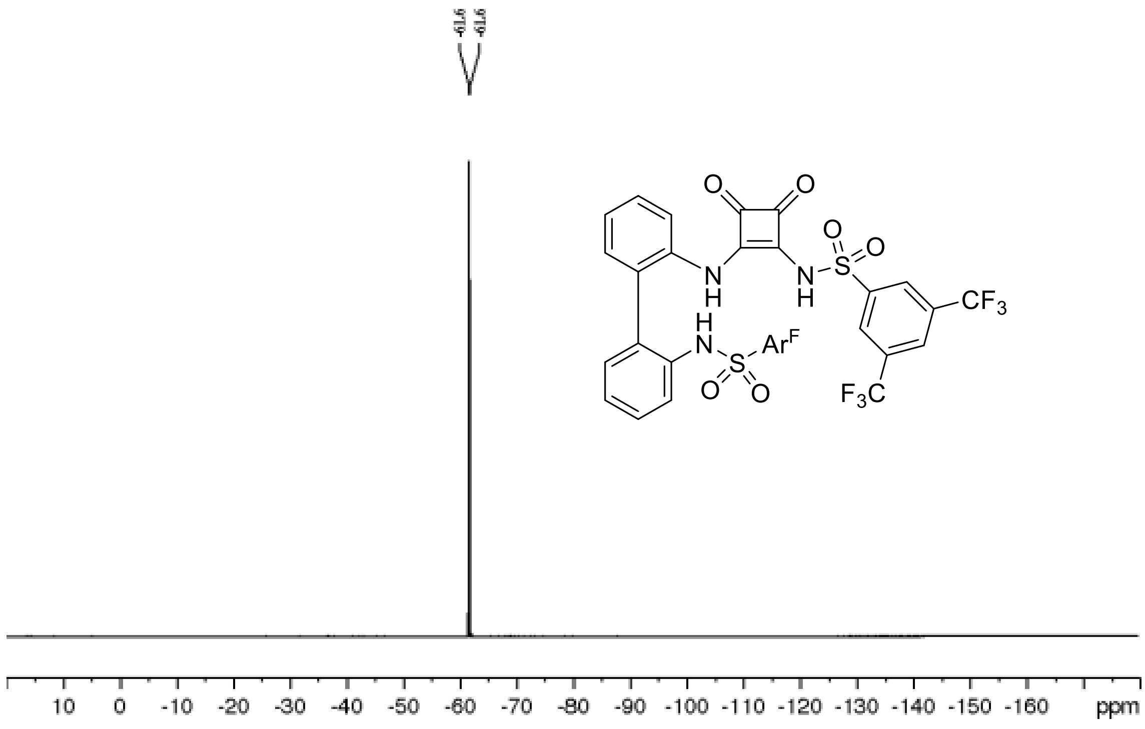

^{19}F NMR (471 MHz, DMSO-d_6) of **VII**

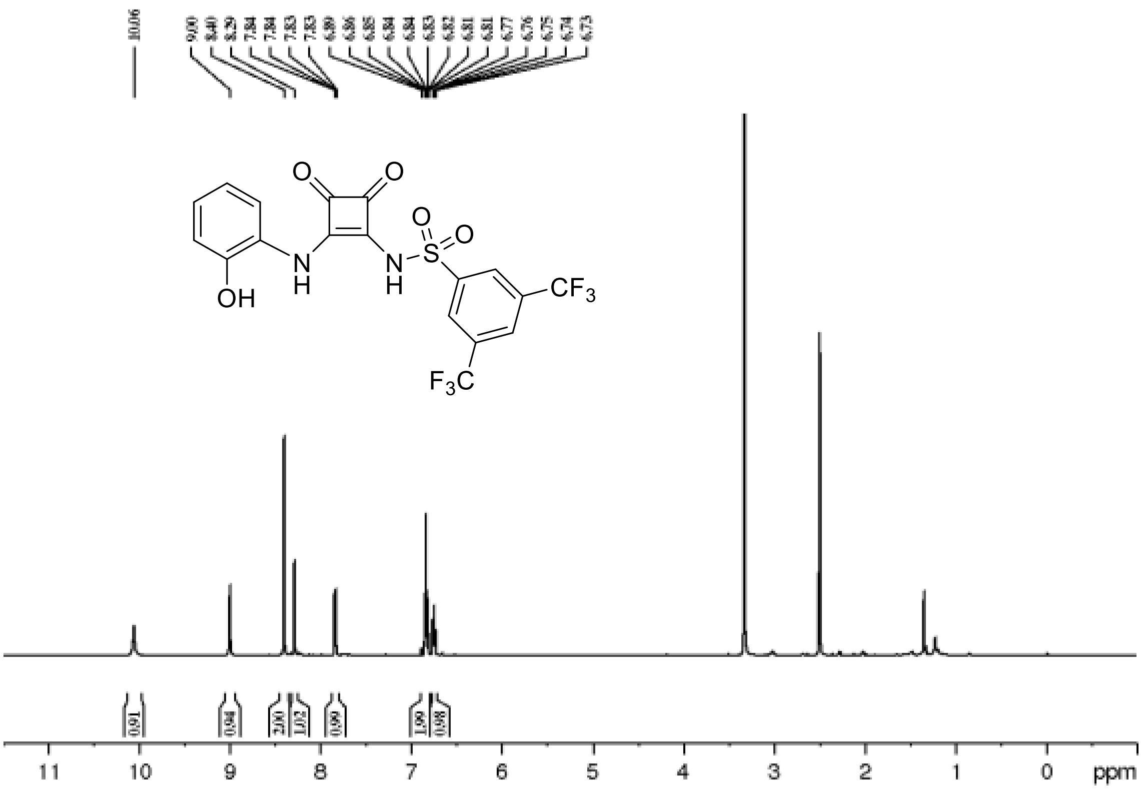

^{1}H NMR (500 MHz, DMSO-d_6) of **VIII**

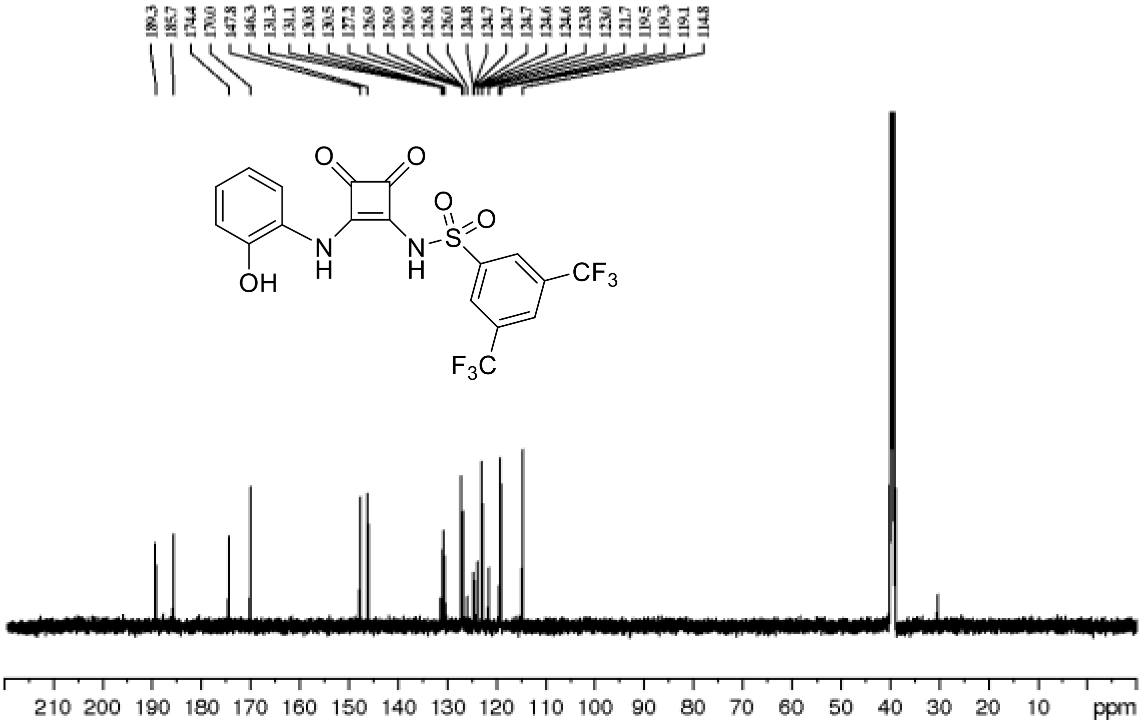

^{13}C NMR (126 MHz, DMSO-d_6) of **VIII**

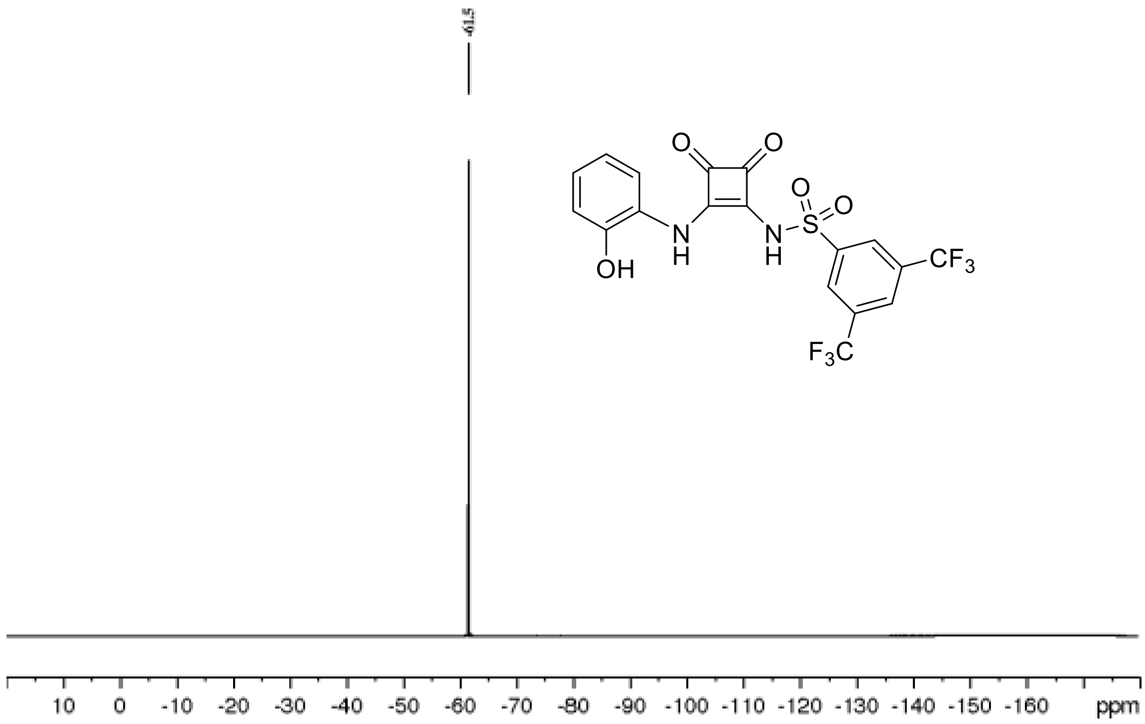

^{19}F NMR (471 MHz, DMSO-d_6) of **VIII**

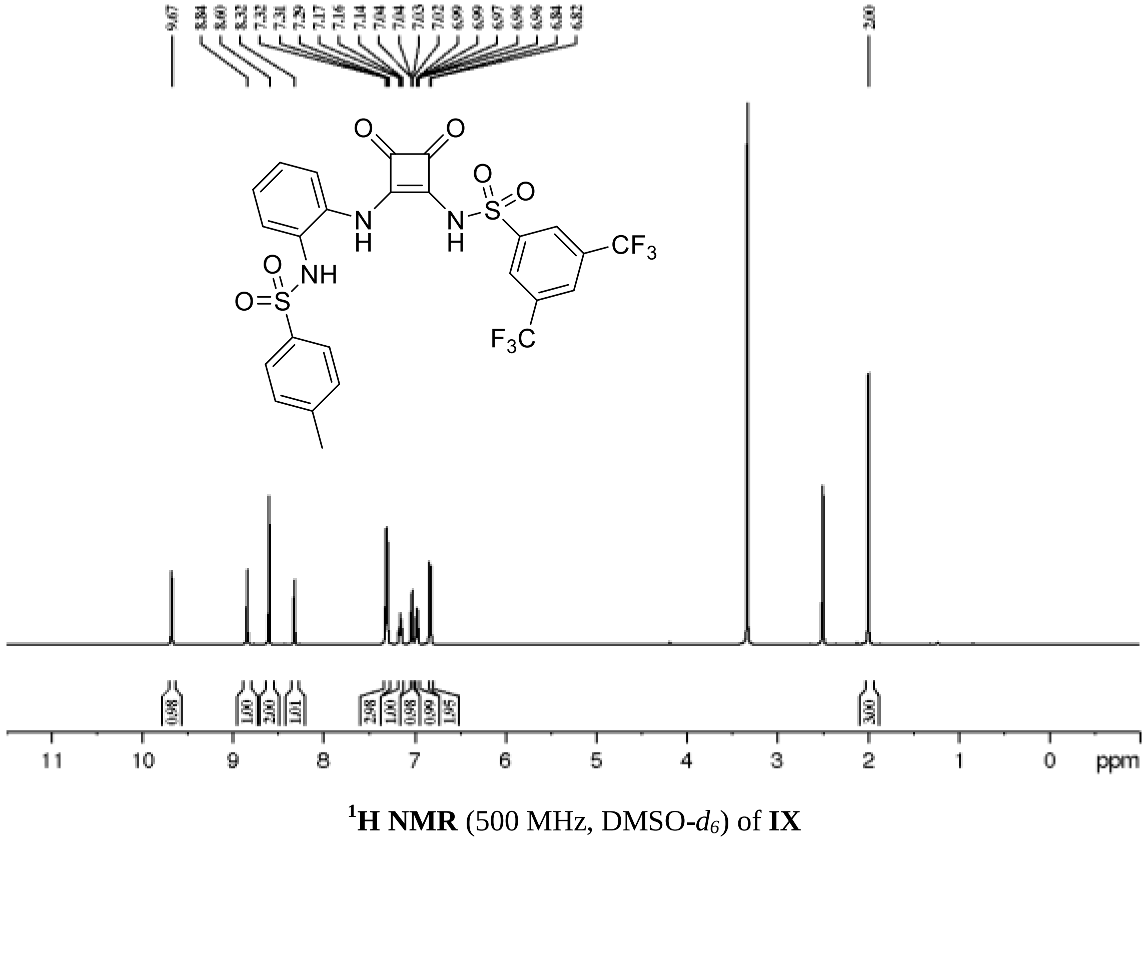

^{1}H NMR (500 MHz, DMSO-d_6) of **IX**

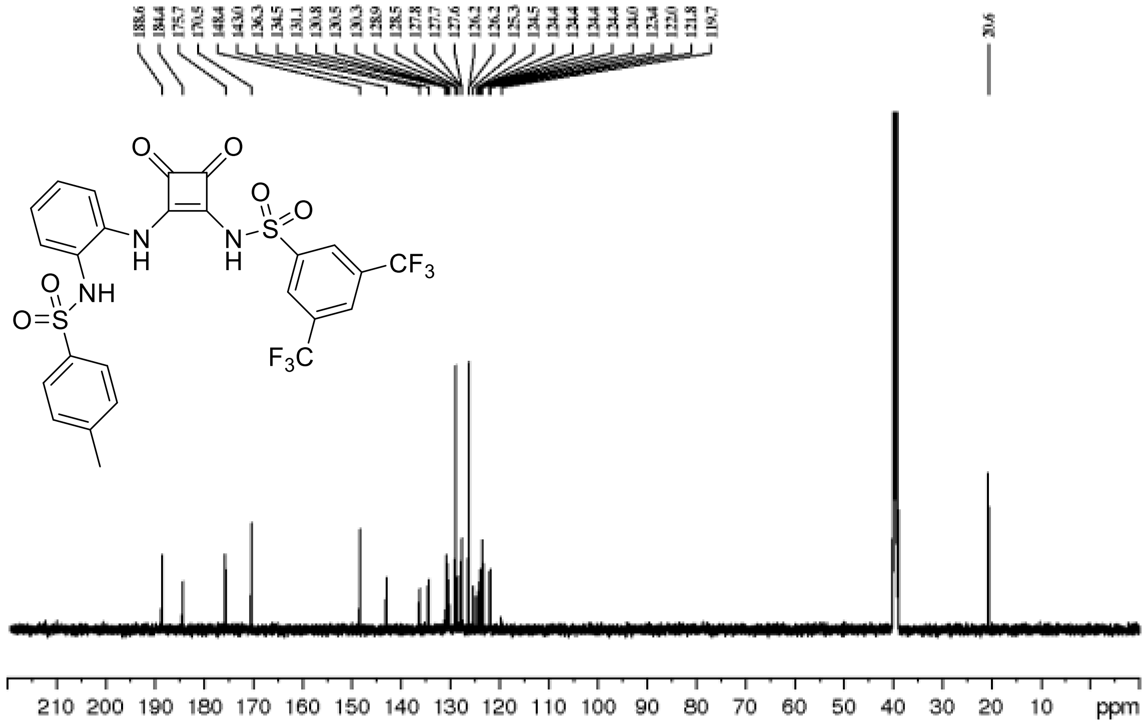

^{13}C NMR (126 MHz, DMSO-d_6) of **IX**

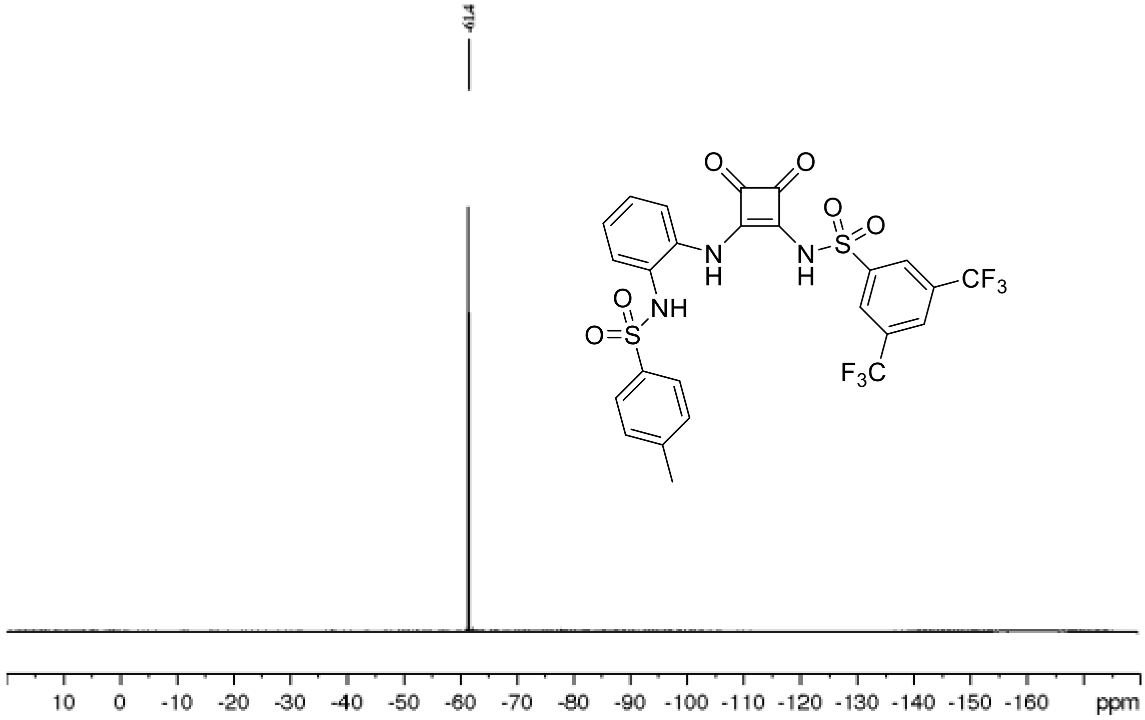

^{19}F NMR (282 MHz, DMSO-d_6) of **IX**

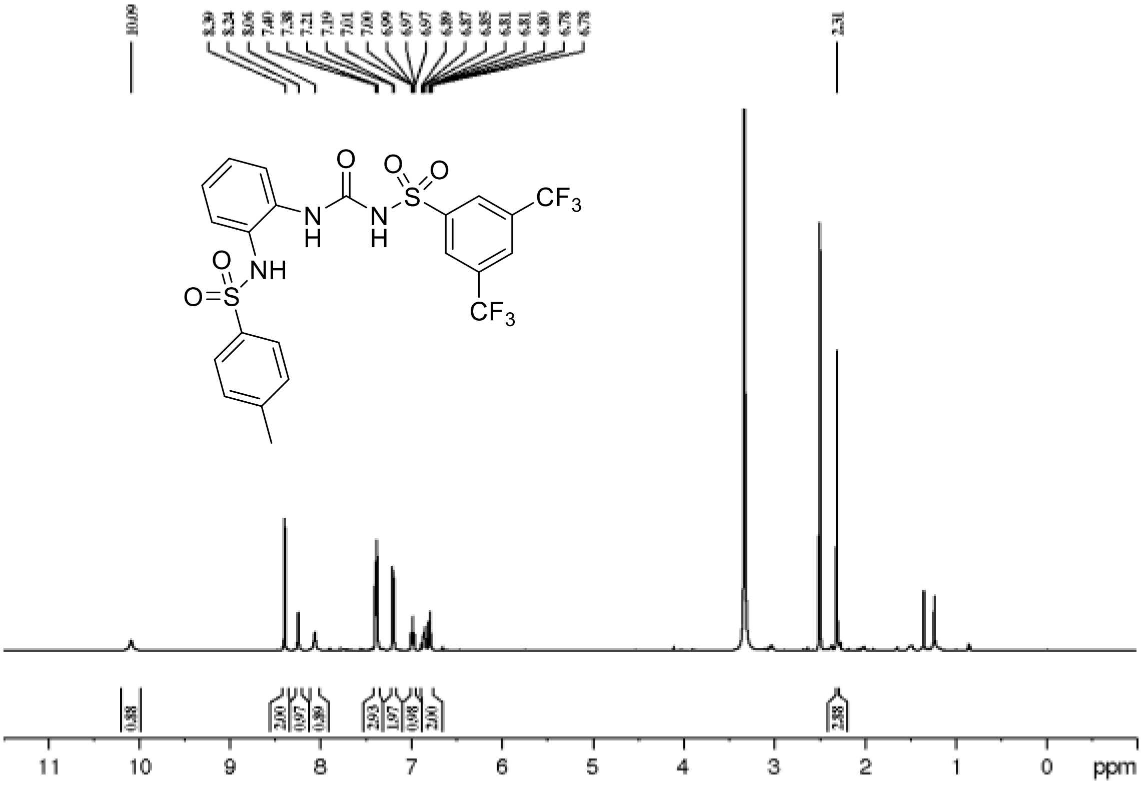

^{1}H NMR (500 MHz, DMSO-*d*$_6$) of **X**

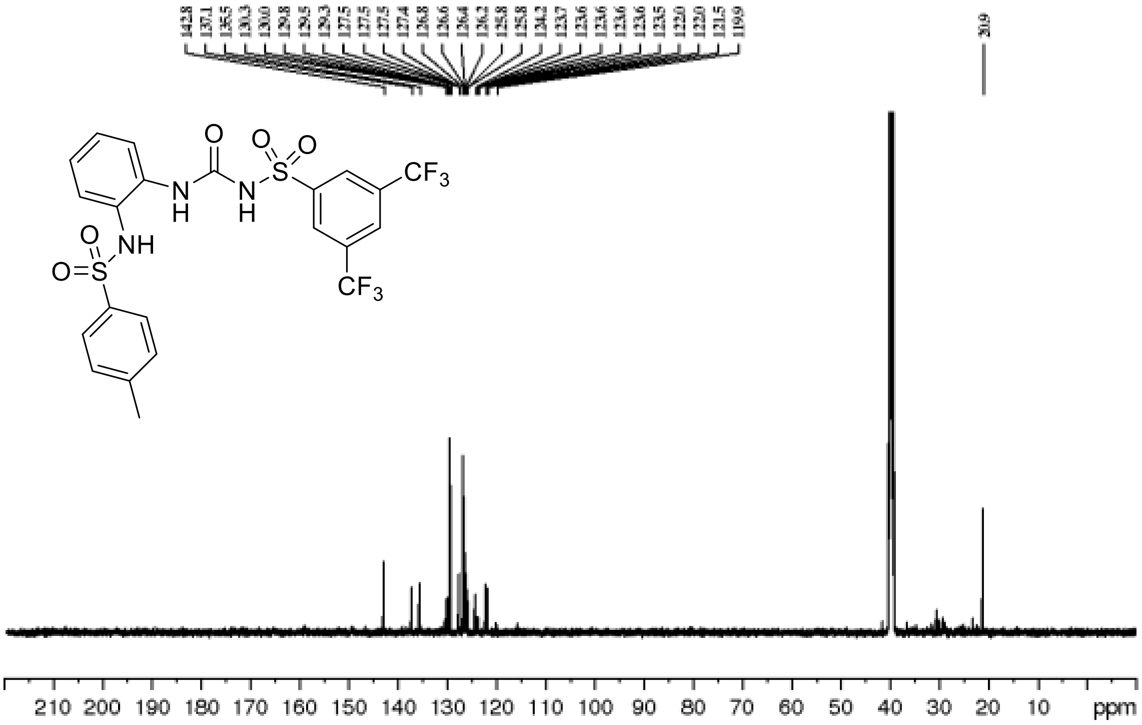

^{13}C NMR (126 MHz, DMSO-*d*$_6$) of **X**

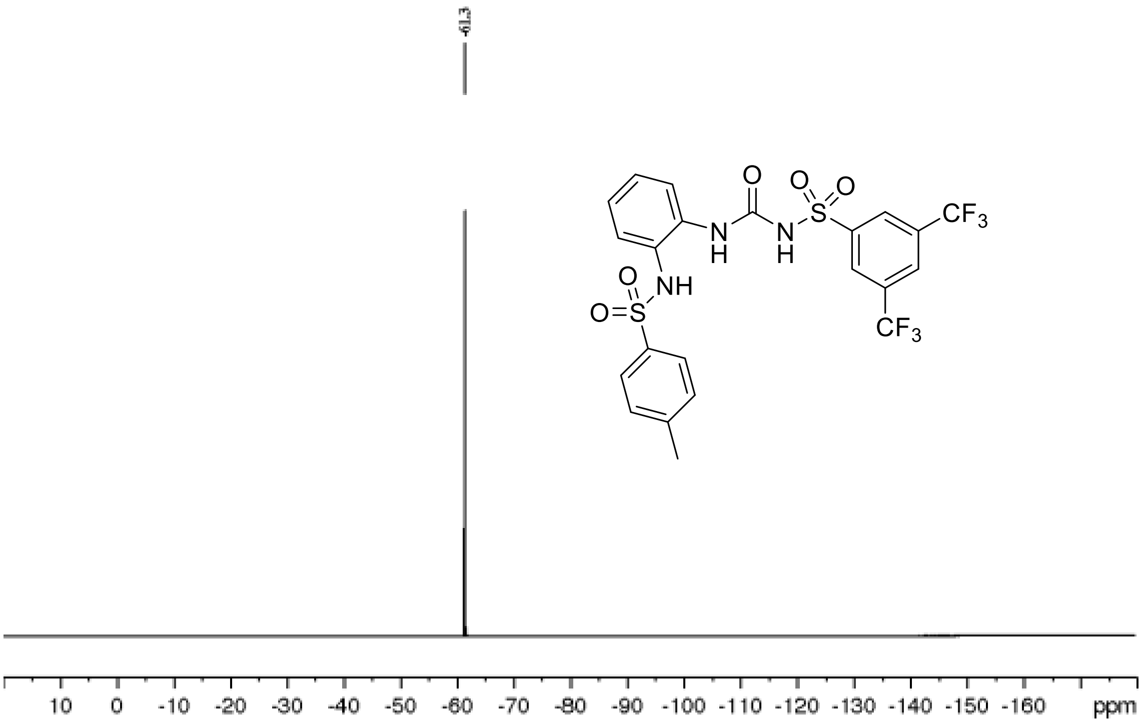

^{19}F NMR (471 MHz, DMSO-d_6) of **X**

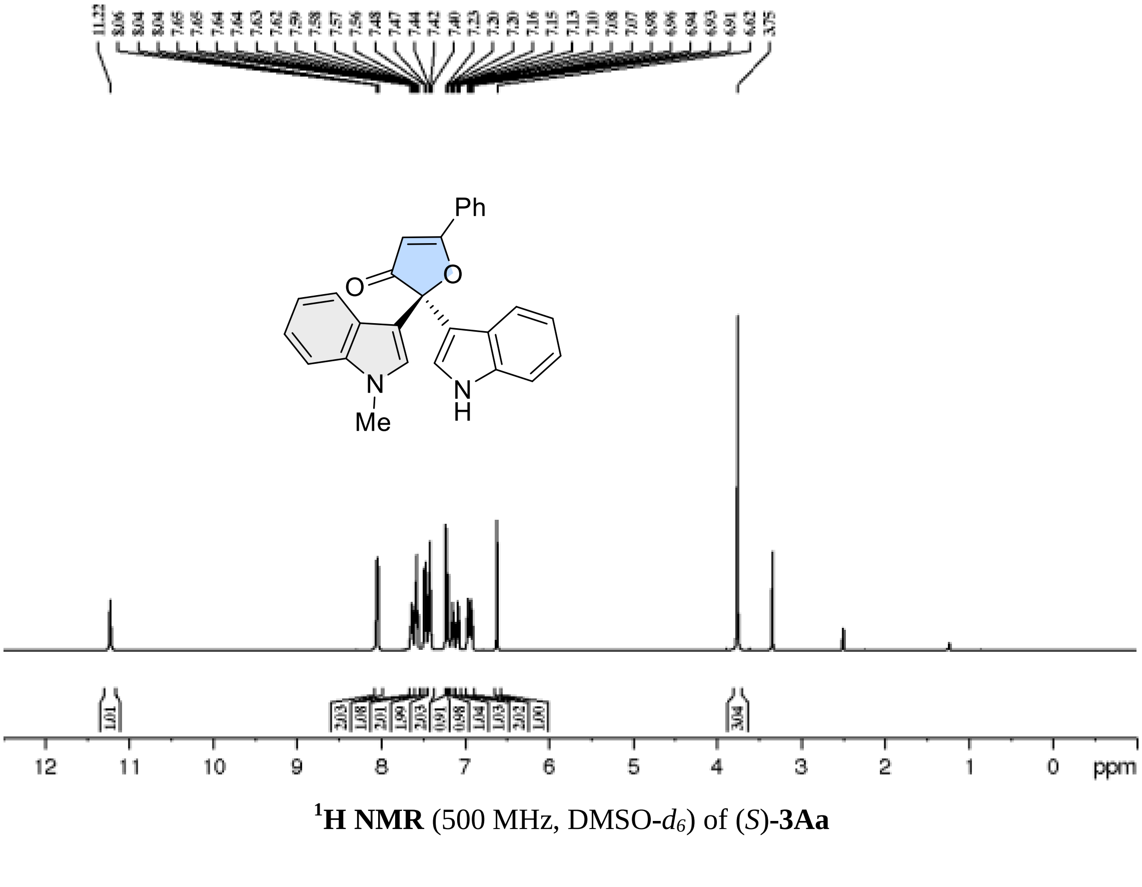

^{1}H NMR (500 MHz, DMSO-d_6) of (*S*)-**3Aa**

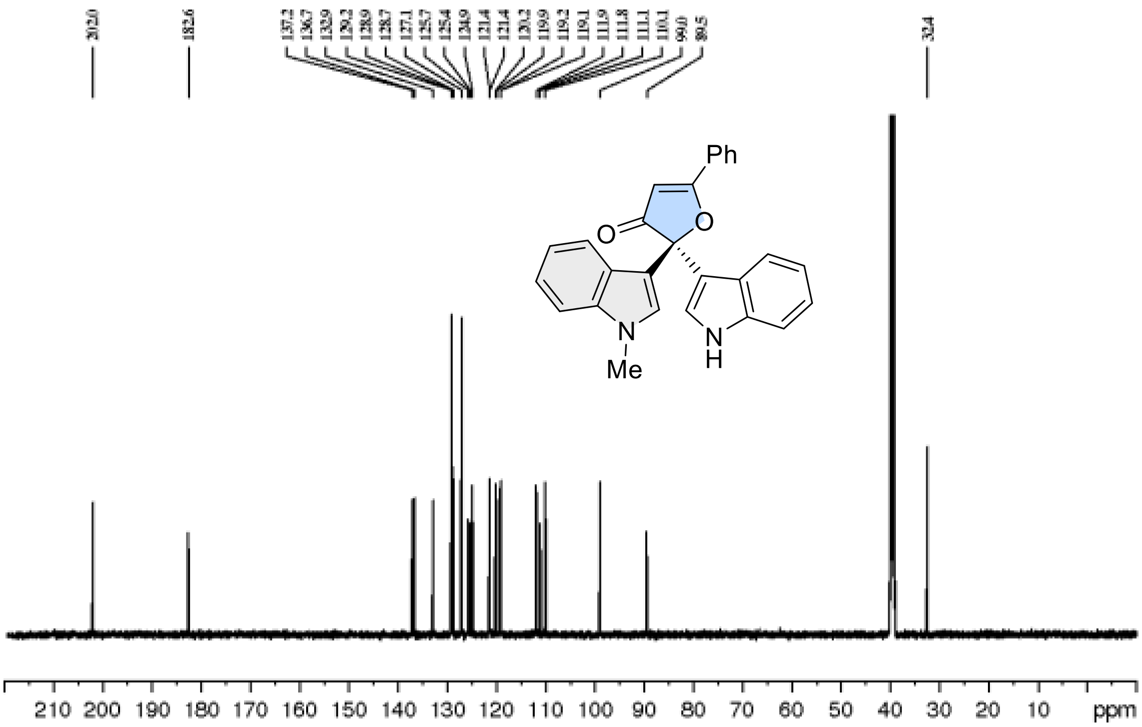

^{13}C NMR (126 MHz, DMSO-d_6) of (S)-**3Aa**

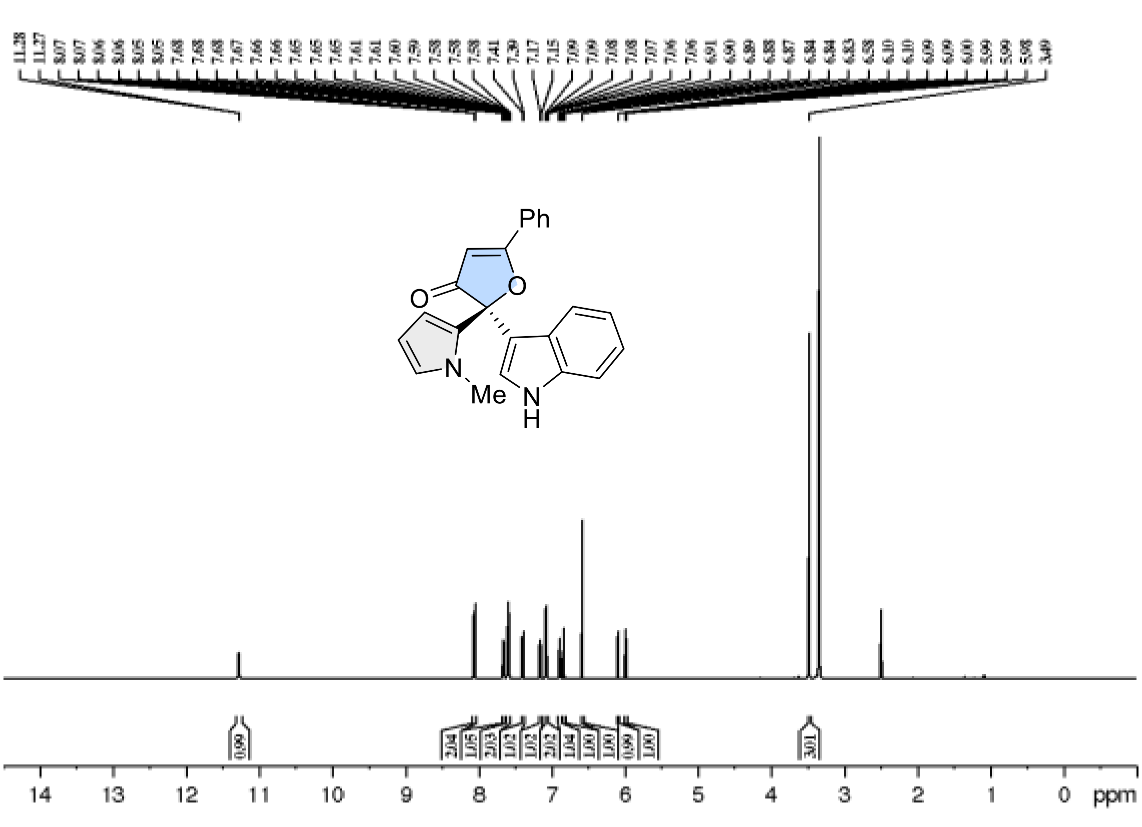

^{1}H NMR (500 MHz, DMSO-d_6) of (S)-**3Ba**

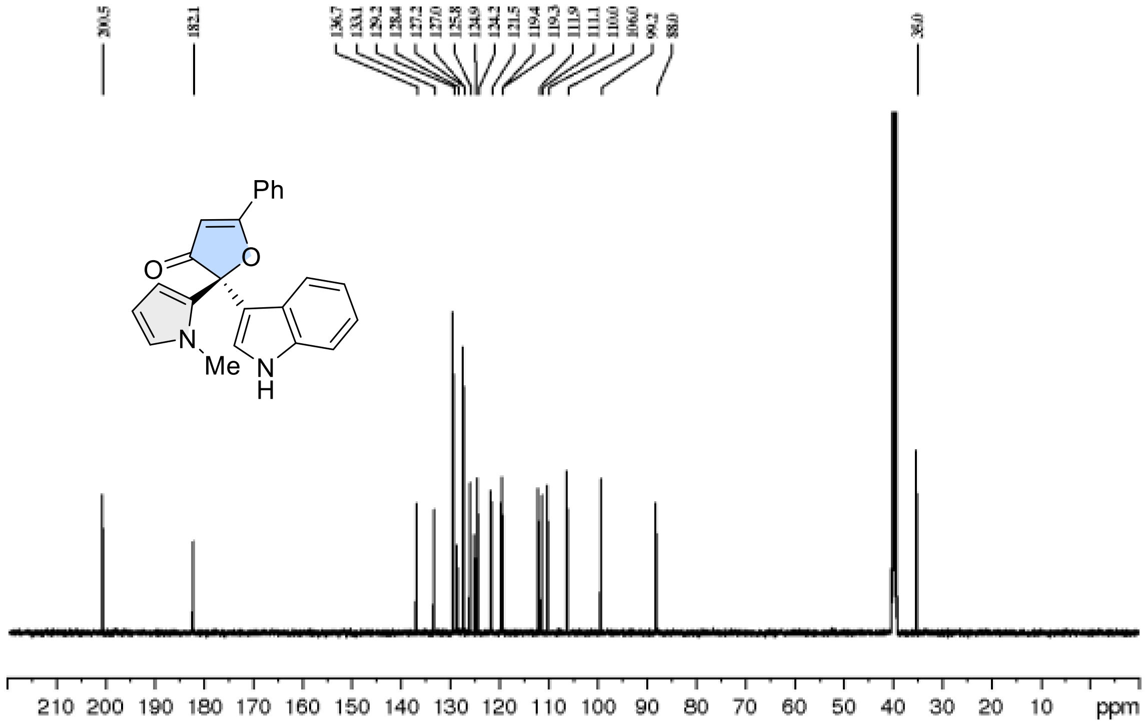

^{13}C NMR (126 MHz, DMSO-d_6) of (S)-**3Ba**

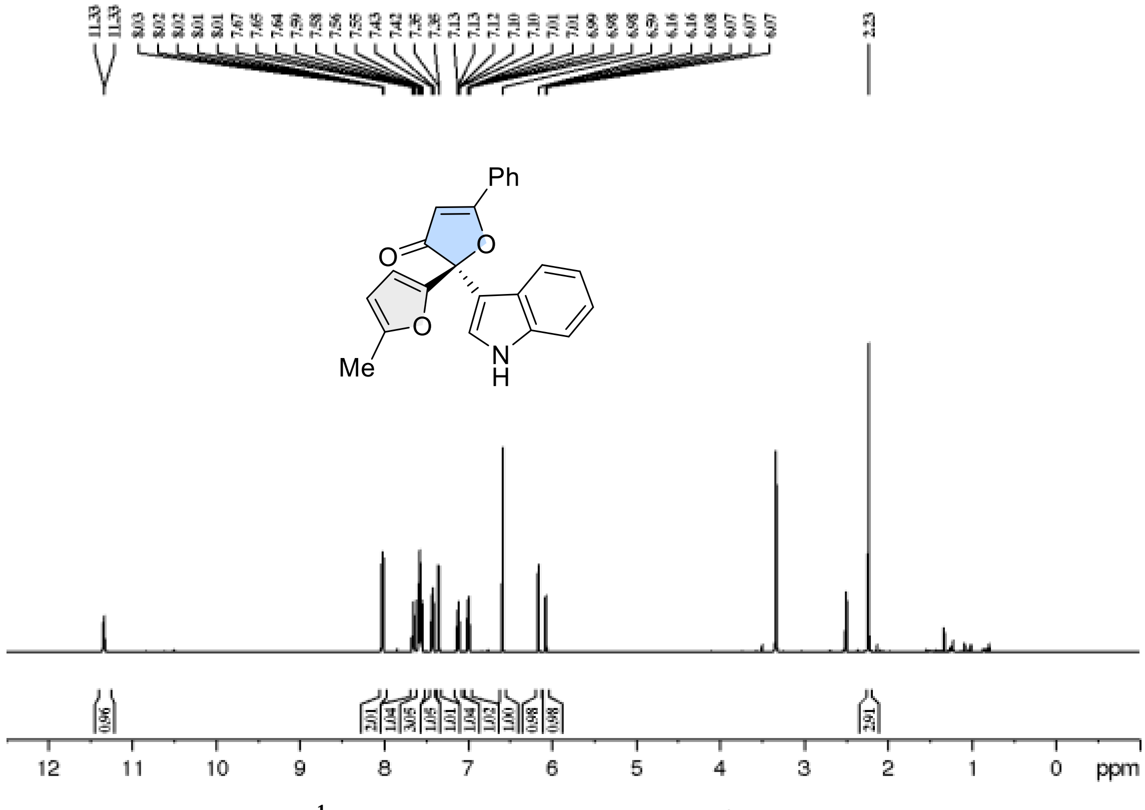

^{1}H NMR (500 MHz, DMSO-d_6) of (S)-**3Ca**

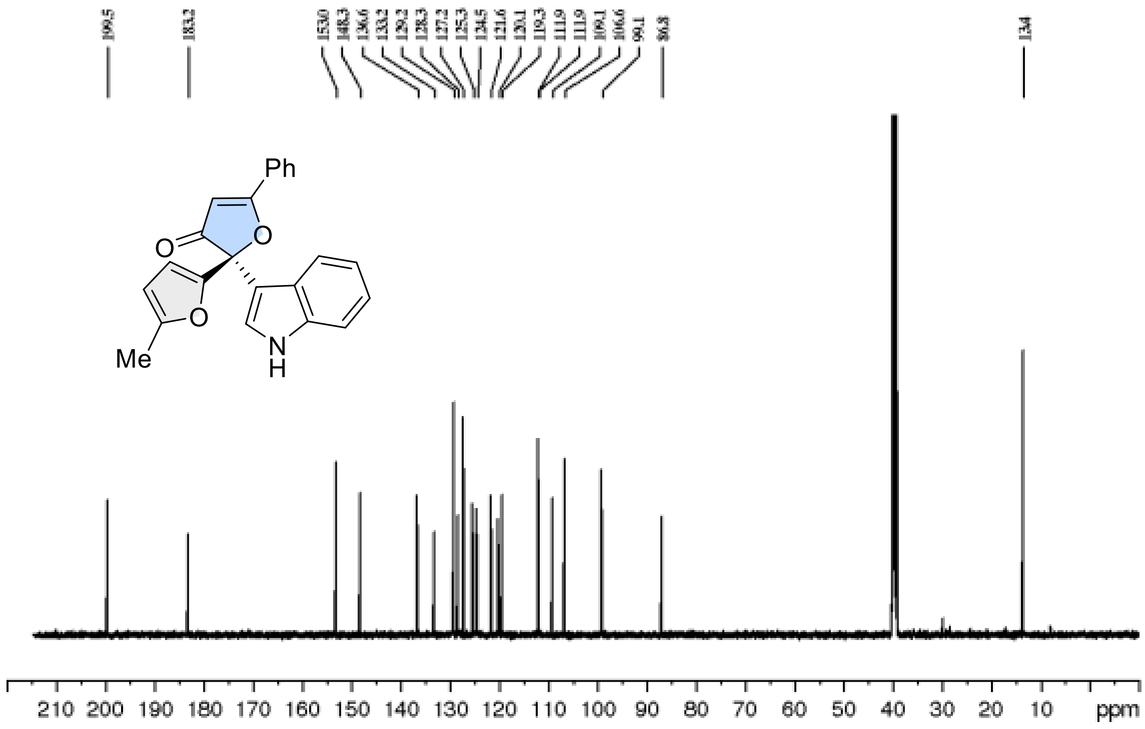

^{13}**C NMR** (126 MHz, DMSO-d_6) of (*S*)-**3Ca**

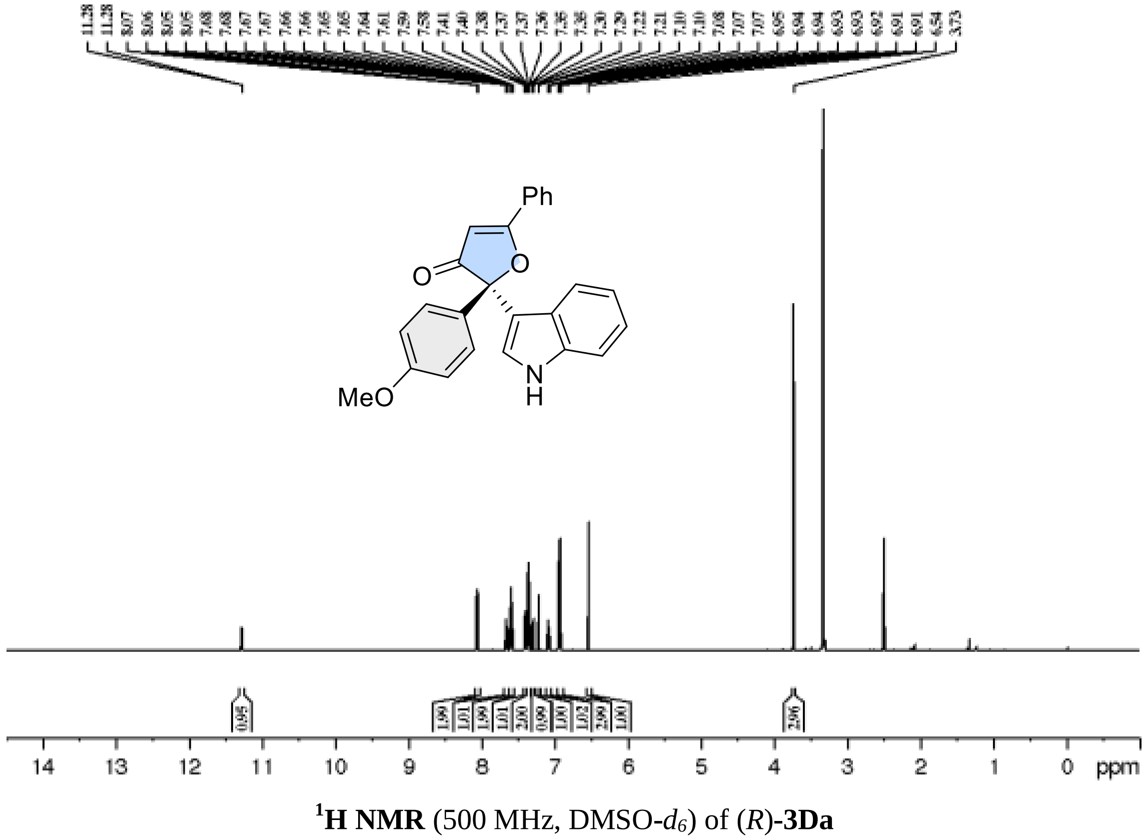

^{1}**H NMR** (500 MHz, DMSO-d_6) of (*R*)-**3Da**

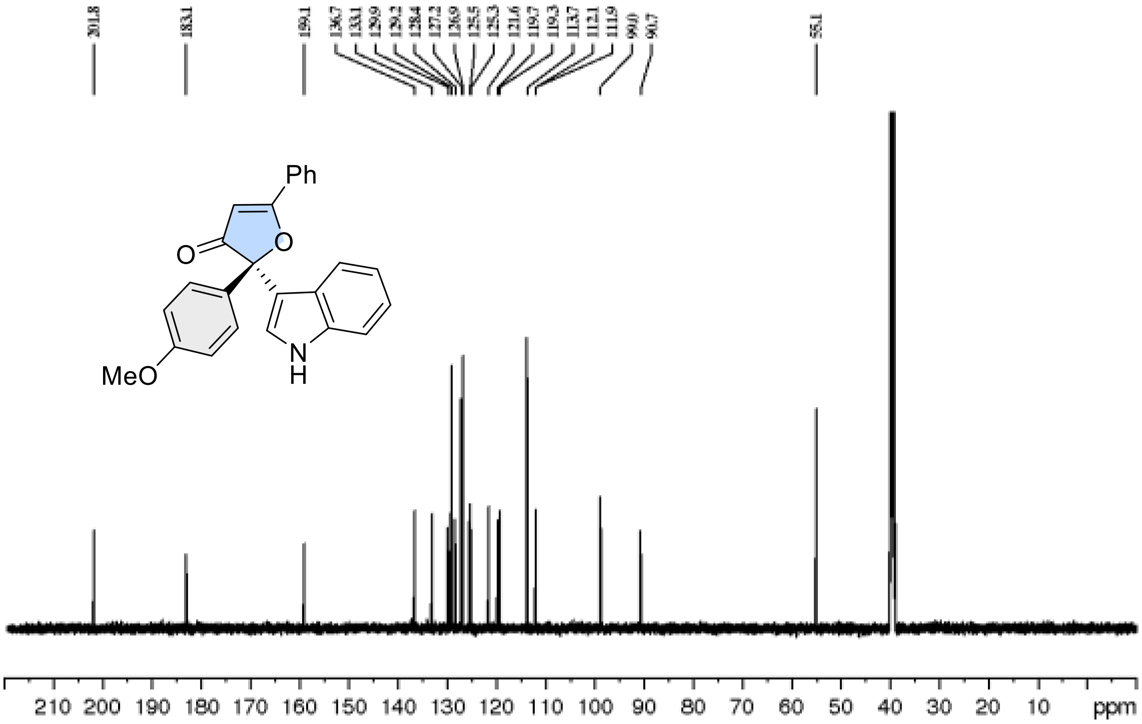

^{13}C NMR (126 MHz, DMSO-d_6) of (*R*)-**3Da**

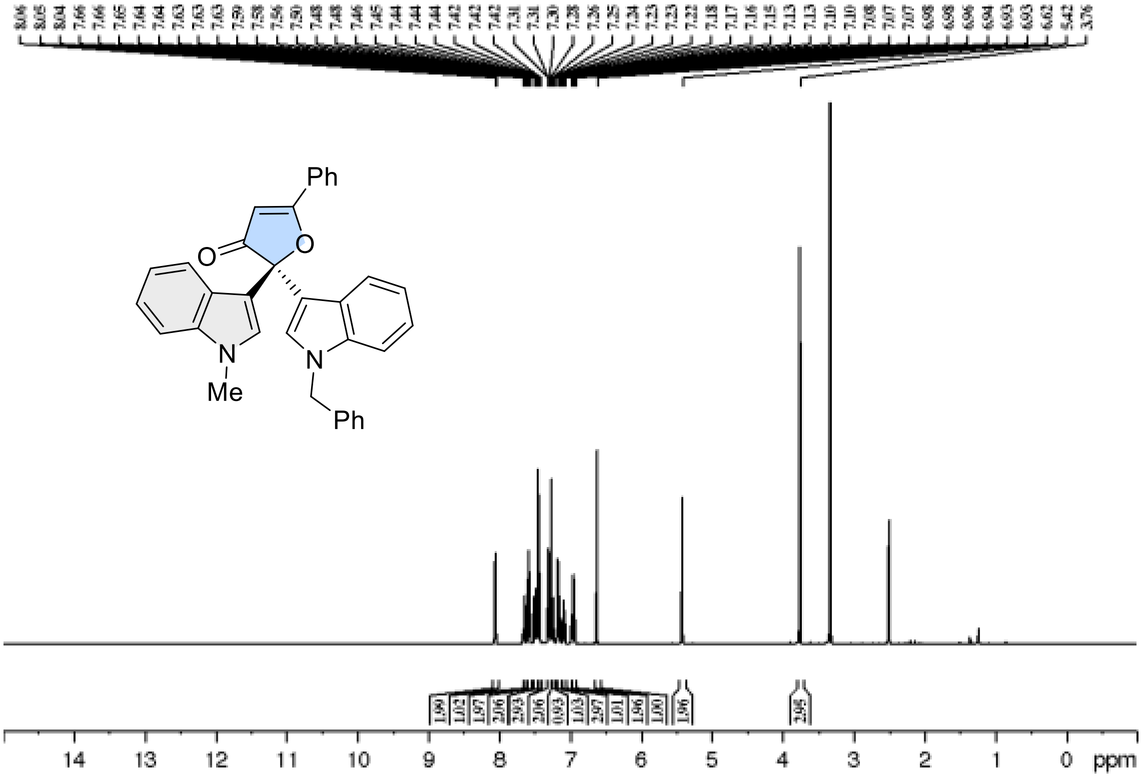

^{1}H NMR (500 MHz, DMSO-d_6) of (*R*)-**3Ab**

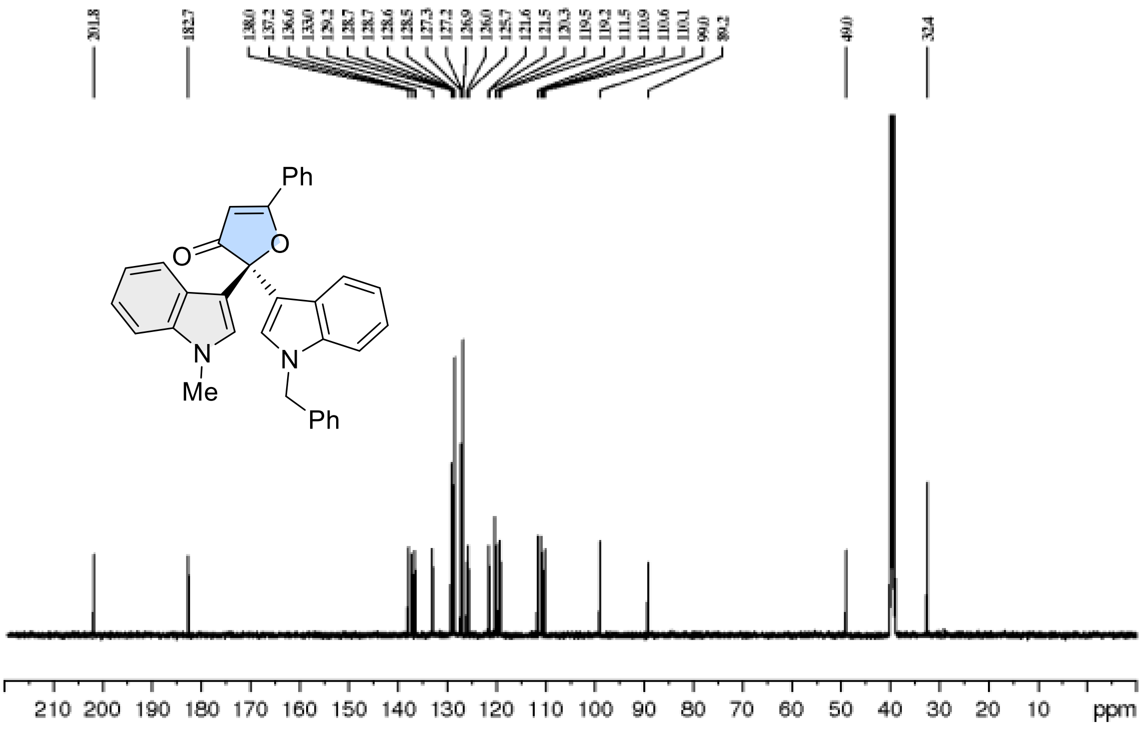

^{13}**C NMR** (126 MHz, DMSO-d_6) of (R)-**3Ab**

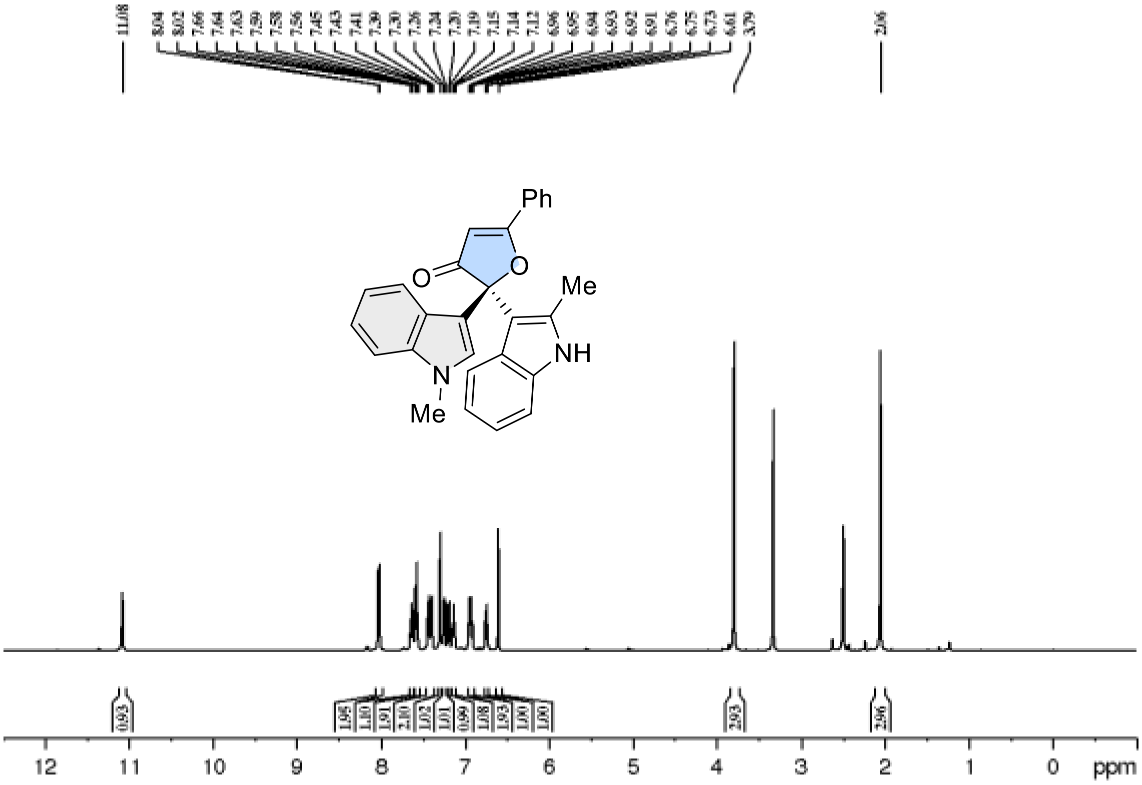

^{1}**H NMR** (500 MHz, DMSO-d_6) of (R)-**3Ad**

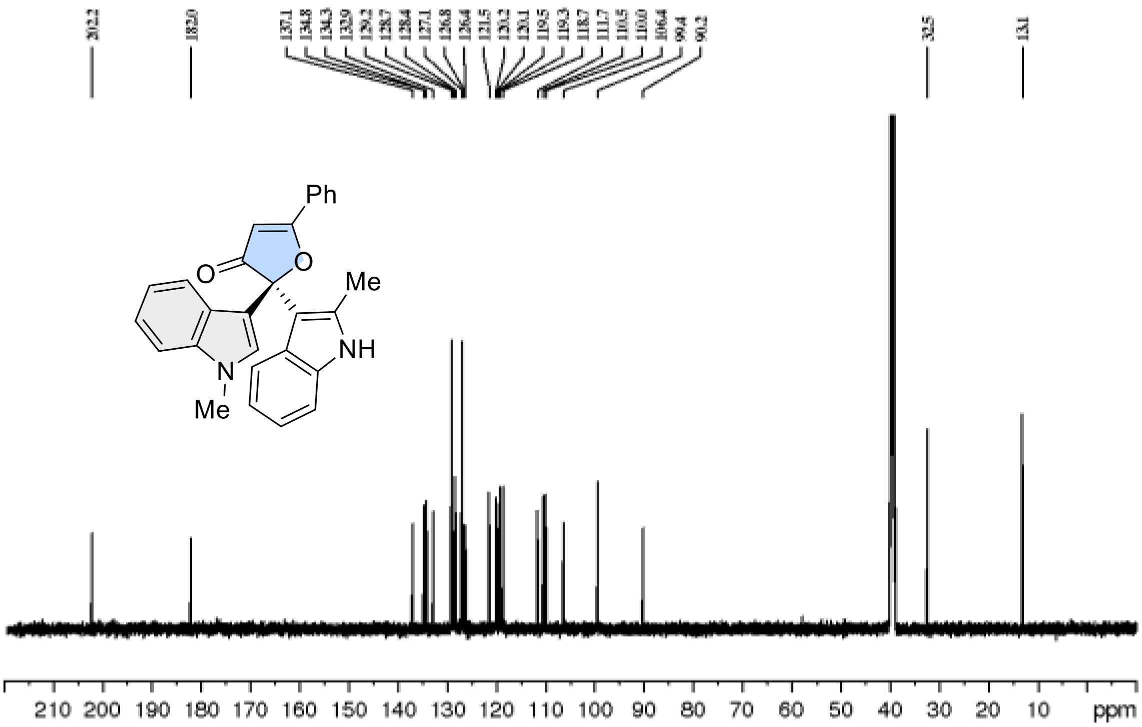

^{13}C NMR (126 MHz, DMSO-d_6) of (R)-**3Ad**

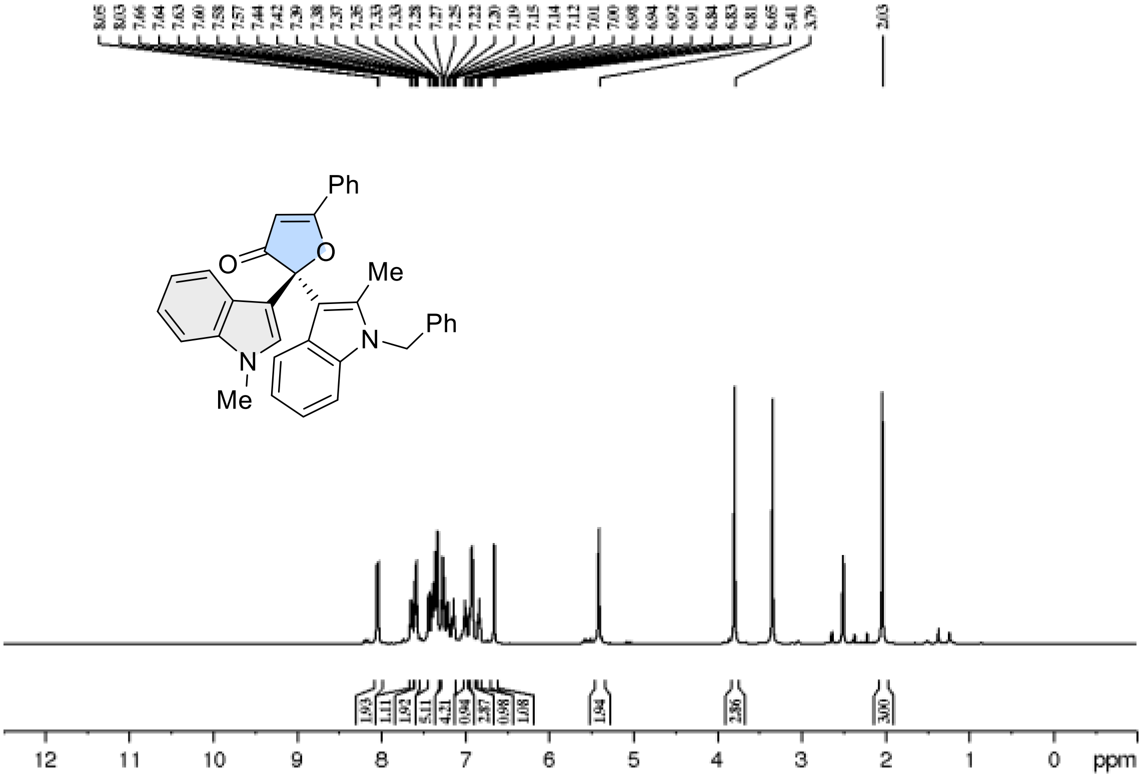

^{1}H NMR (500 MHz, DMSO-d_6) of (R)-**3Ae**

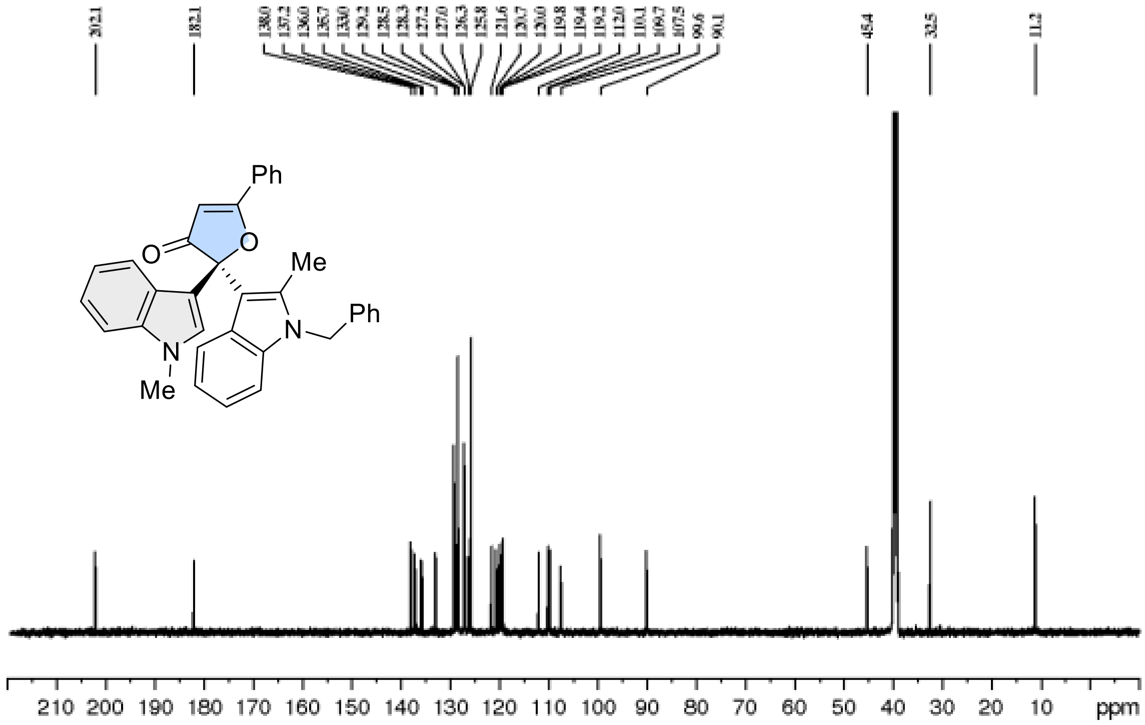

^{13}C NMR (126 MHz, DMSO-d_6) of (*R*)-**3Ae**

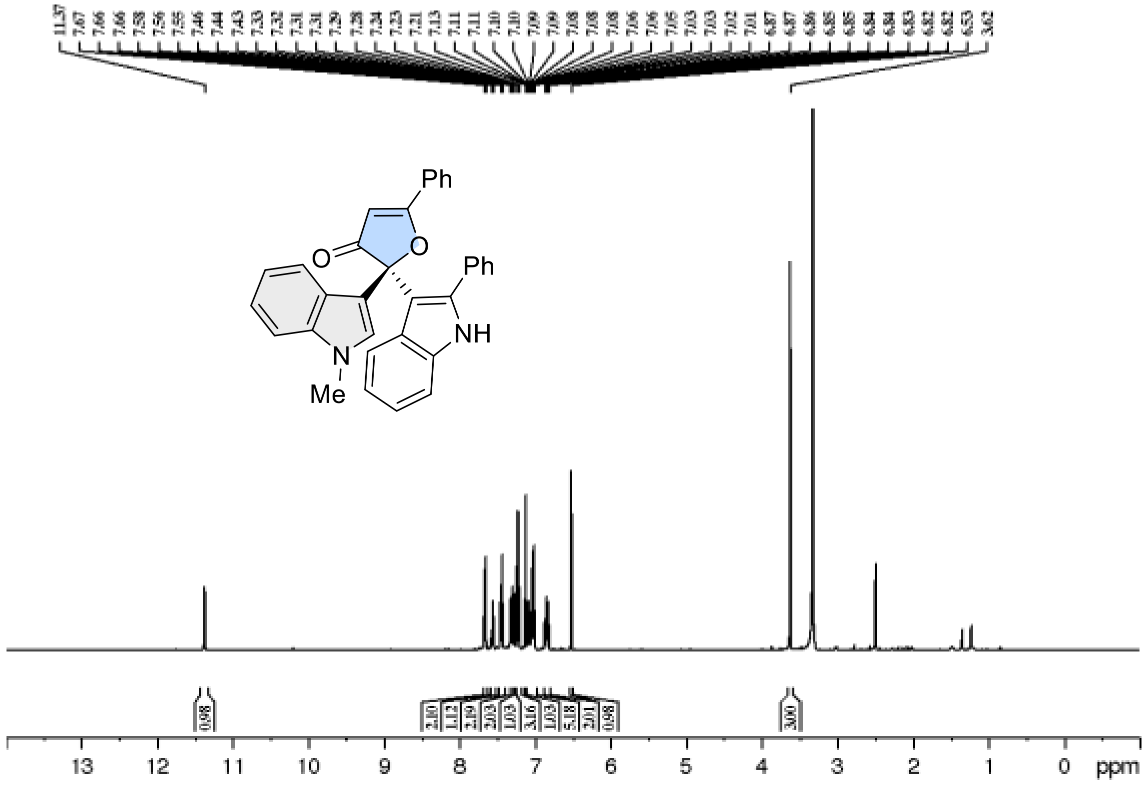

^{1}H NMR (300 MHz, DMSO-d_6) of (*R*)-**3Af**

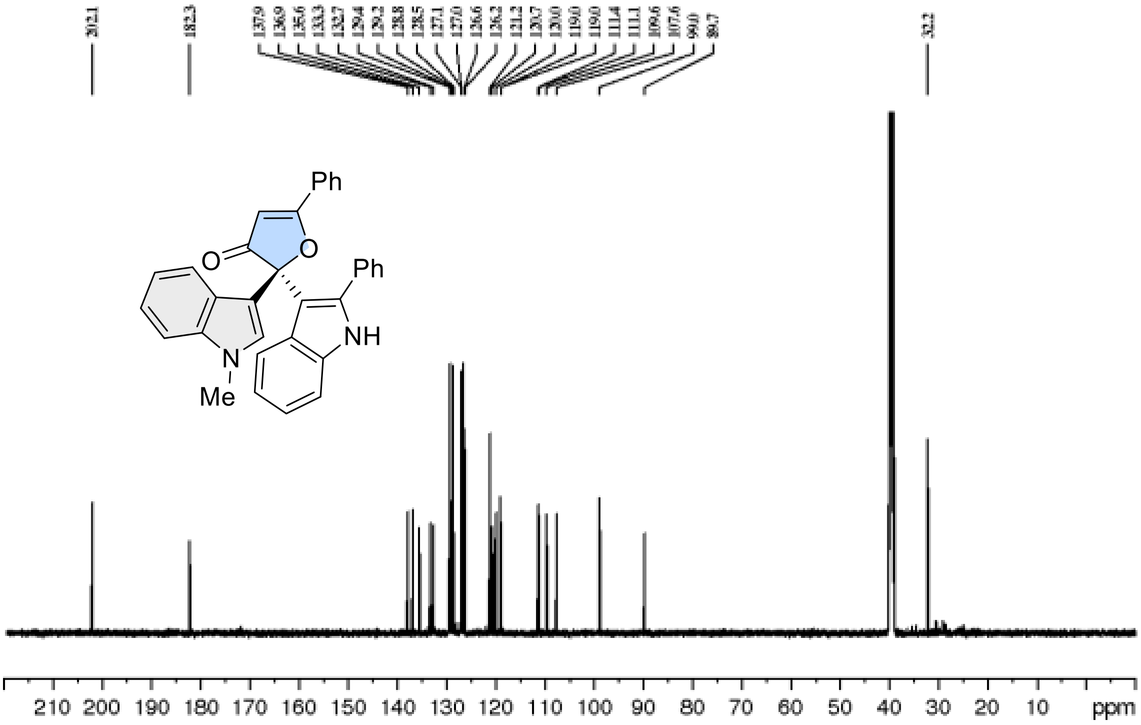

^{13}**C NMR** (126 MHz, DMSO-d_6) of (*R*)-**3Af**

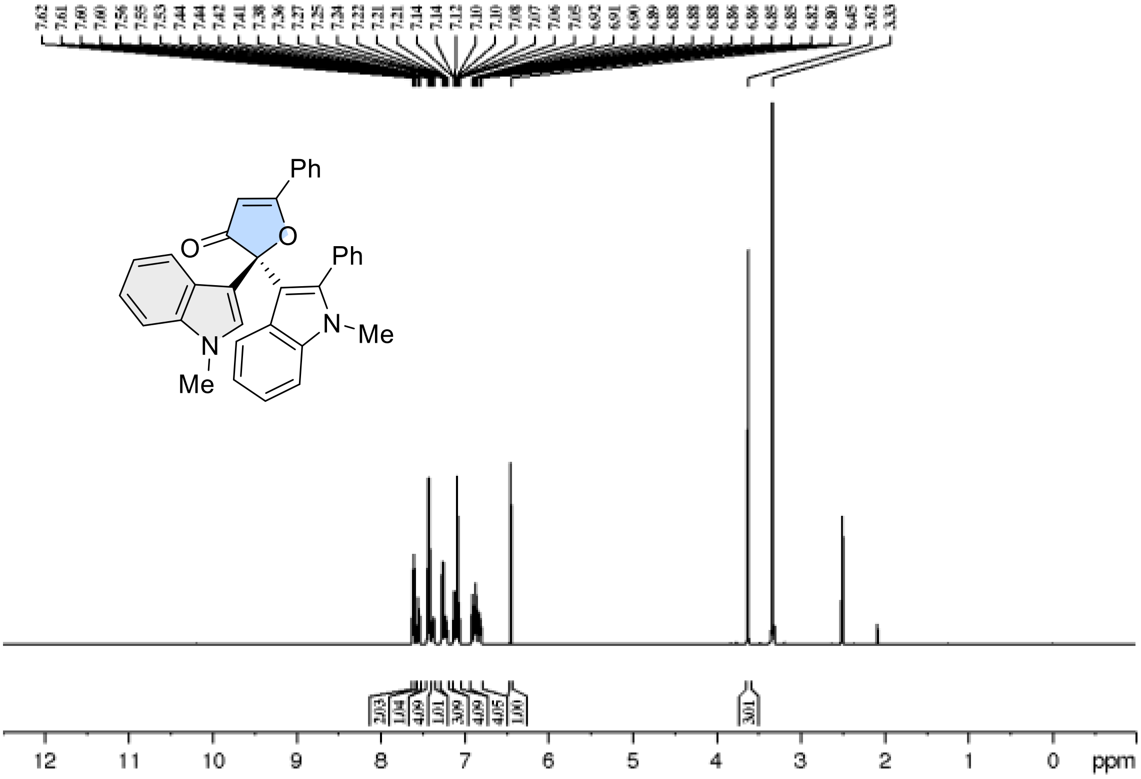

^{1}**H NMR** (500 MHz, DMSO-d_6) of (*R*)-**3Ag**

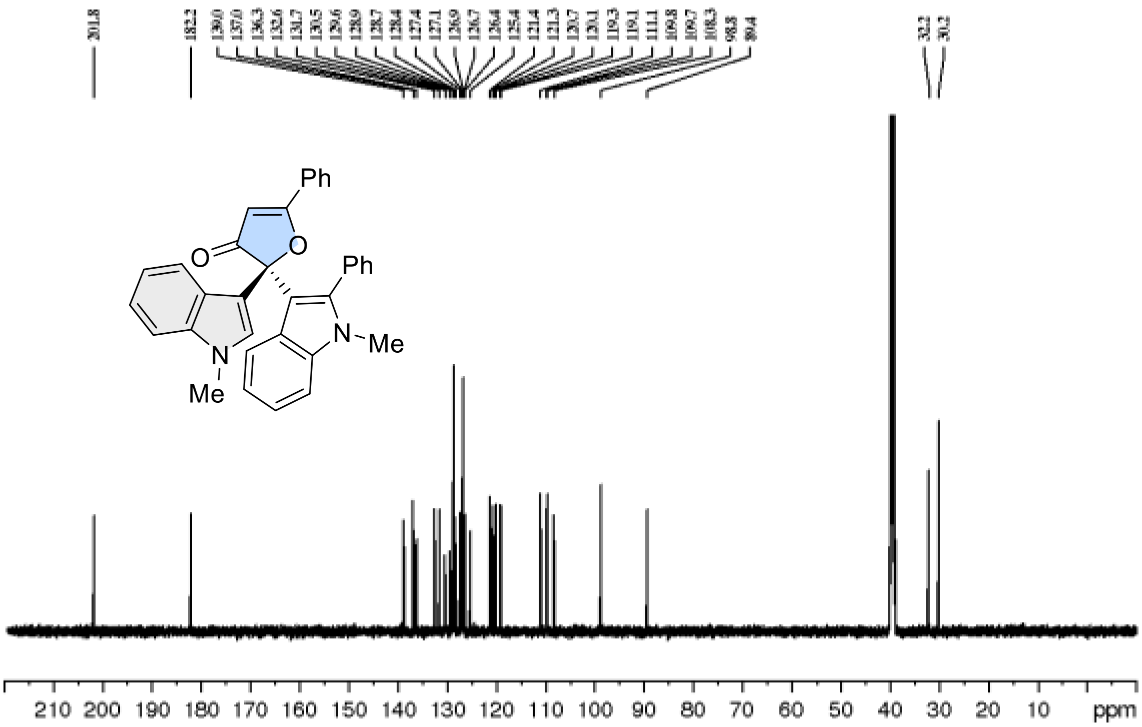

^{13}C NMR (126 MHz, DMSO-d_6) of (*R*)-**3Ag**

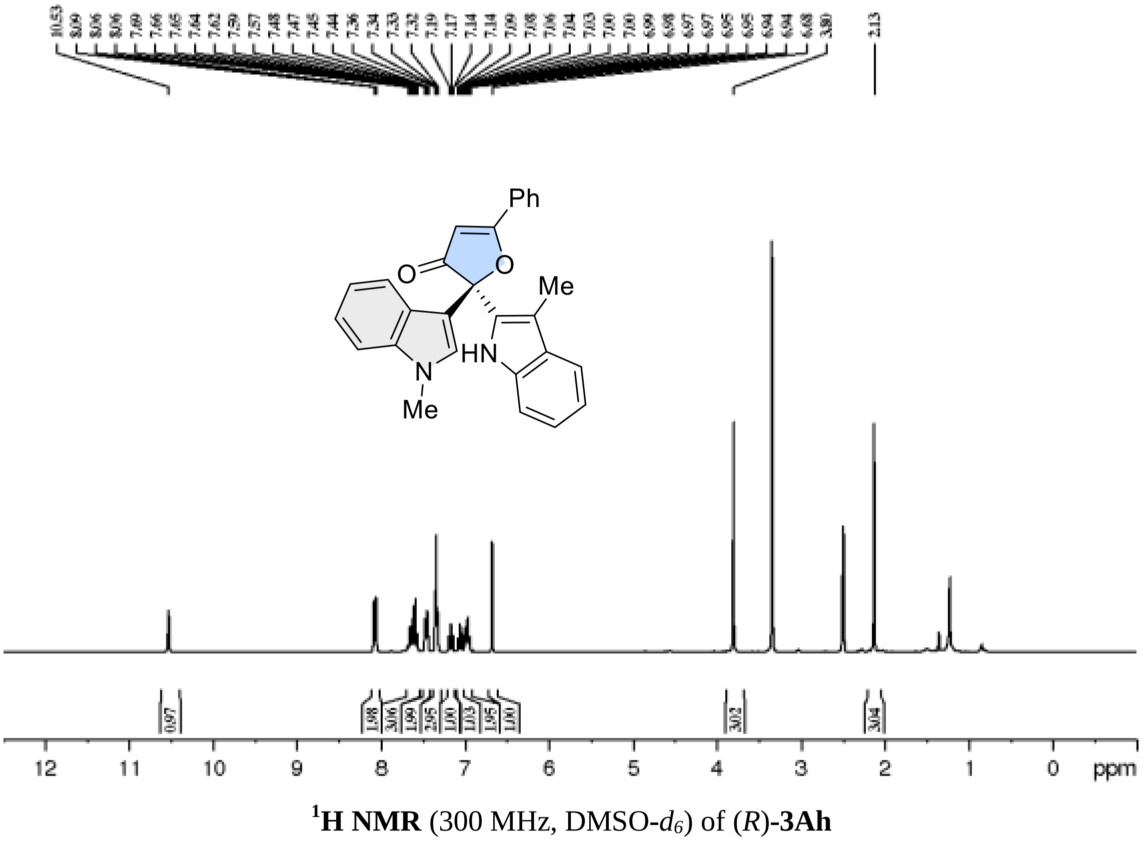

^{1}H NMR (300 MHz, DMSO-d_6) of (*R*)-**3Ah**

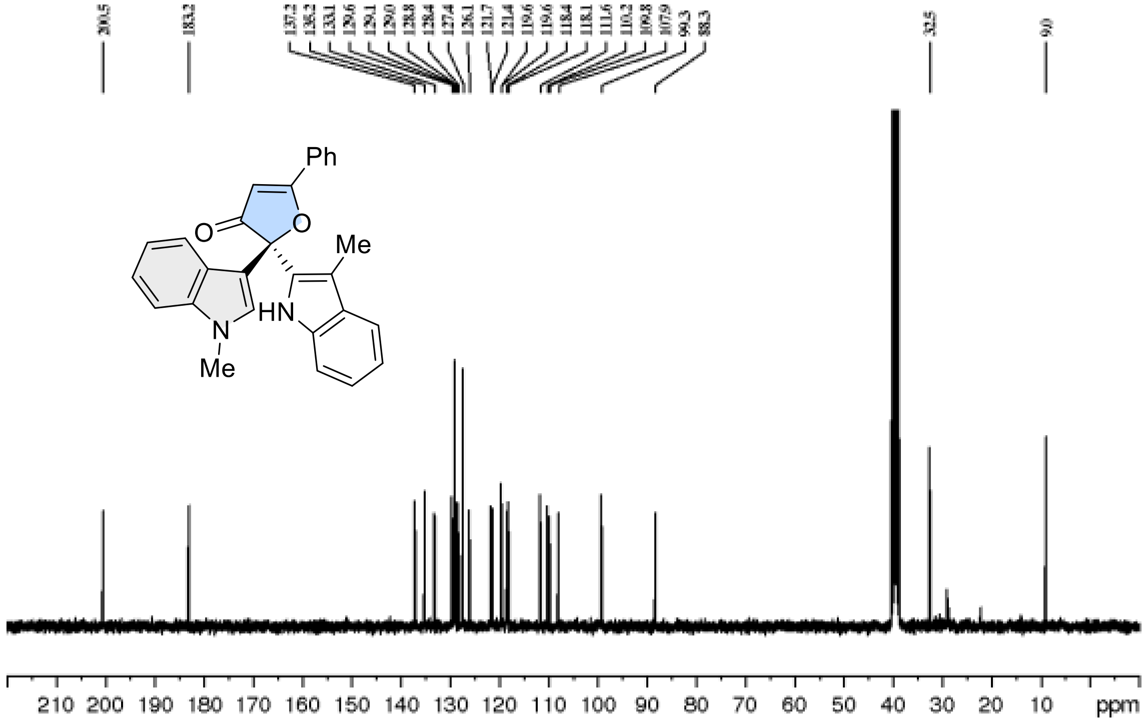

^{13}**C NMR** (75.5 MHz, DMSO-d_6) of (R)-**3Ah**

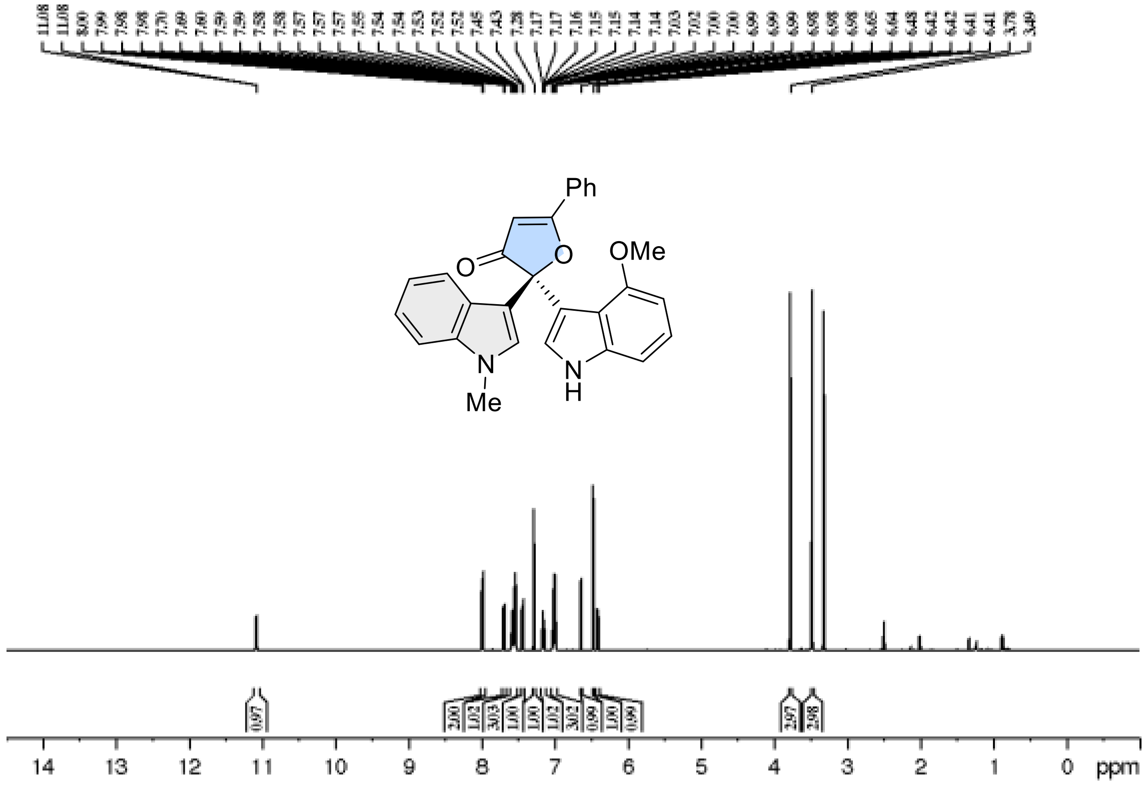

^{1}**H NMR** (500 MHz, DMSO-d_6) of (S)-**3Ai**

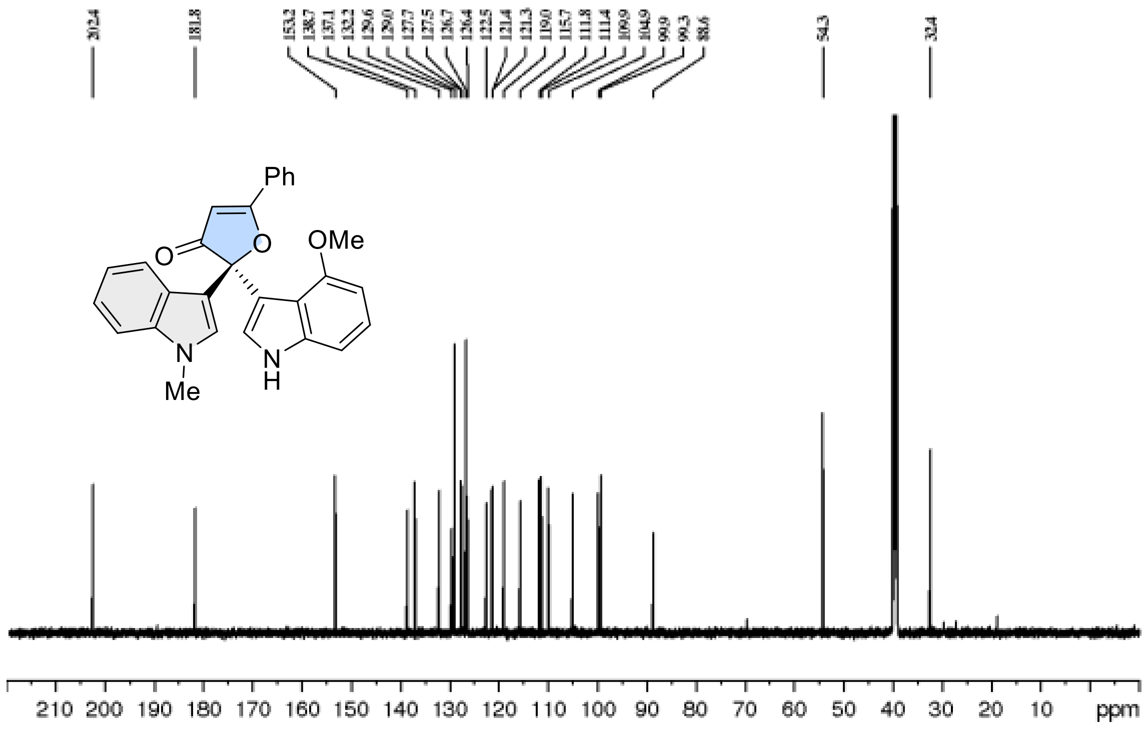

^{13}C NMR (126 MHz, DMSO-d_6) of (*S*)-**3Ai**

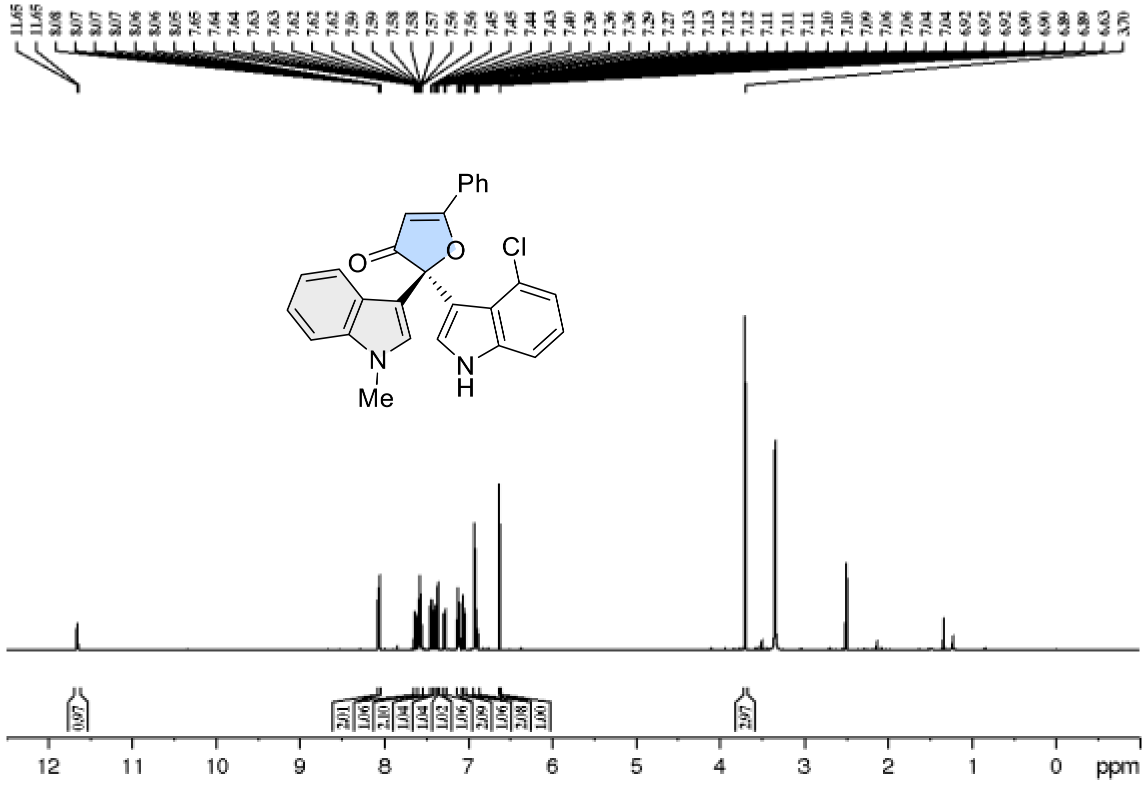

^{1}H NMR (500 MHz, DMSO-d_6) of (*S*)-**3Aj**

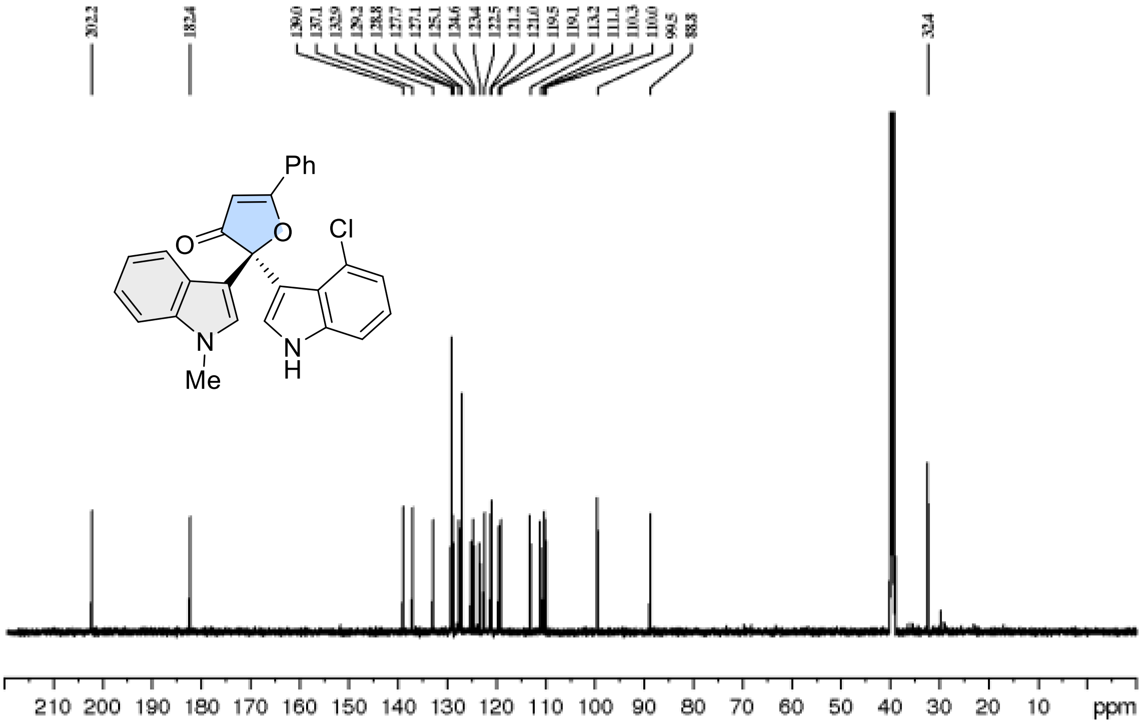

^{13}C NMR (126 MHz, DMSO-d_6) of (*S*)-**3Aj**

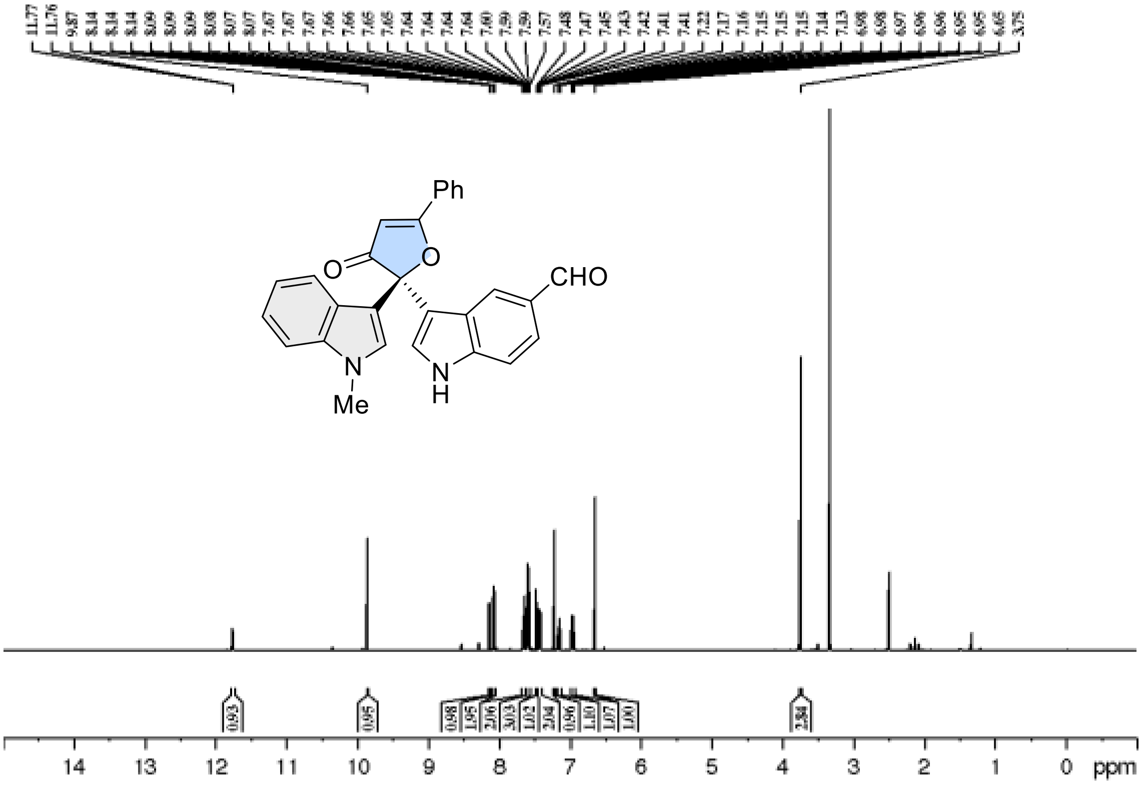

^{1}H NMR (500 MHz, DMSO-d_6) of (*S*)-**3Ak**

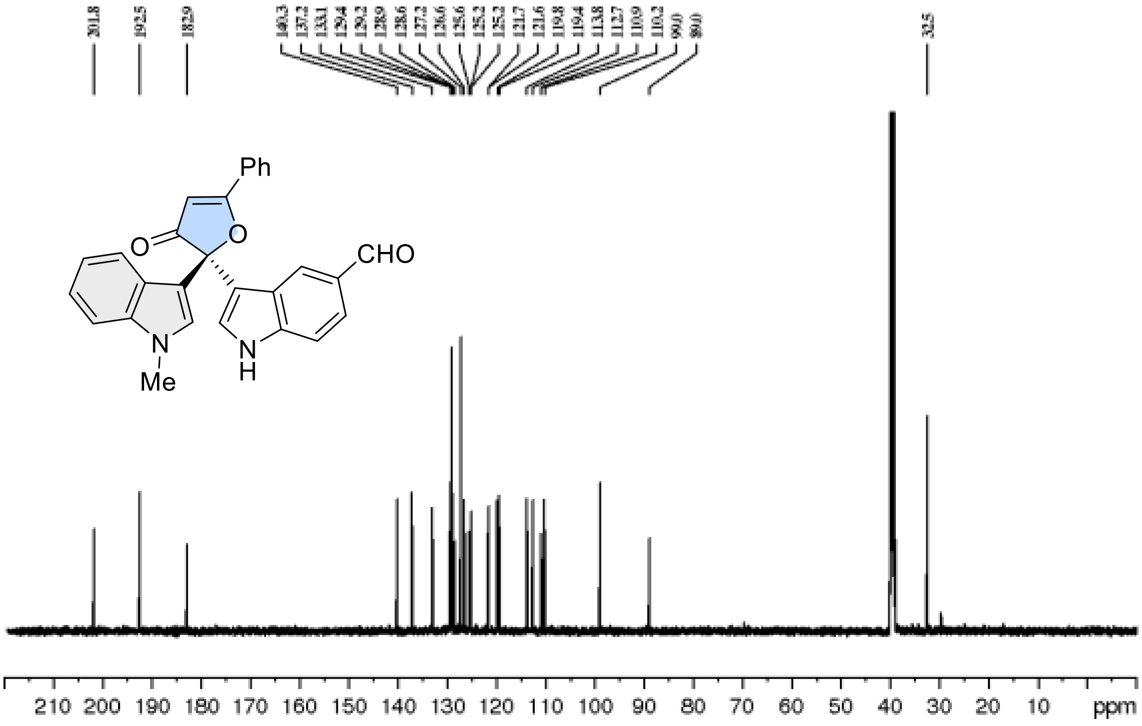

^{13}**C NMR** (126 MHz, DMSO-d_6) of (*S*)-**3Ak**

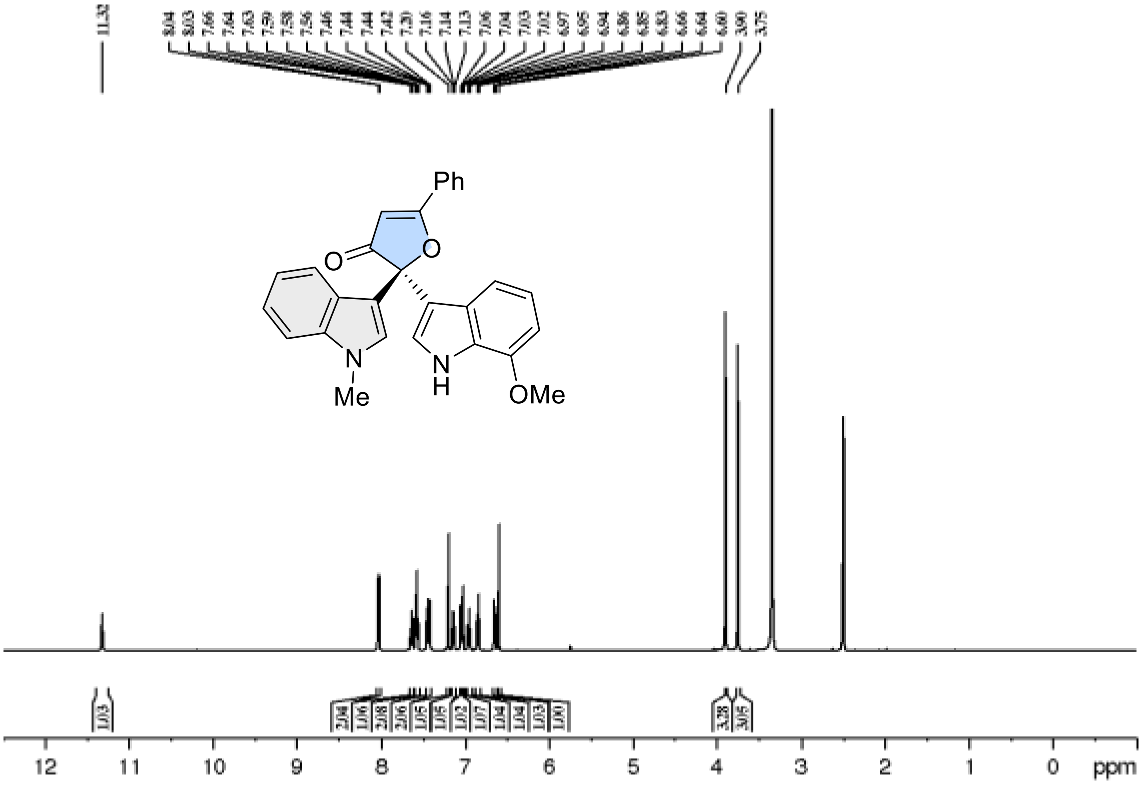

^{1}**H NMR** (500 MHz, DMSO-d_6) of (*S*)-**3Al**

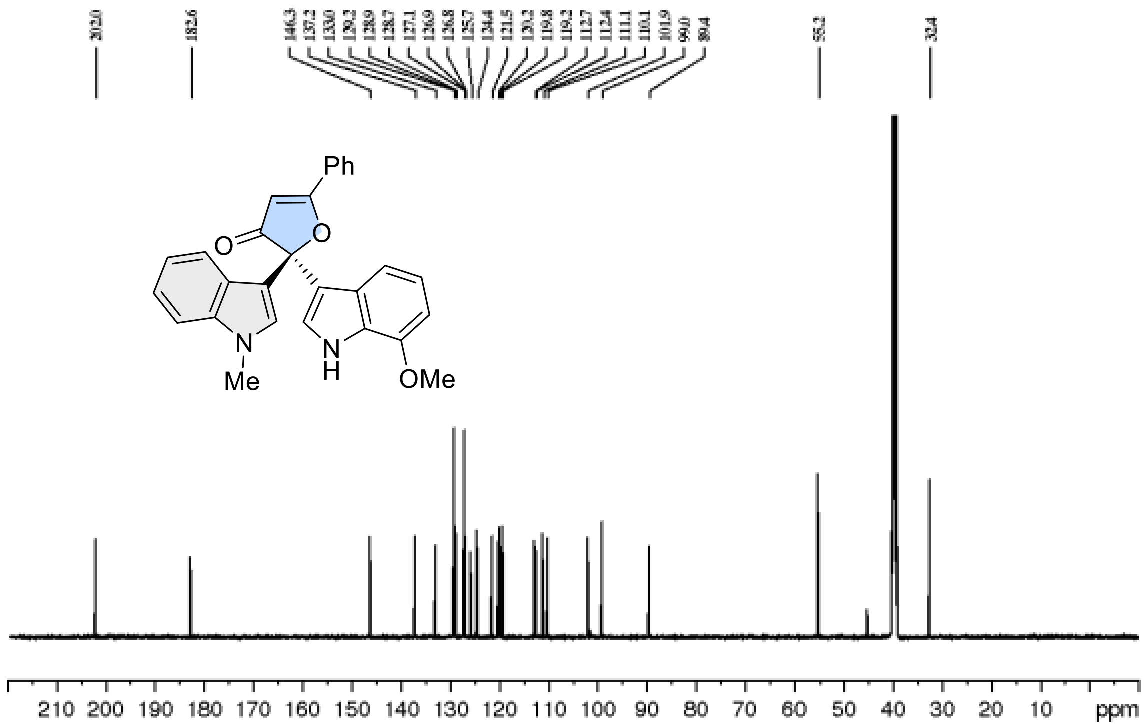

^{13}C NMR (126 MHz, DMSO-d_6) of (S)-**3Al**

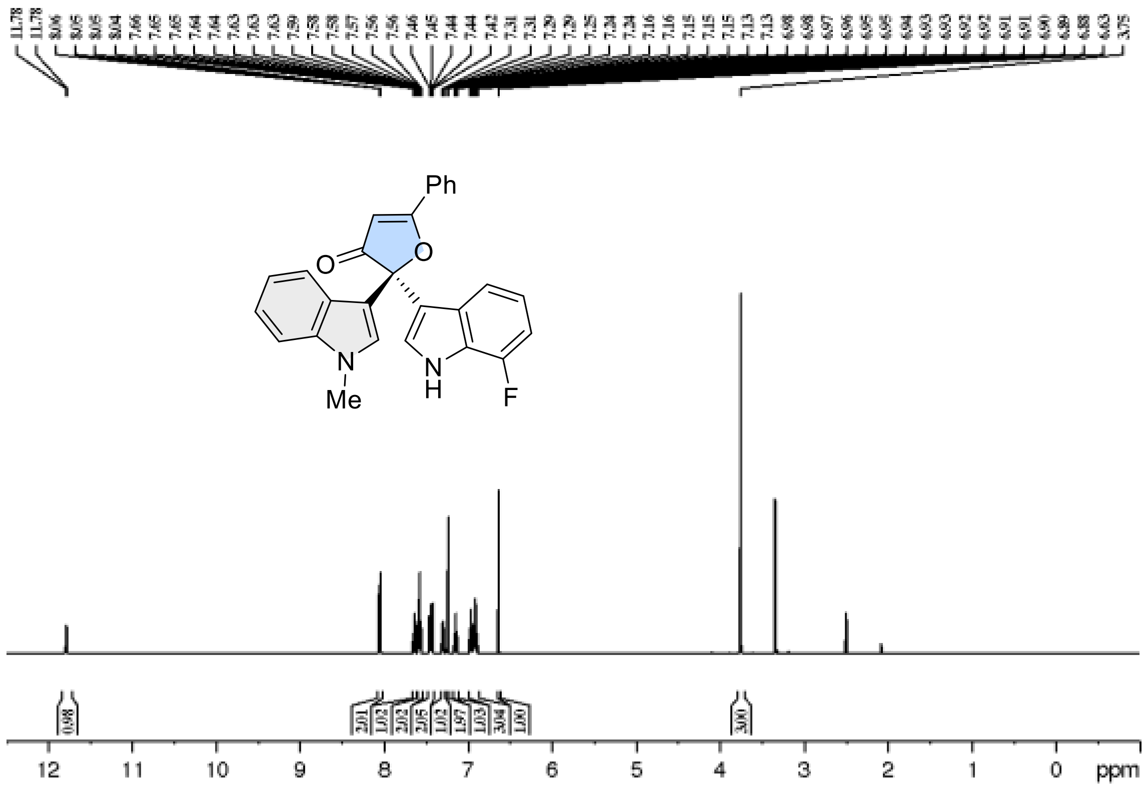

^{1}H NMR (500 MHz, DMSO-d_6) of (S)-**3Am**

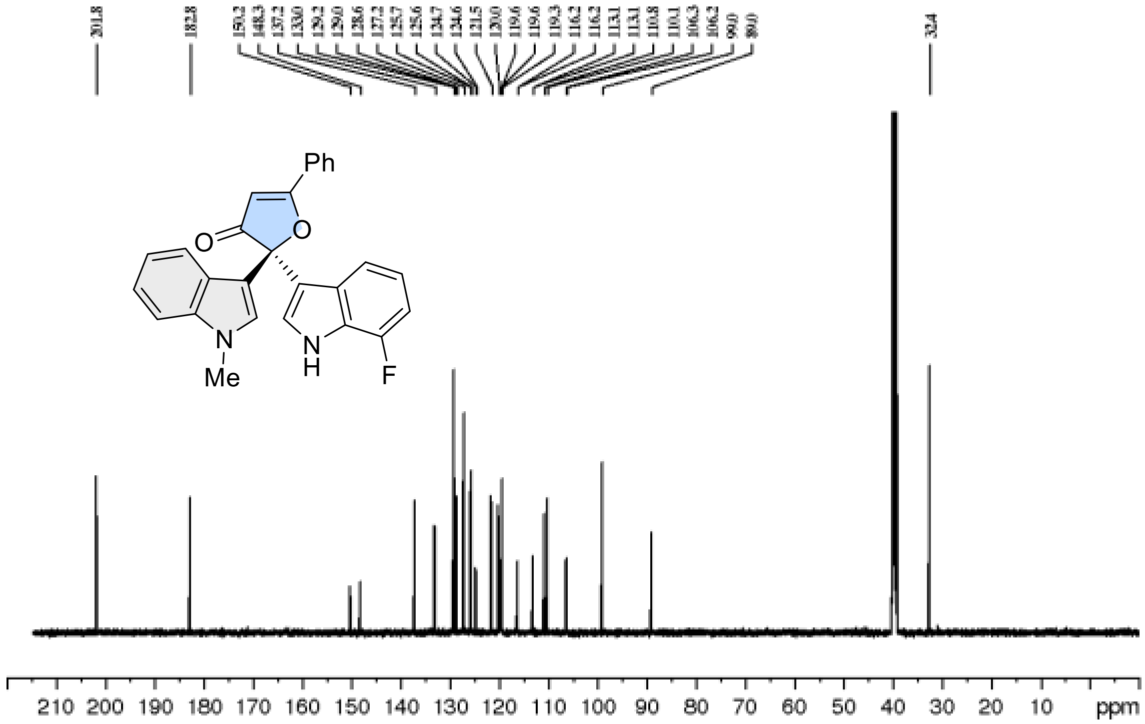

^{13}C NMR (126 MHz, DMSO-d_6) of (S)-**3Am**

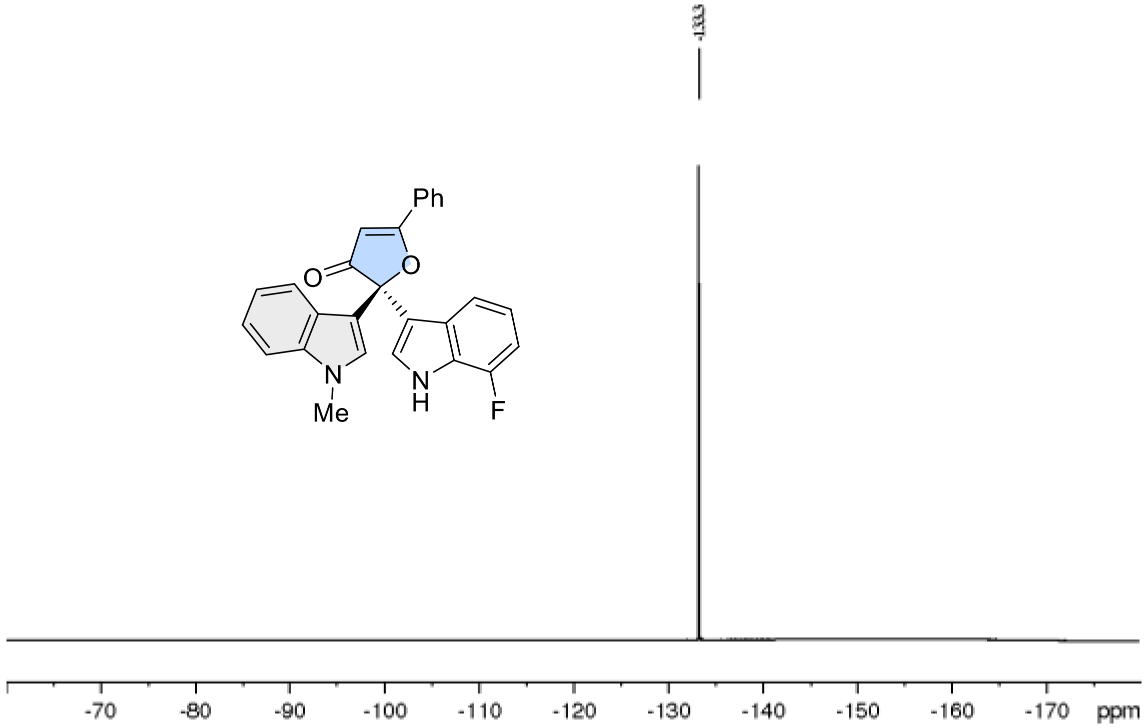

^{19}F NMR (471 MHz, DMSO-d_6) of (S)-**3Am**

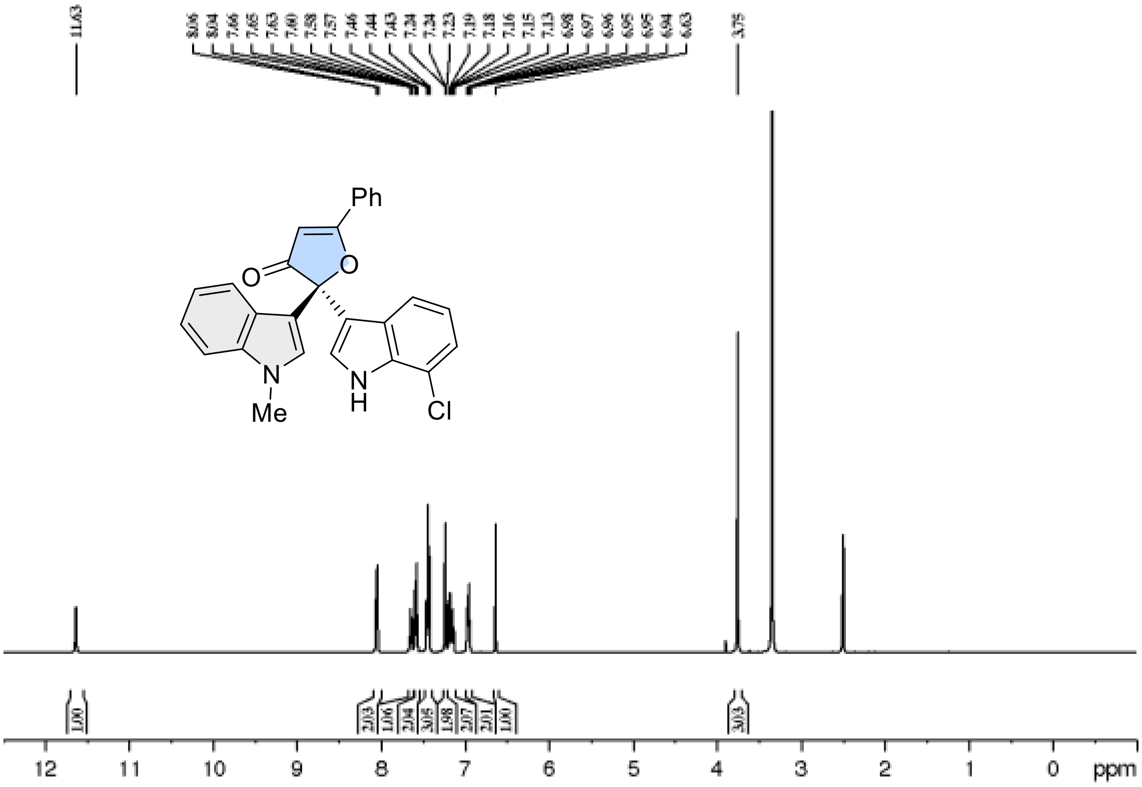

^{1}H NMR (500 MHz, DMSO-d_6) of (*S*)-**3An**

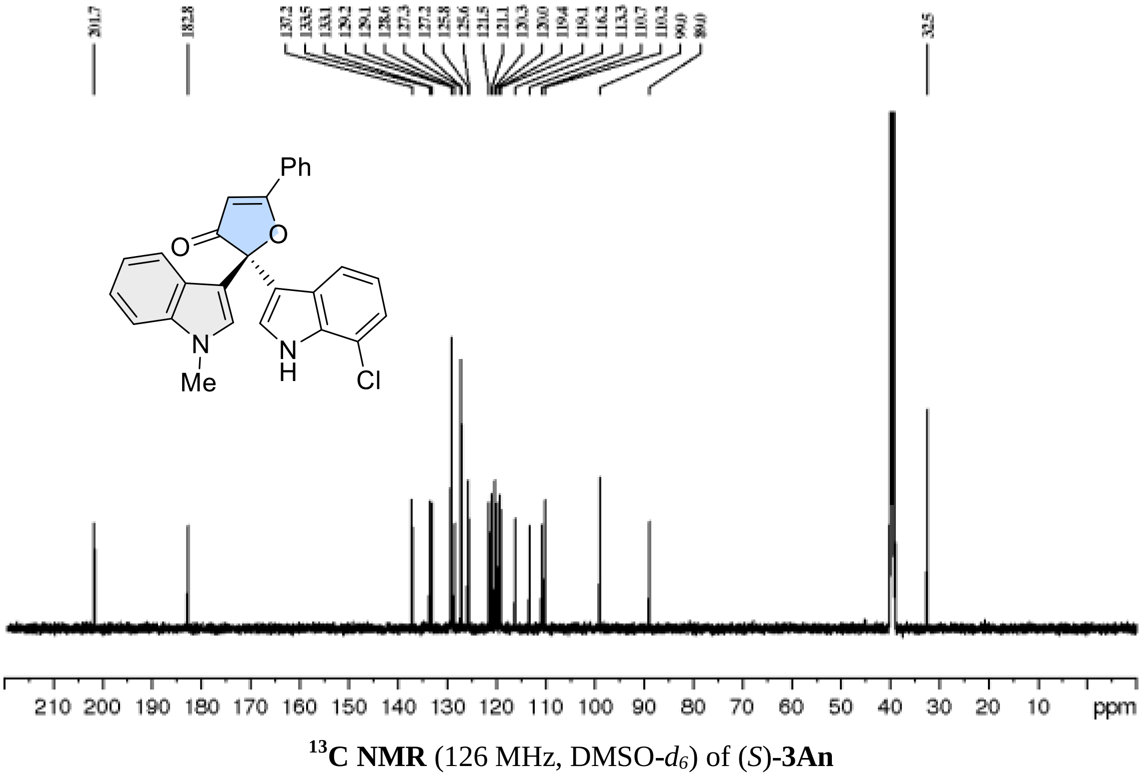

^{13}C NMR (126 MHz, DMSO-d_6) of (*S*)-**3An**

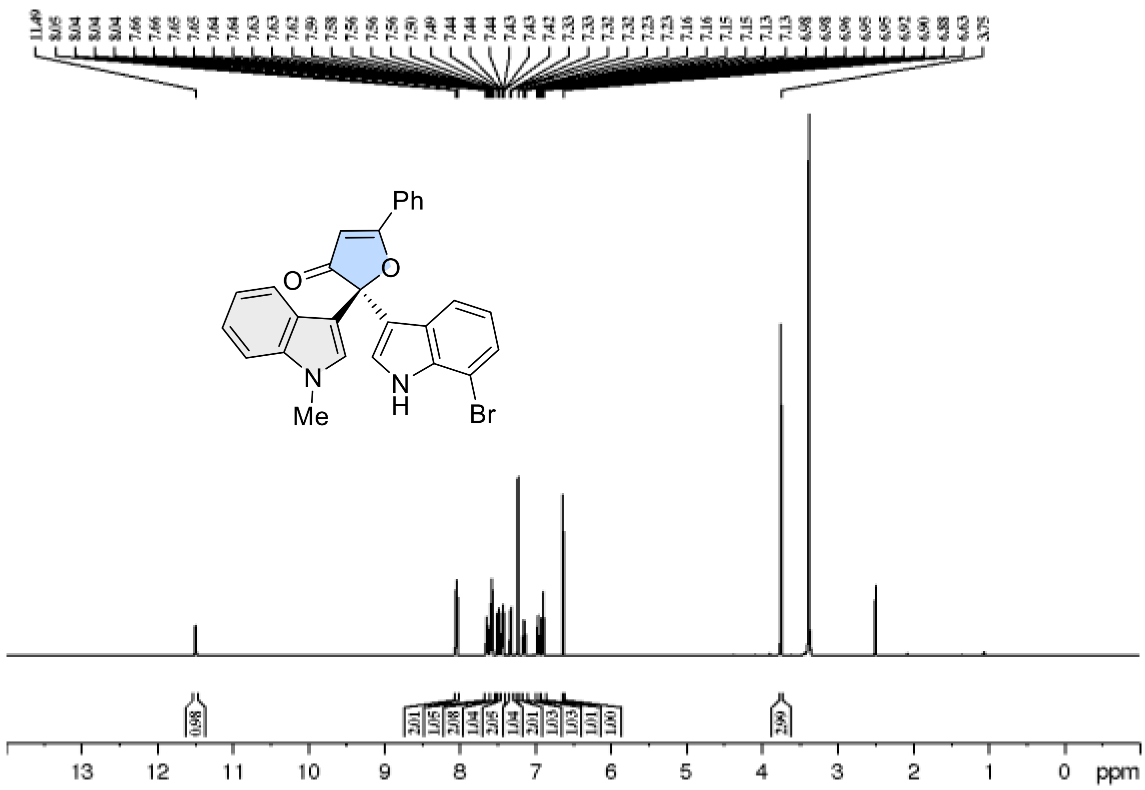

^{1}H NMR (500 MHz, DMSO-d_6) of (S)-**3Ao**

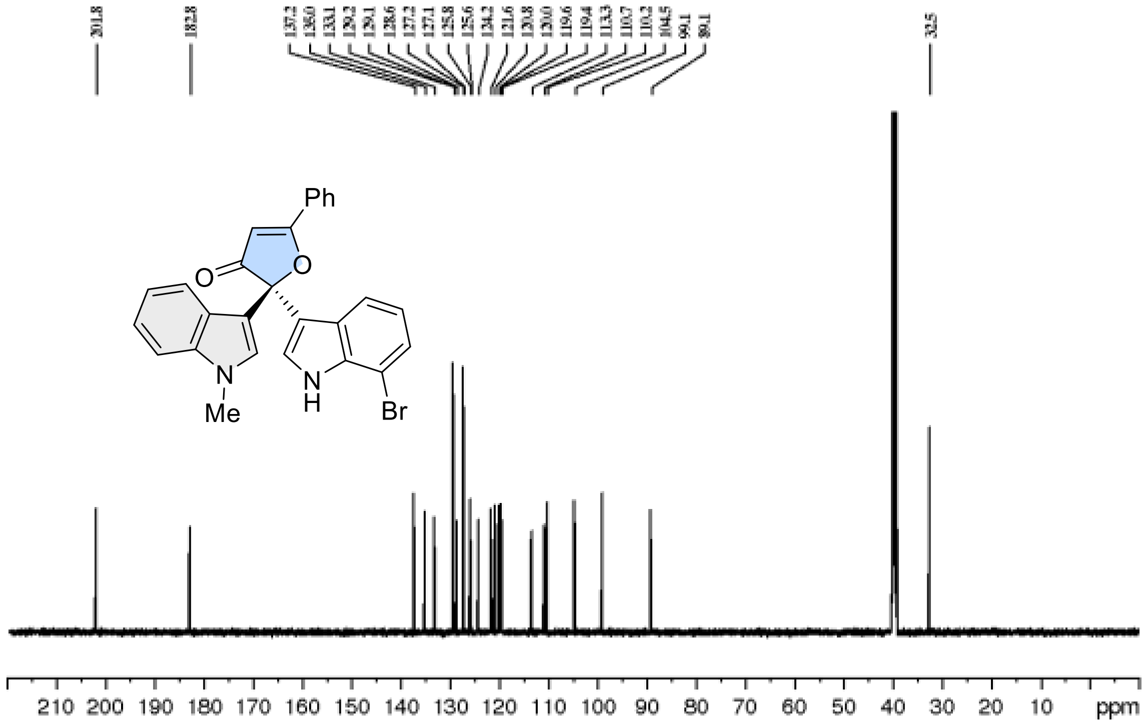

^{13}C NMR (126 MHz, DMSO-d_6) of (S)-**3Ao**

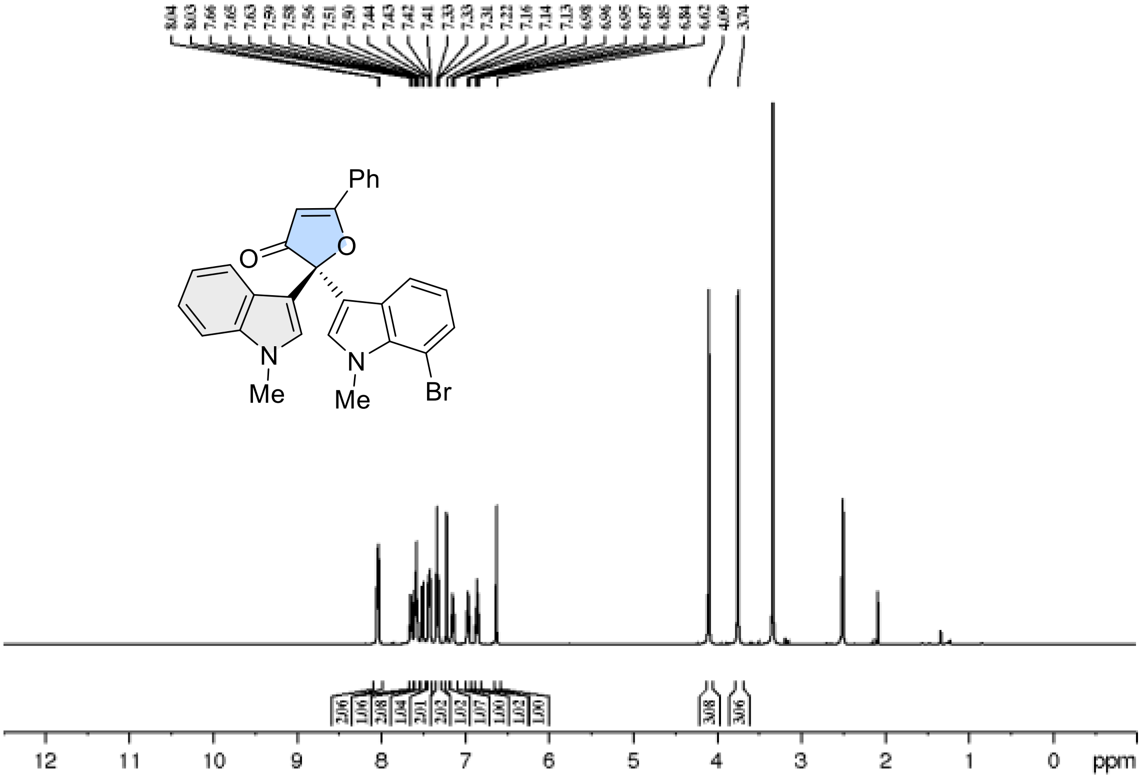

^{1}H NMR (500 MHz, DMSO-d_6) of (*R*)-**3Ap**

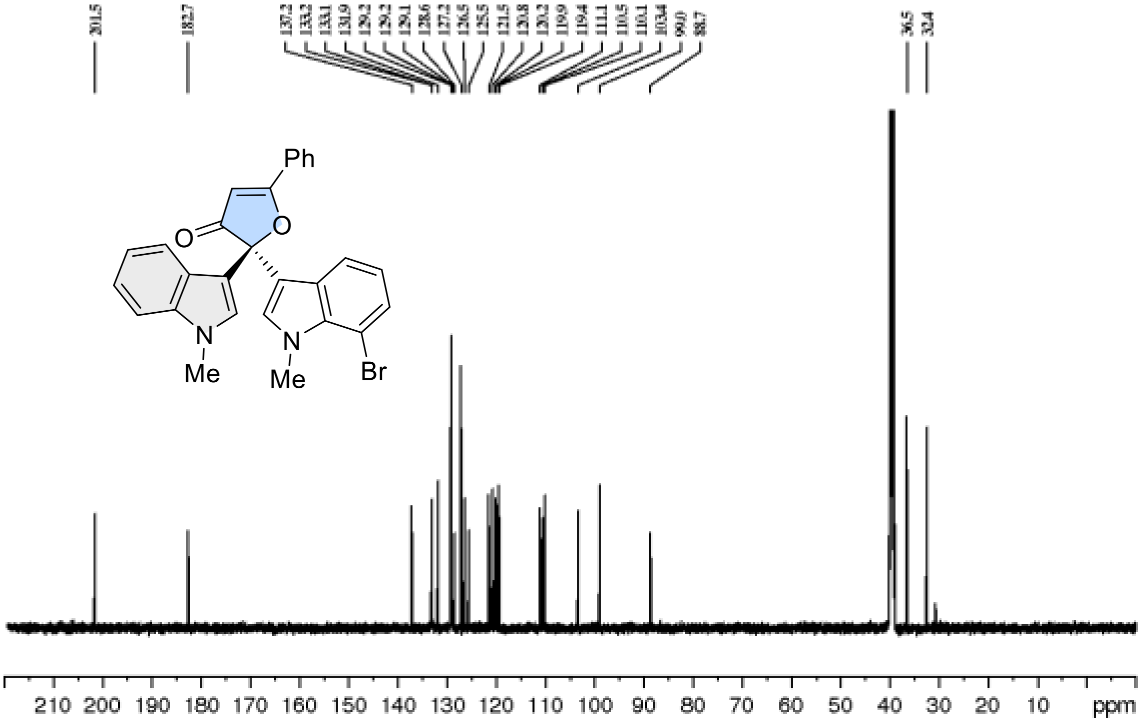

^{13}C NMR (126 MHz, DMSO-d_6) of (*R*)-**3Ap**

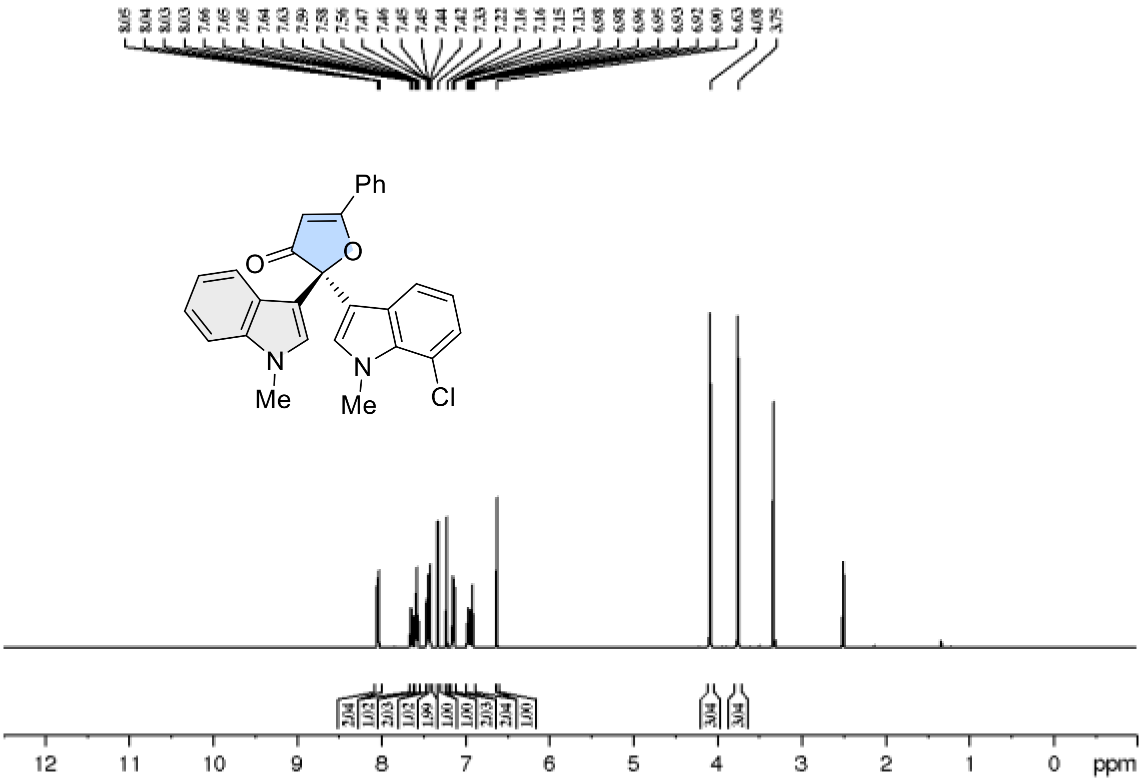

^{1}H NMR (500 MHz, DMSO-d_6) of (*R*)-**3Aq**

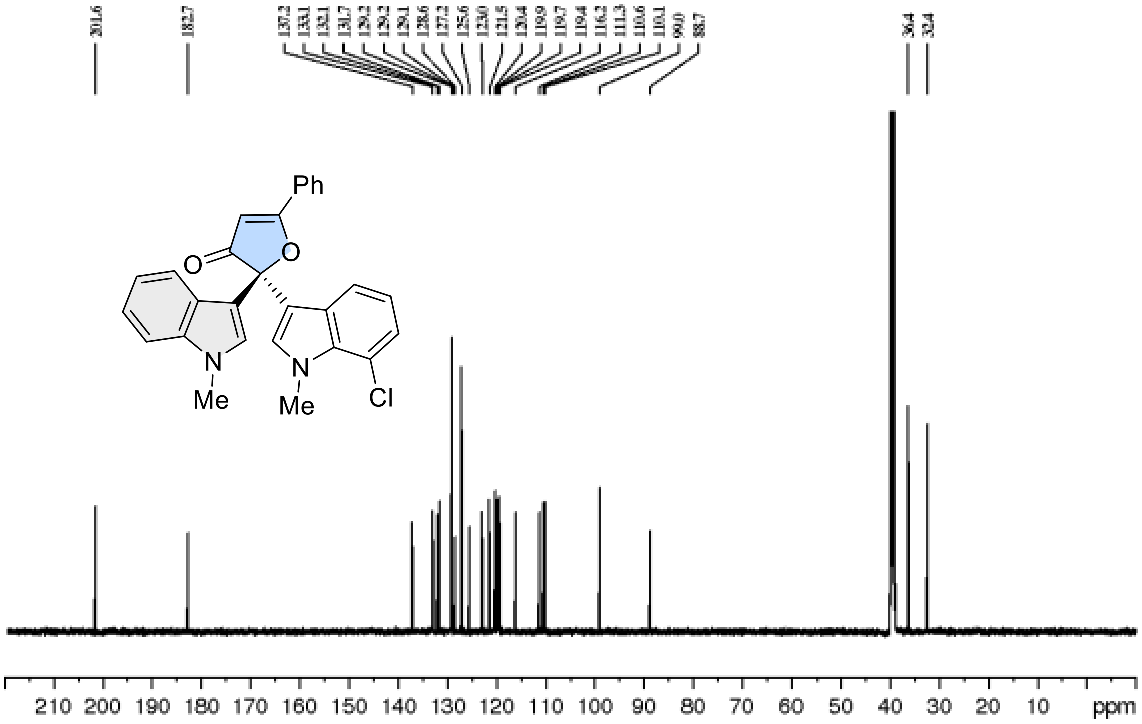

^{13}C NMR (126 MHz, DMSO-d_6) of (*R*)-**3Aq**

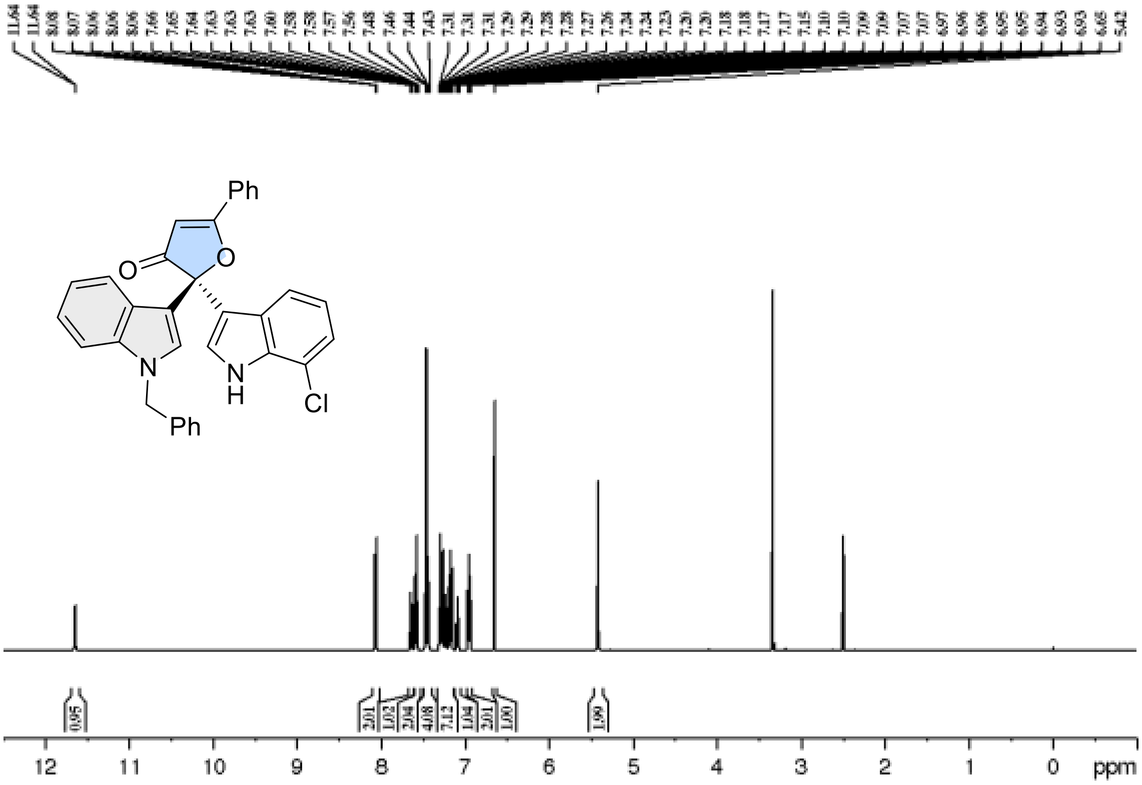

^{1}**H NMR** (500 MHz, DMSO-d_6) of (*S*)-**3En**

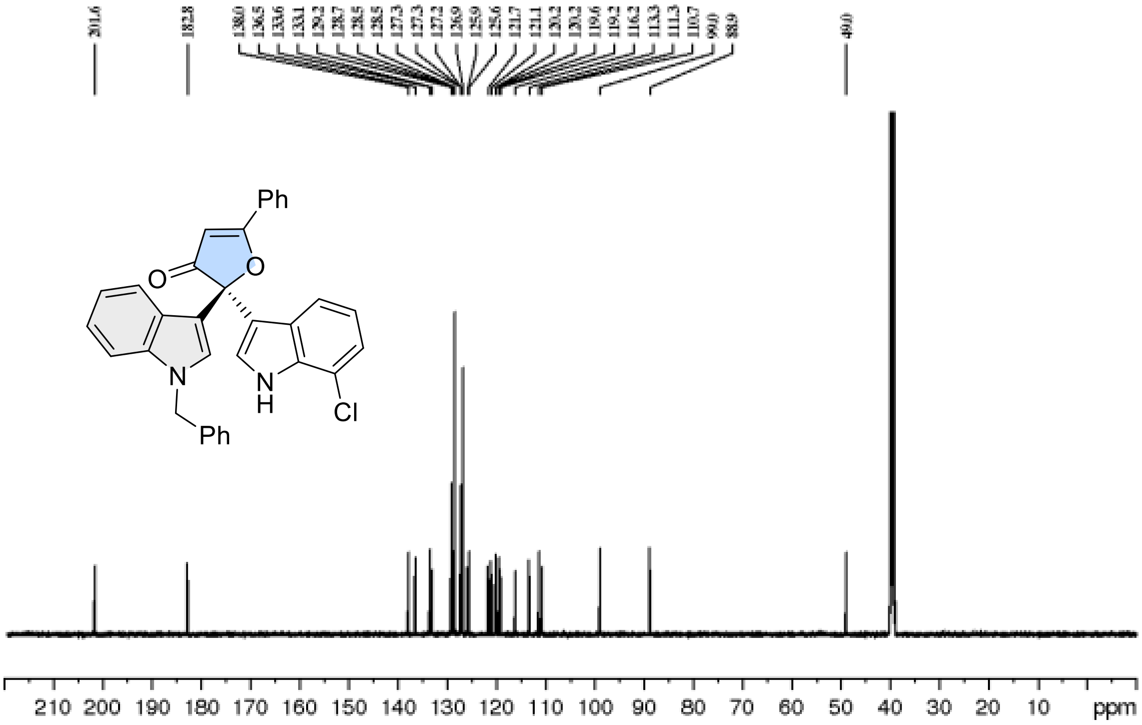

^{13}**C NMR** (126 MHz, DMSO-d_6) of (*S*)-**3En**

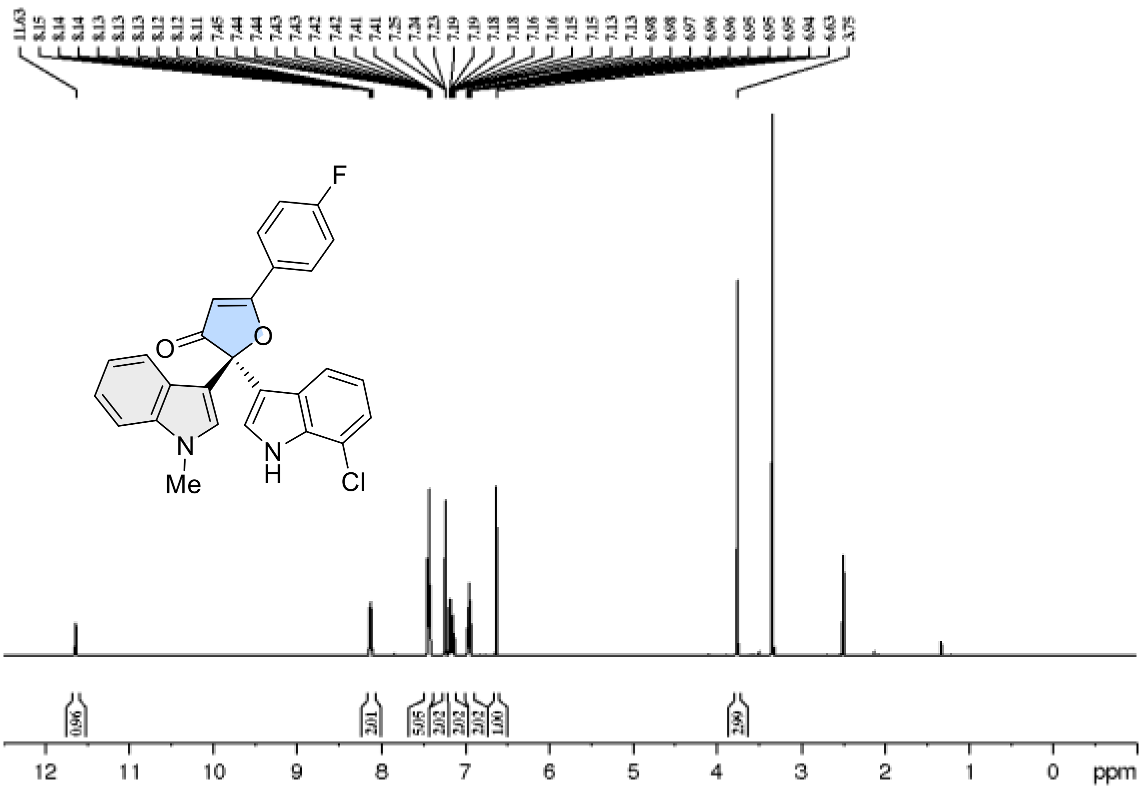

^{1}H NMR (500 MHz, DMSO-d_6) of (*S*)-**3Hn**

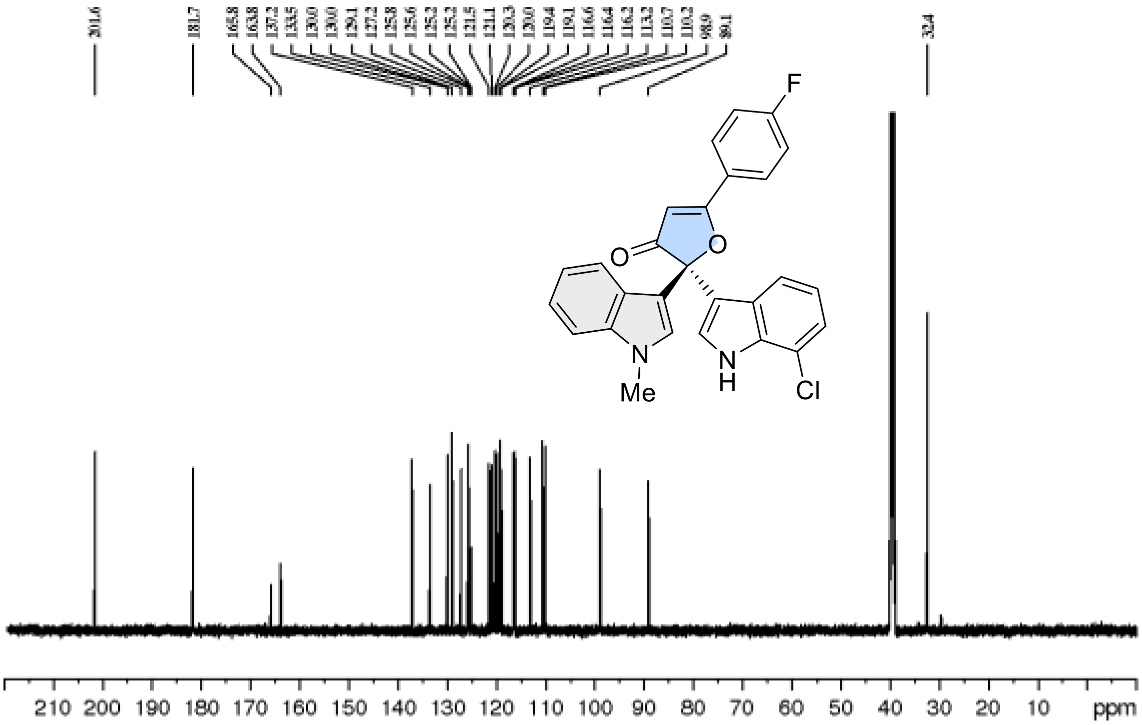

^{13}C NMR (126 MHz, DMSO-d_6) of (*S*)-**3Hn**

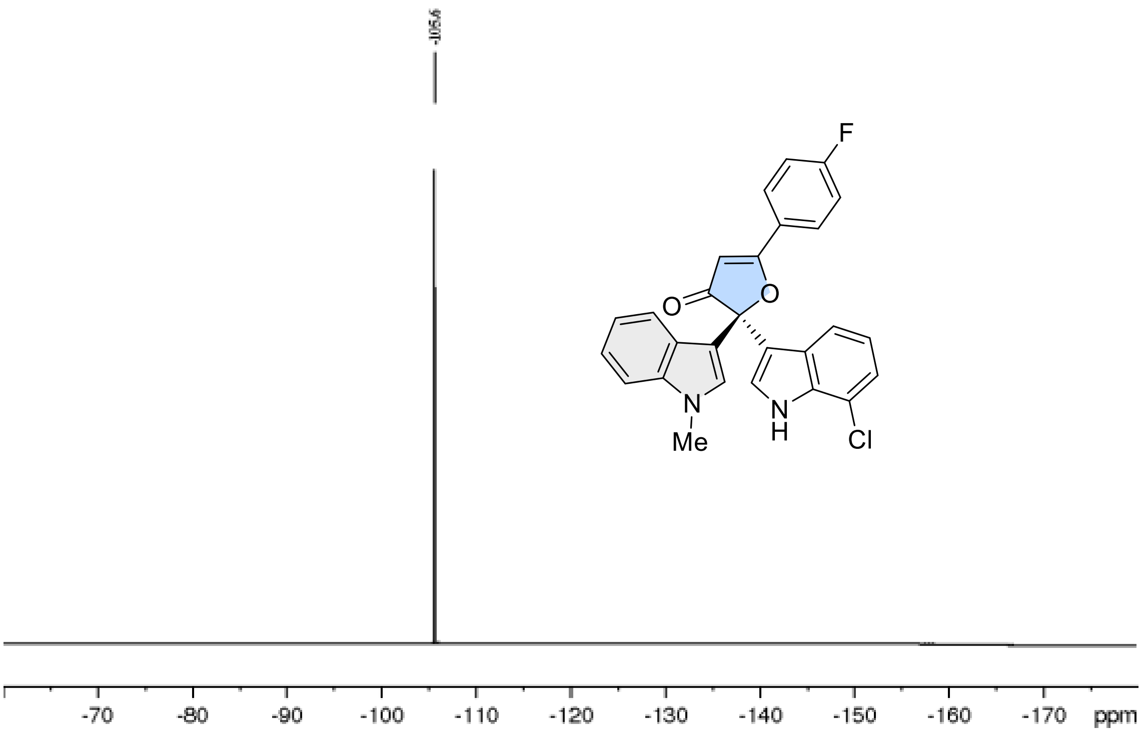

^{19}F NMR (471 MHz, DMSO-d_6) of (*S*)-**3Hn**

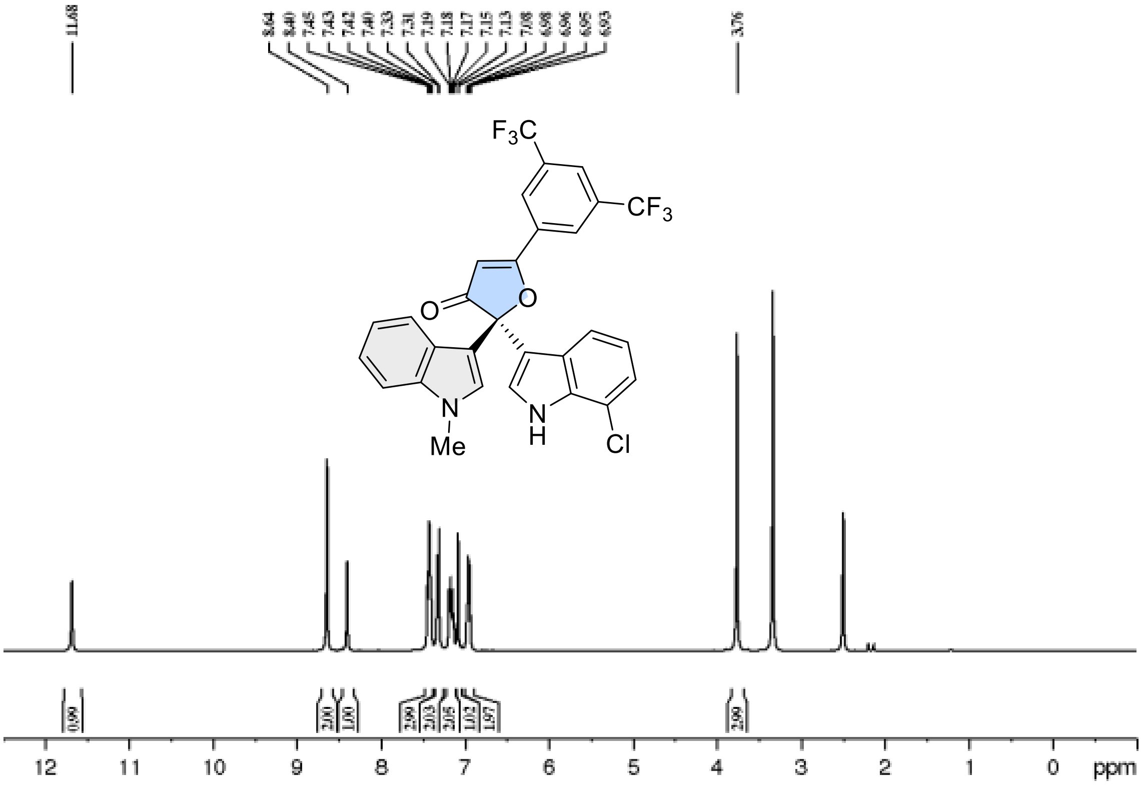

^{1}H NMR (500 MHz, DMSO-d_6) of (*S*)-**3In**

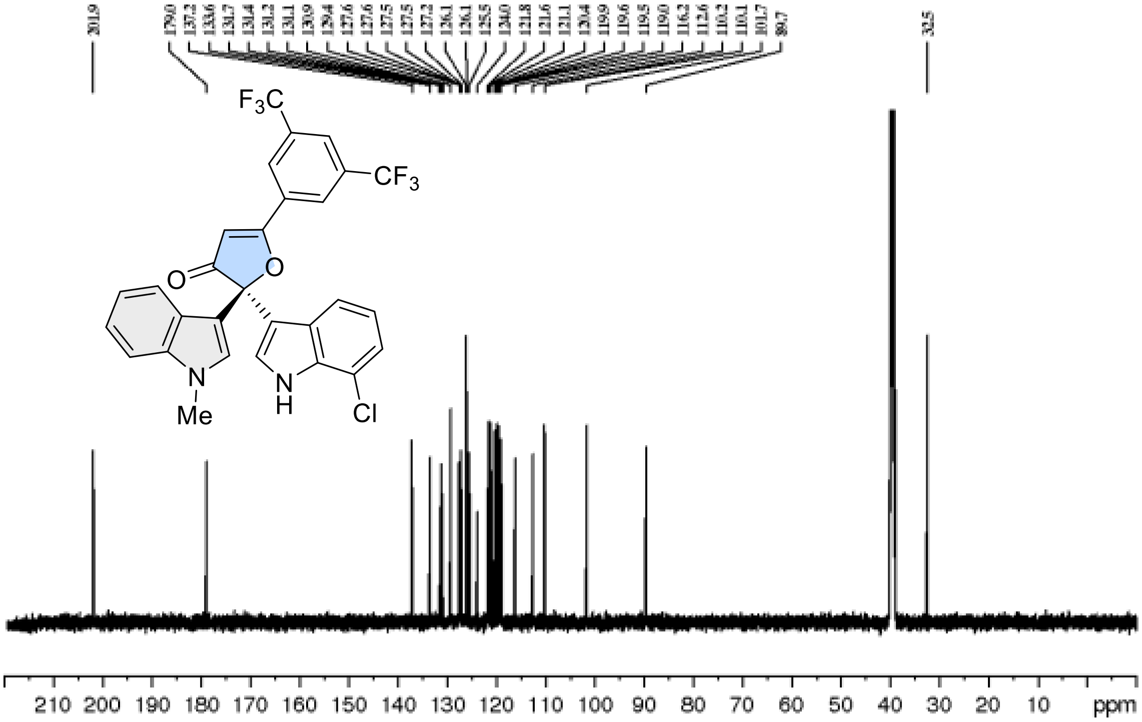

^{13}C NMR (126 MHz, DMSO-d_6) of (*S*)-**3In**

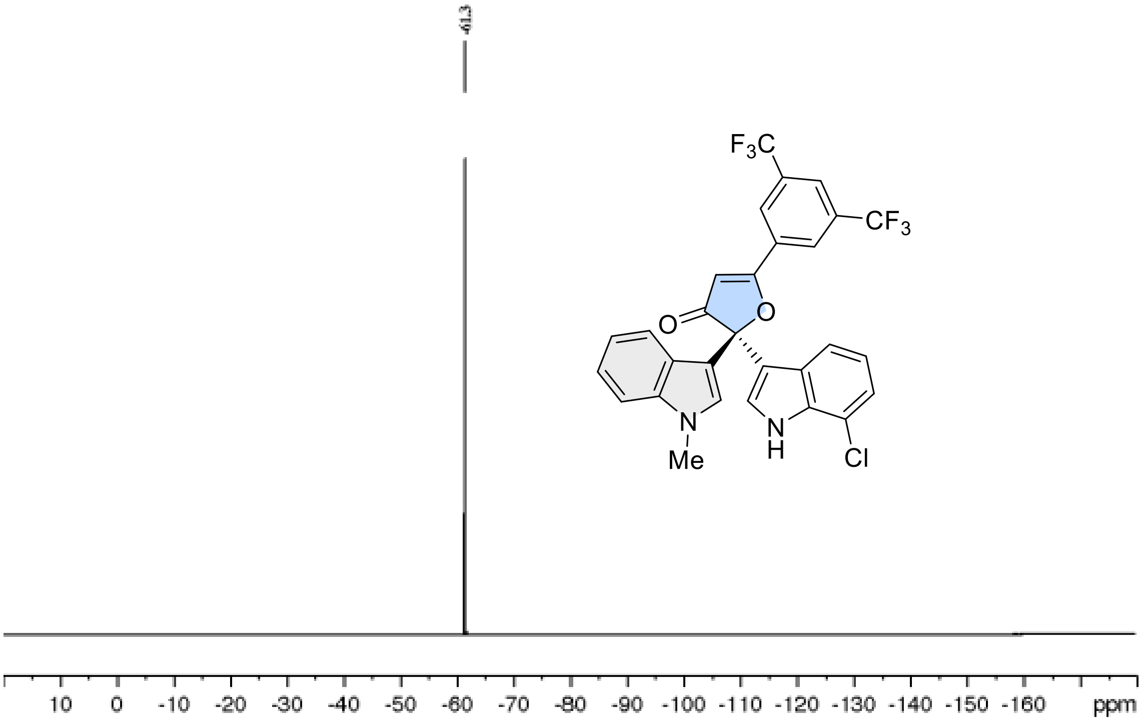

^{19}F NMR (471 MHz, DMSO-d_6) of (*S*)-**3In**

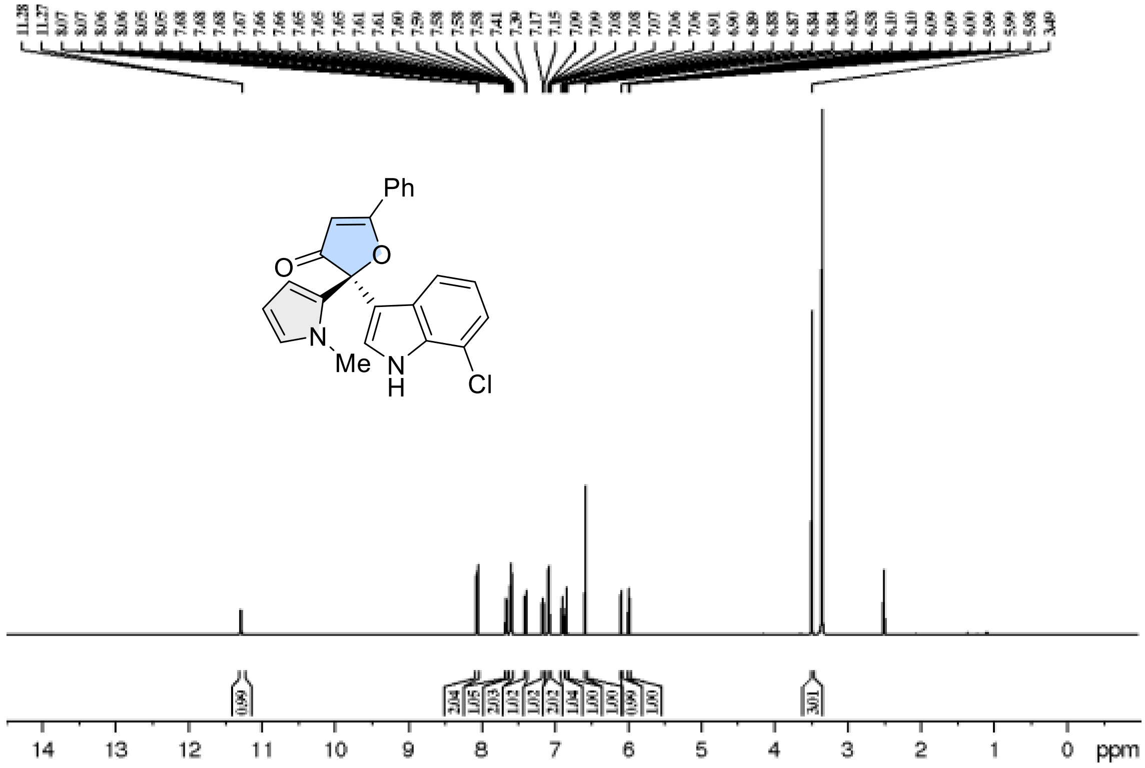

^{1}H NMR (500 MHz, DMSO-d_6) of (*S*)-**3Bn**

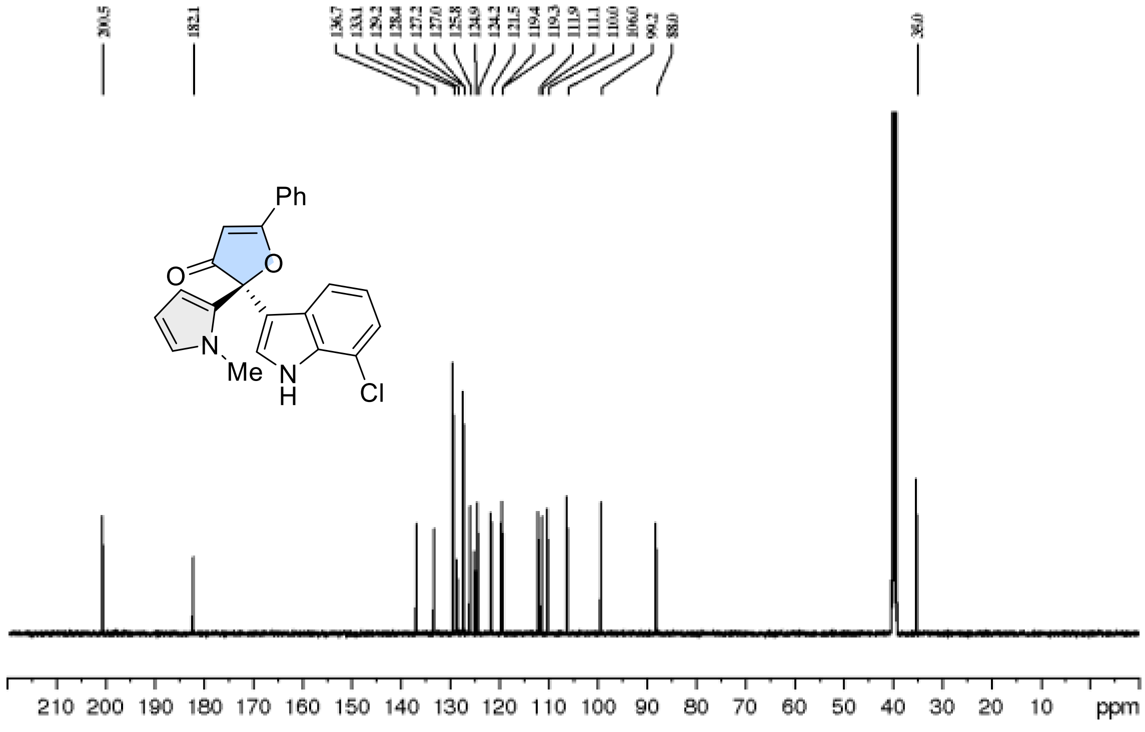

^{13}C NMR (126 MHz, DMSO-d_6) of (*S*)-**3Bn**

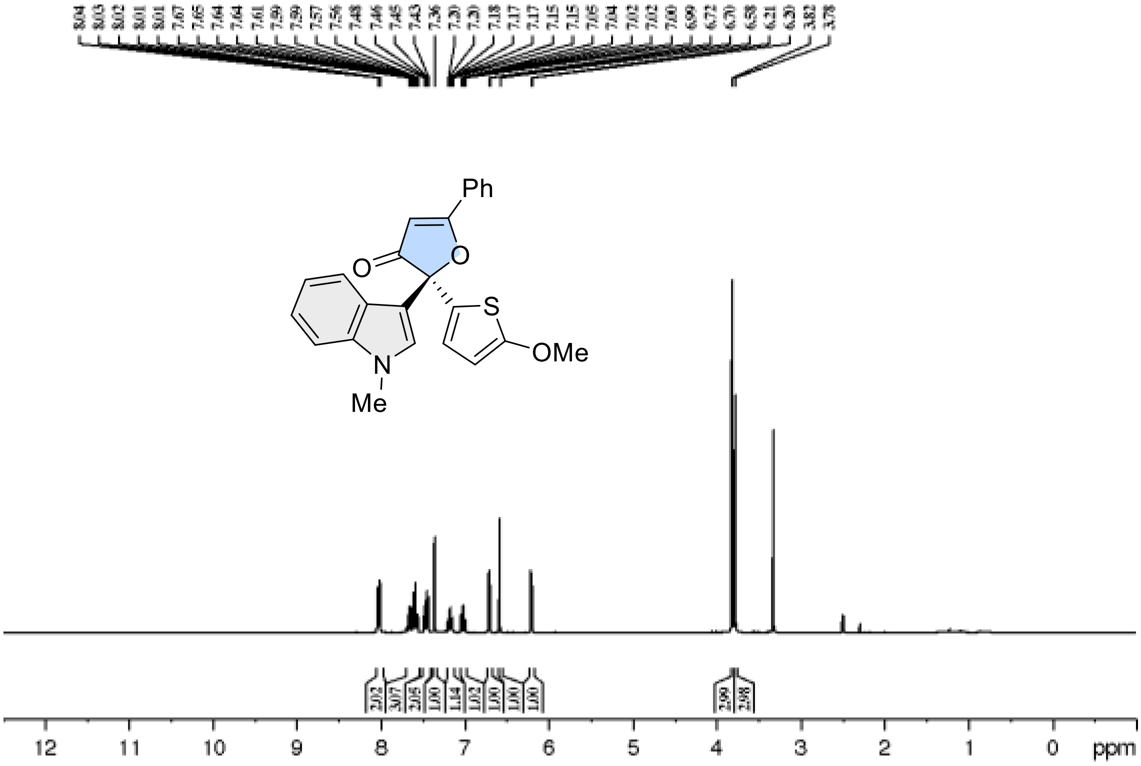

^{1}H NMR (500 MHz, DMSO-d_6) of (*S*)-**3Ar**

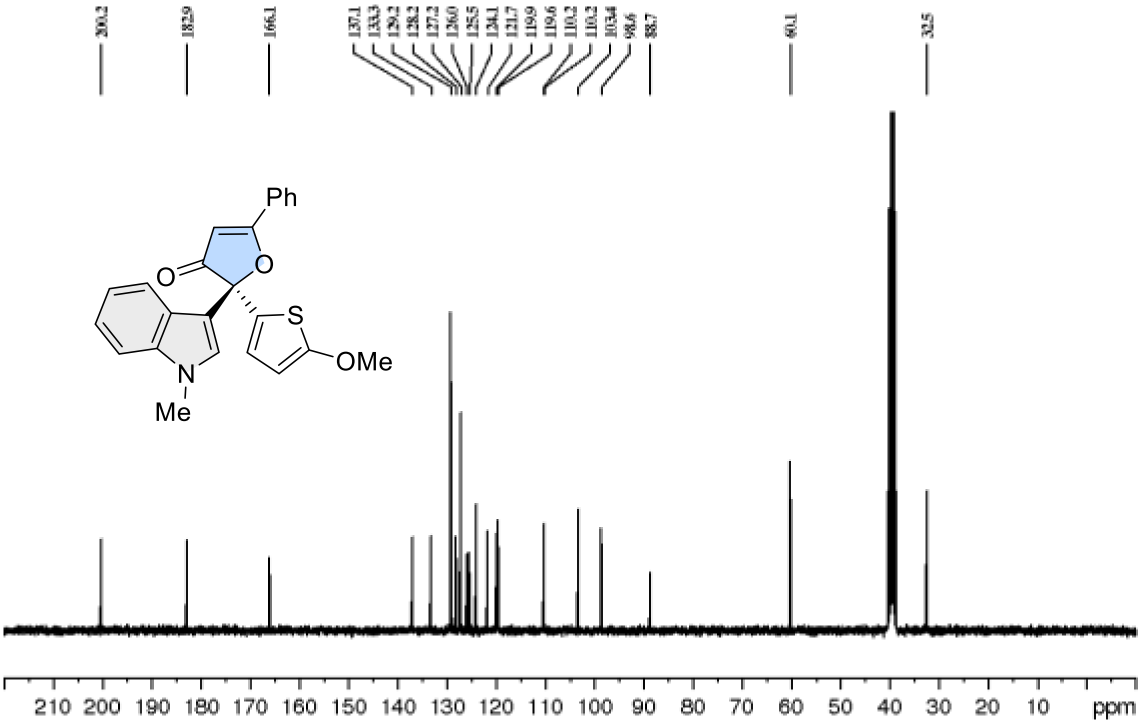

^{13}C NMR (126 MHz, DMSO-d_6) of (*S*)-**3Ar**
