# Supplementary figures and images for: Hydrogen-Bonding Activation of Gold(I) Chloride Complexes: Enantioselective Synthesis of 3(2H)-Furanones by a Cycloisomerization-Addition Cascade
Source: Org Lett. 2024 Jul 11;26(28):5995–6000. doi: 10.1021/acs.orglett.4c02091 (PMC11267603; doi:10.1021/acs.orglett.4c02091)

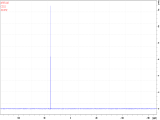

Supplement: Supplementary file 2 — ol4c02091_si_002.zip [file ol4c02091_si_002.zip › FID for Publication/Gold(I) Chloride Complexes/Au3/31P NMR/pdata/1/thumb.png]

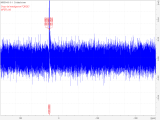

Supplement: Supplementary file 2 — ol4c02091_si_002.zip [file ol4c02091_si_002.zip › FID for Publication/Gold(I) Chloride Complexes/Au5/31P NMR/pdata/1/thumb.png]
