# Supplementary material for: Hydrogen-Bonding Activation of Gold(I) Chloride Complexes: Enantioselective Synthesis of 3(2H)-Furanones by a Cycloisomerization-Addition Cascade
Source: Org Lett. 2024 Jul 11;26(28):5995–6000. doi: 10.1021/acs.orglett.4c02091 (PMC11267603; doi:10.1021/acs.orglett.4c02091)
Supplement: Supplementary file 2 — ol4c02091_si_002.zip [file ol4c02091_si_002.zip › FID for Publication/Spectrometer specifications.docx]

**Spectrometer specifications**

**-**Manufacturer of spectrometers used to collect the data: **BRUKER**

-Acquisition software and processing programs: processing programs are integrated into software for acquisition. The software versions used for acquisition and data analysis were **TopSpin 3.7** and **TopSpin 4.3**

-Spectrometers: **Bruker Avance NEO 300** and **Bruker Avance NEO 500**

-Field strength used to measure each nucleus: **300 MHz** and **500 MHz** for **^1^H NMR**, **75.5 MHz** and **126 MHz** for **^13^C NMR**, **282 MHz** and **471 MHz** for **^19^F NMR**, **121.5 MHz** and **202.5 MHz** for **^31^P NMR**
