# Supplementary material for: Hydrogen-Bonding Activation of Gold(I) Chloride Complexes: Enantioselective Synthesis of 3(2H)-Furanones by a Cycloisomerization-Addition Cascade
Source: Org Lett. 2024 Jul 11;26(28):5995–6000. doi: 10.1021/acs.orglett.4c02091 (PMC11267603; doi:10.1021/acs.orglett.4c02091)
Supplement: Supplementary file 2 — ol4c02091_si_002.zip [file ol4c02091_si_002.zip › FID for Publication/Ynedione Substrates/Ynedione 1I/13C NMR/10/pdata/1/email_MBN-642C_10_1.pdf]

Grupo de Investigacion FQM263
MBN-642C

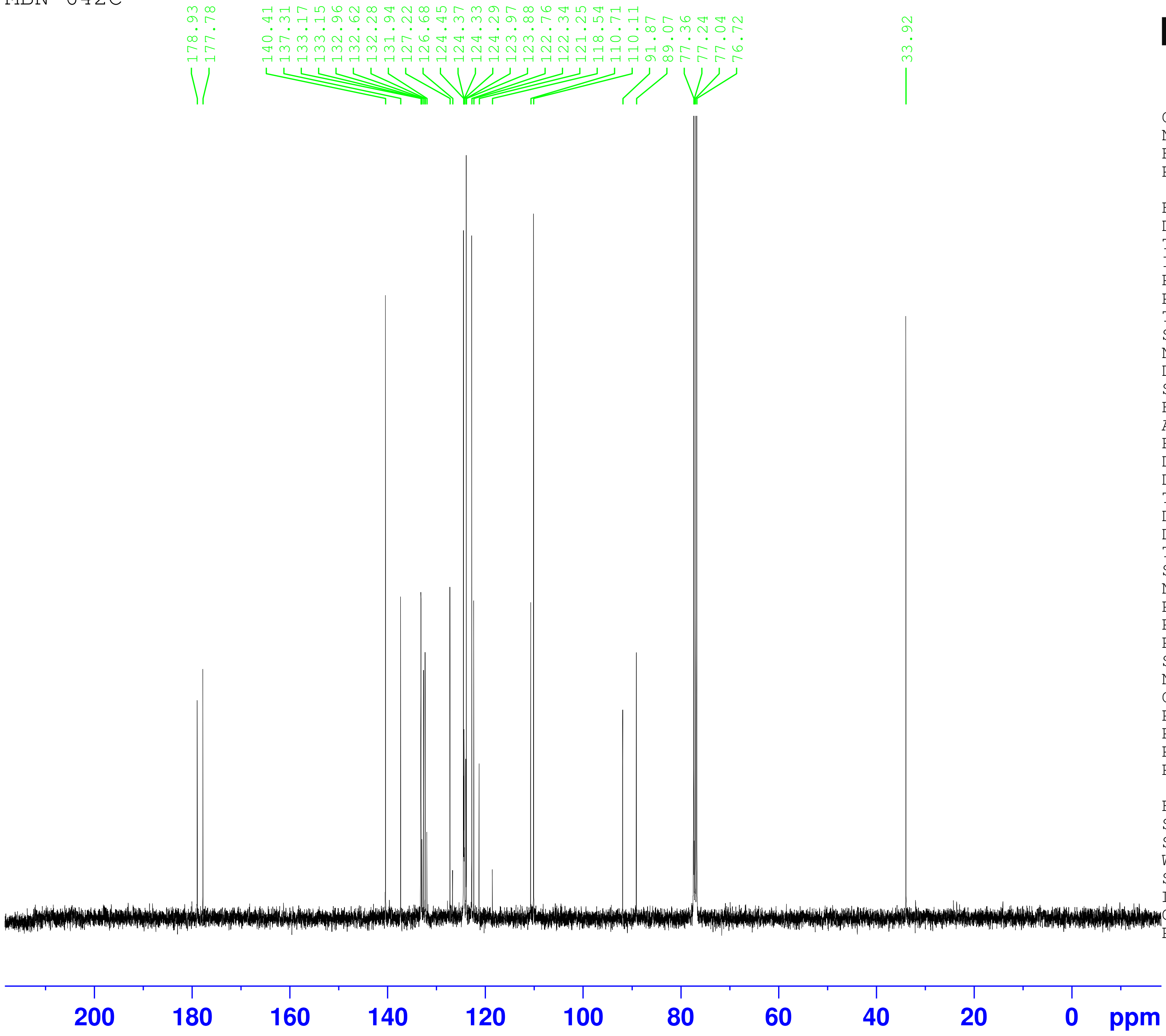

Current Data Parameters

NAME	MBN-642C
EXPNO	10
PROCNO	1

F2 - Acquisition Parameters

Date_	20240108
Time	16.08 h
INSTRUM	Avance
PROBHD	Z175994_0010 (
PULPROG	zgpg30
TD	65536
SOLVENT	CDCl3
NS	1024
DS	4
SWH	23809.523 Hz
FIDRES	0.726609 Hz
AQ	1.3762560 sec
RG	101
DW	21.000 usec
DE	12.00 usec
TE	298.0 K
D1	2.00000000 sec
D11	0.03000000 sec
TD0	1
SFO1	100.6303741 MHz
NUC1	13C
P0	5.00 usec
P1	15.00 usec
PLW1	77.41400146 W
SFO2	400.1616006 MHz
NUC2	1H
CPDPRG[2	waltz65
PCPD2	90.00 usec
PLW2	11.53999996 W
PLW12	0.09118000 W
PLW13	0.04586300 W

F2 - Processing parameters

SI	32768
SF	100.6203120 MHz
WDW	EM
SSB	0
LB	1.00 Hz
GB	0
PC	1.40
